# A Para‐Selective Kolbe–Schmitt Reaction

**DOI:** 10.1002/anie.202522503

**Published:** 2025-12-12

**Authors:** Xia Liu, Gregory J. P. Perry, Duanyang Kong

**Affiliations:** ^1^ State Key Laboratory of Chemical Resource Engineering Beijing University of Chemical Technology Beijing 100029 China; ^2^ School of Chemistry and Chemical Engineering University of Southampton Southampton SO17 1BJ UK

**Keywords:** Carboxylation, CO_2_ transfer, Dual‐function reagent, Isotope labeling, Kolbe–Schmitt

## Abstract

The Kolbe–Schmitt reaction is the archetypal carboxylation reaction in organic synthesis yet generally suffers from the requirement of high temperatures and CO_2_ pressures. We report a Kolbe–Schmitt‐type carboxylation of phenols using near equimolar amounts of a CO_2_ source at relatively low temperatures. The reaction uses the cesium salt of triphenylacetic acid as a combined source of base and CO_2_. Whereas the traditional Kolbe–Schmitt reaction provides products of ortho carboxylation (salicylic acids), this method, which employs cesium salts, delivers high para selectivity to give 4‐hydroxybenzoic acids. With the advantage of using near stoichiometric amounts of the carboxylating reagent, we demonstrate a practical and efficient preparation of ^13^C‐labeled 4‐hydroxybenzoic acid derivatives, thereby opening opportunities for applying Kolbe–Schmitt‐type chemistry in the area of carbon isotope labeling.

## Graphical abstract

Discovered over 150 years ago, the Kolbe–Schmitt carboxylation has established itself as one of the most well‐known chemical reactions. However, the process often requires extreme pressures and high temperatures. Here, we present a unique para‐selective Kolbe–Schmitt‐type carboxylation that proceeds at relatively low temperatures and uses near equimolar quantities of a carboxylating agent.

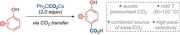

The reaction of phenols with carbon dioxide—the Kolbe–Schmitt reaction—has been known for over 150 years and sits as one of the most important and well‐recognized carboxylation reactions (Scheme 1a).^[^ ^1^, ^2^, ^3^ ^]^ The reaction is of high industrial relevance as it takes commodity chemicals and transforms them into hydroxybenzoic acids, which are key structures in food, pharmaceuticals, cosmetics, agrochemicals, and materials.^[^ ^4^, ^5^ ^]^ Indeed, this reaction has long been associated with the industrial production of 2‐hydroxybenzoic acid (salicylic acid), the precursor to aspirin.^[^ ^6^ ^]^ Despite a century having passed since its inception, the reaction remains relevant with few adaptations from the original report. At present, the current state‐of‐the‐art can be summarized as follows: 1) The reaction requires high temperatures (>120 °C) and high pressures of CO_2_ (>20 atm).^[^ ^1^, ^7^, ^8^, ^9^, ^10^, ^11^, ^12^, ^13^, ^14^, ^15^, ^16^, ^17^, ^18^, ^19^, ^20^, ^21^, ^22^, ^23^, ^24^, ^25^, ^26^, ^27^, ^28^, ^29^, ^30^, ^31^, ^32^, ^33^, ^34^ ^]^ In some cases, the temperature and pressure can be lowered, but these are generally select examples with electron‐rich phenols (e.g., resorcinol).^[^ ^35^, ^36^, ^37^, ^38^, ^39^, ^40^ ^]^ 2) The reaction is sensitive to water. This creates a practical issue as in many cases the phenoxide salt must be prepared and thoroughly dried before use, rather than conducting the reaction directly from the phenol.^[^ ^1^, ^41^ ^]^ 3) Sodium phenoxides generally give high ortho selectivity to provide salicylic acids. Potassium and cesium phenoxides generally provide mixtures of ortho/para carboxylation, though a preference towards para‐carboxylation is often observed.^[^ ^1^, ^42^, ^43^ ^]^ Whereas the ortho carboxylation has been well studied, a general method for para‐carboxylation is not available.^[^ ^44^ ^]^

**Scheme 1:**
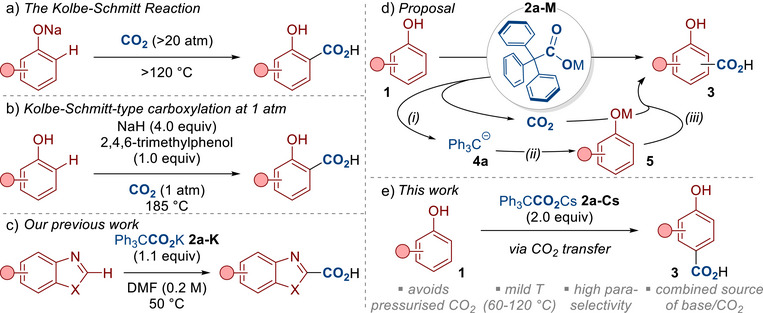
Previous Kolbe–Schmitt‐type reactions and dual‐function reagents for CO_2_ transfer.

Clearly, lowering the temperature and pressure of Kolbe–Schmitt‐type carboxylations would offer a significant improvement, particularly for laboratory scale reactions. In doing so, this would also present a more attractive method for phenol carboxylation within the area of carbon isotope labeling.^[^ ^45^, ^46^, ^47^, ^48^ ^]^ Whereas CO_2_ gas is an abundant and cheap chemical, labeled CO_2_ gases (e.g., ^13^CO_2_, ^14^CO_2_) are significantly more expensive and produced in much lower quantities.^[^ ^49^, ^50^, ^51^ ^]^ Thus, the need for excess/pressurized gases in standard Kolbe–Schmitt chemistry is less suited to isotope labeling where using equimolar amounts of the labeled reagent is a much greater necessity.

Larrosa and co‐workers have presented one of the most notable advancements of the Kolbe–Schmitt reaction (Scheme 1b).^[^ ^52^ ^]^ They revealed that the addition of 2,4,6‐trimethylphenol afforded an effective carboxylation of phenols under atmospheric pressure. However, high reaction temperatures (185 °C) and an excess amount of a strong base (NaH, 4.0 equiv) were required. The reaction also seemed sensitive to water, leading the authors to perform the reactions in a glovebox, thereby restricting the practicality of the procedure. Interestingly, the authors suggested that 2,4,6‐trimethylphenol enabled the process to proceed at low pressures by aiding CO_2_ capture and increasing the concentration of CO_2_ in the reaction mixture.

Our groups and others have recently reported the use of carboxylic acids/carboxylates as a vehicle for delivering CO_2_ (Scheme 1c).^[^ ^53^, ^54^, ^55^, ^56^, ^57^, ^58^ ^]^ We have revealed that the potassium salt of triphenylacetate **2a‐K** (easily prepared from commercially available triphenylacetic acid, CAS: 595–91–5) is particularly effective at CO_2_ transfer, allowing reactions to proceed under milder temperatures and pressures in comparison to related carboxylations. We coined the term “dual‐function reagent” to describe carboxylates **2a‐M** as they provided a combined source of CO_2_ and base for the reaction, delivering further practicality benefits by avoiding sensitive and difficult‐to‐handle reagents. We therefore questioned whether our reagents would offer a practical and milder alternative to the traditional Kolbe–Schmitt reaction (Scheme 1d). We proposed that dual function reagent **2a‐M** would undergo decarboxylation to provide the trityl anion **4a** and CO_2_ (step i). The phenoxides **5** would then be generated from the reaction between phenols **1** and the basic species **4a** (step ii). Capture of the in situ generated CO_2_ in a Kolbe–Schmitt‐type carboxylation would then lead to the hydroxybenzoic acid product **3** (step iii). Here, we reveal that cesium triphenylacetate **2a‐Cs** promotes an efficient Kolbe–Schmitt‐type carboxylation of phenols **1** (Scheme 1e). The reaction proceeds under relatively mild conditions to give 4‐hydroxybenzoic acids, thereby revealing a rare form of para‐selective Kolbe–Schmitt reactivity that can be performed at lower temperatures and pressures.

We began our study by subjecting phenol **1a** to our previously reported conditions, using potassium triphenylacetate **2a‐K** (Scheme 2). A promising yield of 34% of **3a‐Me** was observed (Entry 1), which was further improved by doubling the equivalents of the dual‐function reagent **2a‐K** (Entry 2). Further screening revealed the cesium salt **2a‐Cs** as the optimal carboxylating agent for this reaction, providing the product in excellent yield (Entry 3).^[^ ^59^ ^]^ Impressively, good reactivity was maintained at temperatures as low as 60 °C (Entry 4). We have therefore been able to demonstrate that a Kolbe–Schmitt‐type carboxylation can proceed under remarkably low pressures and temperatures. We are unsure of the exact reasons behind this improved efficiency, but we suggest the following: 1) CO_2_ is generated within the reaction mixture, whereas traditional Kolbe–Schmitt reactions require CO_2_ dissolution or solid–gas interactions, which create inherent barriers to reactivity. In this regard, DMF may also aid our process as it is an effective solvent for dissolving CO_2_.^[^ ^60^ ^]^ 2) Traces of water are removed by the carboxylate salt **2a**. The traditional Kolbe–Schmitt reaction is highly sensitive to moisture and extensive drying of alkali metal phenoxides is often a requirement for good reactivity.^[^ ^1^, ^41^ ^]^ In our process, the carboxylate **2a** delivers a self‐drying mechanism by reacting with any traces of water to produce an innocent by‐product, Ph_3_CH.^[^ ^61^ ^]^ This allows the process to be set up on the bench‐top without special apparatus or rigorous exclusion of water. We were also impressed by the high para‐selectivity in this process. It is known that larger counter cations, such as cesium, guide carboxylation to the para position, but this is a much rarer form of Kolbe–Schmitt‐type reactivity that often affords ortho/para mixtures and a general process is not currently available.^[^ ^1^, ^42^, ^43^, ^44^ ^]^ Finally, when using sodium triphenylacetate **2a‐Na**, high ortho selectivity was observed to provide the usual Kolbe–Schmitt salicylic acid derived product **3a′‐Me** (Scheme 2, Entry 5). Unfortunately, the yield of this process was relatively low, and we have been unable to improve the reaction yield. We believe this is due to the presence of water in these reactions, which is known to negatively affect the Kolbe–Schmitt reaction.^[^ ^1^, ^41^ ^]^ Whereas the potassium and cesium salts **2a‐K** and **2a‐Cs** provided clean elemental analysis readings, water was proposed as an impurity with **2a‐Na**, suggesting the sodium salt is hygroscopic.^[^ ^53^, ^62^ ^]^

**Scheme 2:**
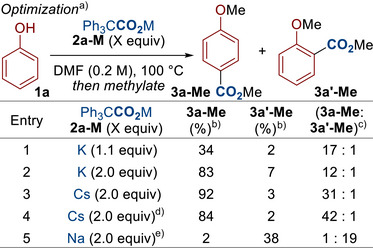
Optimization. ^a)^ Conditions: **1a** (0.2 mmol), **2a‐M** (0.22 – 0.4 mmol), DMF (1.0 mL, 0.2 M), 100 °C, N_2_, 12 h. Then MeI (8.0 equiv), 40 °C, 4 h. ^b)^ Yield was determined by GC using 1,2,4,5‐tetramethylbenzene as the internal standard. ^c)^ Selectivity was determined by GC using 1,2,4,5‐tetramethylbenzene as the internal standard. ^d)^ The carboxylation was carried out at 60 °C. ^e)^ The carboxylation was carried out at 140 °C. DMF = *N*,*N*‐Dimethylformamide.

Regarding the mechanism, previous reports suggest that para‐carboxylation is the result of initial ortho‐carboxylation, followed by rearrangement to the para‐product (see **I1**) rather than a direct para carboxylation (see **I2**, Scheme 3a). High temperatures and “low” pressures of CO_2_ (e.g., 230 °C, 5 atm) were also reported to deliver para‐carboxylation.^[^ ^63^, ^64^ ^]^ Although skeptical of an ortho‐to‐para rearrangement occurring under the relatively low temperatures that our reaction was performed at, we thought it pertinent to investigate this possibility. We therefore subjected salicylic acid **3a′** to our standard reaction conditions to see if rearrangement to the para product **3a** was possible (Scheme 3b). We speculated that, upon heating, salicylate **I1** would be generated alongside 2 equiv of CO_2_ and the side product Ph_3_CH. This represents the same mixture that would theoretically form under our standard reaction conditions, the only difference being that an extra equivalent of CO_2_ would be present in this mechanistic experiment. Under these conditions, rearrangement to the para product **3a‐Me** was not detected and only recovery of the salicylic acid derivative **3a′‐Me** occurred. We therefore believe that direct carboxylation at the para‐position is occurring, rather than the rearrangement pathway that has been previously proposed. We also monitored the reaction to see if the ortho‐carboxylated product **3a′‐Me** is the major isomer at low conversion, but that it rearranges to the para‐carboxylated product **3a‐Me** as the reaction progresses (Scheme 3c). In all cases, the para‐carboxylated product **3a‐Me** was the major isomer, further supporting a mechanism of direct para‐carboxylation. We tentatively propose that, unlike the classical Kolbe–Schmitt reaction using sodium phenoxides, the cesium ion is less effective at directing carboxylation to the ortho position. This causes carboxylation to occur at the least sterically hindered para‐position, in line with classical electrophilic aromatic substitution‐type reactivity. Further studies are required to better delineate this impressive selectivity. Finally, to provide evidence of our proposed mechanism (Scheme 1d), we subjected the deuterated phenol **1a‐D** to the standard reaction conditions (Scheme 3d). This delivered the expected product **3a‐D** alongside the deuterated side product **4a‐D**, supporting the proposed deprotonation event via intermediate **4a** (*c.f*. Scheme 1d, step ii).

**Scheme 3:**
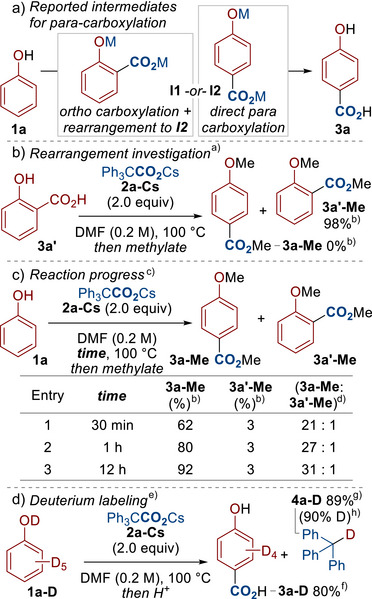
Mechanistic Studies. ^a)^ Conditions: **3a′** (0.2 mmol), **2a‐Cs** (0.4 mmol), DMF (1.0 mL, 0.2 M), 100 °C, N_2_, 12 h. Then MeI (8.0 equiv), 40 °C, 4 h. ^b)^ Yield was determined by GC using 1,2,4,5‐tetramethylbenzene as the internal standard. ^[c]^ Conditions: **1a** (0.2 mmol), **2a‐Cs** (0.4 mmol), DMF (1.0 mL, 0.2 M), 100 °C, N_2_, 30 min, 1 h or 12 h. Then MeI (8.0 equiv), 40 °C, 4 h. ^d)^ Selectivity was determined by GC using 1,2,4,5‐tetramethylbenzene as the internal standard. ^e)^ Conditions: **1a‐D** (0.2 mmol), **2a‐Cs** (0.4 mmol), DMF (1.0 mL, 0.2 M), 100 °C, N_2_, 12 h. Then acidic work up. ^f)^ Yield was determined by GC using 1,2,4,5‐tetramethylbenzene as the internal standard. Yield is with respect to **1a‐D**. ^g)^ Isolated yield. Yield is with respect to **2a‐Cs**. ^h)^ Deuterium incorporation determined by ^1^H NMR. DMF = *N*,*N*‐Dimethylformamide.

With a method for the para‐selective carboxylation of phenols **1** in hand, we looked to demonstrate the scope of this reaction (Schemes 4 and 5). The reaction was first applied to a range of 2‐substituted and 2,3‐disubstituted phenols to provide the para carboxylated products with high selectivity in all cases (**3a**–**3v**). In some cases, we obtained X‐ray crystal structures to confirm the para/ortho selectivity.^[^ ^65^ ^]^ The scope of the reaction was also impressive, showing tolerance to electron donating and electron withdrawing functionalities, including alkenes (**3h**), halogens (**3m**–**3p**) and heterocyclic scaffolds (**3q**–**3u**). 2,5‐ and 2,6‐disubstituted phenols also reacted well under the standard conditions (**3w**–**3z**). We note that in several cases impressive yields were observed even when conducting the reaction at temperatures as low as 60 °C (see **3j**, **3r**, **3s**, **3ab**), or reducing the equivalents of the carboxylating agent **2a‐Cs** (see **3z**, **3ac′**, **3ag′**‐**3ai′**), highlighting the mild and practical conditions we have developed for Kolbe–Schmitt‐type carboxylation. In a few cases trisubstituted phenols were added to aid the reactivity, in accordance with the report by Larrosa and co‐workers (see **3m**–**3p**, **3af′**).^[^ ^52^ ^]^ Interestingly, differing selectivity was observed when using 3‐substituted phenols, for example, small substituents in the 3‐position showed para‐selectivity (**3aa**), whereas larger substituents diverted carboxylation to the ortho position (**3ac′**‐**3ag′**). Finally, if the para‐position is blocked, ortho‐carboxylated products were formed (**3ah′**‐**3aj′**). Overall, high para‐selectivity was observed in all cases, except when the para position was sterically hindered (**3ac′**‐**3ag′**) or was blocked entirely (**3ah′**‐**3aj′**). Unsuccessful examples (**3ak**‐**3aq**) are shown at the bottom of Scheme 5 to illustrate the current limitations of our procedure and further details are included in the supporting information.

**Scheme 4:**
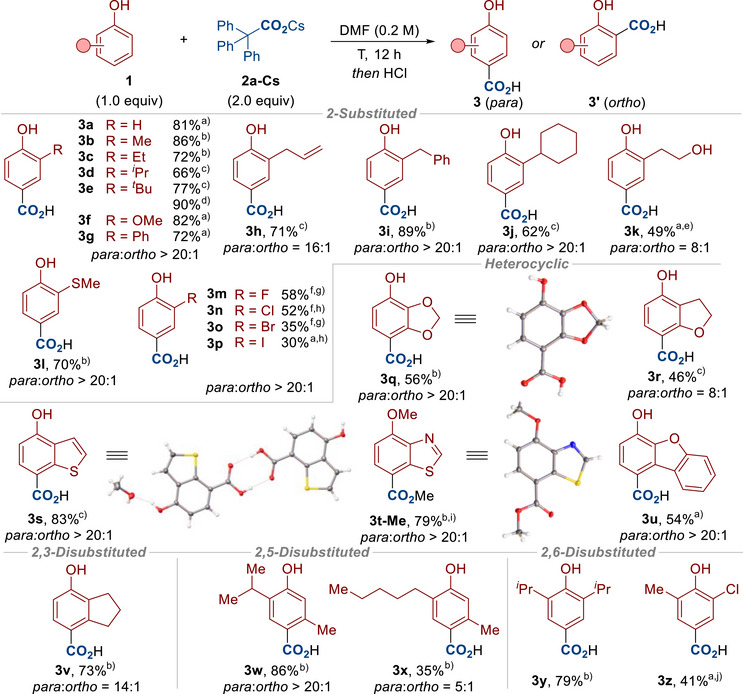
Reaction Scope. Conditions: **1** (0.2 mmol), **2a‐Cs** (0.4 mmol), DMF (1.0 mL, 0.2 M), T (°C), N_2_, 12 h. Then acidic workup *or* MeI (8.0 equiv), 40 °C, 4 h. DMF = *N,N*‐Dimethylformamide. Note: The ratio of para to ortho (*para*:*ortho*) was determined by ^1^H‐NMR analysis of the crude reaction mixture. ^a)^ The carboxylation was carried out at 100 °C. ^b)^ The carboxylation was carried out at 80 °C. ^c)^ The carboxylation was carried out at 60 °C. ^d)^ The carboxylation was carried out with **2a‐K** (2.0 equiv) at 140 °C. ^e)^ 3.0 equiv of **2a‐Cs** was used. ^f)^ The carboxylation was carried out at 120 °C. ^g)^ 1.0 equiv of 2,4,6‐trimethylphenol was added. ^h)^ 1.0 equiv of 2,6‐di‐*tert*‐butyl‐4‐methoxyphenol was added. ^i)^ The product was isolated after methylation. ^j)^ 1.5 equiv of **2a‐Cs** was used.

**Scheme 5:**
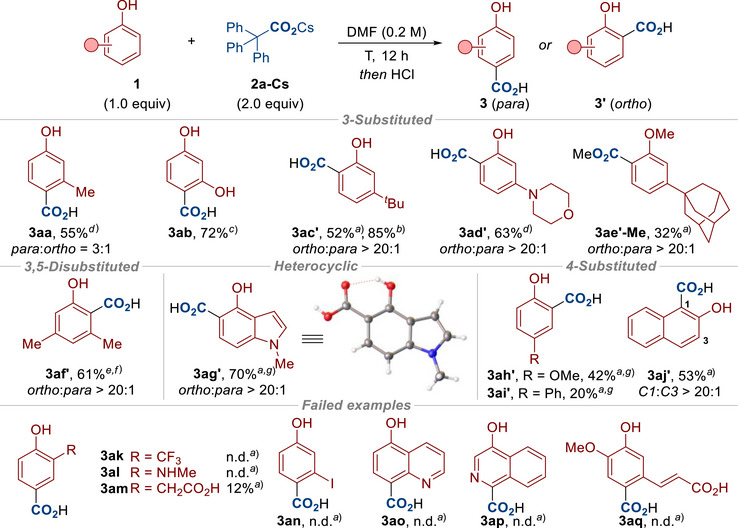
Reaction Scope (continued). Conditions: **1a** (0.2 mmol), **2a‐Cs** (0.4 mmol), DMF (1.0 mL, 0.2 M), T (°C), N_2_, 12 h. Then acidic workup *or* MeI (8.0 equiv), 40 °C, 4 h. DMF = *N,N*‐Dimethylformamide. Note: The ratio of para to ortho (*para*:*ortho*) was determined by ^1^H‐NMR analysis of the crude reaction mixture. ^a)^ The carboxylation was carried out at 100 °C. ^b)^ The carboxylation was carried out with **2a‐K** (1.1 equiv) at 140 °C. ^c)^ The carboxylation was carried out at 60 °C. ^d)^ The carboxylation was carried out at 80 °C. ^e)^ The carboxylation was carried out at 120 °C. ^f)^ **1.0 equiv of 2,6‐di‐tert‐butyl‐4‐methoxyphenol was added. ^g)^ 1.0 equiv of **2a‐Cs** was used.

To demonstrate the utility of the 4‐hydroxybenzoic acid products, we submitted several products to further transformations (Scheme 6a). For example, nitration of 4‐hydroxybenzoic acid **3a** provided compound **6**, which is a known intermediate toward local anesthetic medicine orthocaine **7**.^[^ ^66^ ^]^ The thioether‐bearing hydroxybenzoic acid was oxidized to the sulfone **8**. Finally, the allyl substituted compound **3h** underwent cyclization with diphenyl diselenide to provide the dihydrobenzofuran **9**, which is a derivative of medicines for treating Alzheimer's and Parkinson's disease.^[^ ^67^, ^68^ ^]^

**Scheme 6:**
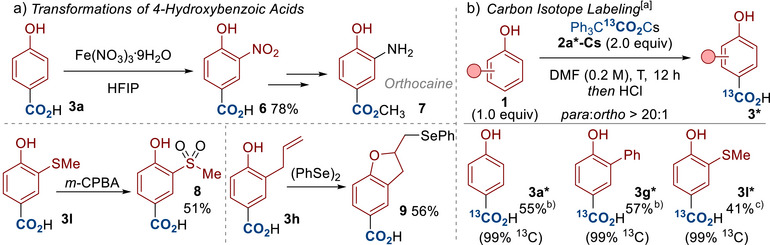
Transformations of 4‐hydroxybenzoic acids and carbon isotope labeling. ^a)^ Conditions: **1** (0.1 mmol), 2a*‐Cs (0.2 mmol), DMF (0.5 mL, 0.2 M), T (°C), N_2_, 12 h. Then acidic workup. DMF = *N,N*‐Dimethylformamide. Note: The ratio of para to ortho (*para*:*ortho*) was determined by ^1^H‐NMR analysis of the crude reaction mixture. ^b)^ The carboxylation was carried out at 100 °C. ^c)^ The carboxylation was carried out at 80 °C.

A key advantage of this adapted Kolbe–Schmitt reaction is the ability to conduct carboxylation with only 2 equiv of the carboxylating agent **2a‐Cs**. Isotope chemistry is of great importance in academia and industry, for example, labeled compounds are vital for absorption, distribution, metabolism, and elimination (ADME) studies in the development of medicines and agrochemicals.^[^ ^45^, ^46^, ^47^, ^48^ ^]^ Carboxylation is one of the key methods for installing carbon isotope labels, such as ^13^C and ^14^C, but there are relatively few examples of using the Kolbe–Schmitt reaction in isotope labeling.^[^ ^69^, ^70^ ^]^ This might be due to the high pressures/excesses of CO_2_ gas required for Kolbe–Schmitt chemistry, which are incompatible with costly and sometimes hazardous labeled reagents (for example ^14^CO_2_ is radioactive and costs >£1000 mmol^−1^).^[^ ^49^, ^50^, ^51^ ^]^ Indeed, most methods for preparing labeled hydroxybenzoic acids proceed through alternative routes involving multiple steps and metalation chemistry.^[^ ^71^ ^]^ Scheme 6b demonstrates the application of our method in the preparation of several ^13^C‐labeled 4‐hydroxybenzoic acids. We believe this presents an effective and practical method that will rekindle interest towards implementing the Kolbe–Schmitt reaction in carbon isotope labeling, which is an area of vital importance in the pharmaceutical and agrochemical industries.

We have developed a carboxylation of phenols that avoids the high temperatures and pressures that are commonly encountered in Kolbe‐Schmitt‐type reactions. The reaction proceeds efficiently at relatively low temperatures and with near equimolar amounts of the carboxylating agent. Interestingly, by employing a cesium carboxylate as a CO_2_ transfer reagent the reaction displays high para selectivity to give 4‐hydroxybenzoic acids. Mechanistic studies suggest that this is a result of direct para‐carboxylation, rather than a rearrangement process that has been previously postulated. The reaction has been applied to a variety of phenols to deliver important 4‐hydroxybenzoic acid scaffolds. Finally, we display the potential of this method for effective isotope labeling via Kolbe–Schmitt‐type carboxylation. We believe this represents one of the most accessible methods for performing Kolbe–Schmitt‐type chemistry while also delivering unique examples of the para‐selective carboxylation of phenols.

## Supporting Information

The data supporting this article is included in the Supporting Information. Small molecule crystal data is deposited with the Cambridge Crystallographic Data Centre (CCDC) with deposition numbers 2488331 (for **3q**), 2488328 (for **3s**), 2488327 (for **3t‐Me**), 2488332 (for **3ad′**), and 2488330 (for **3ag′**).

## Conflict of Interests

The authors declare no conflict of interest.

## Supporting information



Supporting Information

Supporting Information

## Data Availability

The data that support the findings of this study are available in the Supporting Information of this article.
