# Supplementary material for: A Para‐Selective Kolbe–Schmitt Reaction
Source: Angew Chem Int Ed Engl. 2025 Dec 12;65(4):e22503. doi: 10.1002/anie.202522503 (PMC12828480; doi:10.1002/anie.202522503)
Supplement: Supplementary file 1 — Supporting Information [file ANIE-65-e22503-s001.pdf]

Supplementary Information

A Para-Selective Kolbe-Schmitt Reaction

Xia Liu,[1] Gregory J. P. Perry,[2,*] and Duanyang Kong[1,*]

[1] State Key Laboratory of Chemical Resource Engineering, Beijing University of Chemical Technology, Beijing 100029, China

[2] School of Chemistry and Chemical Engineering, University of Southampton, Southampton, SO17 1BJ (UK)

*Correspondence: gregory.perry@soton.ac.uk, kongdy@buct.edu.cn

Content

I. Instrumentation and chemicals S-1
II. Substrates synthesis S-2
III. Optimization studies S-6
IV. General procedure for carboxylation S-11
V. Specific experimental details and product characterization data S-12
VI. Experimental procedures for synthetic applications and associated characterization data S-35
VII. X-ray crystallographic data S-37
VIII. Mechanistic studies and associated characterization data S-42
IX. References S-46
X. NMR spectra S-47

I. Instrumentation and chemicals

^{1}H NMR (400 MHz), ^{13}C NMR (101 MHz) and ^{19}F NMR (376 MHz) spectra were recorded on a Bruker AVANCE III 400 MHz spectrometer at 25 °C unless otherwise noted. For ^{13}C NMR spectra, all spectra were recorded with decoupling to ^{1}H unless otherwise noted. Chemical shifts in ^{1}H NMR spectra are reported in delta (δ) units, parts per million (ppm) relative to residual $CDCl_3$ (δ = 7.26 ppm), CD_3OD (δ = 3.31 ppm), Acetone-*d*$_6$ (δ = 2.05 ppm) or DMSO-*d*$_6$ (δ = 2.50 ppm). Chemical shifts in ^{13}C NMR spectra are reported in delta (δ) units, parts per million (ppm) relative to $CDCl_3$ (δ = 77.16 ppm), CD_3OD (δ = 49.00 ppm), Acetone-*d*$_6$ (δ = 29.84 ppm and 206.26 ppm) or DMSO-*d*$_6$ (δ = 39.52 ppm). The following abbreviations are used for spin multiplicity: s = singlet, d = doublet, t = triplet, q = quartet, m = multiplet, br = broad. High resolution mass spectra (HRMS) were obtained on a Waters Xevo G2 Qtof instrument (ESI) in a positive or a negative ionization mode.

All reactions were conducted under an inert atmosphere of N_2 gas employing standard Schlenk technique or by the use of a N_2-filled glove box unless otherwise noted. The carboxylation reactions were carried out using borosilicate glass vials and heated using an aluminium heating block. All reagents were purchased from commercial sources and used without further purification unless otherwise noted. Anhydrous DMF (*N,N*-dimethylformamide), anhydrous DMA (*N,N*-dimethylacetamide), anhydrous DMSO (dimethyl sulfoxide), anhydrous CPME (cyclopentyl methyl ether), anhydrous Diglyme (diethylene glycol dimethyl ether) and anhydrous 1,4-Dioxane were all purchased from commercial vendors (Energy Chemical), transferred to pre-dried 100 mL glass bottles respectively and stored over 4Å molecular sieves under a nitrogen atmosphere. Dehydrated Toluene was purchased from commercial vendors (Titan) and stored under a nitrogen atmosphere using a solvent purification system. 2,2,2-Triphenylacetic acid (Ph_3C-CO_2H) was purchased from commercial vendors (Leyan). ^{13}C-labeled 2,2,2-triphenylacetic acid (Ph_3C-$^{13}CO_2H$) was synthesized according to the literature procedure from the Ph_3CH and [^{13}C]CO_2 gas.[1] [^{13}C]CO_2 gas (99 atom% ^{13}C) was purchased from Sigma-Aldrich. Carboxylate salts **2a** (**2a-Li**, **2a-Na**, **2a-K**, **2a-Cs** and **2a*-Cs**) were synthesized from Ph_3C-CO_2H and Ph_3C-$^{13}CO_2H$.[1]

II. Substrates synthesis

1) List of substrates

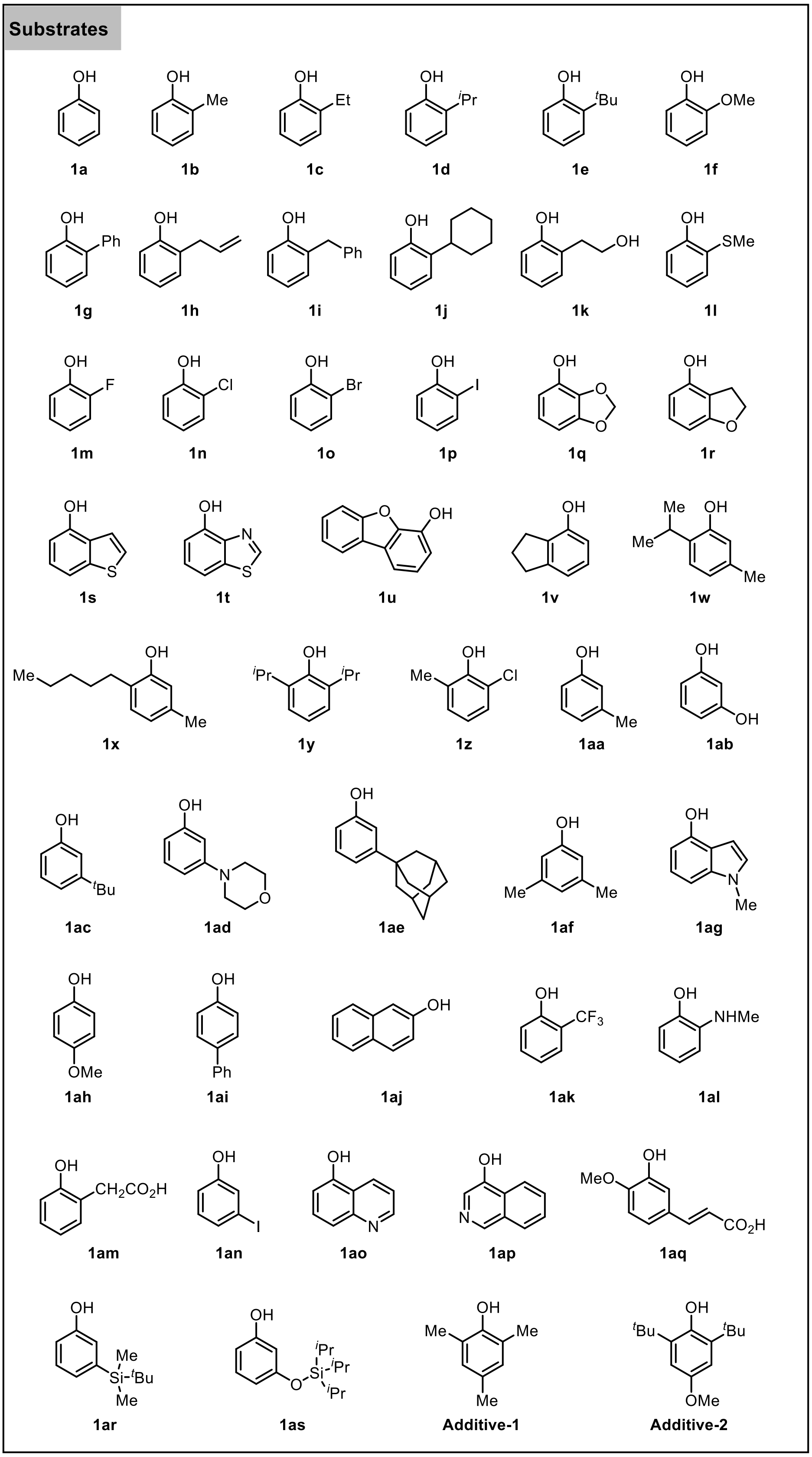

Substrates **1a-1ad**, **1af-1aq** and **Additive 1-Additive 2** were purchased from commercial sources (Leyan, Titan and Bidepharm) and used without further purification.

2) General procedure for the preparation of sterically hindered substrates

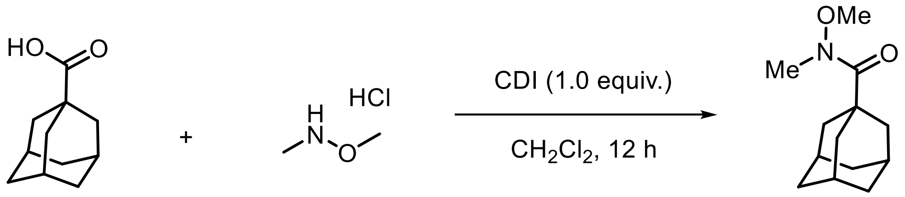

N-Methoxy-N-methyladamantane-1-carboxamide: A 250 mL Schlenk flask was charged with 1-adamantane carboxylic acid (10.0 g, 55.48 mmol, 1.0 equiv.), dichloromethane (150 mL), and a stir bar. To the resulting solution was added 1,1-carbonyldiimidazole (9.0 g, 55.48 mmol, 1.0 equiv.) in separate portions. The reaction mixture was stirred at ambient temperature for 1 h and then *N,O*-dimethylhydroxylamine hydrochloride (6.49 g, 66.58 mmol, 1.2 equiv.) was added. After stirring for an additional 12 h at room temperature, the reaction mixture was transferred to a separatory funnel. The organic fraction was washed with an aqueous solution of saturated $NaHCO_3$, aqueous HCl (1 N), and brine. After drying over anhydrous Na_2SO_4, the solution was concentrated under reduced pressure to afford the desired product as a white solid (10.77 g, 87% yield).

^{1}H NMR (400 MHz, $CDCl_3$): δ 3.62 (s, 3H), 3.10 (s, 3H), 1.94 (s, 9H), 1.66 (s, 6H).

^{13}C NMR (101 MHz, $CDCl_3$): δ 178.4, 60.8, 42.1, 37.9 (3C), 36.8 (3C), 33.9, 28.4 (3C): 13 carbons.

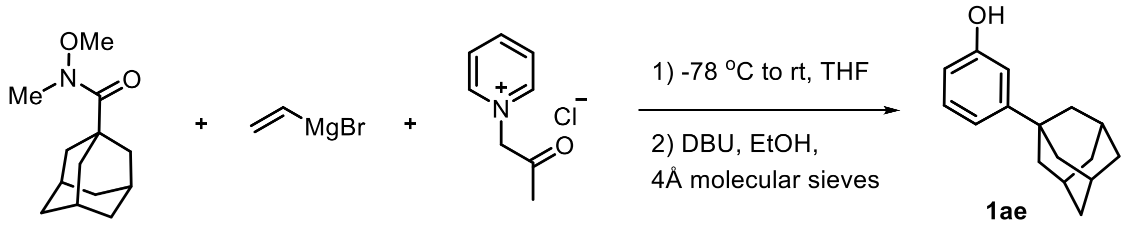

1ae

1-(Adamantan-1-yl)prop-1-en-1-one: A 250 mL flask was charged with *N*-Methoxy-*N*-methyladamantane-1-carboxamide (4.46 g, 10 mmol, 1.0 equiv.), anhydrous THF (150 mL), and a stir bar. After cooling to -78 °C, vinyl magnesium bromide (20 mL, 1 M in THF, 20 mmol) was added dropwise to the reaction mixture. Removal of the cooling bath caused the reaction mixture to warm to room temperature at which time it was stirred for 12 h. The reaction was quenched through the addition of an aqueous solution that was saturated with NH_4Cl and then diluted with ethyl acetate. The aqueous layer was extracted with ethyl acetate (2 × 60 mL). The organic layers were combined, dried over anhydrous Na_2SO_4, and then concentrated under reduced pressure to afford the desired product as a yellow oil. The crude product was used for next step without purification.

3-(adamantan-1-yl) phenol 1ae: The crude product was used in the subsequent reaction without purification. Under argon atmosphere, 2.8 g of 1,8-diazabicyclo[5.4.0]undec-7-ene (DBU) was added into an ice-cooled suspension containing 2.44 g of the crude product of 1-(adamantan-1-yl)prop-1-en-1-one, 3.6 g of 1-(2-oxopropyl)pyridinium chloride, and 2.4 g of 4Å molecular sieves in 50 mL of ethanol. The mixture was stirred at room temperature for 24 h. Under ice cooling, 1 N HCl was added into the reaction mixture to adjust the pH = 1, followed by extraction with ethyl acetate. The organic layer was separated, washed with brine three times, dried over Na_2SO_4, filtered, and concentrated. The residue thus

obtained was subjected to flash chromatography (silica gel, 1:15 v/v mixture of ethyl acetate/hexane elution). The concentration of the relevant fractions then gave product **1ae** (1.39 g, 61% yield) (Rf = 0.4 in 1:15 v/v ethyl acetate/ hexane) as a white solid.[2]
^{1}H NMR (400 MHz, $CDCl_3$): δ 7.19 (t, *J* = 8.0 Hz, 1H), 6.95 (d, *J* = 8.0 Hz, 1H), 6.84 (t, *J* = 2.4 Hz, 1H), 6.65 (dd, *J* = 8.0, 2.4 Hz, 1H), 4.68 (br, s, 1H), 2.11 – 2.07 (m, 3H), 1.90 – 1.89 (m, 6H), 1.82 – 1.72 (m, 6H).
^{13}C NMR (101 MHz, $CDCl_3$): δ 155.5, 153.8, 129.3, 117.5, 112.5, 112.3, 43.3 (3C), 36.9 (3C), 36.3, 29.1 (3C).: 16 carbons.

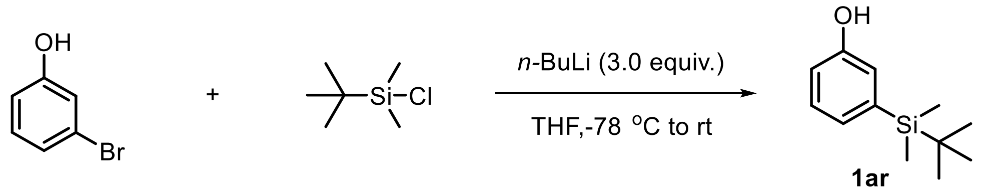

1ar

A magnetically stirred solution of m-bromophenol (5.19 g, 30 mmol, 1.0 equiv.) in dry tetrahydrofuran (150 mL) was treated with n-butyllithium (68 mL of a 1.33 M solution in tetrahydrofuran, 90 mmol, 3 equiv.) at −78 °C. The resulting solution was stirred for 2 h at −78 °C. Then *tert*-butyldimethylchlorosilane (17.3 mmol, 3.0 equiv.) was added into the reaction. Next, the reaction mixture was allowed to warm to room temperature (20 °C) and stirred at this temperature for 8 h before ammonium chloride (1 × 60 mL of a saturated aqueous solution) was added. The separated aqueous phase was extracted with ethyl acetate (3 × 60 mL), and the combined organic phases were washed with brine (2 × 50 mL) before being dried (Na_2SO_4), filtered, and concentrated under reduced pressure. The residue thus obtained was subjected to flash chromatography (silica gel, 1:10 v/v mixture of ethyl acetate/hexane elution), and concentration of the relevant fractions then gave product **1ar** (1.12 g, 18% yield) (Rf = 0.2 in 1:10 v/v ethyl acetate/ hexane) as a colorless oil.[3]
^{1}H NMR (400 MHz, $CDCl_3$): δ 7.10 (t, *J* = 7.6 Hz, 1H), 6.95 (d, *J* = 7.2 Hz, 1H), 6.85 (d, *J* = 2.4 Hz, 1H), 6.72 – 6.69 (m, 1H), 4.93 (s, 1H), 0.74 (s, 9H), 0.12 (s, 6H).
^{13}C NMR (101 MHz, $CDCl_3$): 154.7, 140.1, 128.9 (d, *J* = 6.4 Hz), 127.1 (d, *J* = 7.5 Hz), 121.1 (d, *J* = 9.3 Hz), 115.8 (d, *J* = 6.8 Hz), 26.6 (d, *J* = 7.6 Hz, 3C), 17.0, -6.0 (d, *J* = 4.5 Hz, 2C): 12 carbons.

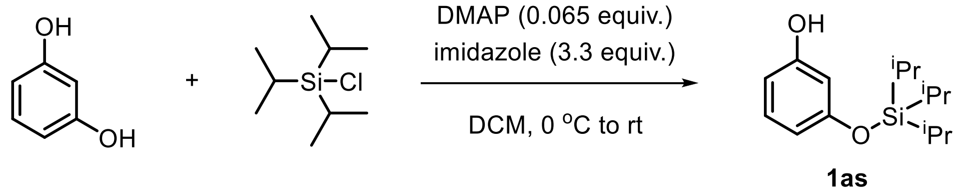

1as

To a solution of Resorcinol (824 mg, 7.5 mmol, 1.5 equiv.) in dichloromethane (20 mL) at 0 °C were successively added triisopropylsilyl chloride (1.1 mL, 5.0 mmol, 1.0 equiv.), imidazole (1.1 g, 16.5 mmol, 3.3 equiv.) and *N*,*N*-dimethyl-4-aminopyridine (40 mg, 0.34 mmol, 0.065 equiv.). After stirring at room temperature for 12 h, the reaction mixture was poured over a saturated aqueous solution of NH_4Cl and diluted with Et_2O. The separated aqueous layer was extracted with Et_2O then dried over Na_2SO_4, filtered and concentrated. Purification by flash-chromatography over silica gel (PE/EtOAc gradient: 95/5 to 60/40)

afforded product **1as** (1.11 g, 83% yield, colorless oil).[4]

^{1}H NMR (400 MHz, $CDCl_3$): δ 7.06 (t, *J* = 8.4 Hz, 1H), 6.49 – 6.39 (m, 3H), 4.97 (s, 1H), 1.31 – 1.20 (m, 3H), 1.10 (d, *J* = 7.2 Hz, 18H).

^{13}C NMR (101 MHz, $CDCl_3$): 157. 5, 156.7, 130.0 (d, *J* = 8.2 Hz), 112.7, 108.3, 107.5, 18.0 (3C), 12.8 (6C): 15 carbons.

III. Optimization studies

General procedure for optimization studies

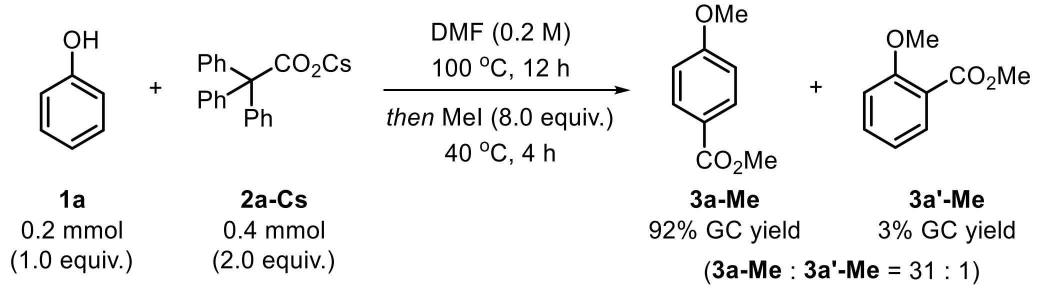

In a glovebox filled with N_2, a 2 mL thick-walled pressure-resistant tube equipped with a stirring bar was charged with phenol **1a** (0.2 mmol, 1.0 equiv., 18.8 mg), cesium 2,2,2-triphenylacetate **2a-Cs** (0.4 mmol, 2.0 equiv., 168.1 mg) and DMF (1.0 mL, 0.2 M) sequentially. The tube was sealed with a PTFE threaded sealing cap and removed from the glovebox. The reaction mixture was transferred to a preheated stir plate and stirred at 100 °C in an aluminum block for 12 hours. After that time, the reaction was cooled down to room temperature (25 °C). Then methyl iodide (MeI) (1.6 mmol, 8.0 equiv., 100 μL) was added into the reaction and the mixture was stirred at 40 °C for 4 hours. The crude reaction mixture was analyzed by GC using 1,2,4,5-tetramethylbenzene as an internal standard.

Table S1. Screening of Counter Cation

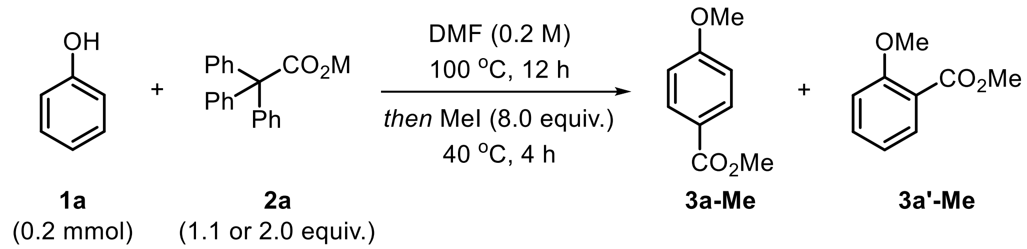

Entry	Ph_3CCO_2M **2a** (x equiv.)	**3a-Me** (%)[a]	**3a'-Me** (%)[a]	(**3a-Me** : **3a'-Me**)[b]
1	Li (2.0 equiv.)	7	10	1 : 1.4
2	Na (2.0 equiv.)[c]	2	38	1 : 19
3	K (1.1 equiv.)	34	2	17 : 1
4	K (2.0 equiv.)	83	7	12 : 1
**5**	**Cs (2.0 equiv.)**	**92**	**3**	**31 : 1**

Reaction conditions: **1a** (0.2 mmol, 1.0 equiv.), **2a** (x equiv.), DMF (1 mL, 0.2 M), 100 °C, 12 h. Then MeI (1.6 mmol, 8.0 equiv., 100 μL), 40 °C, 4 h. [a] Yield was determined by GC using 1,2,4,5-tetramethylbenzene as the internal standard. [b] Selectivity (**3a-Me** : **3a'-Me**) was determined by GC using 1,2,4,5-tetramethylbenzene as the internal standard. [c] The reaction was carried out at 140 °C for 36 h.

Table S2. Evaluation of Substrate Ration

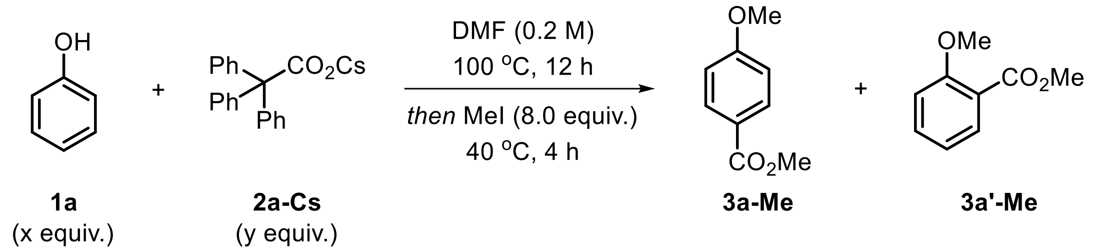

Entry	x : y	**3a-Me** (%)[a]	**3a'-Me** (%)[a]	(**3a-Me** : **3a'-Me**)[b]
1	2 :1	2	n.d.	-
2	1 : 1	26	1	26 : 1
3	1 : 1.5	57	2	28 : 1
**4**	**1 : 2**	**92**	**3**	**31 : 1**
5	1 : 2.5	91	3	30 : 1

Reaction conditions: **1a** (0.2 mmol, x equiv.), **2a-Cs** (y equiv.), DMF (1 mL, 0.2 M), 100 °C, 12 h. Then MeI (1.6 mmol, 8.0 equiv., 100 μL), 40 °C, 4 h. [a] Yield was determined by GC using 1,2,4,5-tetramethylbenzene as the internal standard. [b] Selectivity (**3a-Me** : **3a'-Me**) was determined by GC using 1,2,4,5-tetramethylbenzene as the internal standard. n.d. = not detected.

Table S3. Evaluation of Temperature

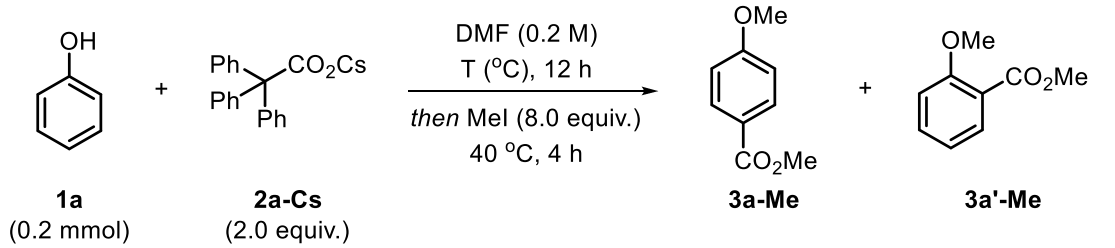

Entry	Temperature (°C)	**3a-Me** (%)[a]	**3a'-Me** (%)[a]	(**3a-Me** : **3a'-Me**)[b]
1	40	18	n.d.	-
2	60	84	2	42 : 1
**3**	**100**	**92**	**3**	**31 : 1**
4	120	79	3	26 : 1

Reaction conditions: **1a** (0.2 mmol, 1.0 equiv.), **2a-Cs** (0.4 mmol, 2.0 equiv.), DMF (1 mL, 0.2 M), T (°C), 12 h. Then MeI (1.6 mmol, 8.0 equiv., 100 μL), 40 °C, 4 h. [a] Yield was determined by GC using 1,2,4,5-tetramethylbenzene as the internal standard. [b] Selectivity (**3a-Me** : **3a'-Me**) was determined by GC using 1,2,4,5-tetramethylbenzene as the internal standard. n.d. = not detected.

Table S4. Evaluation of Solvent

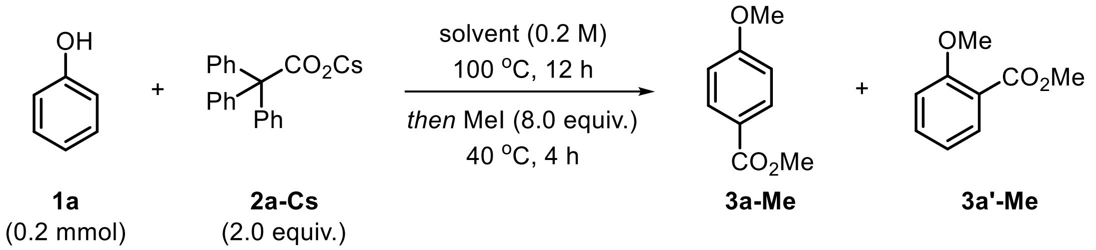

Entry	Solvent	**3a-Me** (%)[a]	**3a'-Me** (%)[a]	(**3a-Me** : **3a'-Me**)[b]
**1**	**DMF**	**92**	**3**	**31 : 1**
2	DMA	25	1	25 : 1
3	DMSO	32	3	11 : 1
4	Toluene	n.d.	n.d.	-
5	CPME	n.d.	n.d.	-
6	Diglyme	57	28	2 : 1
7	Dioxane	n.d.	n.d.	-

Reaction conditions: **1a** (0.2 mmol, 1.0 equiv.), **2a-Cs** (0.4 mmol, 2.0 equiv.), solvent (1 mL, 0.2 M), 100 °C, 12 h. Then MeI (1.6 mmol, 8.0 equiv., 100 µL), 40 °C, 4 h. [a] Yield was determined by GC using 1,2,4,5-tetramethylbenzene as the internal standard. [b] Selectivity (**3a-Me** : **3a'-Me**) was determined by GC using 1,2,4,5-tetramethylbenzene as the internal standard. n.d. = not detected.

Table S5. Evaluation of Concentration

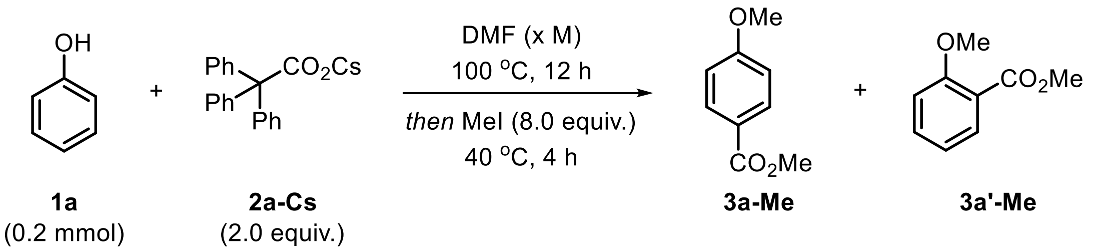

Entry	DMF (x M)	**3a-Me** (%)[a]	**3a'-Me** (%)[a]	(**3a-Me** : **3a'-Me**)[b]
1	0.05	63	1	63 : 1
2	0.10	84	2	42 : 1
**3**	**0.20**	**92**	**3**	**31 : 1**
4	0.30	90	4	22 : 1

Reaction conditions: **1a** (0.2 mmol, 1.0 equiv.), **2a-Cs** (0.4 mmol, 2.0 equiv.), DMF (x M), 100 °C, 12 h. Then MeI (1.6 mmol, 8.0 equiv., 100 µL), 40 °C, 4 h. [a] Yield was determined by GC using 1,2,4,5-tetramethylbenzene as the internal standard. [b] Selectivity (**3a-Me** : **3a'-Me**) was determined by GC using 1,2,4,5-tetramethylbenzene as the internal standard.

Table S6. Evaluation of Carboxylating Agents

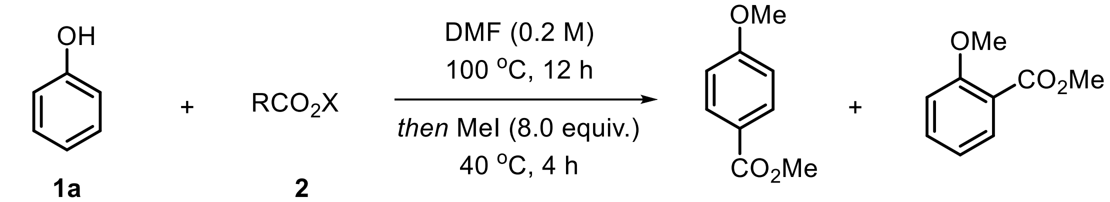

1a (1 equiv.) + **2** (2 equiv.) → **3a-Me** + **3a'-Me**

Entry	**2**	**3a-Me** (%)[a]	**3a'-Me** (%)[a]	(**3a-Me** : **3a'-Me**)[b]
1	**2a-K**	83	7	12:1
2	**2a-Cs**	92	3	31:1
3	**2b-K**	28	2	14:1
4	**2b-Cs**	34	2	17:1
5	**2c-K**	n.d.	n.d.	-
6	**2c-Cs**	n.d.	n.d.	-
7	**2d-K**	n.d.	n.d.	-
8	**2d-Cs**	n.d.	n.d.	-
9	**2e-K**	n.d.	n.d.	-
10	**2e-Cs**	n.d.	n.d.	-
11	**2f-K**	3	n.d.	-
12	**2f-Cs**	5	n.d.	-

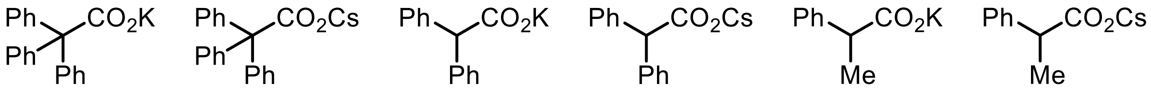

2a-K **2a-Cs** **2b-K** **2b-Cs** **2c-K** **2c-Cs**

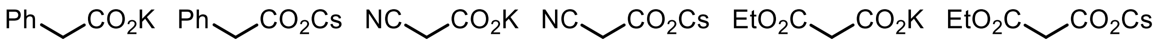

2d-K **2d-Cs** **2e-K** **2e-Cs** **2f-K** **2f-Cs**

Reaction conditions: **1a** (0.2 mmol, 1 equiv.), **2** (2 equiv.), DMF (1 mL, 0.2 M), 100 °C, 12 h. Then MeI (1.6 mmol, 8.0 equiv., 100 μL), 40 °C, 4 h. [a] Yield was determined by GC using 1,2,4,5-tetramethylbenzene as the internal standard. [b] Selectivity (**3a-Me** : **3a'-Me**) was determined by GC using 1,2,4,5-tetramethylbenzene as the internal standard. n.d. = not detected.

Table S7. Investigation on the Equivalent of Cesium Triphenylacetate under CO_2 Atmosphere

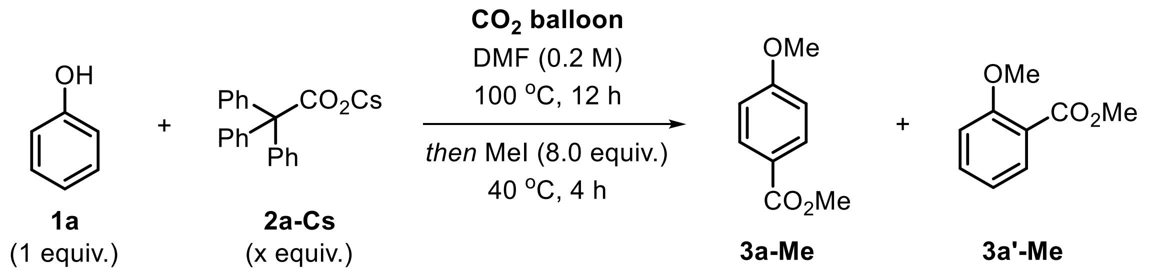

Entry	**2a-Cs**	**3a-Me** (%)[a]	**3a'-Me** (%)[a]	(**3a-Me** : **3a'-Me**)[b]
1	1.0 equiv.	21	n.d.	-
2	0.5 equiv.	n.d.	n.d.	-
3	0.2 equiv.	n.d.	n.d.	-
4	0.1 equiv.	n.d.	n.d.	-

Reaction conditions: **1a** (0.2 mmol, 1.0 equiv.), **2a-Cs** (x equiv.), DMF (1 mL, 0.2 M), 100 °C, 12 h under CO_2 balloon. Then MeI (1.6 mmol, 8.0 equiv., 100 μL), 40 °C, 4 h. [a] Yield was determined by GC using 1,2,4,5-tetramethylbenzene as the internal standard. [b] Selectivity (**3a-Me** : **3a'-Me**) was determined by GC using 1,2,4,5-tetramethylbenzene as the internal standard. n.d. = not detected.

IV. General procedure for carboxylation

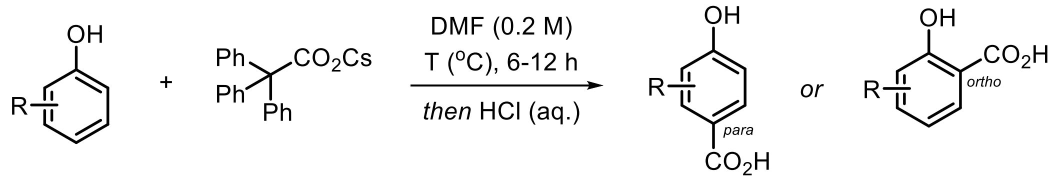

General Procedure: In a glovebox filled with N_2, a 2 mL thick-walled pressure-resistant tube equipped with a stirring bar was charged with phenols **1** (0.2 mmol, 1.0 equiv.), cesium 2,2,2-triphenylacetate **2a-Cs** (0.2-0.6 mmol, 1.0-3.0 equiv.) and DMF (1.0 mL, 0.2 M) sequentially. The tube was sealed with a PTFE threaded sealing cap and removed from the glovebox. The reaction mixture was transferred to a preheated stir plate and stirred at the corresponding temperature in an aluminum block for 6-12 h. After that time, the reaction was cooled down to room temperature (25 °C). The mixture was diluted with H_2O (5 mL) and washed with EtOAc (3 x 6 mL) to remove side-products. Then the aqueous layer was acidified using 1 M HCl (2 mL) solution. The aqueous layer was extracted with EtOAc (3 x 6 mL) and the combined organic layers were washed with 1:1 mixture of H_2O and brine (8 × 2 mL), dried over anhydrous Na_2SO_4, filtered, and then concentrated in vacuo. The crude product was purified by flash column chromatography on silica gel (Hexane/EtOAc with 0.1% AcOH, v/v) to obtain the desired carboxylic acid product **3** or **3'**.

For some substrates, after completion of the above carboxylation reaction, the mixture was cooled down to room temperature (25 °C). Then methyl iodide (MeI) (1.6 mmol, 8.0 equiv., 100 μL) was added into the reaction and the mixture was stirred at 40 °C for 4 h. The reaction was diluted with H_2O (3 mL) and extracted with EtOAc (2 × 6 mL). The combined organic layers were dried over Na_2SO_4, filtered, and then concentrated under reduced pressure. The crude product was purified by flash column chromatography on silica gel using a mixture of hexane and ethyl acetate as an eluting solvent to afford the desired carboxylic acid esters **3-Me**.

Note 1: For substrates **1m**-**1p** and **1ae**, **Additive 1** (0.2 mmol, 1.0 equiv.) or **Additive 2** (0.2 mmol, 1.0 equiv.) was added to the corresponding reaction to improve the yield.

Note 2: The reactions were weighed in the glovebox as a precaution to avoid moisture. However, similar results have been observed when weighing reagents on the bench top. For example, if the reagents were weighed on the bench in air and the reaction was carried out under inert N_2 atmosphere the yield of **3a** was 90% (*para*:*ortho* > 20:1). If the reagents were weighed on the bench in air and the reaction was carried out under air the yield of **3a** was 70% (*para*:*ortho* > 20:1)

V. Specific experimental details and product characterization data

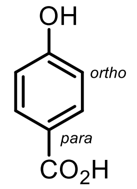

3a

White powder (22.4 mg, 81% yield, *para*:*ortho* > 20:1, m.p. 212–214 °C)
Compound **3a** was obtained from **1a** (0.2 mmol, 1.0 equiv., 18.8 mg) and **2a-Cs** (0.4 mmol, 2.0 equiv., 168.1 mg) in DMF (1 mL, 0.2 M) according to **General Procedure**. The reaction mixture was allowed to stir at 100 °C for 12 hours. After purification through a series of extraction, acidification, back extraction and concentration, the desired product **3a** was obtained without further column chromatography purification.

^{1}H NMR (400 MHz, CD_3OD): δ 7.89 – 7.86 (m, 2H), 6.83 – 6.80 (m, 2H).
^{13}C NMR (101 MHz, CD_3OD): δ 170.1, 163.3, 133.0 (2C), 122.7, 116.0 (2C): 7 carbons.

^{1}H NMR (400 MHz, DMSO-*d_6*): δ 12.41 (br, s, 1H), 10.21 (br, s, 1H), 7.78 (d, J = 8.8 Hz, 2H), 6.82 (d, J = 8.8 Hz, 2H).
^{13}C NMR (101 MHz, DMSO-*d_6*): δ 167.2, 161.6, 131.5 (2C), 121.4, 115.1 (2C): 7 carbons.
^{1}H and ^{13}C NMR data agreed with those previously reported.[5]

HRMS (ESI) m/z: Calcd for $C_7H_5O_3$ $[M-H]^-$: 137.0244; Found 137.0248.

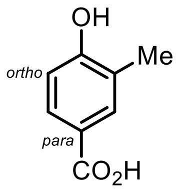

3b

White crystalline solid (26.1 mg, 86% yield, *para*:*ortho* > 20:1, m.p. 167–169 °C)
Compound **3b** was obtained from **1b** (0.2 mmol, 1.0 equiv., 21.6 mg) and **2a-Cs** (0.4 mmol, 2.0 equiv., 168.1 mg) in DMF (1 mL, 0.2 M) according to **General Procedure**. The reaction mixture was allowed to stir at 80 °C for 12 hours. After purification through a series of extractions and flash column chromatography (Hexane/EtOAc (0.1% AcOH, v/v) = 4/1), the desired product **3b** was obtained.

^{1}H NMR (400 MHz, CD_3OD): δ 7.76 (d, J = 1.2 Hz, 1H), 7.71 (dd, J = 8.4, 2.4 Hz, 1H), 6.77 (d, J = 8.0 Hz, 1H), 2.20 (s, 3H).
^{13}C NMR (101 MHz, CD_3OD): δ 170.4, 161.4, 133.7, 130.4, 125.6, 122.4, 115.0, 16.1: 8 carbons.

HRMS (ESI) m/z: Calcd for $C_8H_7O_3$ $[M-H]^-$: 151.0400; Found 151.0403.

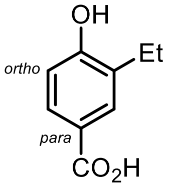

3c

Beige crystalline solid (23.8 mg, 72% yield, *para*:*ortho* > 20:1, m.p. 127–129 °C)
Compound **3c** was obtained from **1c** (0.2 mmol, 1.0 equiv., 24.4 mg) and **2a-Cs** (0.4 mmol, 2.0 equiv., 168.1 mg) in DMF (1 mL, 0.2 M) according to **General Procedure**. The reaction mixture was allowed to stir at 80 °C for 12 hours. After purification through a series of extractions and flash column chromatography (Hexane/EtOAc (0.1% AcOH, v/v) = 4/1), the desired product **3c** was obtained.

^{1}H NMR (400 MHz, CD_3OD): δ 7.78 (d, J = 2.4 Hz, 1H), 7.71 (dd, J = 8.4, 2.0 Hz, 1H), 6.78 (d, J = 8.4 Hz, 1H), 4.96 (br, s, 1H), 2.63 (q, J = 7.6 Hz, 2H), 1.20 (t, J = 7.6 Hz, 3H).
^{13}C NMR (101 MHz, CD_3OD): δ 170.5, 161.1, 132.2, 131.7, 130.4, 122.6, 115.3, 24.1, 14.4: 9 carbons.

HRMS (ESI) m/z: Calcd for $C_9H_9O_3$ $[M-H]^-$: 165.0557; Found 165.0561.

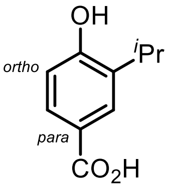

3d

White crystalline solid (23.7 mg, 66% yield, *para*:*ortho* > 20:1, m.p. 132–134 °C)
Compound **3d** was obtained from **1d** (0.2 mmol, 1.0 equiv., 27.2 mg) and **2a-Cs** (0.4 mmol, 2.0 equiv., 168.1 mg) in DMF (1 mL, 0.2 M) according to **General Procedure**. The reaction mixture was allowed to stir at 60 °C for 12 hours. After purification through a series of extraction, acidification, back extraction and concentration, the desired product **3d** was obtained without further column chromatography purification.

^{1}H NMR (400 MHz, CD_3OD): δ 7.85 (d, J = 2.4 Hz, 1H), 7.71 (dd, J = 8.4, 2.4 Hz, 1H), 6.78 (d, J = 8.4 Hz, 1H), 3.30 – 3.23 (m, 1H), 1.23 (d, J = 7.2 Hz, 6H).
^{13}C NMR (101 MHz, CD_3OD): δ 170.5, 160.6, 136.0, 130.1, 129.4, 122.5, 115.5, 28.0, 22.8 (2C): 10 carbons.

HRMS (ESI) m/z: Calcd for $C_{10}H_{11}O_3$ $[M-H]^-$: 179.0713; Found 179.0703.

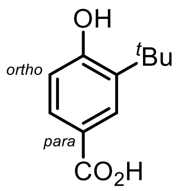

3e

Pale yellow powder (30.0 mg, 77% yield, *para*:*ortho* > 20:1, m.p. 152–154 °C)
Compound **3e** was obtained from **1e** (0.2 mmol, 1.0 equiv., 30.0 mg) and **2a-Cs** (0.4 mmol, 2.0 equiv., 168.1 mg) in DMF (1 mL, 0.2 M) according to **General Procedure**. The reaction mixture was allowed to stir at 60 °C for 12 hours. After purification through a series of extractions and flash column chromatography (Hexane/EtOAc (0.1% AcOH, v/v) = 4/1), the desired product **3e** was obtained.

^{1}H NMR (400 MHz, CD_3OD): δ 7.94 (d, J = 2.4 Hz, 1H), 7.72 (dd, J = 8.4, 2.0 Hz, 1H), 6.77 (d, J = 8.0 Hz, 1H), 1.40 (s, 9H).
^{13}C NMR (101 MHz, CD_3OD): δ 170.7, 162.2, 137.0, 130.4, 130.0, 122.0, 116.7, 35.6, 29.8 (3C): 11 carbons.

HRMS (ESI) m/z: Calcd for $C_{11}H_{13}O_3$ [M-H]$^-$: 193.0870; Found 193.0869.

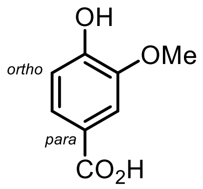

3f

Pale pink powder (27.7 mg, 82% yield, *para*:*ortho* > 20:1, m.p. 209–211 °C)
Compound **3f** was obtained from **1f** (0.2 mmol, 1.0 equiv., 24.8 mg) and **2a-Cs** (0.4 mmol, 2.0 equiv., 168.1 mg) in DMF (1 mL, 0.2 M) according to **General Procedure**. The reaction mixture was allowed to stir at 100 °C for 12 hours. After purification through a series of extraction, acidification, back extraction and concentration, the desired product **3f** was obtained without further column chromatography purification.

^{1}H NMR (400 MHz, CD_3OD): δ 7.57 – 7.54 (m, 2H), 6.84 (d, J = 8.8 Hz, 1H), 3.89 (s, 3H).
^{13}C NMR (101 MHz, CD_3OD): δ 170.0, 152.6, 148.6, 125.3, 123.0, 115.8, 113.8, 56.4: 8 carbons.

HRMS (ESI) m/z: Calcd for $C_8H_7O_4$ [M-H]$^-$: 167.0349; Found 167.0350.

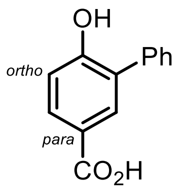

3g

Beige crystalline solid (31.0 mg, 72% yield, *para*:*ortho* > 20:1, m.p. 103–105 °C)
Compound **3g** was obtained from **1g** (0.2 mmol, 1.0 equiv., 34.0 mg) and **2a-Cs** (0.4 mmol, 2.0 equiv., 168.1 mg) in DMF (1 mL, 0.2 M) according to **General Procedure**. The reaction mixture was allowed to stir at 100 °C for 12 hours. After purification through a series of extractions and flash column chromatography (Hexane/EtOAc (0.1% AcOH, v/v) = 3/1), the desired product **3g** was obtained.

^{1}H NMR (400 MHz, CD$_3$OD): δ 7.95 (d, J = 2.4 Hz, 1H), 7.85 (dd, J = 8.4, 2.0 Hz, 1H), 7.56 – 7.54 (m, 2H), 7.42 – 7.37 (m, 2H), 7.33 – 7.28 (m, 1H), 6.94 (d, J = 8.4 Hz, 1H).
^{13}C NMR (101 MHz, CD$_3$OD): δ 170.0, 160.2, 139.3, 133.8, 131.7, 130.3 (2C), 129.9, 129.1 (2C), 128.1, 123.1, 116.7: 13 carbons.

HRMS (ESI) m/z: Calcd for $C_{13}H_9O_3$ [M-H]$^-$: 213.0557; Found 213.0559.

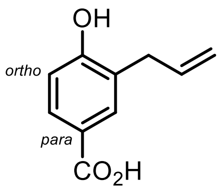

3h

Yellow solid (25.2 mg, 71% yield, *para*:*ortho* = 16:1, m.p. 154–156 °C)
Compound **3h** was obtained from **1h** (0.2 mmol, 1.0 equiv., 26.8 mg) and **2a-Cs** (0.4 mmol, 2.0 equiv., 168.1 mg) in DMF (1 mL, 0.2 M) according to **General Procedure**. The reaction mixture was allowed to stir at 60 °C for 12 hours. After purification through a series of extractions and flash column chromatography (Hexane/EtOAc (0.1% AcOH, v/v) = 5/1), the desired product **3h** was obtained.

^{1}H NMR (400 MHz, CD$_3$OD): δ 7.77 (d, J = 2.0 Hz, 1H), 7.74 (dd, J = 8.4, 2.4 Hz, 1H), 6.80 (d, J = 8.0 Hz, 1H), 6.04 – 5.93 (m, 1H), 5.07 – 5.04 (m, 1H), 5.03 – 5.00 (m, 1H), 3.36 (dt, J = 6.8, 1.6 Hz, 2H).
^{13}C NMR (101 MHz, CD$_3$OD): δ 170.3, 161.0, 137.6, 133.0, 130.8, 127.9, 122.5, 115.9, 115.4, 34.9: 10 carbons.

HRMS (ESI) m/z: Calcd for $C_{10}H_9O_3$ [M-H]$^-$: 177.0557; Found 177.0559.

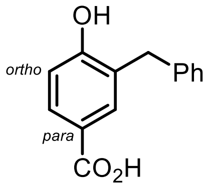

3i

White powder (40.5 mg, 89% yield, *para*:*ortho* > 20:1, m.p. 125–127 °C)
Compound **3i** was obtained from **1i** (0.2 mmol, 1.0 equiv., 36.8 mg) and **2a-Cs** (0.4 mmol, 2.0 equiv., 168.1 mg) in DMF (1 mL, 0.2 M) according to **General Procedure**. The reaction mixture was allowed to stir at 80 °C for 12 hours. After purification through a series of extractions and flash column chromatography (Hexane/EtOAc (0.1% AcOH, v/v) = 4/1), the desired product **3i** was obtained.

^{1}H NMR (400 MHz, CD_3OD): δ 7.75 (dt, *J* = 5.6, 1.2 Hz, 1H), 7.72 (t, *J* = 1.2 Hz, 1H), 7.25 – 7.21 (m, 4H), 7.15 – 7.12 (m, 1H), 6.82 (d, *J* = 5.6 Hz, 1H), 3.95 (s, 2H).
^{13}C NMR (101 MHz, CD_3OD): δ 170.2, 161.1, 142.0, 133.6, 130.8, 129.9 (2C), 129.3 (3C), 126.9, 122.5, 115.5, 36.5: 14 carbons.

HRMS (ESI) m/z: Calcd for $C_{14}H_{11}O_3$ $[M-H]^-$: 227.0713; Found 227.0719.

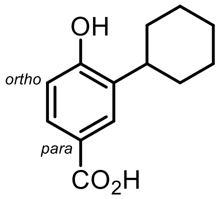

3j

White powder (27.5 mg, 62% yield, *para*:*ortho* > 20:1, m.p. 86–88 °C)
Compound **3j** was obtained from **1j** (0.2 mmol, 1.0 equiv., 35.2 mg) and **2a-Cs** (0.4 mmol, 2.0 equiv., 168.1 mg) in DMF (1 mL, 0.2 M) according to **General Procedure**. The reaction mixture was allowed to stir at 60 °C for 12 hours. After purification through a series of extractions and flash column chromatography (Hexane/EtOAc (0.1% AcOH, v/v) = 4/1), the desired product **3j** was obtained.

^{1}H NMR (400 MHz, CD_3OD): δ 7.83 (d, *J* = 2.0 Hz, 1H), 7.70 (dd, *J* = 8.4, 2.4 Hz, 1H), 6.78 (d, *J* = 8.4 Hz, 1H), 2.96 – 2.89 (m, 1H), 1.85 – 1.82 (m, 4H), 1.76 – 1.71 (m, 1H), 1.49 – 1.35 (m, 4H), 1.33 – 1.22 (m, 1H).
^{13}C NMR (101 MHz, CD_3OD): δ 171.6, 160.1, 135.0, 129.9, 129.8, 123.7, 115.4, 38.2, 34.1 (2C), 28.1 (2C), 27.5: 13 carbons.

HRMS (ESI) m/z: Calcd for $C_{13}H_{15}O_3$ $[M-H]^-$: 219.1026; Found 219.1031.

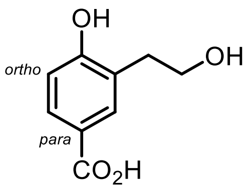

3k

White powder (18.0 mg, 49% yield, *para*:*ortho* = 8:1, m.p. 184–186 °C)
Compound **3k** was obtained from **1k** (0.2 mmol, 1.0 equiv., 27.6 mg) and **2a-Cs** (0.6 mmol, 3.0 equiv., 252.1 mg) in DMF (1 mL, 0.2 M) according to **General Procedure**. The reaction mixture was allowed to stir at 100 °C for 12 hours. After purification through a series of extractions and flash column chromatography (Hexane/EtOAc (0.2% AcOH, v/v) = 4/1), the desired product **3k** was obtained.

^{1}H NMR (400 MHz, CD_3OD): δ 7.81 (d, J = 2.4 Hz, 1H), 7.75 (dd, J = 8.4, 2.0 Hz, 1H), 6.81 (d, J = 8.4 Hz, 1H), 3.76 (t, J = 6.8 Hz, 2H), 2.86 (t, J = 6.8 Hz, 2H).
^{13}C NMR (101 MHz, CD_3OD): δ 170.3, 161.5, 134.1, 131.0, 126.7, 122.6, 115.7, 62.6, 34.9: 9 carbons.

HRMS (ESI) m/z: Calcd for $C_9H_9O_4$ $[M-H]^-$: 181.0506; Found 181.0507.

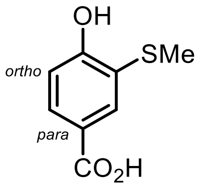

3l

Pale pink crystalline solid (25.8 mg, 70% yield, *para*:*ortho* > 20:1, m.p. 123–125 °C)
Compound **3l** was obtained from **1l** (0.2 mmol, 1.0 equiv., 28.0 mg) and **2a-Cs** (0.4 mmol, 2.0 equiv., 168.1 mg) in DMF (1 mL, 0.2 M) according to **General Procedure**. The reaction mixture was allowed to stir at 80 °C for 12 hours. After purification through a series of extractions and flash column chromatography (Hexane/EtOAc (0.1% AcOH, v/v) = 4/1), the desired product **3l** was obtained.

^{1}H NMR (400 MHz, CD_3OD): δ 7.87 (d, J = 2.0 Hz, 1H), 7.72 (dd, J = 8.4, 2.0 Hz, 1H), 6.81 (d, J = 8.4 Hz, 1H), 2.41 (s, 3H).
^{13}C NMR (101 MHz, CD_3OD): δ 170.5, 160.2, 130.7, 130.0, 126.1, 124.4, 114.9, 15.3: 8 carbons.

HRMS (ESI) m/z: Calcd for $C_8H_7O_3S$ $[M-H]^-$: 183.0121; Found 183.0124.

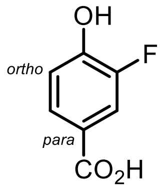

3m

White powder (18.2 mg, 58% yield, *para*:*ortho* > 20:1, m.p. 150–152 °C)
Compound **3m** was obtained from **1m** (0.2 mmol, 1.0 equiv., 22.4 mg), **2a-Cs** (0.4 mmol, 2.0 equiv., 168.1 mg) and **Additive 1** (0.2 mmol, 1.0 equiv., 27.2 mg) in DMF (1 mL, 0.2 M) according to **General Procedure**. The reaction mixture was allowed to stir at 120 °C for 12 hours. After purification through a series of extractions and flash column chromatography (Hexane/EtOAc (0.1% AcOH, v/v) = 5/1), the desired product **3m** was obtained.

^{1}H NMR (400 MHz, CD_3OD): δ 7.70 – 7.65 (m, 2H), 6.98 – 6.93 (m, 1H).
^{13}C NMR (101 MHz, CD_3OD): δ 169.0, 152.1 (d, J = 242.0 Hz), 151.0 (d, J = 13.0 Hz), 128.0 (d, J = 3.0 Hz), 123.3 (d, J = 6.0 Hz), 118.3 (d, J = 19.7 Hz), 118.2 (d, J = 2.4 Hz): 7 carbons.
^{19}F NMR (376 MHz, CD_3OD): δ -139.0 (s, 1F).

HRMS (ESI) m/z: Calcd for $C_7H_4FO_3$ $[M-H]^-$: 155.0149; Found 155.0154.

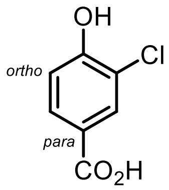

3n

Pale yellow crystalline solid (17.9 mg, 52% yield, *para*:*ortho* > 20:1, m.p. 169–171 °C)
Compound **3n** was obtained from **1n** (0.2 mmol, 1.0 equiv., 25.7 mg), **2a-Cs** (0.4 mmol, 2.0 equiv., 168.1 mg) and **Additive 2** (0.2 mmol, 1.0 equiv., 47.3 mg) in DMF (1 mL, 0.2 M) according to **General Procedure**. The reaction mixture was allowed to stir at 120 °C for 12 hours. After purification through a series of extraction, acidification, back extraction and recrystallization in EtOAc, the desired product **3n** was obtained.

^{1}H NMR (400 MHz, CD_3OD): δ 7.94 (d, J = 2.4 Hz, 1H), 7.79 (dd, J = 8.4, 2.0 Hz, 1H), 6.95 (d, J = 8.4 Hz, 1H).
^{13}C NMR (101 MHz, CD_3OD): δ 168.8, 158.8, 132.9, 131.0, 124.0, 121.6, 117.1: 7 carbons.

^{1}H NMR (400 MHz, DMSO-d_6): δ 12.74 (br, s, 1H), 11.08 (br, s, 1H), 7.84 (d, J = 2.0 Hz, 1H), 7.74 (dd, J = 8.4, 2.0 Hz, 1H), 7.03 (d, J = 8.4 Hz, 1H).
^{13}C NMR (101 MHz, DMSO-d_6): δ 166.2, 157.2, 131.2, 129.9, 122.6, 119.7, 116.3: 7 carbons.
^{1}H and ^{13}C NMR data agreed with those previously reported.[6]

HRMS (ESI) m/z: Calcd for $C_7H_4ClO_3$ $[M-H]^-$: 170.9854; Found 170.9859.

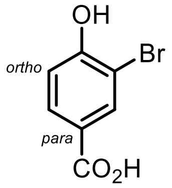

3o

Beige powder (15.2 mg, 35% yield, *para*:*ortho* > 20:1, m.p. 176–178 °C)
Compound **3o** was obtained from **1o** (0.2 mmol, 1.0 equiv., 34.6 mg), **2a-Cs** (0.4 mmol, 2.0 equiv., 168.1 mg) and **Additive 1** (0.2 mmol, 1.0 equiv., 27.2 mg) in DMF (1 mL, 0.2 M) according to **General Procedure**. The reaction mixture was allowed to stir at 120 °C for 12 hours. After purification through a series of extractions and flash column chromatography (Hexane/EtOAc (0.1% AcOH, v/v) = 4/1), the desired product **3o** was obtained.

^{1}H NMR (400 MHz, CD_3OD): δ 8.11 (d, J = 2.4 Hz, 1H), 7.83 (dd, J = 8.4, 2.0 Hz, 1H), 6.93 (d, J = 8.8 Hz, 1H).
^{13}C NMR (101 MHz, CD_3OD): δ 168.6, 159.8, 136.1, 131.7, 124.3, 116.6, 110.5: 7 carbons.

^{1}H NMR (400 MHz, Acetone-d_6): δ 9.76 (br, s, 1H), 8.15 (d, J = 2.4 Hz, 1H), 7.88 (dd, J = 8.8, 2.0 Hz, 1H), 7.10 (d, J = 8.4 Hz, 1H), 6.75 (br, s, 1H).
^{13}C NMR (101 MHz, Acetone-d_6): δ 166.4, 159.0, 135.7, 131.5, 124.3, 116.9, 110.1: 7 carbons.

HRMS (ESI) m/z: Calcd for $C_7H_4BrO_3$ [M-H]$^-$: 214.9349; Found 214.9351.

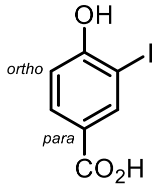

3p

Pale yellow crystalline solid (16.1 mg, 30% yield, *para*:*ortho* > 20:1, m.p. 164–166 °C)
Compound **3p** was obtained from **1p** (0.2 mmol, 1.0 equiv., 44.0 mg), **2a-Cs** (0.4 mmol, 2.0 equiv., 168.1 mg) and **Additive 2** (0.2 mmol, 1.0 equiv., 47.3 mg) in DMF (1 mL, 0.2 M) according to **General Procedure**. The reaction mixture was allowed to stir at 100 °C for 12 hours. After purification through a series of extraction, acidification, back extraction and recrystallization in EtOAc, the desired product **3p** was obtained.

^{1}H NMR (400 MHz, CD_3OD): δ 8.33 (d, J = 2.0 Hz, 1H), 7.85 (dd, J = 8.4, 2.0 Hz, 1H), 6.86 (d, J = 8.4 Hz, 1H).
^{13}C NMR (101 MHz, CD_3OD): δ 169.0, 162.2, 142.4, 132.5, 125.1, 114.9, 83.9: 7 carbons.

HRMS (ESI) m/z: Calcd for $C_7H_4IO_3$ [M-H]$^-$: 262.9210; Found 262.9208.

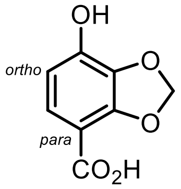

3q

White powder (20.6 mg, 56% yield, *para*:*ortho* > 20:1, m.p. 224–226 °C)
Compound **3q** was obtained from **1q** (0.2 mmol, 1.0 equiv., 27.6 mg) and **2a-Cs** (0.4 mmol, 2.0 equiv., 168.1 mg) in DMF (1 mL, 0.2 M) according to **General Procedure**. The reaction mixture was allowed to stir at 80 °C for 12 hours. After purification through a series of extractions and flash column chromatography (Hexane/EtOAc (0.1% AcOH, v/v) = 4/1), the desired product **3q** was obtained.

^{1}H NMR (400 MHz, CD_3OD): δ 7.29 (d, *J* = 8.8 Hz, 1H), 6.45 (d, *J* = 9.2 Hz, 1H), 6.03 (s, 2H).
^{13}C NMR (101 MHz, CD_3OD): δ 167.8, 151.6, 146.6, 136.0, 125.4, 112.1, 106.7, 103.2: 8 carbons.

HRMS (ESI) m/z: Calcd for $C_8H_5O_5$ $[M-H]^-$: 181.0142; Found 181.0144.

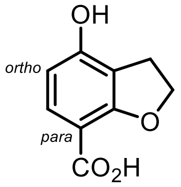

3r

White powder (16.5 mg, 46% yield, *para*:*ortho* = 8:1, m.p. 195–197 °C)
Compound **3r** was obtained from **1r** (0.2 mmol, 1.0 equiv., 27.2 mg) and **2a-Cs** (0.4 mmol, 2.0 equiv., 168.1 mg) in DMF (1 mL, 0.2 M) according to **General Procedure**. The reaction mixture was allowed to stir at 60 °C for 12 hours. After purification through a series of extractions and flash column chromatography (Hexane/EtOAc (0.1% AcOH, v/v) = 4/1), the desired product **3r** was obtained.

^{1}H NMR (400 MHz, CD_3OD): δ 7.57 (d, *J* = 8.4 Hz, 1H), 6.35 (d, *J* = 8.8 Hz, 1H), 4.66 (t, *J* = 8.8 Hz, 2H), 3.10 (t, *J* = 8.8 Hz, 2H).
^{13}C NMR (101 MHz, CD_3OD): δ 168.7, 164.3, 160.1, 132.9, 114.7, 109.4, 106.1, 73.7, 27.1: 9 carbons.

HRMS (ESI) m/z: Calcd for $C_9H_7O_4$ $[M-H]^-$: 179.0349; Found 179.0354.

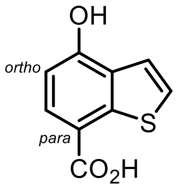

3s

Pale pink crystalline solid (32.1 mg, 83% yield, *para*:*ortho* > 20:1, m.p. 225–226 °C)
Compound **3s** was obtained from **1s** (0.2 mmol, 1.0 equiv., 30.0 mg) and **2a-Cs** (0.4 mmol, 2.0 equiv., 168.1 mg) in DMF (1 mL, 0.2 M) according to **General Procedure**. The reaction mixture was allowed to stir at 60 °C for 12 hours. After purification through a series of extractions and flash column chromatography (Hexane/EtOAc (0.1% AcOH, v/v) = 4/1), the desired product **3s** was obtained.

^{1}H NMR (400 MHz, CD_3OD): δ 7.99 (d, J = 8.4 Hz, 1H), 7.53 – 7.48 (m, 2H), 6.79 (d, J = 8.0 Hz, 1H).
^{13}C NMR (101 MHz, CD_3OD): δ 169.5, 158.6, 144.1, 131.5, 130.8, 128.0, 120.7, 116.9, 109.4: 9 carbons.

HRMS (ESI) m/z: Calcd for $C_9H_5O_3S$ $[M-H]^-$: 192.9964; Found 192.9968.

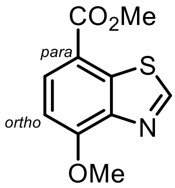

3t-Me

White powder (35.5 mg, 79% yield, *para*:*ortho* > 20:1, m.p. 141–143 °C)
Compound **3t-Me** was obtained from **1t** (0.2 mmol, 1.0 equiv., 30.2 mg) and **2a-Cs** (0.4 mmol, 2.0 equiv., 168.1 mg) in DMF (1 mL, 0.2 M) according to **General Procedure**. The reaction mixture was allowed to stir at 80 °C for 12 hours. Then MeI (1.6 mmol, 8.0 equiv., 100 μL) was added into this solution and the mixture was stirred at 40 °C for 4 h. After purification through a series of extractions and flash column chromatography (Hexane/EtOAc = 2/1), the desired product **3t-Me** was obtained.

^{1}H NMR (400 MHz, $CDCl_3$): δ 8.99 (s, 1H), 8.12 (d, J = 8.4 Hz, 1H), 6.94 (d, J = 8.8 Hz, 1H), 4.10 (s, 3H), 3.96 (s, 3H).
^{13}C NMR (101 MHz, $CDCl_3$): δ 165.9, 157.7, 156.0, 143.9, 136.4, 129.9, 116.6, 106.5, 56.4, 52.4: 10 carbons.

HRMS (ESI) m/z: Calcd for $C_{10}H_{10}NO_3S$ $[M+H]^+$: 224.0376; Found 224.0380.

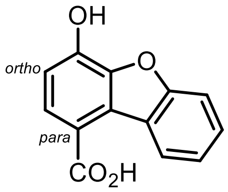

3u

White powder (24.6 mg, 54% yield, *para*:*ortho* > 20:1, m.p. 211–213 °C)
Compound **3u** was obtained from **1u** (0.2 mmol, 1.0 equiv., 36.8 mg) and **2a-Cs** (0.4 mmol, 2.0 equiv., 168.1 mg) in DMF (1 mL, 0.2 M) according to **General Procedure**. The reaction mixture was allowed to stir at 100 °C for 12 hours. After purification through a series of extractions and flash column chromatography (Hexane/EtOAc (0.1% AcOH, v/v) = 4/1), the desired product **3u** was obtained.

^{1}H NMR (400 MHz, CD_3OD): δ 8.89 (d, J = 8.0 Hz, 1H), 7.92 (d, J = 8.4 Hz, 1H), 7.60 (d, J = 8.4 Hz, 1H), 7.51 – 7.47 (m, 1H), 7.34 – 7.30 (m, 1H), 6.97 (d, J = 8.4 Hz, 1H).
^{13}C NMR (101 MHz, CD_3OD): δ 170.0, 158.1, 148.5, 146.1, 129.3, 129.1, 127.7, 127.1, 124.7, 123.6, 117.9, 113.5, 112.2: 13 carbons.

HRMS (ESI) m/z: Calcd for $C_{13}H_7O_4$ [M-H]$^-$: 227.0349; Found 227.0350.

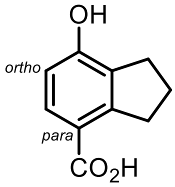

3v

White powder (26.1 mg, 73% yield, *para*:*ortho* = 14:1, m.p. 200–202 °C)
Compound **3v** was obtained from **1v** (0.2 mmol, 1.0 equiv., 26.8 mg) and **2a-Cs** (0.4 mmol, 2.0 equiv., 168.1 mg) in DMF (1 mL, 0.2 M) according to **General Procedure**. The reaction mixture was allowed to stir at 80 °C for 12 hours. After purification through a series of extractions and flash column chromatography (Hexane/EtOAc (0.1% AcOH, v/v) = 4/1), the desired product **3v** was obtained.

^{1}H NMR (400 MHz, CD_3OD): δ 7.70 (d, J = 8.4 Hz, 1H), 6.61 (d, J = 8.4 Hz, 1H), 3.23 (t, J = 7.6 Hz, 2H), 2.82 (t, J = 7.6 Hz, 2H), 2.08 – 2.01 (m, 2H).
^{13}C NMR (101 MHz, CD_3OD): δ 171.0, 158.8, 150.8, 132.1, 131.8, 119.6, 113.6, 35.6, 29.8, 25.6: 10 carbons.

HRMS (ESI) m/z: Calcd for $C_{10}H_9O_3$ [M-H]$^-$: 177.0557; Found 177.0560.

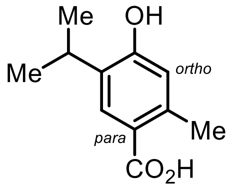

3w

White powder (33.0 mg, 86% yield, *para*:*ortho* > 20:1, m.p. 146–148 °C)
Compound **3w** was obtained from **1w** (0.2 mmol, 1.0 equiv., 30.0 mg) and **2a-Cs** (0.4 mmol, 2.0 equiv., 168.1 mg) in DMF (1 mL, 0.2 M) according to **General Procedure**. The reaction mixture was allowed to stir at 80 °C for 12 hours. After purification through a series of extractions and flash column chromatography (Hexane/EtOAc (0.1% AcOH, v/v) = 4/1), the desired product **3w** was obtained.

^{1}H NMR (400 MHz, CD_3OD): δ 7.81 (s, 1H), 6.61 (s, 1H), 3.27 – 3.17 (m, 1H), 2.48 (s, 3H), 1.20 (d, *J* = 6.8 Hz, 6H).
^{13}C NMR (101 MHz, CD_3OD): δ 171.3, 159.4, 141.3, 133.4, 131.0, 121.3, 118.7, 27.7, 22.9 (2C), 22.1: 11 carbons.

HRMS (ESI) m/z: Calcd for $C_{11}H_{13}O_3$ $[M-H]^-$: 193.0870; Found 193.0872.

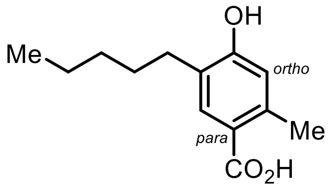

3x

Yellow crystalline solid (15.7 mg, 35% yield, *para*:*ortho* = 5:1, m.p. 125–127 °C)
Compound **3x** was obtained from **1x** (0.2 mmol, 1.0 equiv., 35.6 mg) and **2a-Cs** (0.4 mmol, 2.0 equiv., 168.1 mg) in DMF (1 mL, 0.2 M) according to **General Procedure**. The reaction mixture was allowed to stir at 80 °C for 12 hours. After purification through a series of extractions and flash column chromatography (Hexane/EtOAc (0.1% AcOH, v/v) = 4/1), the desired product **3x** was obtained.

^{1}H NMR (400 MHz, CD_3OD): δ 7.71 (s, 1H), 6.61 (s, 1H), 2.54 (t, *J* = 7.6 Hz, 2H), 2.48 (s, 3H), 1.61 – 1.54 (m, 2H), 1.38 – 1.28 (m, 4H), 0.90 (t, *J* = 6.8 Hz, 3H).
^{13}C NMR (101 MHz, CD_3OD): δ 171.2, 160.1, 141.6, 134.7, 127.7, 121.2, 118.7, 32.8, 30.5 (2C), 23.6, 22.1, 14.4: 13 carbons.

HRMS (ESI) m/z: Calcd for $C_{13}H_{17}O_3$ $[M-H]^-$: 221.1183; Found 221.1185.

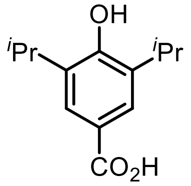

3y

White powder (35.2 mg, 79% yield, m.p. 147–149 °C)
Compound **3y** was obtained from **1y** (0.2 mmol, 1.0 equiv., 35.6 mg) and **2a-Cs** (0.4 mmol, 2.0 equiv., 168.1 mg) in DMF (1 mL, 0.2 M) according to **General Procedure**. The reaction mixture was allowed to stir at 80 °C for 12 hours. After purification through a series of extraction, acidification, back extraction and concentration, the desired product **3y** was obtained without further flash column chromatography purification.

^{1}H NMR (400 MHz, CD_3OD): δ 7.74 (s, 2H), 3.36 – 3.25 (m, 2H), 1.23 (d, J = 6.8 Hz, 12H).
^{13}C NMR (101 MHz, CD_3OD): δ 170.8, 156.9, 136.1 (2C), 126.6 (2C), 122.8, 27.8 (2C), 23.2 (4C): 13 carbons.

^{1}H NMR (400 MHz, DMSO-*d*$_6$): δ 12.31 (br, s, 1H), 8.88 (s, 1H), 7.61 (s, 2H), 3.36 – 3.25 (m, 2H), 1.16 (d, J = 6.8 Hz, 12H).
^{13}C NMR (101 MHz, DMSO-*d*$_6$): δ 167.6, 155.2, 134.7 (2C), 124.9 (2C), 121.8, 26.1 (2C), 22.8 (4C): 13 carbons.
^{1}H and ^{13}C NMR data agreed with those previously reported.[7]

HRMS (ESI) m/z: Calcd for $C_{13}H_{17}O_3$ $[M-H]^-$: 221.1183; Found 221.1185.

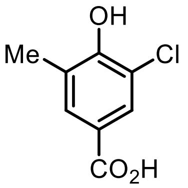

3z

White powder (15.4 mg, 41% yield, m.p. > 300 °C)
Compound **3z** was obtained from **1z** (0.2 mmol, 1.0 equiv., 28.5 mg) and **2a-Cs** (0.3 mmol, 1.5 equiv., 126.1 mg) in DMF (1 mL, 0.2 M) according to **General Procedure**. The reaction mixture was allowed to stir at 100 °C for 12 hours. After purification through a series of extractions and flash column chromatography (Hexane/EtOAc (0.1% AcOH, v/v) = 4/1), the desired product **3z** was obtained.

^{1}H NMR (400 MHz, CD_3OD): δ 7.79 (d, J = 1.6 Hz, 1H), 7.69 (d, J = 1.2 Hz, 1H), 2.26 (s, 3H).
^{13}C NMR (101 MHz, CD_3OD): δ 169.8, 156.2, 131.9, 130.0, 127.7, 124.7, 121.1, 16.8: 8 carbons.

HRMS (ESI) m/z: Calcd for $C_8H_6ClO_3$ $[M-H]^-$: 185.0010; Found 185.0011.

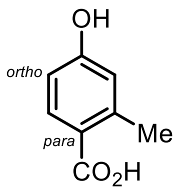

3aa

White powder (16.6 mg, 55% yield, *para*:*ortho* = 3:1, m.p. 175–177 °C)
Compound **3aa** was obtained from **1aa** (0.2 mmol, 1.0 equiv., 21.6 mg) and **2a-Cs** (0.4 mmol, 2.0 equiv., 168.1 mg) in DMF (1 mL, 0.2 M) according to **General Procedure**. The reaction mixture was allowed to stir at 80 °C for 12 hours. After purification through a series of extractions and flash column chromatography (Hexane/EtOAc (0.1% AcOH, v/v) = 5/1), the desired product **3aa** was obtained.

^{1}H NMR (400 MHz, CD$_3$OD): δ 7.85 (d, J = 8.4 Hz, 1H), 6.66 – 6.62 (m, 2H), 2.52 (s, 3H).
^{13}C NMR (101 MHz, CD$_3$OD): δ 170.9, 162.2, 144.4, 134.6, 121.7, 119.1, 113.5, 22.5: 8 carbons.

^{1}H NMR (400 MHz, DMSO-d_6): δ 10.02 (br, s, 1H), 7.75 (d, J = 9.2 Hz, 1H), 6.65 – 6.62 (m, 2H), 2.46 (s, 3H).
^{13}C NMR (101 MHz, DMSO-d_6): δ 168.1, 160.5, 142.3, 133.1, 120.5, 118.1, 112.7, 22.0: 8 carbons.
^{1}H and ^{13}C NMR data agreed with those previously reported.[8]

HRMS (ESI) m/z: Calcd for $C_8H_7O_3$ [M-H]$^-$: 151.0400; Found 151.0391.

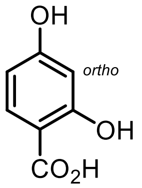

3ab

White powder (22.2 mg, 72% yield, m.p. 207–209 °C)
Compound **3ab** was obtained from **1ab** (0.2 mmol, 1.0 equiv., 22.0 mg) and **2a-Cs** (0.4 mmol, 2.0 equiv., 168.1 mg) in DMF (1 mL, 0.2 M) according to **General Procedure**. The reaction mixture was allowed to stir at 60 °C for 12 hours. After purification through a series of extractions and flash column chromatography (Hexane/EtOAc (0.1% AcOH, v/v) = 4/1), the desired product **3ab** was obtained.

^{1}H NMR (400 MHz, CD$_3$OD): δ 7.69 (d, J = 8.8 Hz, 1H), 6.32 (dd, J = 8.8, 2.4 Hz, 1H), 6.28 (d, J = 2.4 Hz, 1H), 5.21 (br, s, 2H).
^{13}C NMR (101 MHz, CD$_3$OD): δ 173.5, 165.3, 165.2, 133.2, 108.7, 105.9, 103.3: 7 carbons.

HRMS (ESI) m/z: Calcd for $C_7H_5O_4$ [M-H]$^-$: 153.0193; Found 153.0192.

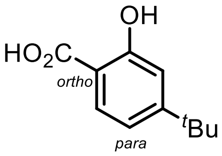

3ac'

Yellow crystalline solid (20.0 mg, 52% yield, *ortho*:*para* > 20:1, m.p. 136–138 °C)

Compound **3ac′** was obtained from **1ac** (0.2 mmol, 1.0 equiv., 30.0 mg) and **2a-Cs** (0.4 mmol, 2.0 equiv., 168.1 mg) in DMF (1 mL, 0.2 M) according to **General Procedure**. The reaction mixture was allowed to stir at 100 °C for 12 hours. After purification through a series of extractions and flash column chromatography (Hexane/EtOAc (0.1% AcOH, v/v) = 4/1), the desired product **3ac′** was obtained.

^{1}H NMR (400 MHz, CD_3OD): δ 7.76 (d, J = 8.0 Hz, 1H), 6.95 (dd, J = 8.4, 1.6 Hz, 1H), 6.93 (d, J = 1.6 Hz, 1H), 1.30 (s, 9H).
^{13}C NMR (101 MHz, CD_3OD): δ 173.4, 163.0, 161.0, 131.2, 117.6, 114.8, 111.3, 35.9, 31.3 (3C): 11 carbons.

HRMS (ESI) m/z: Calcd for $C_{11}H_{13}O_3$ $[M-H]^-$: 193.0870; Found 193.0873.

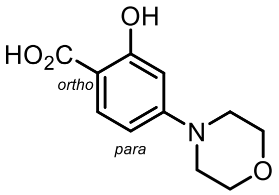

3ad'

White crystalline solid (28.0 mg, 63% yield, *ortho*:*para* > 20:1, m.p. 195–197 °C)

Compound **3ad′** was obtained from **1ad** (0.2 mmol, 1.0 equiv., 35.8 mg) and **2a-Cs** (0.4 mmol, 2.0 equiv., 168.1 mg) in DMF (1 mL, 0.2 M) according to **General Procedure**. The reaction mixture was allowed to stir at 80 °C for 12 hours. After purification through a series of extraction, acidification, back extraction and recrystallization in methanol, the desired product **3ad′** was obtained.

^{1}H NMR (400 MHz, DMSO-d_6): δ 7.58 (d, J = 9.2 Hz, 1H), 6.50 (dd, J = 9.2, 2.4 Hz, 1H), 6.33 (d, J = 2.4 Hz, 1H), 3.71 – 3.68 (m, 4H), 3.26 – 3.23 (m, 4H).
^{13}C NMR (101 MHz, DMSO-d_6): δ 171.8, 162.9, 156.1, 131.1, 105.8, 102.5, 99.7, 65.8 (2C), 46.6 (2C): 11 carbons.

HRMS (ESI) m/z: Calcd for $C_{11}H_{12}NO_4$ $[M-H]^-$: 222.0771; Found 222.0775.

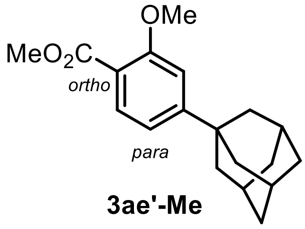

3ae'-Me

Pale yellow solid (19.2 mg, 32% yield, *ortho*:*para* > 20:1, m.p. 106–108 °C)
Compound **3ae′-Me** was obtained from **1ae** (0.2 mmol, 1.0 equiv., 45.7 mg) and **2a-Cs** (0.4 mmol, 2.0 equiv., 168.1 mg) in DMF (1 mL, 0.2 M) according to **General Procedure**. The reaction mixture was allowed to stir at 100 °C for 12 hours. Then MeI (1.6 mmol, 8.0 equiv., 100 μL) was added into this solution and the mixture was stirred at 40 °C for 4 h. After purification through a series of extractions and flash column chromatography (Hexane/EtOAc = 15/1), the desired product **3ae′-Me** was obtained.

^{1}H NMR (400 MHz, $CDCl_3$): *δ* 7.76 (d, *J* = 8.4 Hz, 1H), 6.98 (dd, *J* = 8.4, 1.6 Hz, 1H), 6.96 (d, *J* = 1.6 Hz, 1H), 3.92 (s, 3H), 3.87 (s, 3H), 2.12 – 2.09 (m, 3H), 1.91 (d, *J* = 2.8 Hz, 6H), 1.82 – 1.73 (m, 6H).
^{13}C NMR (101 MHz, $CDCl_3$): *δ* 166.7, 159.4, 158.0, 131.6, 117.2, 117.1, 109.1, 56.1, 51.9, 43.0 (3C), 37.0, 36.8 (3C), 28.9 (3C): 19 carbons.

HRMS (ESI) m/z: Calcd for $C_{19}H_{25}O_3$ $[M+H]^+$: 301.1798; Found 301.1806.

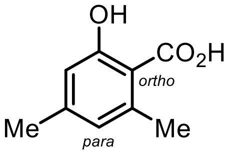

3af'

White powder (20.1 mg, 61% yield, *ortho*:*para* > 20:1, m.p. > 300 °C)
Compound **3af′** was obtained from **1af** (0.2 mmol, 1.0 equiv., 24.4 mg), **2a-Cs** (0.4 mmol, 2.0 equiv., 168.1 mg) and **Additive 2** (0.2 mmol, 1.0 equiv., 47.3 mg) in DMF (1 mL, 0.2 M) according to **General Procedure**. The reaction mixture was allowed to stir at 120 °C for 12 hours. After purification through a series of extractions and flash column chromatography (Hexane/EtOAc (0.1% AcOH, v/v) = 4/1), the desired product **3af′** was obtained.

^{1}H NMR (400 MHz, CD_3OD): *δ* 6.53 (s, 1H), 6.50 (s, 1H), 2.51 (s, 3H), 2.22 (s, 3H).
^{13}C NMR (101 MHz, CD_3OD): *δ* 174.9, 164.1, 146.0, 142.6, 124.8, 116.3, 111.3, 23.8, 21.5: 9 carbons.

HRMS (ESI) m/z: Calcd for $C_9H_9O_3$ $[M-H]^-$: 165.0557; Found 165.0560.

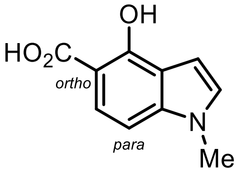

3ag'

Dark green crystalline solid (27.3 mg, 70% yield, *ortho*:*para* > 20:1, m.p. 169–170 °C)

Compound **3ag'** was obtained from **1ag** (0.2 mmol, 1.0 equiv., 29.4 mg) and **2a-Cs** (0.2 mmol, 1.0 equiv., 84.1 mg) in DMF (1 mL, 0.2 M) according to **General Procedure**. The reaction mixture was allowed to stir at 100 °C for 6 hours. After purification through a series of extractions and flash column chromatography (Hexane/EtOAc (0.1% AcOH, v/v) = 3/1), the desired product **3ag'** was obtained.

^{1}H NMR (400 MHz, CD_3OD): δ 7.63 (d, J = 8.8 Hz, 1H), 7.04 (d, J = 2.8 Hz, 1H), 6.85 (d, J = 8.8 Hz, 1H), 6.60 (d, J = 2.8 Hz, 1H), 3.74 (s, 3H).

^{13}C NMR (101 MHz, CD_3OD): δ 175.4, 159.0, 142.6, 129.2, 124.4, 118.9, 103.7, 102.4, 100.8, 33.0: 10 carbons.

HRMS (ESI) m/z: Calcd for $C_{10}H_8NO_3$ [M-H]$^-$: 190.0509; Found 190.0513.

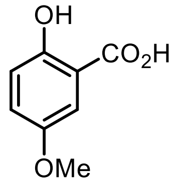

3ah'

White powder (14.1 mg, 42% yield, m.p. 130–132 °C)

Compound **3ah'** was obtained from **1ah** (0.2 mmol, 1.0 equiv., 24.8 mg) and **2a-Cs** (0.2 mmol, 1.0 equiv., 84.1 mg) in DMF (1 mL, 0.2 M) according to **General Procedure**. The reaction mixture was allowed to stir at 100 °C for 12 hours. After purification through a series of extractions and flash column chromatography (Hexane/EtOAc (0.1% AcOH, v/v) = 5/1), the desired product **3ah'** was obtained.

^{1}H NMR (400 MHz, CD_3OD): δ 7.36 (d, J = 3.2 Hz, 1H), 7.03 (dd, J = 9.2, 2.8 Hz, 1H), 6.81 (d, J = 8.8 Hz, 1H), 3.75 (s, 3H).

^{13}C NMR (101 MHz, CD_3OD): δ 173.2, 157.4, 153.4, 124.5, 119.0, 113.7, 113.5, 56.2: 8 carbons.

HRMS (ESI) m/z: Calcd for $C_8H_7O_4$ [M-H]$^-$: 167.0349; Found 167.0356.

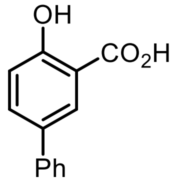

3ai'

Pale yellow powder (8.5 mg, 20% yield, m.p. 207–209 °C)
Compound **3ai′** was obtained from **1ai** (0.2 mmol, 1.0 equiv., 34.0 mg) and **2a-Cs** (0.2 mmol, 1.0 equiv., 84.1 mg) in DMF (1 mL, 0.2 M) according to **General Procedure**. The reaction mixture was allowed to stir at 100 °C for 12 hours. After purification through a series of extractions and flash column chromatography (Hexane/EtOAc (0.1% AcOH, v/v) = 5/1), the desired product **3ai′** was obtained.

^{1}H NMR (400 MHz, CD_3OD): δ 8.09 (d, J = 2.4 Hz, 1H), 7.72 (dd, J = 8.8, 2.8 Hz, 1H), 7.56 – 7.53 (m, 2H), 7.42 – 7.38 (m, 2H), 7.31 – 7.27 (m, 1H), 7.00 (d, J = 8.8 Hz, 1H).
^{13}C NMR (101 MHz, CD_3OD): δ 173.6, 162.6, 141.3, 134.9, 133.5, 129.9 (2C), 129.5, 128.0, 127.4 (2C), 118.7, 114.5: 13 carbons.

HRMS (ESI) m/z: Calcd for $C_{13}H_9O_3$ [M-H]$^-$: 213.0557; Found 213.0561.

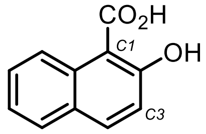

3aj'

Pale yellow crystalline solid (20.1 mg, 53% yield, *C1*:*C3* > 20:1, m.p. 141–143 °C)
Compound **3aj′** was obtained from **1aj** (0.2 mmol, 1.0 equiv., 28.8 mg) and **2a-Cs** (0.4 mmol, 2.0 equiv., 168.1 mg) in DMF (1 mL, 0.2 M) according to **General Procedure**. The reaction mixture was allowed to stir at 100 °C for 12 hours. After purification through a series of extraction, acidification, back extraction and recrystallization in methanol, the desired product **3aj′** was obtained.

^{1}H NMR (400 MHz, CD_3OD): δ 8.87 (d, J = 8.8 Hz, 1H), 7.92 (d, J = 8.8 Hz, 1H), 7.76 (d, J = 8.0 Hz, 1H), 7.54 – 7.49 (m, 1H), 7.35 – 7.31 (m, 1H), 7.12 (d, J = 9.2 Hz, 1H).
^{13}C NMR (101 MHz, CD_3OD): δ 175.4, 165.3, 137.4, 133.6, 130.0, 129.9, 129.2, 126.5, 124.5, 119.9, 106.2: 11 carbons.

HRMS (ESI) m/z: Calcd for $C_{11}H_7O_3$ [M-H]$^-$: 187.0400; Found 187.0403.

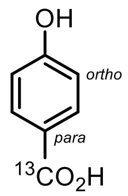

3a*

White powder (7.6 mg, 55% yield, *para*:*ortho* > 20:1, 99% ^{13}C incorporation, m.p. 200–202 °C)

Compound **3a*** was obtained from **1a** (0.1 mmol, 1.0 equiv., 9.4 mg) and **2a*-Cs** (0.2 mmol, 2.0 equiv., 84.2 mg) in DMF (0.5 mL, 0.2 M) according to **General Procedure**. The reaction mixture was allowed to stir at 100 °C for 12 hours. After purification through a series of extractions and flash column chromatography (Hexane/EtOAc (0.1% AcOH, v/v) = 4/1), the desired product **3a*** was obtained. The ^{13}C% incorporation was obtained through analysis of high-resolution mass spectrum of the compound **3a***.

^1H NMR (400 MHz, CD_3OD): δ 7.90 – 7.86 (m, 2H), 6.84 – 6.80 (m, 2H).

^{13}C NMR (101 MHz, CD_3OD): δ 170.1, 163.3, 133.0 (d, J = 3.0 Hz, 2C), 122.7 (d, J = 74.7 Hz), 116.0 (d, J = 4.7 Hz, 2C): 7 carbons.

HRMS (ESI) m/z: Calcd for $C_6[^{13}C]H_5O_3$ $[M-H]^-$: 138.0277; Found 138.0280.

Information on Determination of ^{13}C Incorporation for compound 3a*

Percent ^{13}C isotope incorporation was determined by comparison of the mass spectral patterns of carbon-13 labeled product versus authentic starting material and calculated by the expressions below.

% ^{13}C incorp. = [(Corrected abundance of ^{13}C) / (Corrected abundance of ^{13}C + Observed abundance of ^{12}C)] x 100, where observed abundance of ^{12}C is obtained from the mass signal intensities at M (m/z);

Corrected abundance of ^{13}C = Observed abundance of ^{13}C - (Observed abundance of ^{12}C x Relative Natural Abundance of ^{13}C / 100), where observed abundance of ^{13}C is obtained from the mass signal intensities at M+1 (m/z).

Corrected abundance of ^{13}C of compound 3a* = 3470000 – 36300 x 7.6 / 100 = 3467241.2;

% ^{13}C incorp. of compound 3a* = 3467241.2 / (3467241.2 + 36300) x 100 = 99%.

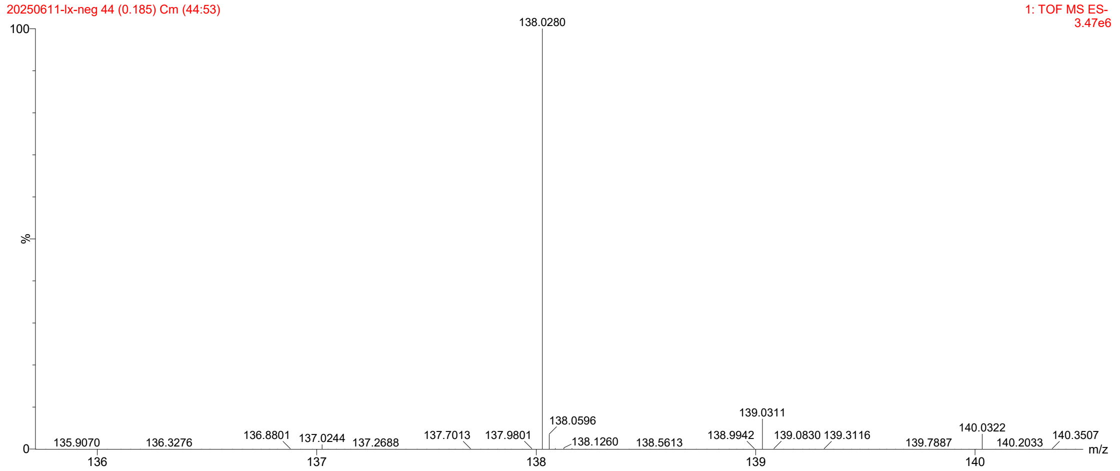

m/z	Relative Natural Abundance (%)	Observed Abundance (%)	Corrected Abundance (%)	Isotopic Enrichment Relative (%)
[M-H]+0	100	36300	36300	1
[M-H]+1	7.6	3470000	3467241.2	99

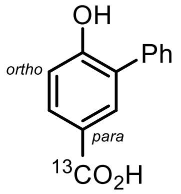

3g*

White powder (12.2 mg, 57% yield, *para*:*ortho* > 20:1, 99% ^{13}C incorporation, m.p. 141–143 °C)

Compound **3g*** was obtained from **1g** (0.1 mmol, 1.0 equiv., 17.0 mg) and **2a*-Cs** (0.2 mmol, 2.0 equiv., 84.2 mg) in DMF (0.5 mL, 0.2 M) according to **General Procedure**. The reaction mixture was allowed to stir at 100 °C for 12 hours. After purification through a series of extractions and flash column chromatography (Hexane/EtOAc (0.1% AcOH, v/v) = 4/1), the desired product **3g*** was obtained. The ^{13}C% incorporation was obtained through analysis of high-resolution mass spectrum of the compound **3g***.

^{1}H NMR (400 MHz, CD_3OD): δ 7.95 (dd, J = 4.0, 2.4 Hz, 1H), 7.86 (ddd, J = 8.4, 4.0, 2.0 Hz, 1H), 7.56 – 7.54 (m, 2H), 7.42 – 7.37 (m, 2H), 7.32 – 7.28 (m, 1H), 6.95 (d, J = 8.4 Hz, 1H).
^{13}C NMR (101 MHz, CD_3OD): δ 170.0, 160.1, 139.3, 133.8 (d, J = 3.3 Hz), 131.7 (d, J = 2.8 Hz), 130.3 (2C), 129.8 (d, J = 4.5 Hz), 129.1 (2C), 128.1, 123.1 (d, J = 74.5 Hz), 116.6 (d, J = 4.8 Hz): 13 carbons.

HRMS (ESI) m/z: Calcd for $C_{12}[^{13}C]H_9O_3$ $[M-H]^-$: 214.0590; Found 214.0586.

Information on Determination of ^{13}C Incorporation for compound 3g*
Corrected abundance of ^{13}C of compound 3g* = 7040000 – 77900 x 14.1 / 100 = 7029016.1;
% ^{13}C incorp. of compound 3g* = 7029016.1 / (7029016.1 + 77900) x 100 = 99%.

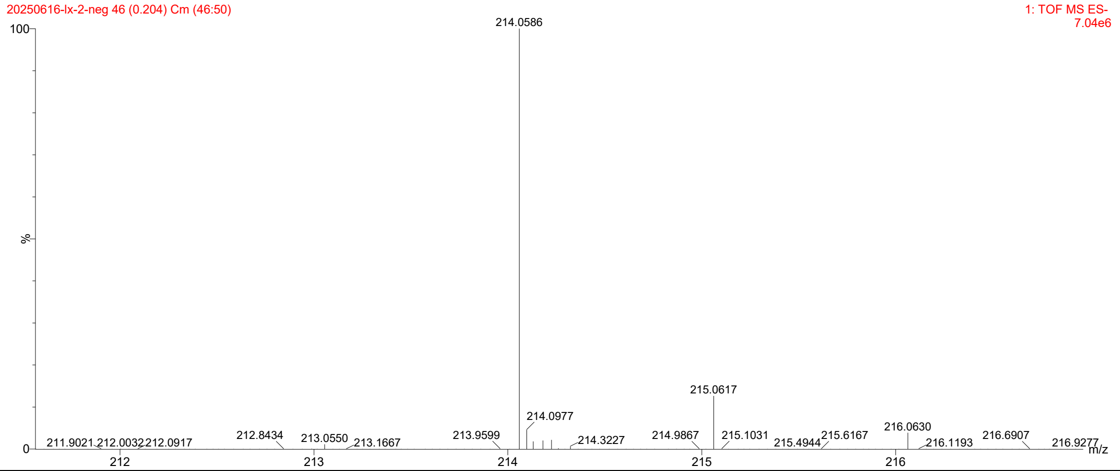

m/z	Relative Natural Abundance (%)	Observed Abundance (%)	Corrected Abundance (%)	Isotopic Enrichment Relative (%)
[M-H]+0	100	77900	77900	1
[M-H]+1	14.1	7040000	7029016.1	99

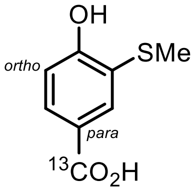

3l*

White powder (7.6 mg, 41% yield, *para*:*ortho* > 20:1, 99% ^{13}C incorporation, m.p. 119–120 °C)

Compound **3l*** was obtained from **1l** (0.1 mmol, 1.0 equiv., 14.0 mg) and **2a*-Cs** (0.2 mmol, 2.0 equiv., 84.2 mg) in DMF (0.5 mL, 0.2 M) according to **General Procedure**. The reaction mixture was allowed to stir at 80 °C for 12 hours. After purification through a series of extractions and flash column chromatography (Hexane/EtOAc (0.1% AcOH, v/v) = 4/1), the desired product **3l*** was obtained. The ^{13}C% incorporation was obtained through analysis of high-resolution mass spectrum of the compound **3l***.

^{1}H NMR (400 MHz, CD_3OD): δ 7.86 (q, J = 2.0 Hz, 1H), 7.73 (ddd, J = 8.4, 4.0, 2.0 Hz, 1H), 6.82 (dd, J = 8.4, 0.8 Hz, 1H), 2.42 (s, 3H).

^{13}C NMR (101 MHz, CD_3OD): δ 169.7, 160.5, 130.7 (d, J = 3.0 Hz), 130.0 (d, J = 2.2 Hz), 126.3 (d, J = 5.3 Hz), 123.5 (d, J = 74.4 Hz), 114.9 (d, J = 5.0 Hz), 15.3: 8 carbons.

HRMS (ESI) m/z: Calcd for $C_7[^{13}C]H_7O_3S$ $[M-H]^-$: 184.0154; Found 184.0160.

Information on Determination of ^{13}C Incorporation for compound 3l*

Corrected abundance of ^{13}C of compound 3l* = 11000000 – 114000 x 8.7 / 100 = 10990082;

% ^{13}C incorp. of compound 3l* = 10990082 / (10990082 + 114000) x 100 = 99%.

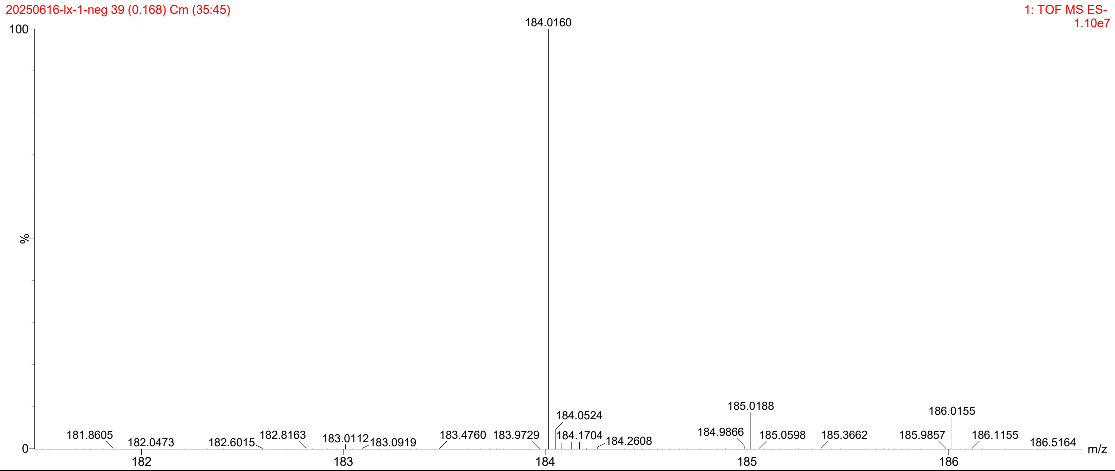

m/z	Relative Natural Abundance (%)	Observed Abundance (%)	Corrected Abundance (%)	Isotopic Enrichment Relative (%)
[M-H]+0	100	114000	114000	1
[M-H]+1	8.7	11000000	10990082	99

Failed examples

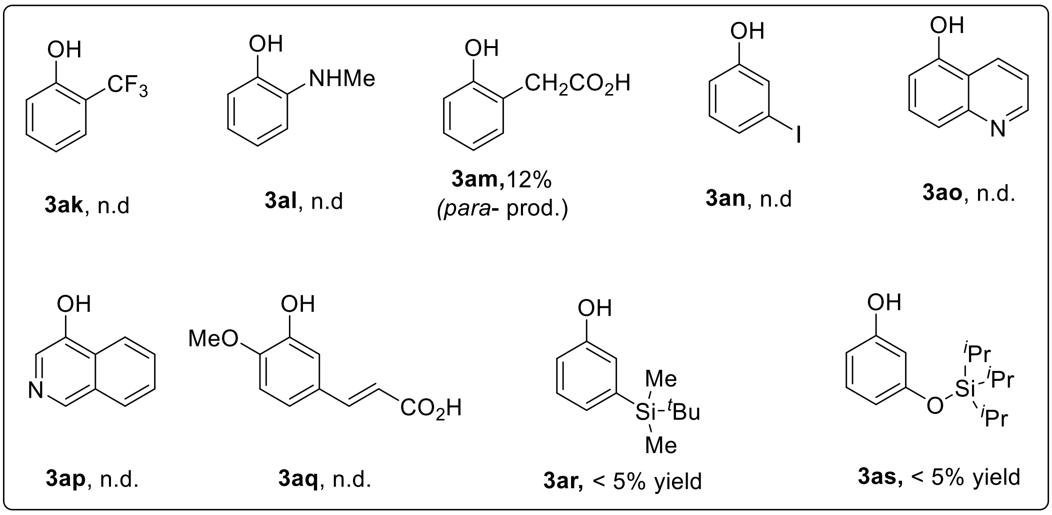

For substrates **3ak**, **3al**, **3an**, **3ao**, **3ap** and **3aq**, the reaction activity is very poor, no carboxylated product can be detected, and a large amount of the starting material is recovered.

For substrate **3am**, the reaction system is relatively complex, generating some by-products.

Substrate **3ar** exhibits poor reactivity, resulting in low yield (< 5%) and the recovery of a large amount of the starting material.

Substrate **3as** is unstable during the reaction. The O-Si bond undergoes cleavage, leading to the decomposition of the starting material, so it fails to form a significant amount of the target product.

VI. Experimental procedures for synthetic applications and associated characterization data

1) Procedure for the preparation of compound 6

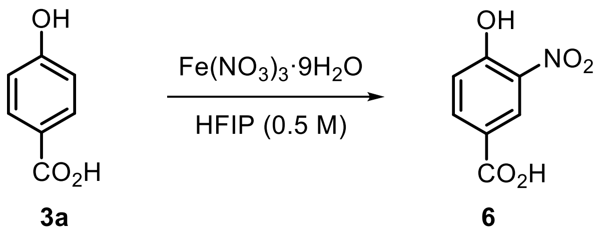

Adapted from the literature,[9] under the air condition, a 10 mL oven-dried vial equipped with a magnetic stir bar was charged with **3a** (0.5 mmol, 1.0 equiv., 69.1 mg), $Fe(NO_3)_3$•$9H_2O$ (0.5 mmol, 1.0 equiv., 202.0 mg) and HFIP (1.0 mL, 0.5 M). The reaction mixture was stirred at room temperature for 12 hours. After completion of the reaction (monitored by TLC), the solvent was removed under reduced pressure, and the crude product was purified by flash column chromatography (Hexane/EtOAc (0.1% AcOH, v/v) = 2/1) on silica gel to give the desired nitration product **6** (71.4 mg, 78% yield, m.p. 182–184 °C) as a yellow solid.

^{1}H NMR (400 MHz, CD_3OD): δ 8.64 (s, 1H), 8.15 (d, J = 8.8 Hz, 1H), 7.18 (d, J = 8.8 Hz, 1H).
^{13}C NMR (101 MHz, CD_3OD): δ 167.8, 158.3, 138.1, 135.8, 128.3, 124.3, 120.9: 7 carbons.
HRMS (ESI) m/z: Calcd for $C_7H_4NO_5$ $[M-H]^-$: 182.0094; Found 182.0098.

2) Procedure for the preparation of compound 8

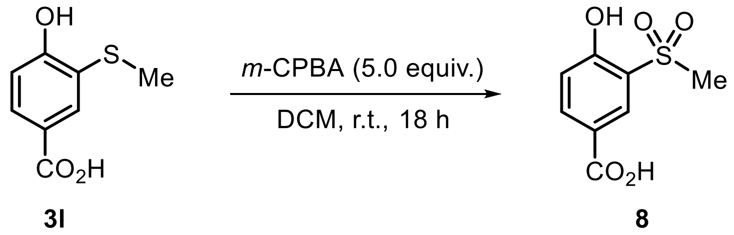

Adapted from the literature,[10] under the air condition, an oven-dried 20 mL screw-cap vial equipped with a magnetic stir bar was charged with **3l** (0.6 mmol, 1.0 equiv., 110.5 mg) and DCM (12.0 mL, 0.05 M) sequentially. Then *m*-CPBA (3.0 mmol, 5.0 equiv., 609.1 mg) was added slowly. The resulting mixture was stirred at room temperature for 18 hours. After completion of the reaction (monitored by TLC), the solvent was removed under reduced pressure. The residue was purified by flash column chromatography (Hexane/EtOAc (0.1% AcOH, v/v) = 2/1) to afford product **8** (66.1 mg, 51% yield, m.p. 198–200 °C) as a beige powder.

^{1}H NMR (400 MHz, CD_3OD): δ 8.48 (d, J = 2.0 Hz, 1H), 8.15 (dd, J = 8.8, 2.4 Hz, 1H), 7.08 (d, J = 8.4 Hz, 1H), 3.27 (s, 3H).
^{13}C NMR (101 MHz, CD_3OD): δ 168.2, 161.2, 137.9, 132.3, 127.7, 123.3, 118.2, 42.8: 8 carbons.
HRMS (ESI) m/z: Calcd for $C_8H_7O_5S$ $[M-H]^-$: 215.0019; Found 215.0025.

3) Procedure for the preparation of compound 9

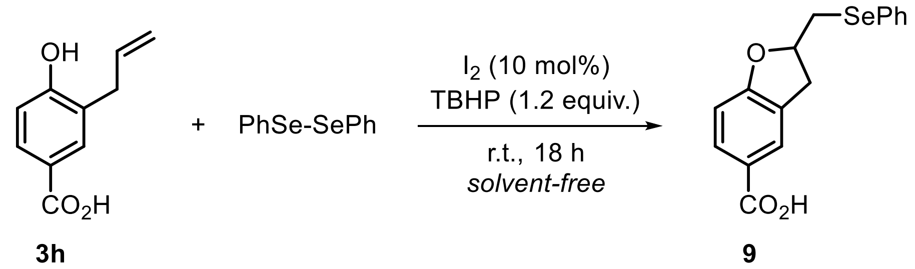

Adapted from the literature,[11] in a glovebox filled with N_2, an oven-dried 4 mL screw-cap vial equipped with a stirring bar was charged with **3h** (0.75 mmol, 1.0 equiv., 133.5 mg), diphenyl diselenide (PhSe-SePh) (0.45 mmol, 0.6 equiv., 140.4 mg) and *tert*-butyl hydroperoxide (TBHP) (0.9 mmol, 1.2 equiv., 123.6 μL) sequentially. Finally, molecular iodine (I_2) (0.075 mmol, 10 mol%, 19.0 mg) was added into the reaction. The vial was sealed with a PTFE-lined cap and removed from the glovebox. The mixture was magnetically stirred for 18 hours at room temperature (25 °C). After completion of the reaction, the crude product was purified by flash column chromatography (Hexane/EtOAc (0.1% AcOH, v/v) = 5/1) on silica gel to give the desired product **9** (140.0 mg, 56% yield, m.p. 179–181 °C) as an orange powder.

^{1}H NMR (400 MHz, $CDCl_3$): δ 12.06 (br, s, 1H), 7.96 (dd, *J* = 8.4, 1.2 Hz, 1H), 7.91 (s, 1H), 7.58 – 7.54 (m, 2H), 7.31 – 7.27 (m, 3H), 6.77 (d, *J* = 8.4 Hz, 1H), 5.09 – 5.02 (m, 1H), 3.42 – 3.31 (m, 2H), 3.15 – 3.03 (m, 2H).
^{13}C NMR (101 MHz, $CDCl_3$): δ 172.3, 164.1, 133.3 (2C), 132.1, 129.3 (2C), 129.1, 127.6, 127.6, 127.0, 122.0, 109.4, 83.5, 34.8, 32.6: 16 carbons.
HRMS (ESI) m/z: Calcd for $C_{16}H_{13}O_3Se$ [M-H]$^-$: 333.0035; Found 333.0029.

VII. X-ray crystallographic data

A single crystal of compound **3q** suitable for X-ray crystallography was obtained via slow evaporation crystallization of its methanol solution at room temperature. The structure of **3q** was then determined by X-ray crystallographic analysis at 119.0 (10) K on an Agilent Gemini E diffractometer. Crystal data and refinement parameters for **3q** (CCDC 2488331) are summarized below.

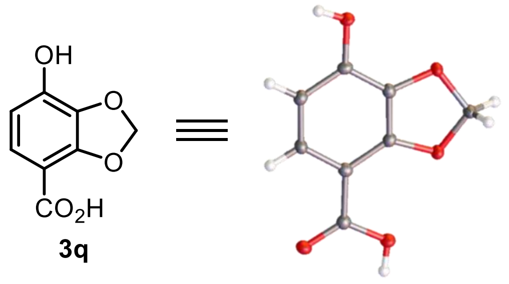

Identification code	exp_10026
Empirical formula	$C_8H_7O_{5.5}$
Formula weight	191.14
Temperature / K	119.0(10)
Crystal system	triclinic
Space group	P-1
a / Å, b / Å, c / Å	8.6449(17), 9.4956(15), 10.2500(16)
α/°, β/°, γ/°	85.823(13), 67.990(17), 76.614(15)
Volume / Å^3	758.8(2)
Z	4
ρ_{calc} / mg mm^{-3}	1.673
μ / mm^{-1}	1.263
F(000)	396
Crystal size / mm^3	0.34 × 0.23 × 0.21
2Θ range for data collection	9.3 to 133.62°
Index ranges	$-10 \le h \le 9$, $-8 \le k \le 11$, $-12 \le l \le 11$
Reflections collected	4194
Independent reflections	2587[R(int) = 0.0240 (inf-0.9Å)]
Data/restraints/parameters	2587/3/254
Goodness-of-fit on F^2	1.031
Final R indexes [I>2σ (I) i.e. F_o>4σ (F_o)]	R_1 = 0.0364, wR_2 = 0.0986
Final R indexes [all data]	R_1 = 0.0417, wR_2 = 0.1035
Largest diff. peak/hole / e Å^{-3}	0.249/-0.205
Flack Parameters	N
Completeness	0.9946

A single crystal of compound **3s** suitable for X-ray crystallography was obtained via slow evaporation crystallization of its methanol solution at room temperature. The structure of **3s** was then determined by X-ray crystallographic analysis at 116.4 (2) K on an Agilent Gemini E diffractometer. Crystal data and refinement parameters for **3s** (CCDC 2488328) are summarized below.

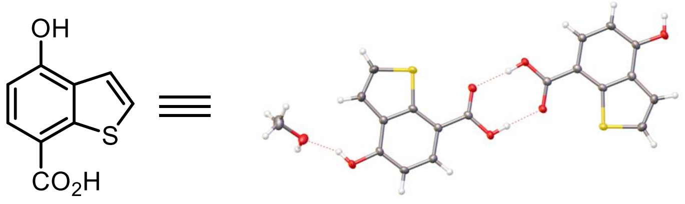

3s

Identification code	exp_10038
Empirical formula	$C_{9.5}H_8O_{3.5}S$
Formula weight	210.22
Temperature / K	116.4(2)
Crystal system	triclinic
Space group	P-1
a / Å, b / Å, c / Å	7.3343(5), 8.7623(7), 14.6810(12)
α/°, β/°, γ/°	76.765(7), 89.247(6), 82.777(6)
Volume / Å^3	911.01(12)
Z	4
ρ_{calc} / mg mm^{-3}	1.533
μ / mm^{-1}	3.027
F(000)	436
Crystal size / mm^3	0.31 × 0.27 × 0.13
2Θ range for data collection	10.46 to 133.44°
Index ranges	$-8 \le h \le 8$, $-6 \le k \le 10$, $-17 \le l \le 17$
Reflections collected	5284
Independent reflections	3124[R(int) = 0.0320 (inf-0.9Å)]
Data/restraints/parameters	3124/0/259
Goodness-of-fit on F^2	1.032
Final R indexes [I>2σ (I) i.e. F_o>4σ (F_o)]	R_1 = 0.0381, wR_2 = 0.1019
Final R indexes [all data]	R_1 = 0.0424, wR_2 = 0.1065
Largest diff. peak/hole / e Å^{-3}	0.405/-0.277
Flack Parameters	N
Completeness	0.9979

A single crystal of compound **3t-Me** suitable for X-ray crystallography was obtained via slow evaporation crystallization of its hexane/chloroform (6/1) solution at room temperature. The structure of **3t-Me** was then determined by X-ray crystallographic analysis at 117.2 (2) K on an Agilent Gemini E diffractometer. Crystal data and refinement parameters for **3t-Me** (CCDC 2488327) are summarized below.

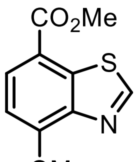

3t-Me

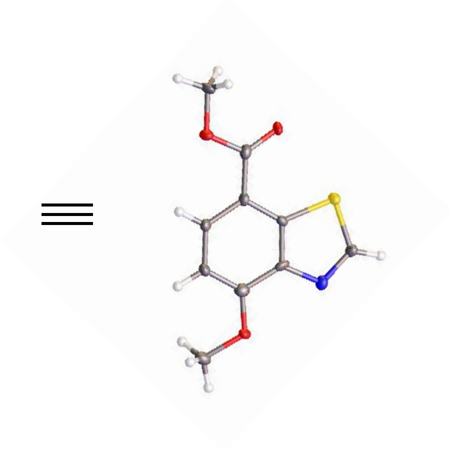

Identification code	exp_10072
Empirical formula	$C_{10}H_9NO_3S$
Formula weight	223.24
Temperature / K	117.2(2)
Crystal system	monoclinic
Space group	$P2_1$
a / Å, b / Å, c / Å	3.8191(3), 7.7927(7), 15.8598(19)
α/°, β/°, γ/°	90.00, 90.698(10), 90.00
Volume / Å^3	471.97(8)
Z	2
ρ_{calc} / mg mm^{-3}	1.571
μ / mm^{-1}	2.949
F(000)	232
Crystal size / mm^3	0.31 × 0.16 × 0.03
2Θ range for data collection	11.16 to 132.82°
Index ranges	$-4 \le h \le 4$, $-8 \le k \le 9$, $-16 \le l \le 18$
Reflections collected	2680
Independent reflections	1613[R(int) = 0.0372 (inf-0.9Å)]
Data/restraints/parameters	1613/1/138
Goodness-of-fit on F^2	1.189
Final R indexes [I>2σ (I) i.e. F_o>4σ (F_o)]	R_1 = 0.0784, wR_2 = 0.2269
Final R indexes [all data]	R_1 = 0.0793, wR_2 = 0.2278
Largest diff. peak/hole / e Å^{-3}	0.853/-0.603
Flack Parameters	0.05(6)
Completeness	0.9988

A single crystal of compound **3ad′** suitable for X-ray crystallography was obtained via slow evaporation crystallization of its methanol solution at room temperature. The structure of **3ad′** was then determined by X-ray crystallographic analysis at 116.8 (3) K on an Agilent Gemini E diffractometer. Crystal data and refinement parameters for **3ad′** (CCDC 2488332) are summarized below.

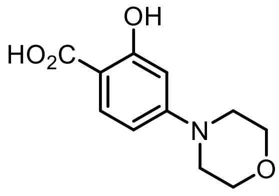

3ad'

≡

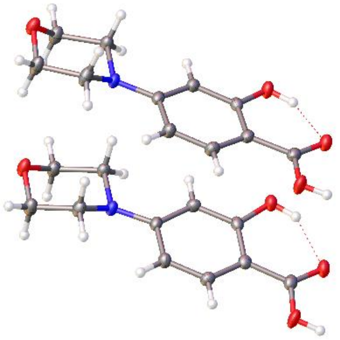

Identification code	exp_10016
Empirical formula	$C_{11}H_{13}NO_4$
Formula weight	223.22
Temperature / K	116.8(3)
Crystal system	monoclinic
Space group	$P2_1/n$
a / Å, b / Å, c / Å	10.5629(7), 16.6731(5), 11.7683(3)
α/°, β/°, γ/°	90.00, 97.007(3), 90.00
Volume / $Å^3$	2057.11(16)
Z	8
ϱ_{calc} / mg mm^{-3}	1.442
μ / mm^{-1}	0.928
F(000)	944
Crystal size / mm^3	0.11 × 0.05 × 0.03
2Θ range for data collection	9.24 to 132.92°
Index ranges	$-12 \le h \le 12$, $-18 \le k \le 19$, $-9 \le l \le 13$
Reflections collected	7225
Independent reflections	3532[R(int) = 0.0464 (inf-0.9Å)]
Data/restraints/parameters	3532/0/293
Goodness-of-fit on F^2	1.043
Final R indexes [I>2σ (I) i.e. F_o>4σ (F_o)]	R_1 = 0.0469, wR_2 = 0.1119
Final R indexes [all data]	R_1 = 0.0630, wR_2 = 0.1251
Largest diff. peak/hole / e $Å^{-3}$	0.229/-0.210
Flack Parameters	N
Completeness	0.9982

A single crystal of compound **3ag′** suitable for X-ray crystallography was obtained via slow evaporation crystallization of its methanol solution at room temperature. The structure of **3ag′** was then determined by X-ray crystallographic analysis at 114.0 (3) K on an Agilent Gemini E diffractometer. Crystal data and refinement parameters for **3ag′** (CCDC 2488330) are summarized below.

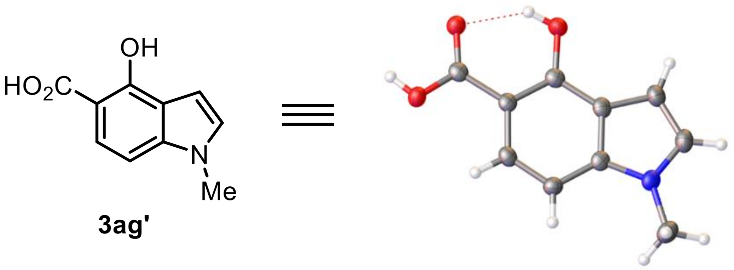

Identification code	exp_10037
Empirical formula	$C_{10}H_9NO_3$
Formula weight	191.18
Temperature / K	114.0(3)
Crystal system	monoclinic
Space group	$P2_1/c$
a / Å, b / Å, c / Å	6.5145(3), 21.1549(14), 12.8281(6)
α/°, β/°, γ/°	90.00, 101.237(5), 90.00
Volume / $Å^3$	1734.01(16)
Z	8
ρ_{calc} / mg mm^{-3}	1.465
μ / mm^{-1}	0.918
F(000)	800
Crystal size / mm^3	0.32 × 0.30 × 0.02
2Θ range for data collection	8.18 to 132.92°
Index ranges	$-5 \le h \le 7, -22 \le k \le 24, -12 \le l \le 15$
Reflections collected	5852
Independent reflections	2974[R(int) = 0.0452 (inf-0.9Å)]
Data/restraints/parameters	2974/0/259
Goodness-of-fit on F^2	1.054
Final R indexes [I>2σ (I) i.e. F_o>4σ (F_o)]	R_1 = 0.0545, wR_2 = 0.1396
Final R indexes [all data]	R_1 = 0.0694, wR_2 = 0.1564
Largest diff. peak/hole / e $Å^{-3}$	0.306/-0.251
Flack Parameters	N
Completeness	0.9979

VIII. Mechanistic studies and associated characterization data

1) Deuterium labeling experiment and associated characterization data

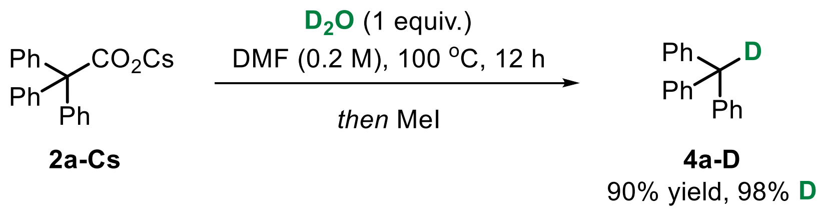

In a glovebox filled with N_2, a 2 mL thick-walled pressure-resistant tube equipped with a stirring bar was charged with cesium 2,2,2-triphenylacetate **2a-Cs** (0.4 mmol, 2.0 equiv., 168.1 mg). In a separate vial, a solution of H_2O (200 μL) in DMF (20 mL) was prepared. 1.0 mL of the H_2O/DMF solution was added into the vial containing cesium 2,2,2-triphenylacetate **2a-Cs**. The tube was sealed with a PTFE threaded sealing cap and removed from the glovebox. The reaction mixture was transferred to a preheated stir plate and stirred at 100 °C in an aluminum block for 12 hours. After that time, the reaction was cooled down to room temperature (25 °C). Then methyl iodide (MeI) (1.6 mmol, 8.0 equiv., 100 μL) was added into the reaction and the mixture was stirred at 40 °C for 4 hours. The reaction was cooled to room temperature and the reaction quenched with saturated $NaHCO_3$ (aq., 5 mL). The reaction mixture was extracted with EtOAc (5 mL × 4), dried over Na_2SO_4, and then concentrated under reduced pressure. The crude reaction mixture was analyzed by ^{1}H NMR using 1,3,5-Trimethoxybenzene as an internal standard. The deuterium incorporation (98%) of **4a-D** was determined by ^{1}H NMR analysis.

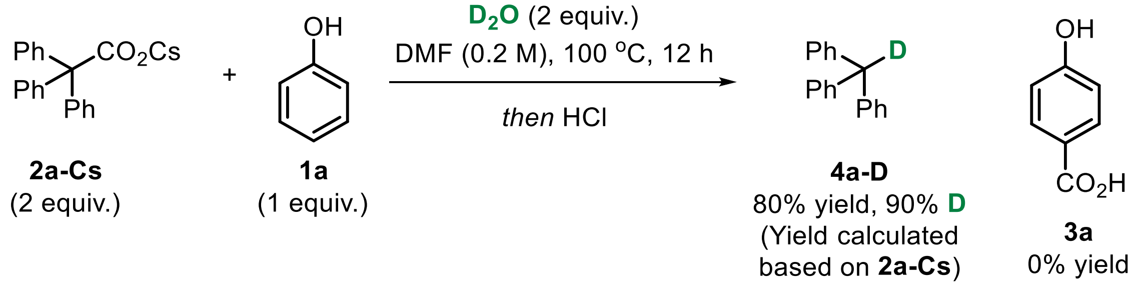

In a glovebox filled with N_2, a 2 mL thick-walled pressure-resistant tube equipped with a stirring bar was charged with phenol **1a** (0.2 mmol, 1.0 equiv., 18.8 mg) and cesium 2,2,2-triphenylacetate **2a-Cs** (0.4 mmol, 2.0 equiv., 168.1 mg). In a separate vial, a solution of H_2O (200 μL) in DMF (20 mL) was prepared. 1.0 mL of the H_2O/DMF solution was added into the vial containing cesium 2,2,2-triphenylacetate **2a-Cs** and **1a**. The tube was sealed with a PTFE threaded sealing cap and removed from the glovebox. The reaction mixture was transferred to a preheated stir plate and stirred at 100 °C in an aluminum block for 12 hours. After that time, the reaction was cooled down to room temperature (25 °C). The mixture was diluted with H_2O (5 mL) and washed with EtOAc (3 x 6 mL). Then the aqueous layer was acidified using 1 M HCl (2 mL) solution. The aqueous layer was extracted with EtOAc (3 x 6 mL) and the combined organic layers were washed with

1:1 mixture of H_2O and brine (8 × 2 mL), dried over anhydrous Na_2SO_4, filtered, and then concentrated in vacuo. No carboxylic acid product **3a** was obtained. And the organic layer was purified by flash column chromatography on silica gel (hexane/EtOAc gradient = 100:1) to give **Ph_3CD** as a white solid (78.5 mg, 80% yield). The deuterium incorporation (90%) of **Ph_3CD** was determined by ^1H NMR analysis.

^1H NMR (400 MHz, $CDCl_3$): δ 7.24 (t, *J* = 7.4 Hz, 6H), 7.19 – 7.15 (m, 3H), 7.10 (d, *J* = 7.2 Hz, 6H).

^{13}C NMR (101 MHz, $CDCl_3$): δ 144.0, 129.6, 128.4, 126.4, 56.5 (t, *J* = 20.2 Hz).

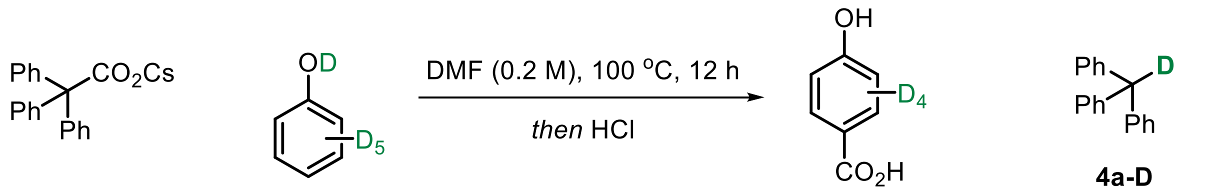

2a-Cs (2 equiv.) **1a-D** (1 equiv.) **3a-D** 80% yield **4a-D** 89% yield, 90% D (Yield calculated based on **2a-Cs**)

In a glovebox filled with N_2, a 2 mL thick-walled pressure-resistant tube equipped with a stirring bar was charged with phenol **1a-D** (0.2 mmol, 1.0 equiv., 20.0 mg), cesium 2,2,2-triphenylacetate **2a-Cs** (0.4 mmol, 2.0 equiv., 168.1 mg) and DMF (1 mL, 0.2 M). The tube was sealed with a PTFE threaded sealing cap and removed from the glovebox. The reaction mixture was transferred to a preheated stir plate and stirred at 100 °C in an aluminum block for 12 hours. After that time, the reaction was cooled down to room temperature (25 °C). The mixture was diluted with H_2O (6 mL) and washed with EtOAc (3 x 6 mL). Collecting the aqueous phase and the organic phase separately. The aqueous layer was acidified using 1 M HCl (2 mL) solution. After acidification, the aqueous layer was extracted with EtOAc (3 x 6 mL) and the combined organic layers were washed with 1:1 mixture of H_2O and brine (8 × 2 mL), dried over anhydrous Na_2SO_4, filtered, and then concentrated in vacuo. The crude product was analyzed by GC using 1,2,4,5-tetramethylbenzene as an internal standard. The yield of the target product **3a-D** is 80%. Additionally, the aforementioned organic layer was purified by flash column chromatography on silica gel (hexane/EtOAc gradient = 100:1) to give **4a-D** as a white solid (87.3 mg, 89% yield). The deuterium incorporation (90%) of **4a-D** was determined by ^1H NMR analysis.

2) The control experiments carried out with crown ethers

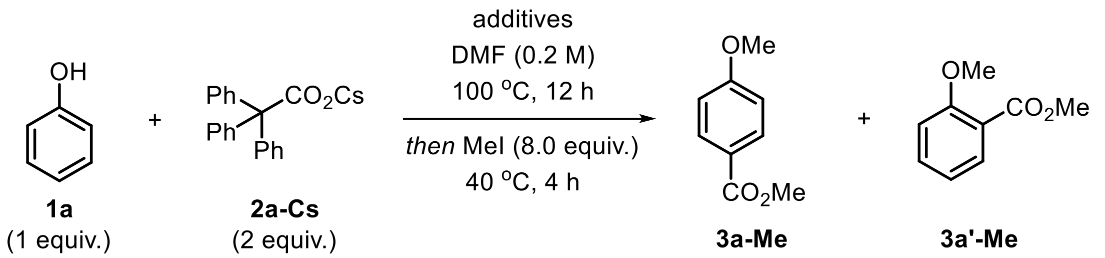

Entry	additives	**3a-Me** (%)[a]	**3a'-Me** (%)[a]	(**3a-Me** : **3a'-Me**)[b]
1	18-crown-6 (0.5 equiv.)	82	2	41:1
2	18-crown-6 (1 equiv.)	82	n.d.	-
3	18-crown-6 (2 equiv.)	77	n.d.	-
4	[2,4]-dibenzo-18-crown-6 (0.5 equiv.)	92	1	92:1
5	[2,4]-dibenzo-18-crown-6 (1 equiv.)	87	1	87:1
6	[2,4]-dibenzo-18-crown-6 (2 equiv.)	81	1	81:1
7	Kryptofix® 222 (0.5 equiv.)	92	1	92:1
8	Kryptofix® 222 (1 equiv.)	88	2	44:1
9	Kryptofix® 222 (2 equiv.)	88	2	44:1

Reaction conditions: **1a** (0.2 mmol, 1 equiv.), **2a-Cs** (2 equiv.), additive (x equiv.), DMF (1 mL, 0.2 M), 100 °C, 12 h. Then MeI (1.6 mmol, 8.0 equiv., 100 μL), 40 °C, 4 h. [a] Yield was determined by GC using 1,2,4,5-tetramethylbenzene as the internal standard. [b] Selectivity (**3a-Me** : **3a'-Me**) was determined by GC using 1,2,4,5-tetramethylbenzene as the internal standard. n.d. = not detected.

3) Investigation on the possibility of *ortho*-to-*para* rearrangement pathway

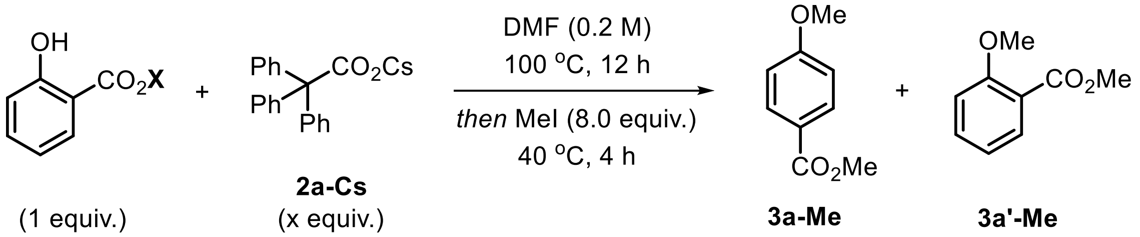

Entry		X	**2a-Cs**	**3a-Me** (%)[a]	**3a'-Me** (%)[a]
1	**3a'**	**H**	2.0 equiv.	n.d.	98
2	**3a'**	**H**	3.0 equiv.	n.d.	98
3	**3a'-Cs**	**Cs**	1.0 equiv.	n.d.	96
4	**3a'-Cs**	**Cs**	2.0 equiv.	n.d.	98

Reaction conditions: **3a'** or **3a'-Cs** (0.2 mmol, 1.0 equiv.), **2a-Cs** (x equiv.), DMF (1 mL, 0.2 M), 100 °C, 12 h. Then MeI (1.6 mmol, 8.0 equiv., 100 μL), 40 °C, 4 h. [a] Yield was determined by GC using 1,2,4,5-tetramethylbenzene as the internal standard. n.d. = not detected.

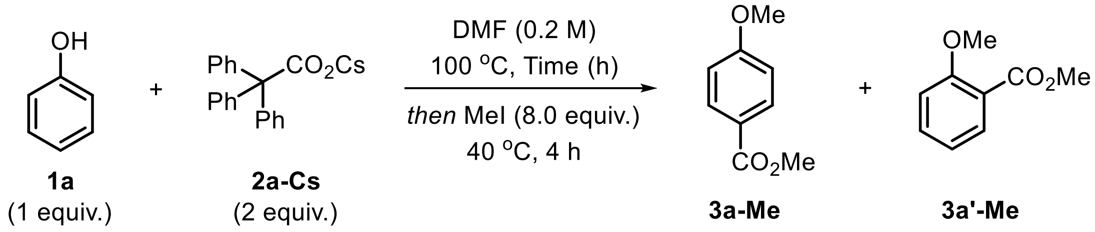

Entry	Time (h)	**3a-Me** (%)[a]	**3a'-Me** (%)[a]	(**3a-Me** : **3a'-Me**)[b]
1	0.5	62	3	21:1
2	1	80	3	27:1
3	1.5	82	3	27:1
4	2	86	3	28:1
5	3	87	3	29:1
6	4	87	3	29:1
7	6	87	3	29:1
8	8	89	3	30:1
9	12	92	3	31:1

Reaction conditions: **1a** (0.2 mmol, 1 equiv.), **2a-Cs** (2 equiv.), DMF (1 mL, 0.2 M), 100 °C. Then MeI (1.6 mmol, 8.0 equiv., 100 μL), 40 °C, 4 h. [a] Yield was determined by GC using 1,2,4,5-tetramethylbenzene as the internal standard. [b] Selectivity (**3a-Me** : **3a'-Me**) was determined by GC using 1,2,4,5-tetramethylbenzene as the internal standard. n.d. = not detected.

IX. References

1. S. Wang, I. Larrosa, H. Yorimitsu and G. J. P. Perry, *Angew. Chem. Int. Ed.*, **2023**, *62*, e202218371.

2. M. Toshio, M. Kenji, Y. Nagahisa, M. Akihisa. World Intellectual Property Organization, WO2010113022 A1, **2010**.

3. A. Cervi, Y. Vo, C. L. L. Chai, M. G. Banwell, P. Lan and A. C. Willis, *J. Org. Chem.*, **2021**, *86*, 178-198.

4. C. Medena, C. Aubert, E. Derat, L. Fensterbank, G. Gontard, O. Khaled, C. Ollivier, N. Vanthuyne, M. Petit and M. Barbazanges, *ChemCatChem*, **2021**, *13*, 4543-4548.

5. H. Yu, S. Ru, G. Dai, Y. Zhai, H. Lin, S. Han and Y. Wei, *Angew. Chem. Int. Ed.*, **2017**, *56*, 3867-3871.

6. T. J. W. Jacques Dit Lapierre, M. G. F. d. M. L. Cruz, N. P. F. Brito, D. d. M. Resende, F. d. O. Souza, E. J. Pilau, M. F. B. da Silva, B. J. Neves, S. M. F. Murta and C. d. O. Rezende Júnior, *Eur. J. Med. Chem.*, **2023**, *256*, 115445.

7. C. Pramanik, S. Kotharkar, P. Patil, D. Gotrane, Y. More, A. Borhade, B. Chaugule, T. Khaladkar, K. Neelakandan, A. Chaudhari, M. G. Kulkarni, N. K. Tripathy and M. K. Gurjar, *Org. Process Res. Dev.*, **2014**, *18*, 152-156.

8. T. Govender, L. C. Chetty, H. G. Kruger, P. I. Arvidsson and T. Naicker, *Synthesis*, **2022**, *54*, 4827-4833.

9. Y. Zheng, Q.-Q. Hu, Q. Huang and Y. Xie, *Org. Lett.*, **2024**, *26*, 3316-3320.

10. C.-P. Zhang, T.-Z. Wang and Y.-F. Liang, *Chem. Commun.*, **2023**, *59*, 14439-14442.

11. R. H. Bartz, P. S. Souza, L. E. B. Iarocz, P. S. Hellwig, R. G. Jacob, M. S. Silva, E. J. Lenardão and G. Perin, *Eur. J. Org. Chem.*, **2025**, *28*, e202401243.

X. NMR spectra

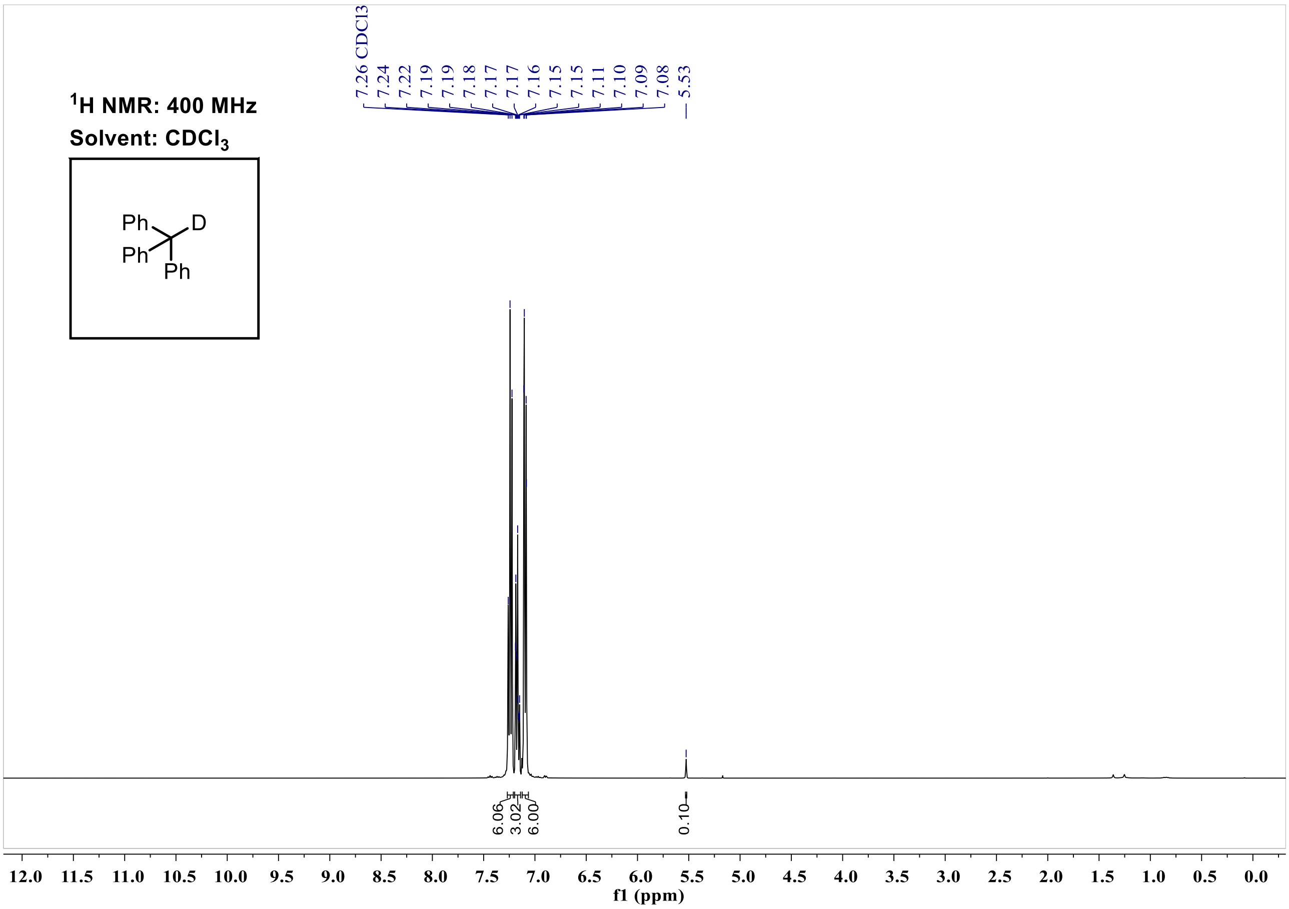

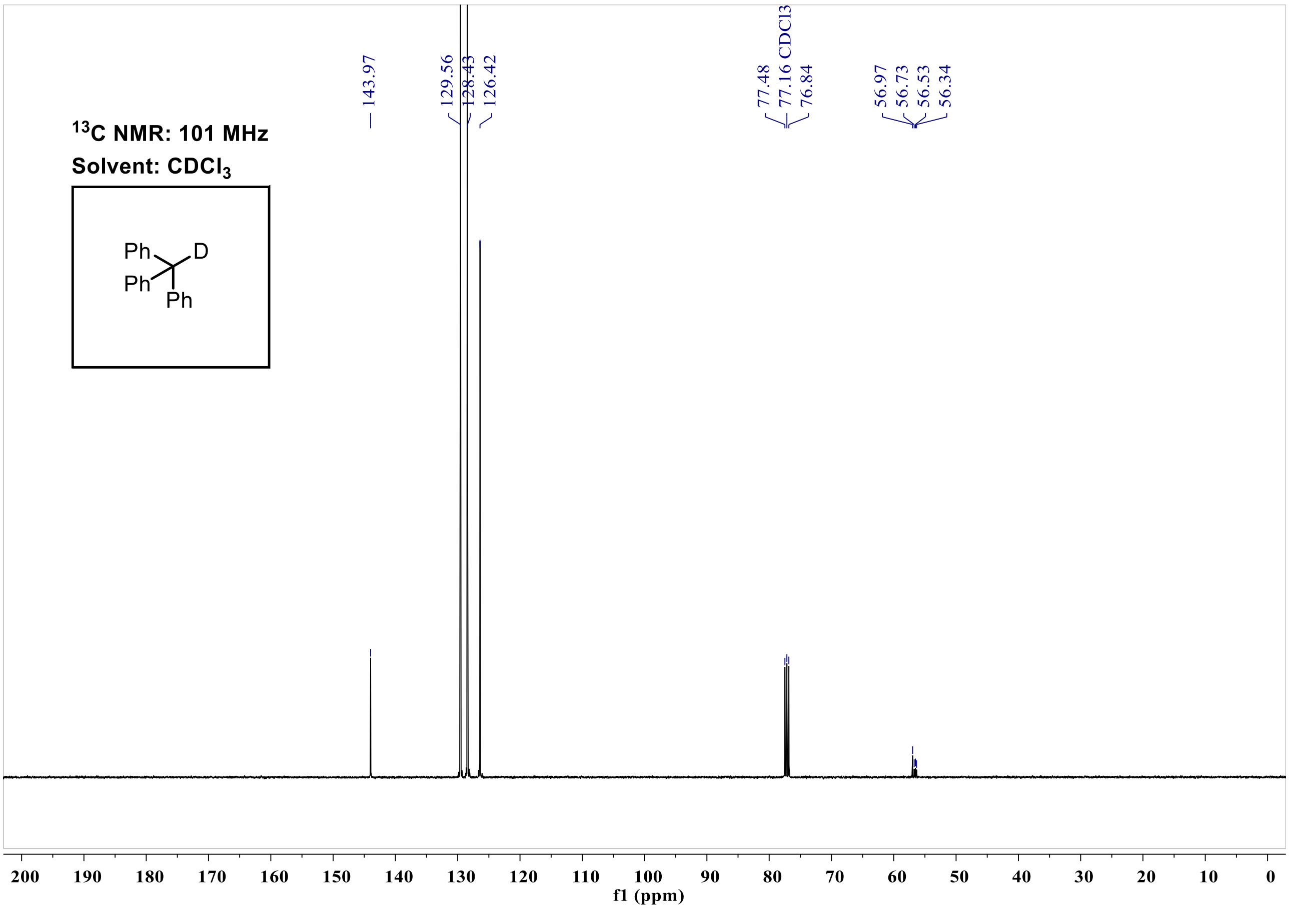

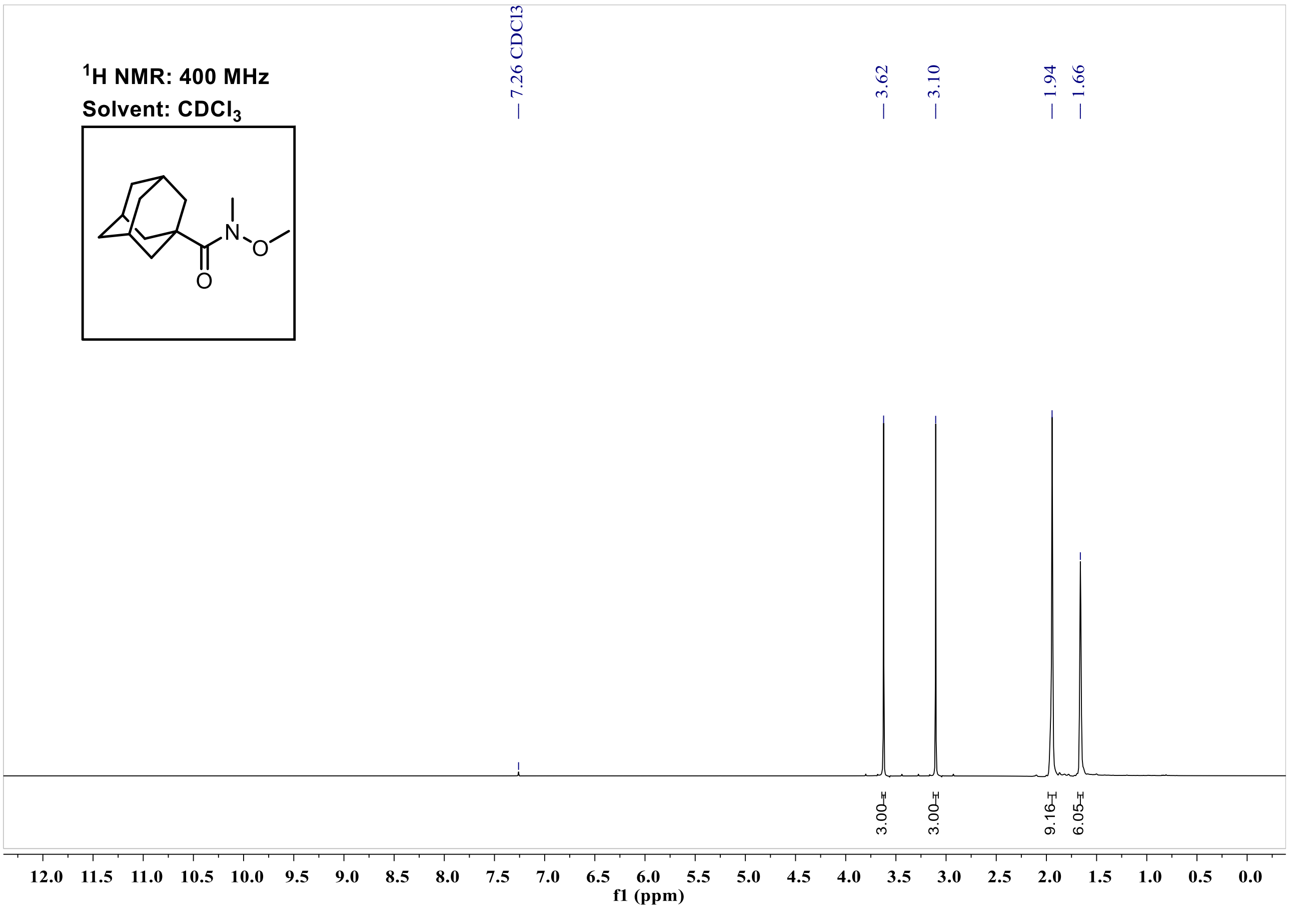

1H NMR: 400 MHz
Solvent: CDCl3
7.26 CDCl3
3.62
3.10
1.94
1.66
3.00
3.00
9.16
6.05
12.0 11.5 11.0 10.5 10.0 9.5 9.0 8.5 8.0 7.5 7.0 6.5 6.0 5.5 5.0 4.5 4.0 3.5 3.0 2.5 2.0 1.5 1.0 0.5 0.0
f1 (ppm)

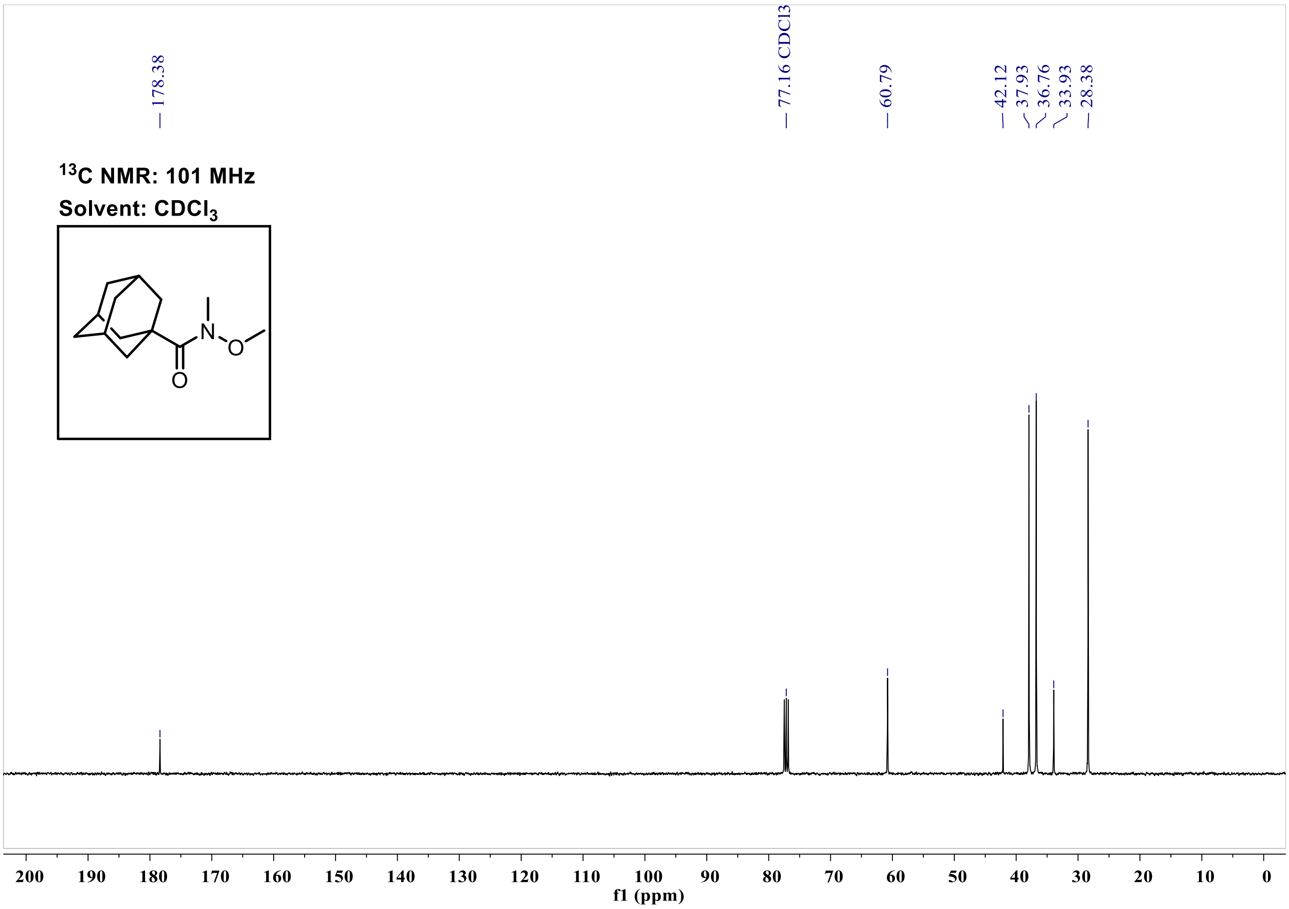

178.38
77.16 CDCl3
60.79
42.12
37.93
36.76
33.93
28.38
13C NMR: 101 MHz
Solvent: CDCl3
200 190 180 170 160 150 140 130 120 110 100 90 80 70 60 50 40 30 20 10 0
f1 (ppm)

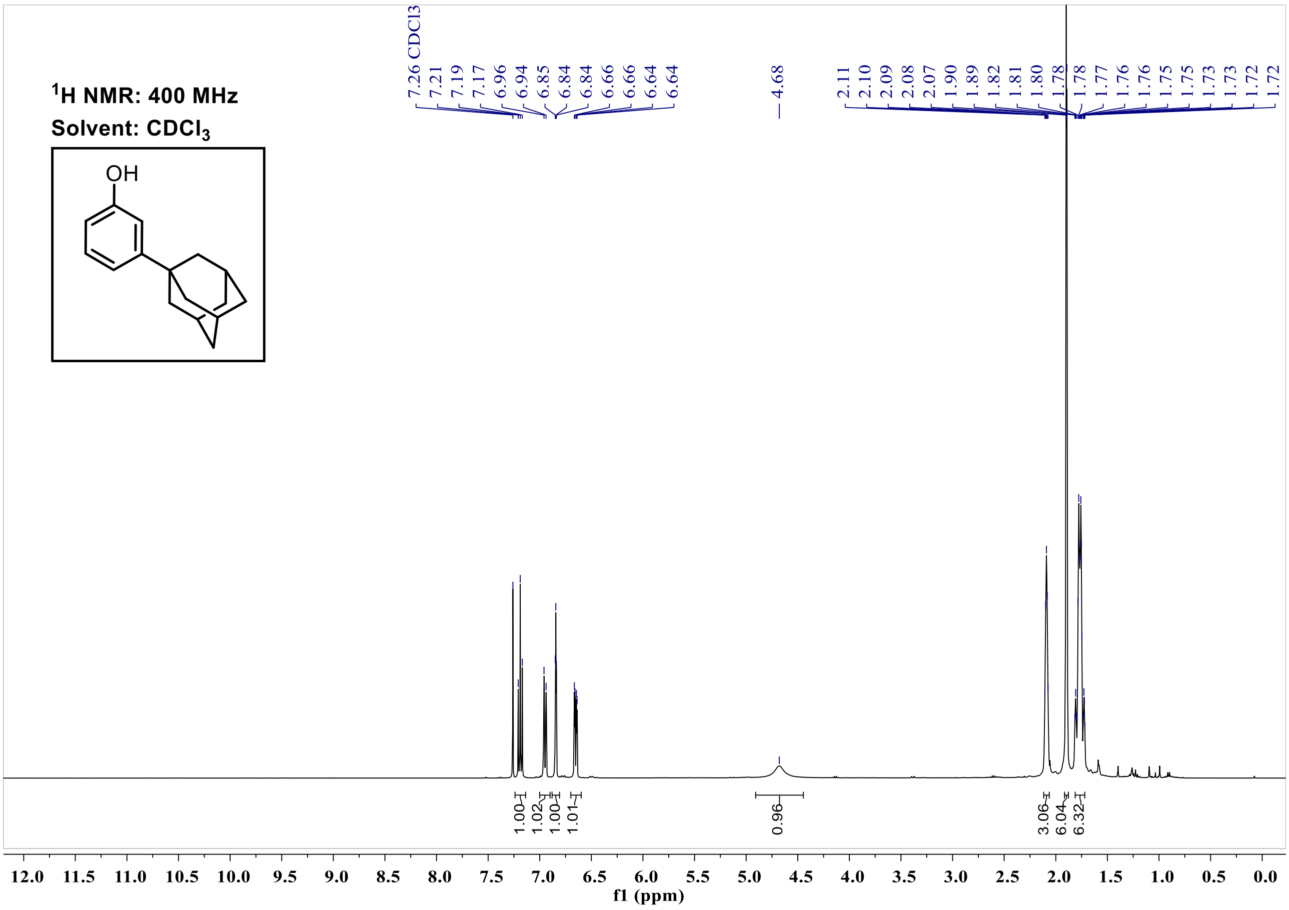

^{1}H NMR: 400 MHz
Solvent: CDCl$_3$
OH
7.26 CDCl3
7.21
7.19
7.17
6.96
6.94
6.85
6.84
6.84
6.66
6.66
6.64
6.64
4.68
2.11
2.10
2.09
2.08
2.07
1.90
1.89
1.82
1.81
1.80
1.78
1.78
1.77
1.76
1.76
1.75
1.75
1.73
1.73
1.72
1.72
1.00
1.02
1.00
1.01
0.96
3.06
6.04
6.32
12.0 11.5 11.0 10.5 10.0 9.5 9.0 8.5 8.0 7.5 7.0 6.5 6.0 5.5 5.0 4.5 4.0 3.5 3.0 2.5 2.0 1.5 1.0 0.5 0.0
f1 (ppm)

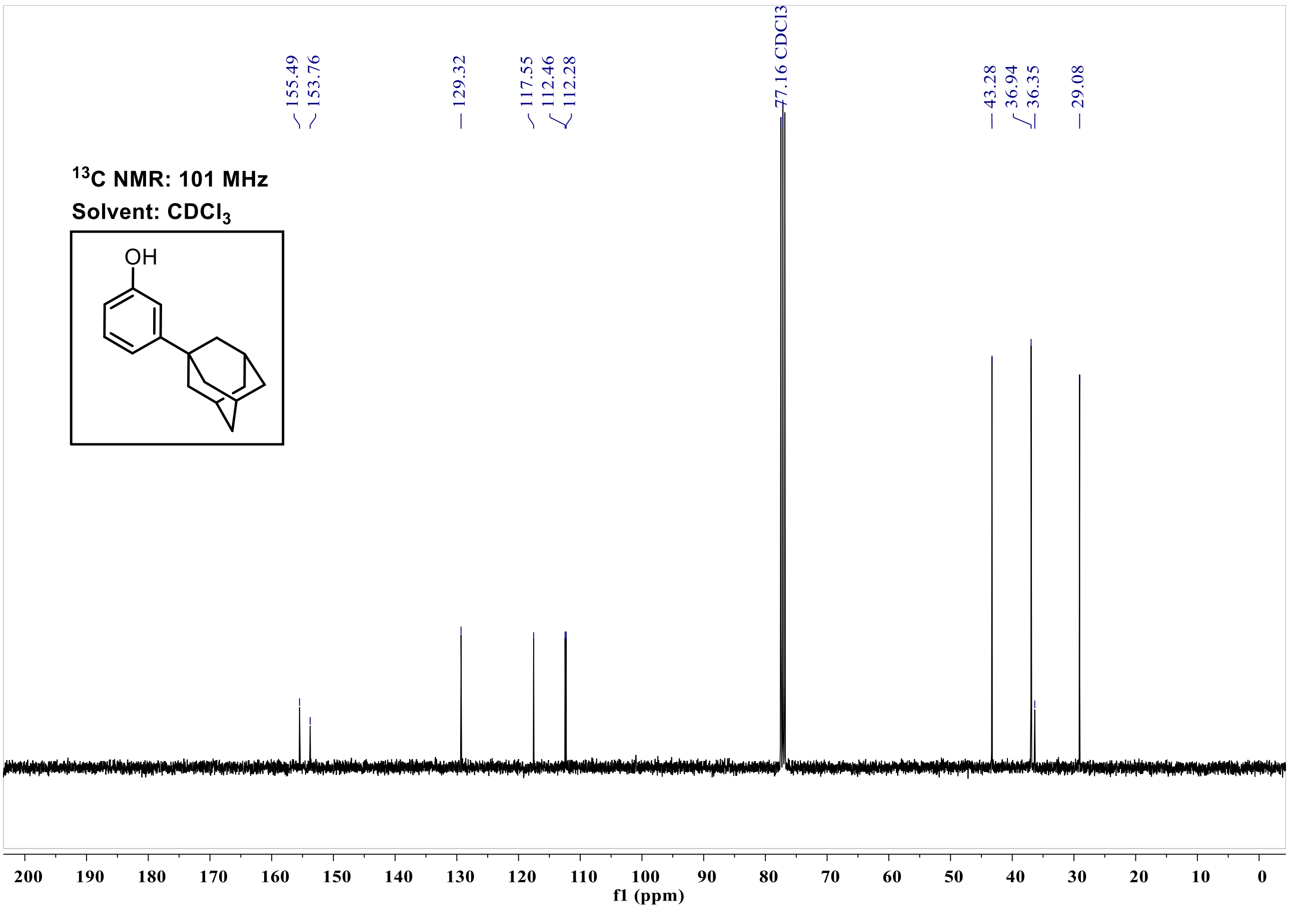

^{13}C NMR: 101 MHz
Solvent: CDCl$_3$
OH
155.49
153.76
129.32
117.55
112.46
112.28
77.16 CDCl3
43.28
36.94
36.35
29.08
200 190 180 170 160 150 140 130 120 110 100 90 80 70 60 50 40 30 20 10 0
f1 (ppm)

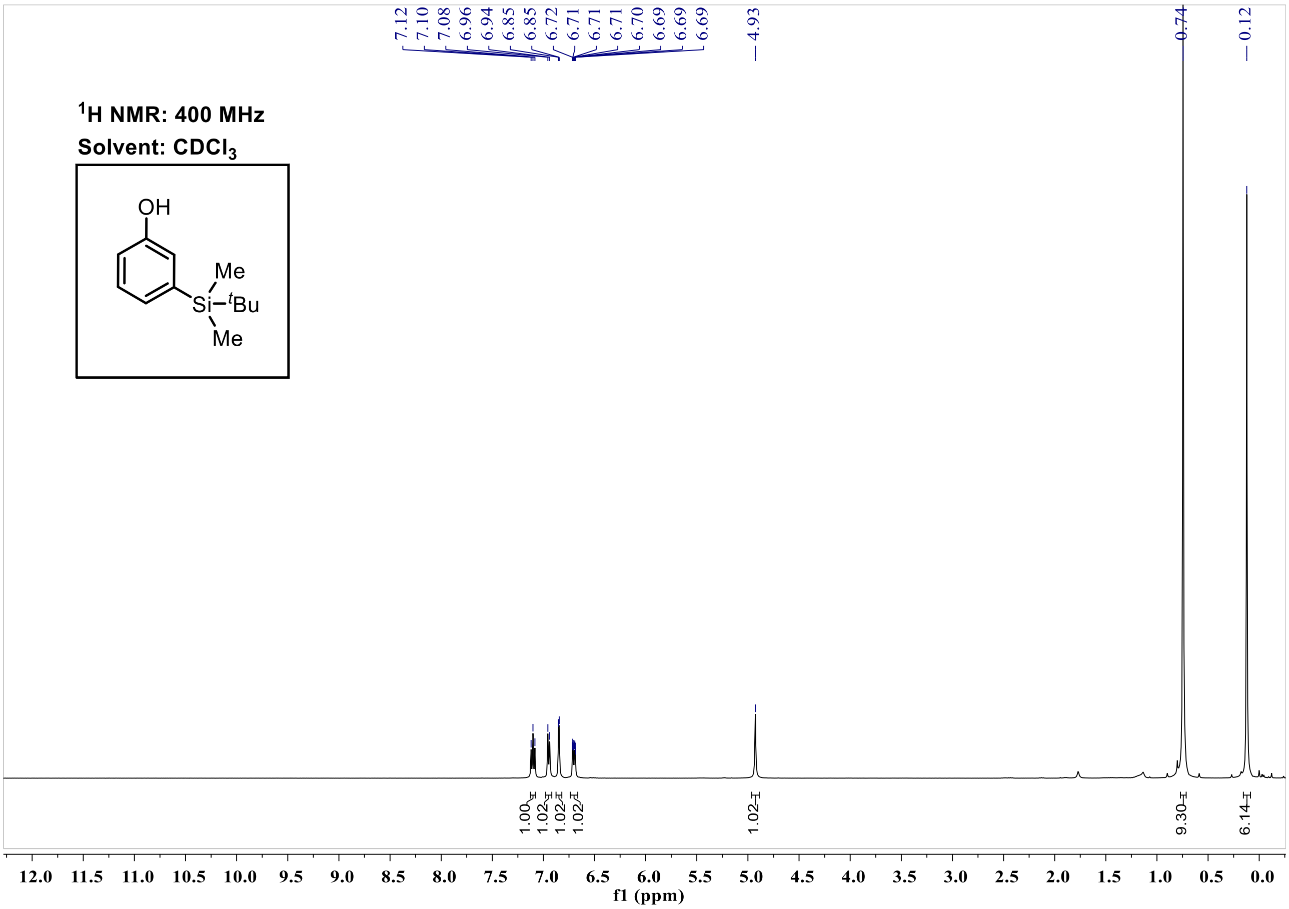

7.12
7.10
7.08
6.96
6.94
6.85
6.72
6.71
6.71
6.71
6.70
6.69
6.69
6.69
4.93
0.74
0.12
¹H NMR: 400 MHz
Solvent: CDCl3
OH
Me
Si–ᵗBu
Me
1.00
1.02
1.02
1.02
1.02
9.30
6.14
12.0 11.5 11.0 10.5 10.0 9.5 9.0 8.5 8.0 7.5 7.0 6.5 6.0 5.5 5.0 4.5 4.0 3.5 3.0 2.5 2.0 1.5 1.0 0.5 0.0
f1 (ppm)

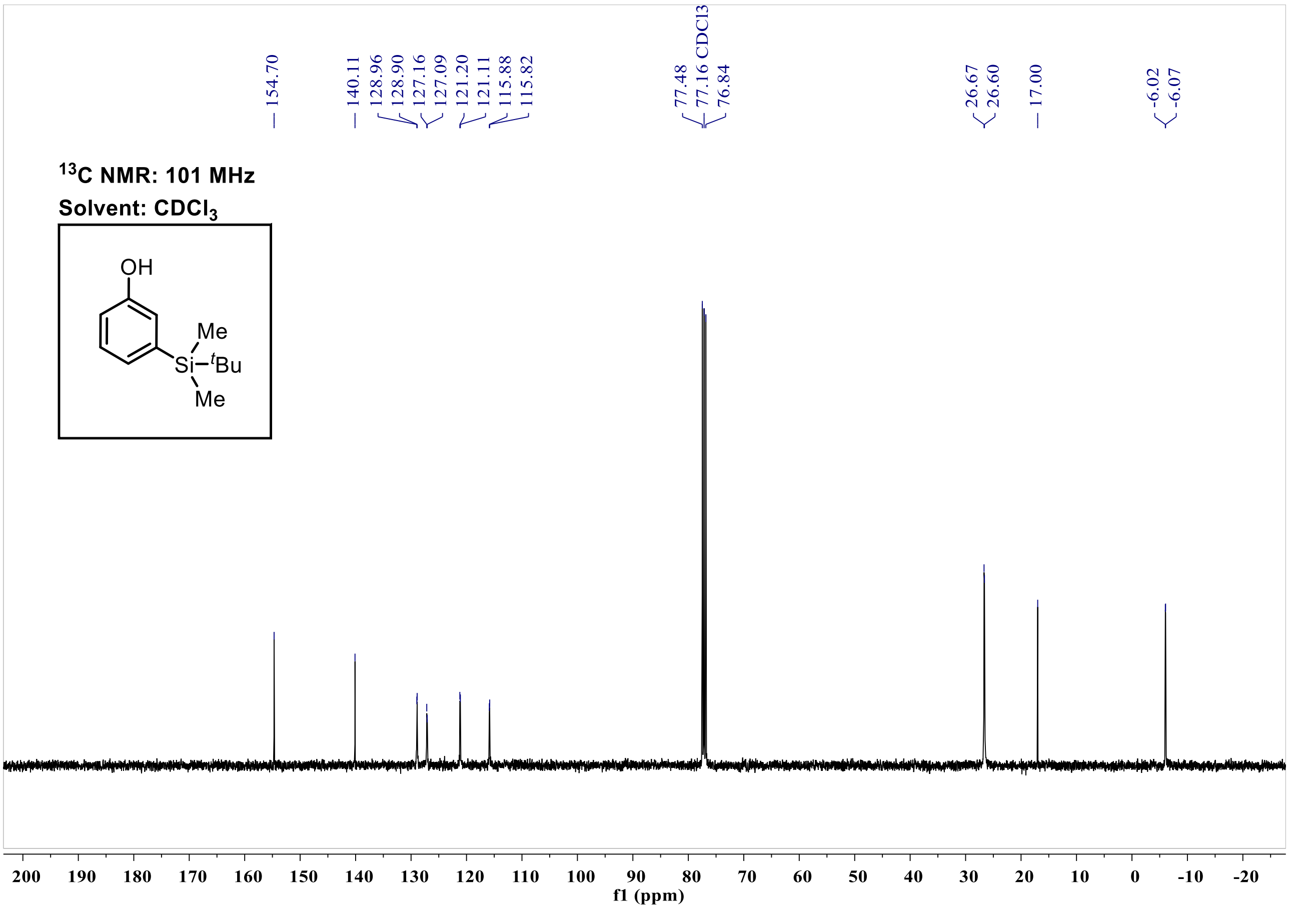

154.70
140.11
128.96
128.90
127.16
127.09
121.20
121.11
115.88
115.82
77.48
77.16 CDCl3
76.84
26.67
26.60
17.00
-6.02
-6.07
¹³C NMR: 101 MHz
Solvent: CDCl3
OH
Me
Si–ᵗBu
Me
200 190 180 170 160 150 140 130 120 110 100 90 80 70 60 50 40 30 20 10 0 -10 -20
f1 (ppm)

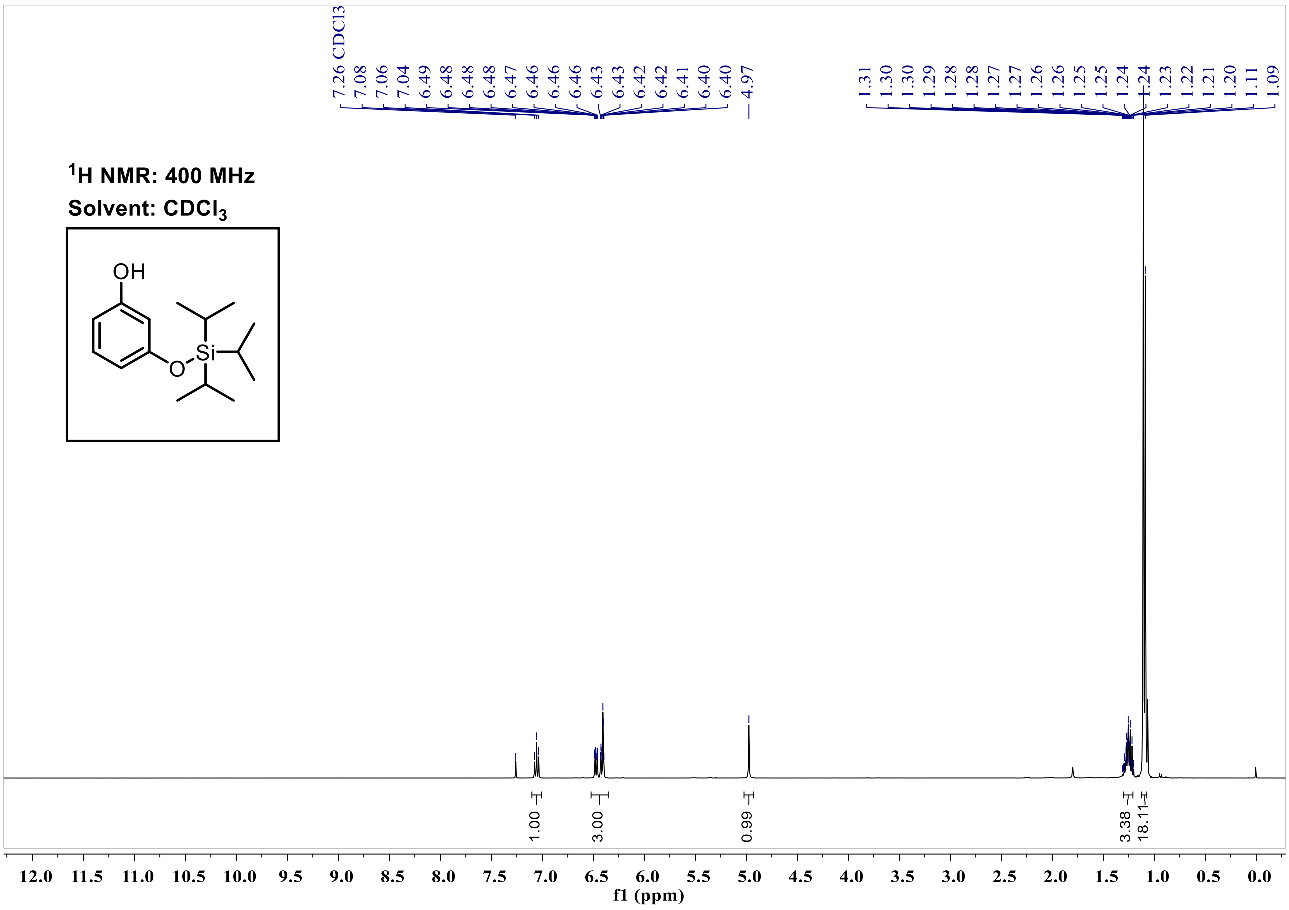

7.26 CDCl3
7.08
7.06
7.04
6.49
6.48
6.48
6.48
6.47
6.46
6.46
6.46
6.43
6.43
6.42
6.42
6.41
6.40
6.40
4.97
1.31
1.30
1.30
1.29
1.28
1.28
1.27
1.27
1.26
1.26
1.25
1.25
1.24
1.24
1.23
1.22
1.21
1.20
1.11
1.09
^{1}H NMR: 400 MHz
Solvent: $CDCl_3$
OH
O
Si
1.00
3.00
0.99
3.38
18.11
12.0 11.5 11.0 10.5 10.0 9.5 9.0 8.5 8.0 7.5 7.0 6.5 6.0 5.5 5.0 4.5 4.0 3.5 3.0 2.5 2.0 1.5 1.0 0.5 0.0
f1 (ppm)

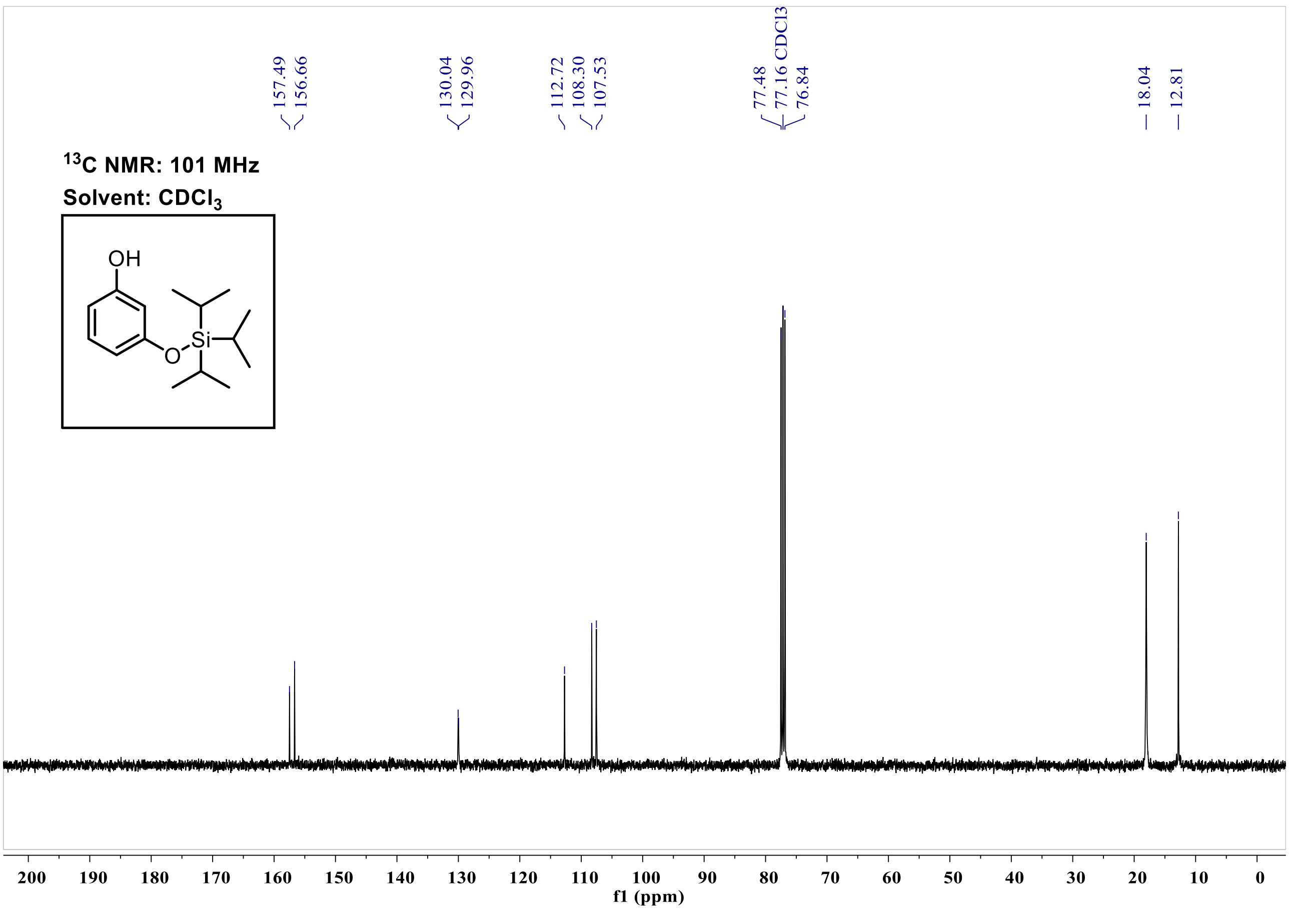

157.49
156.66
130.04
129.96
112.72
108.30
107.53
77.48
77.16 CDCl3
76.84
18.04
12.81
^{13}C NMR: 101 MHz
Solvent: $CDCl_3$
OH
O
Si
200 190 180 170 160 150 140 130 120 110 100 90 80 70 60 50 40 30 20 10 0
f1 (ppm)

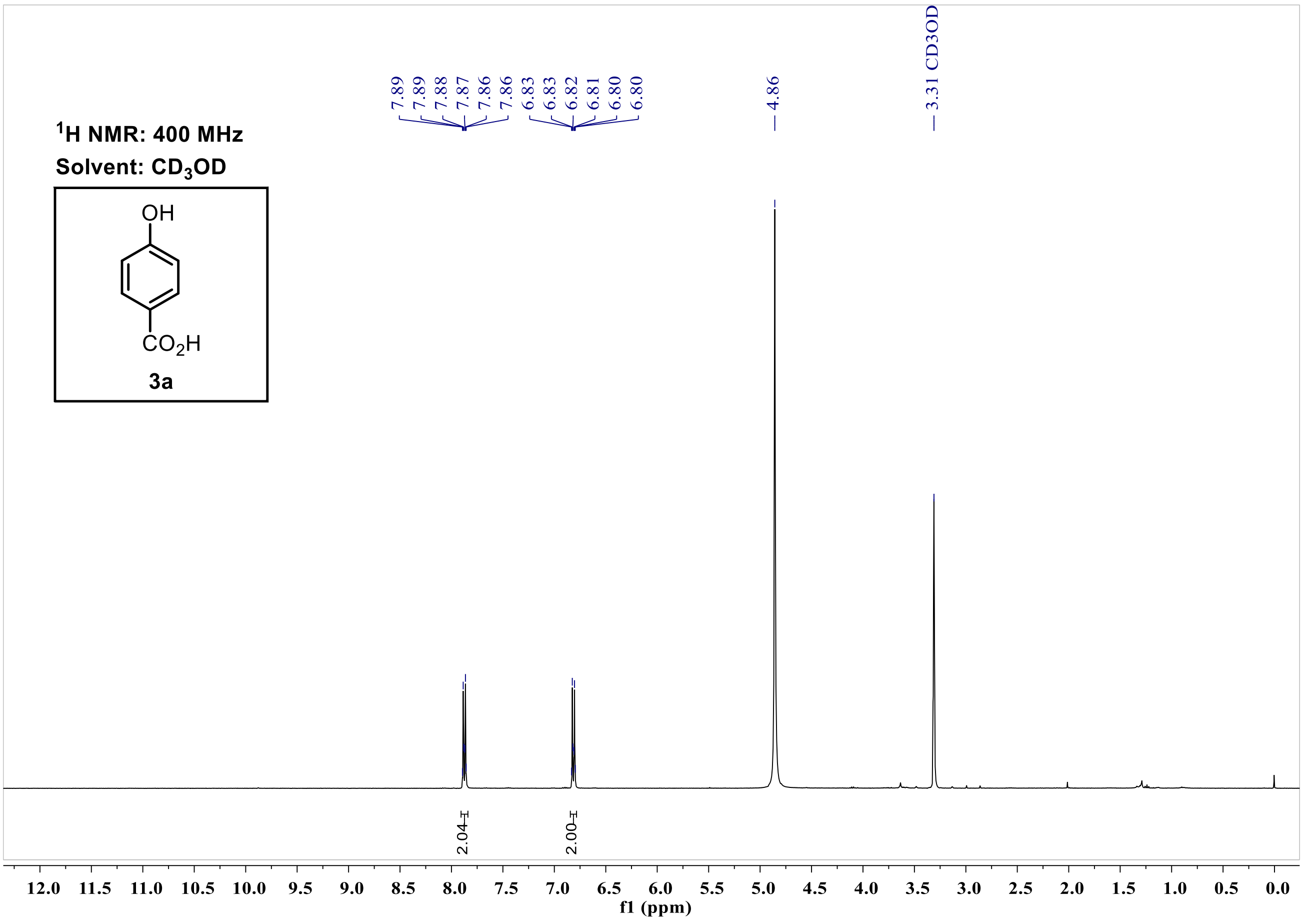
7.89
7.89
7.88
7.87
7.86
7.86
6.83
6.83
6.82
6.81
6.80
6.80
4.86
3.31 CD3OD
1H NMR: 400 MHz
Solvent: CD3OD
OH
CO2H
3a
2.04
2.00
12.0 11.5 11.0 10.5 10.0 9.5 9.0 8.5 8.0 7.5 7.0 6.5 6.0 5.5 5.0 4.5 4.0 3.5 3.0 2.5 2.0 1.5 1.0 0.5 0.0
f1 (ppm)

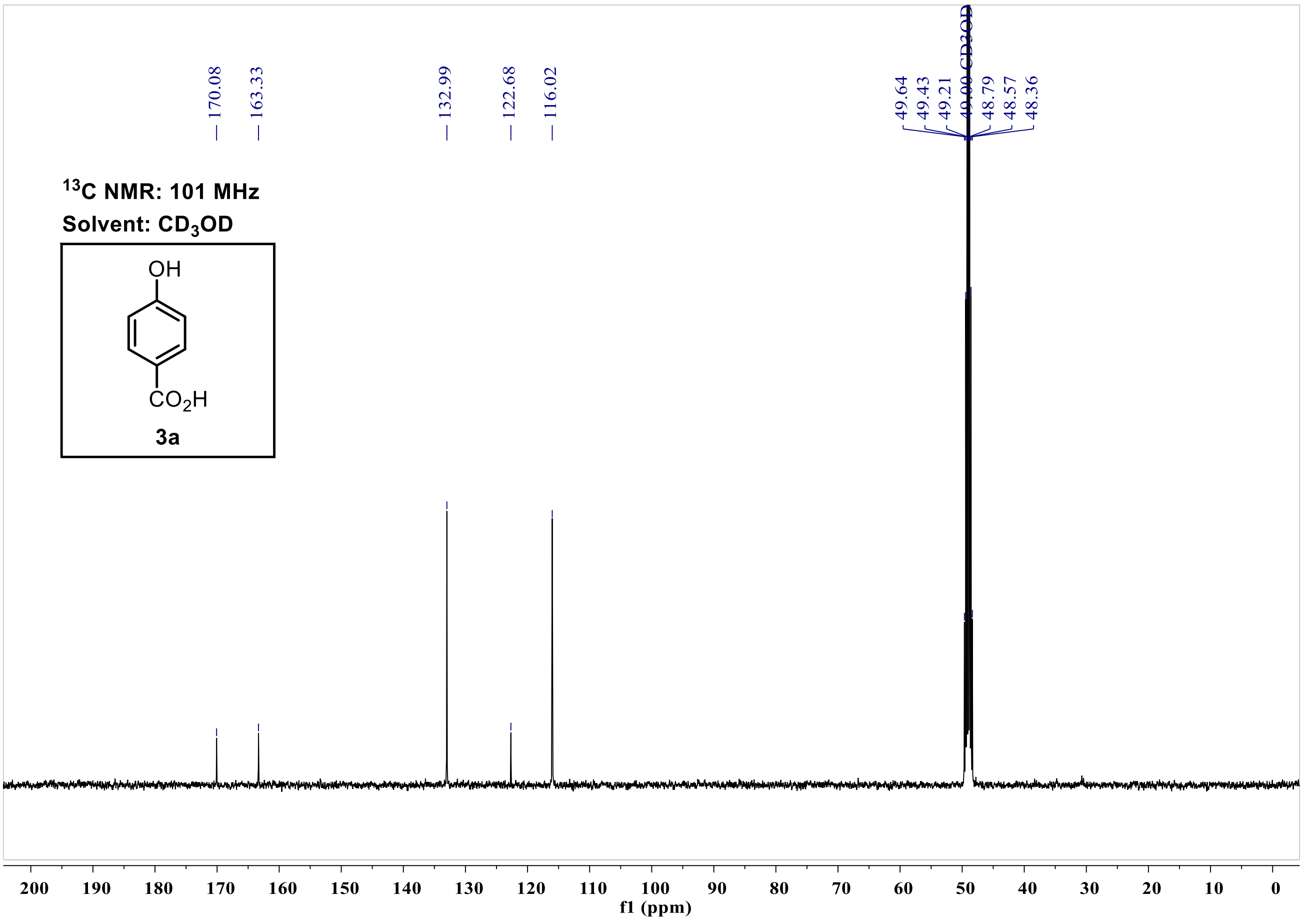
170.08
163.33
132.99
122.68
116.02
49.64
49.43
49.21
49.00 CD3OD
48.79
48.57
48.36
13C NMR: 101 MHz
Solvent: CD3OD
OH
CO2H
3a
200 190 180 170 160 150 140 130 120 110 100 90 80 70 60 50 40 30 20 10 0
f1 (ppm)

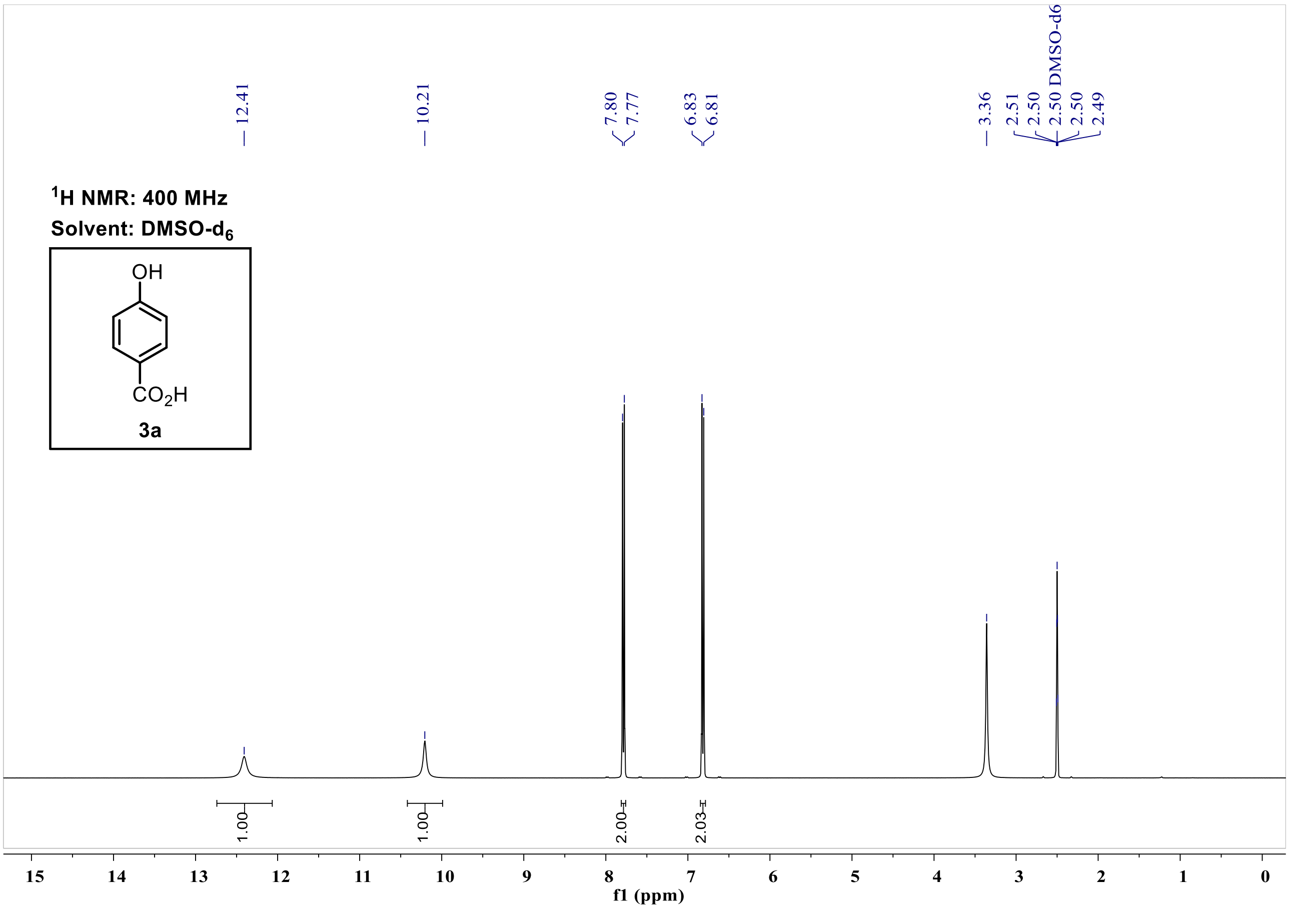
12.41
10.21
7.80
7.77
6.83
6.81
3.36
2.51
2.50
2.50 DMSO-d6
2.50
2.49
¹H NMR: 400 MHz
Solvent: DMSO-d₆
OH
CO₂H
3a
1.00
1.00
2.00
2.03
f1 (ppm)

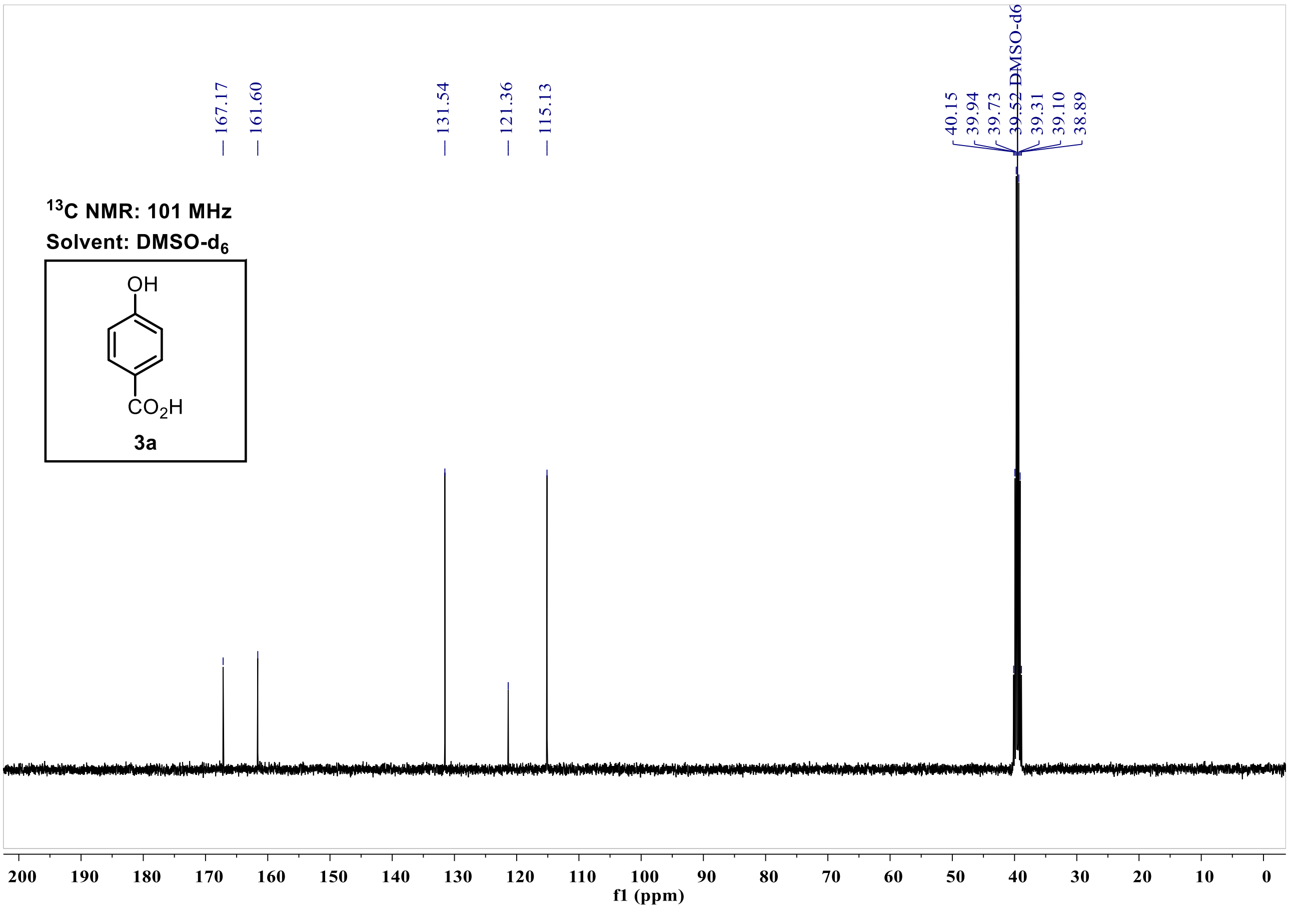
167.17
161.60
131.54
121.36
115.13
40.15
39.94
39.73
39.52 DMSO-d6
39.31
39.10
38.89
¹³C NMR: 101 MHz
Solvent: DMSO-d₆
OH
CO₂H
3a
f1 (ppm)

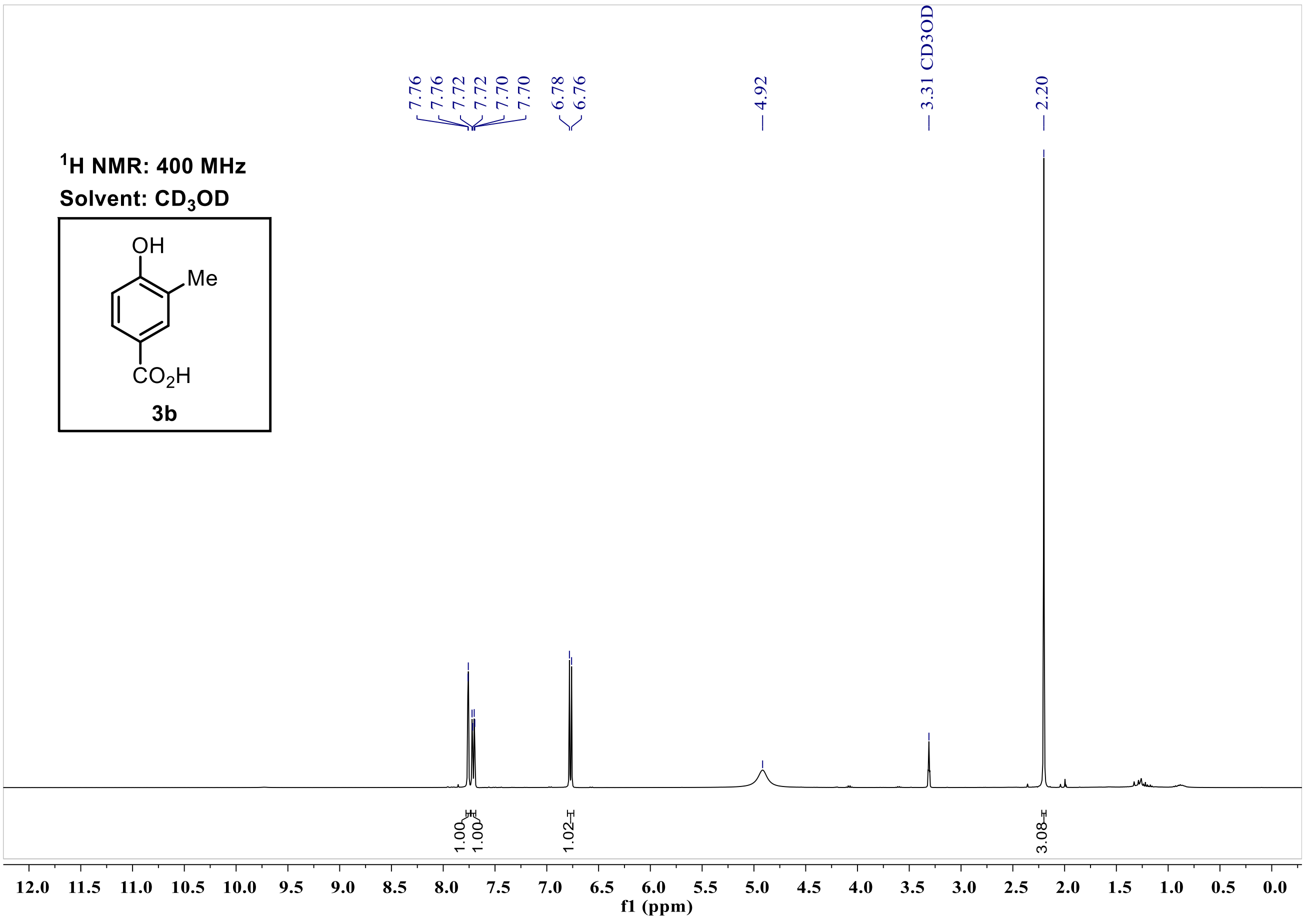
1H NMR: 400 MHz
Solvent: CD3OD
OH
Me
CO2H
3b
7.76
7.76
7.72
7.72
7.70
7.70
6.78
6.76
4.92
3.31 CD3OD
2.20
1.00
1.00
1.02
3.08
f1 (ppm)

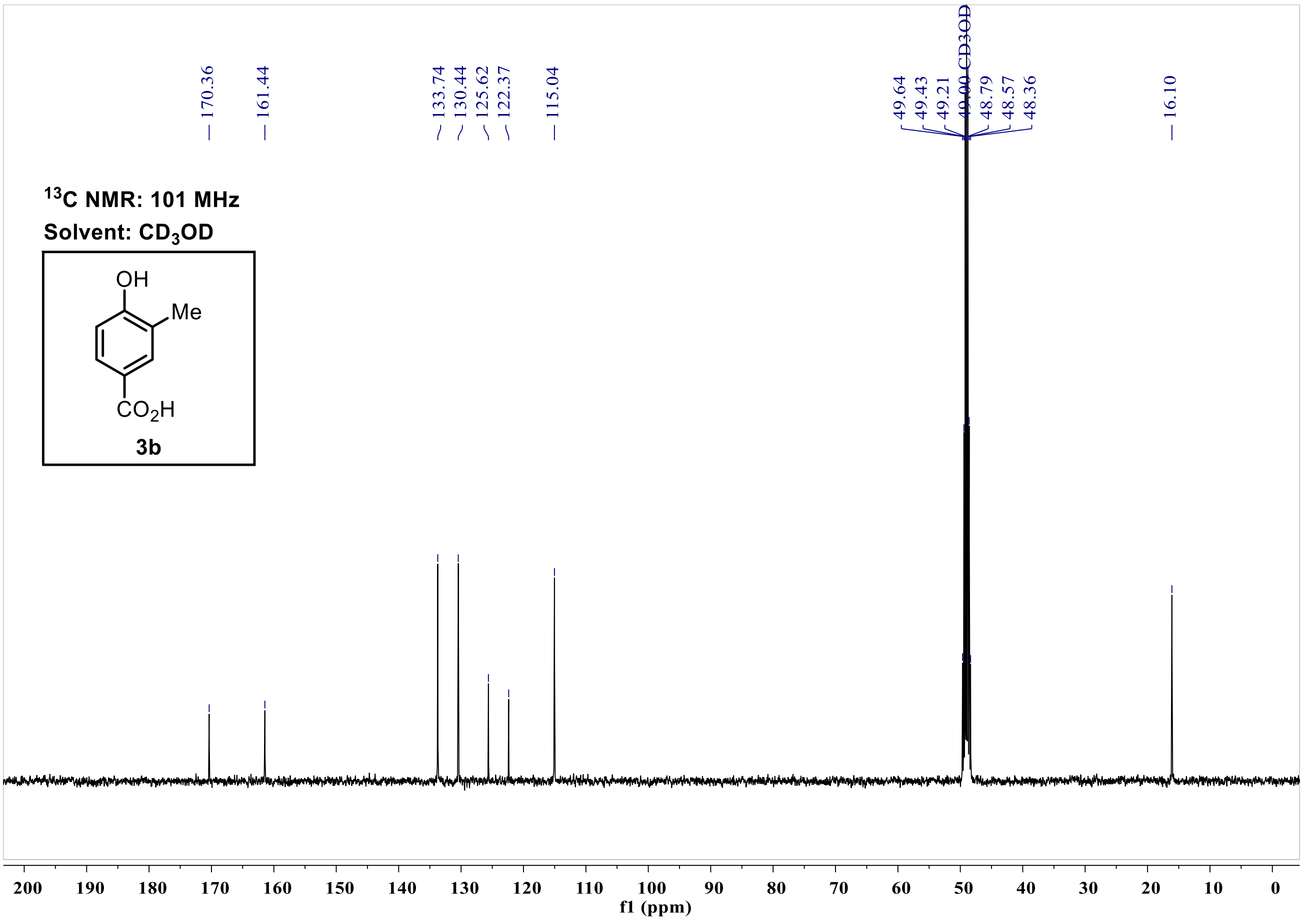
13C NMR: 101 MHz
Solvent: CD3OD
OH
Me
CO2H
3b
170.36
161.44
133.74
130.44
125.62
122.37
115.04
49.64
49.43
49.21
49.00 CD3OD
48.79
48.57
48.36
16.10
f1 (ppm)

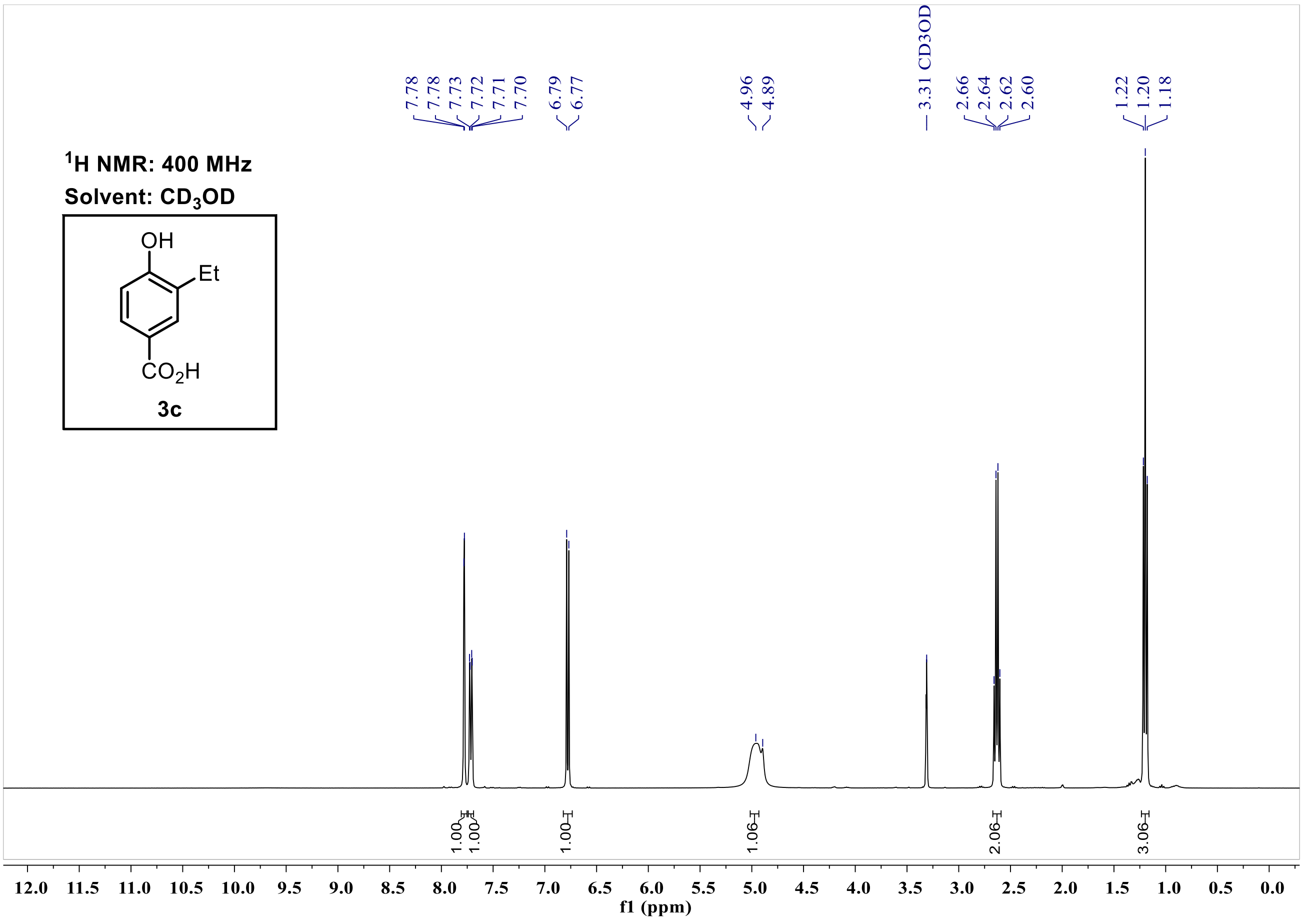

7.78
7.78
7.73
7.72
7.71
7.70
6.79
6.77
4.96
4.89
3.31 CD3OD
2.66
2.64
2.62
2.60
1.22
1.20
1.18
^{1}H NMR: 400 MHz
Solvent: CD_3OD
OH
Et
CO_2H
3c
1.00
1.00
1.00
1.06
2.06
3.06
12.0 11.5 11.0 10.5 10.0 9.5 9.0 8.5 8.0 7.5 7.0 6.5 6.0 5.5 5.0 4.5 4.0 3.5 3.0 2.5 2.0 1.5 1.0 0.5 0.0
f1 (ppm)

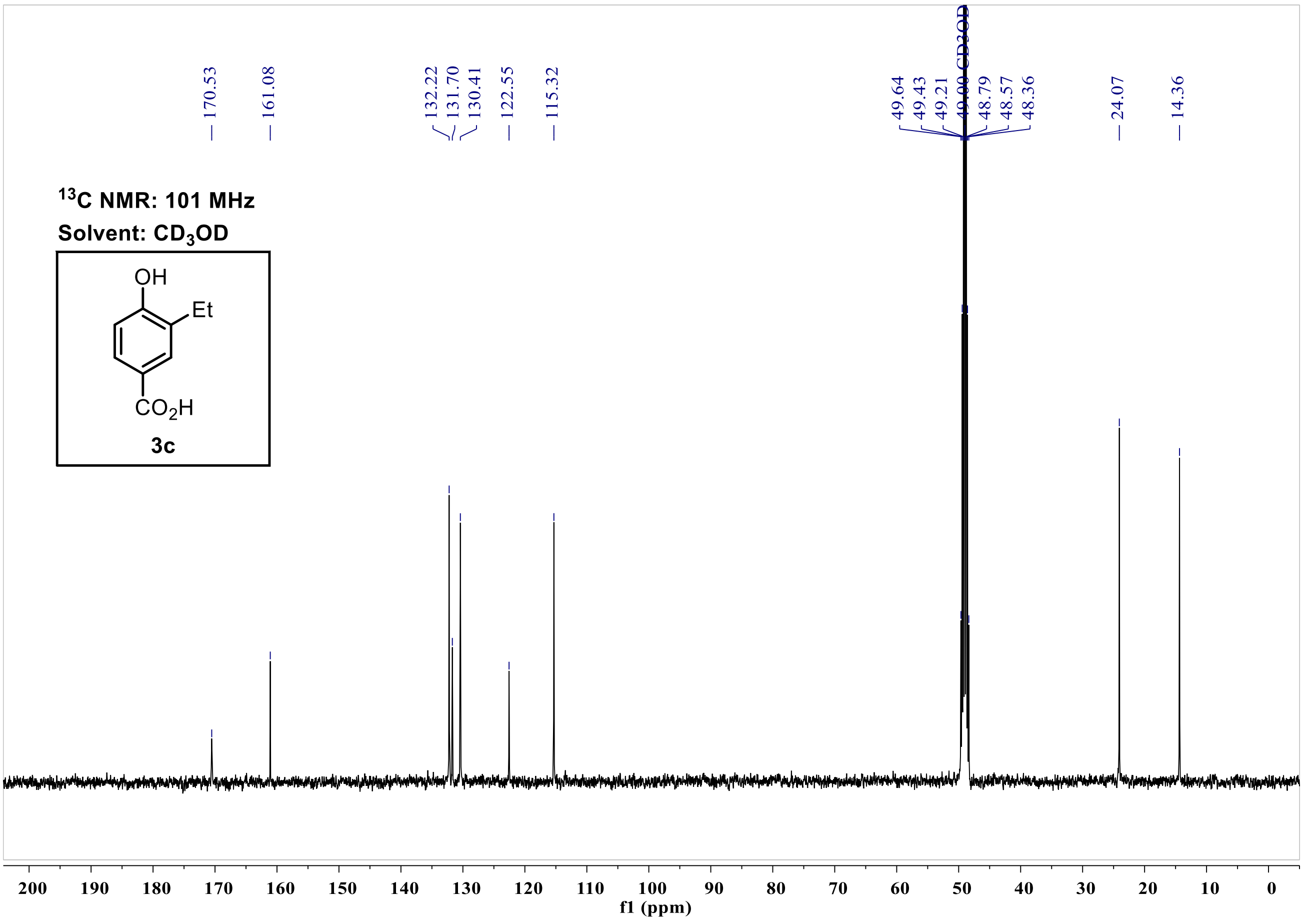

170.53
161.08
132.22
131.70
130.41
122.55
115.32
49.64
49.43
49.21
49.00 CD3OD
48.79
48.57
48.36
24.07
14.36
^{13}C NMR: 101 MHz
Solvent: CD_3OD
OH
Et
CO_2H
3c
200 190 180 170 160 150 140 130 120 110 100 90 80 70 60 50 40 30 20 10 0
f1 (ppm)

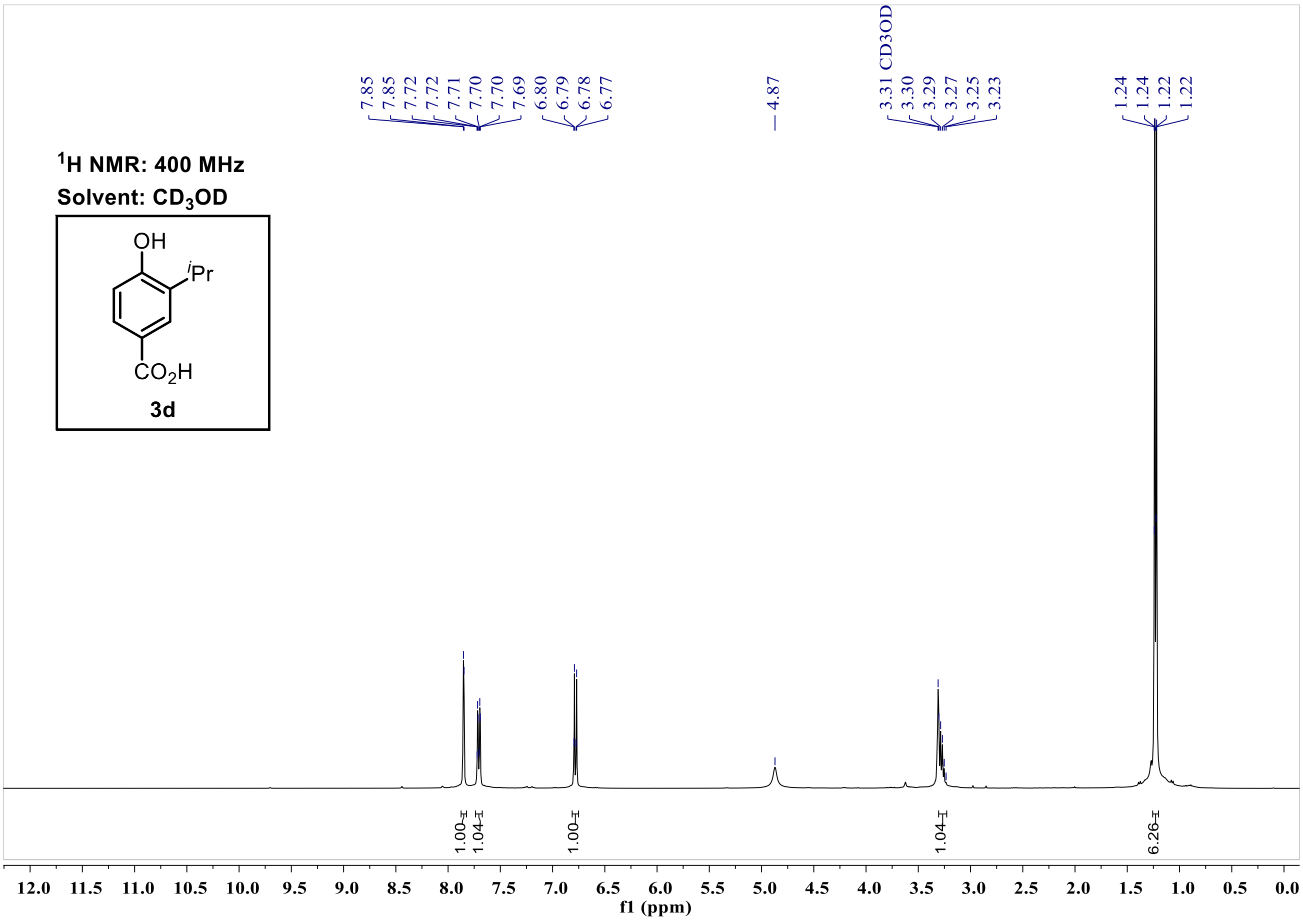

7.85
7.85
7.72
7.72
7.71
7.70
7.70
7.69
6.80
6.79
6.78
6.77
4.87
3.31 CD3OD
3.30
3.29
3.27
3.25
3.23
1.24
1.24
1.22
1.22
^{1}H NMR: 400 MHz
Solvent: CD_3OD
OH
^{i}Pr
CO_2H
3d
1.00
1.04
1.00
1.04
6.26
12.0 11.5 11.0 10.5 10.0 9.5 9.0 8.5 8.0 7.5 7.0 6.5 6.0 5.5 5.0 4.5 4.0 3.5 3.0 2.5 2.0 1.5 1.0 0.5 0.0
f1 (ppm)

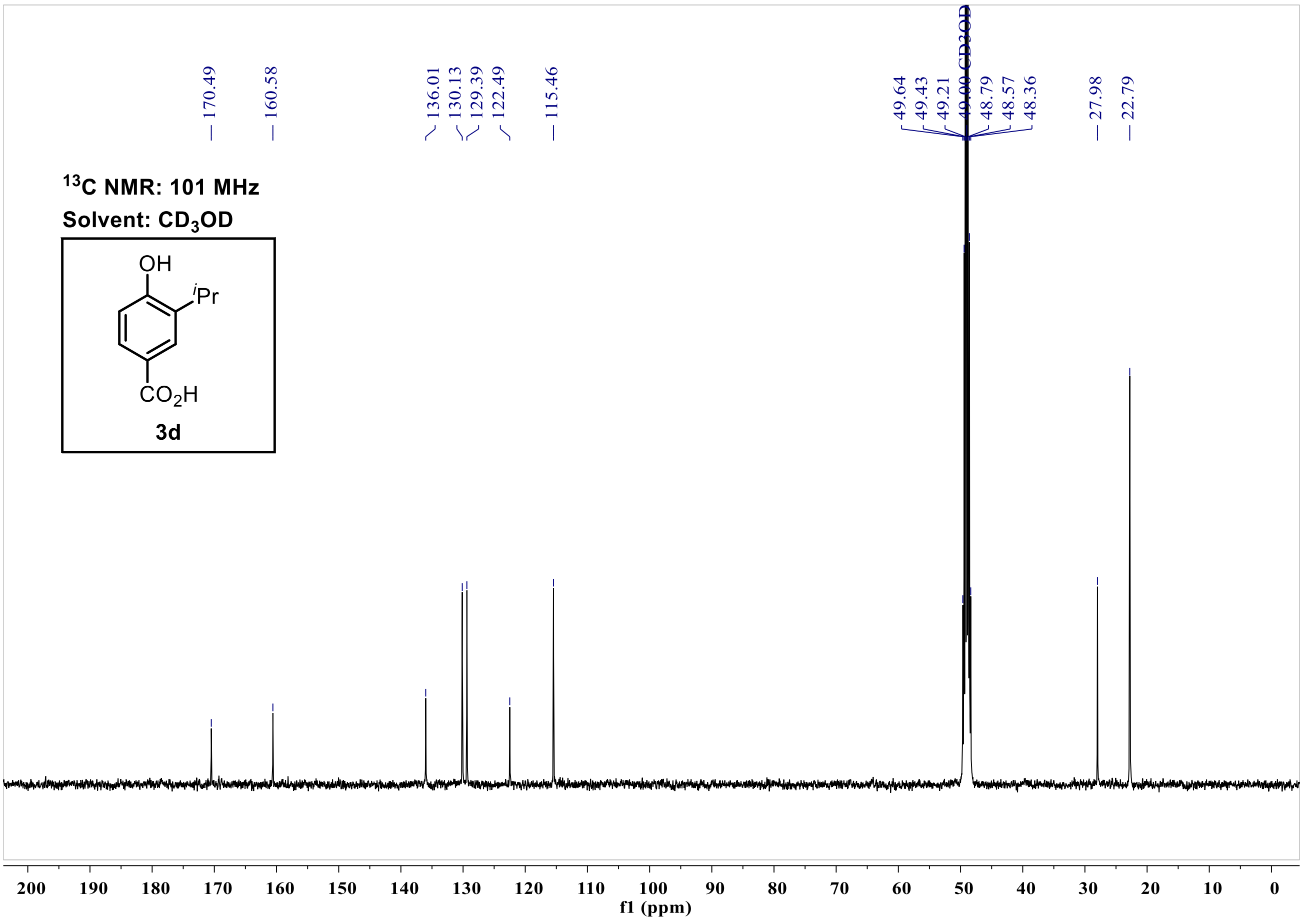

170.49
160.58
136.01
130.13
129.39
122.49
115.46
49.64
49.43
49.21
49.00 CD3OD
48.79
48.57
48.36
27.98
22.79
^{13}C NMR: 101 MHz
Solvent: CD_3OD
OH
^{i}Pr
CO_2H
3d
200 190 180 170 160 150 140 130 120 110 100 90 80 70 60 50 40 30 20 10 0
f1 (ppm)

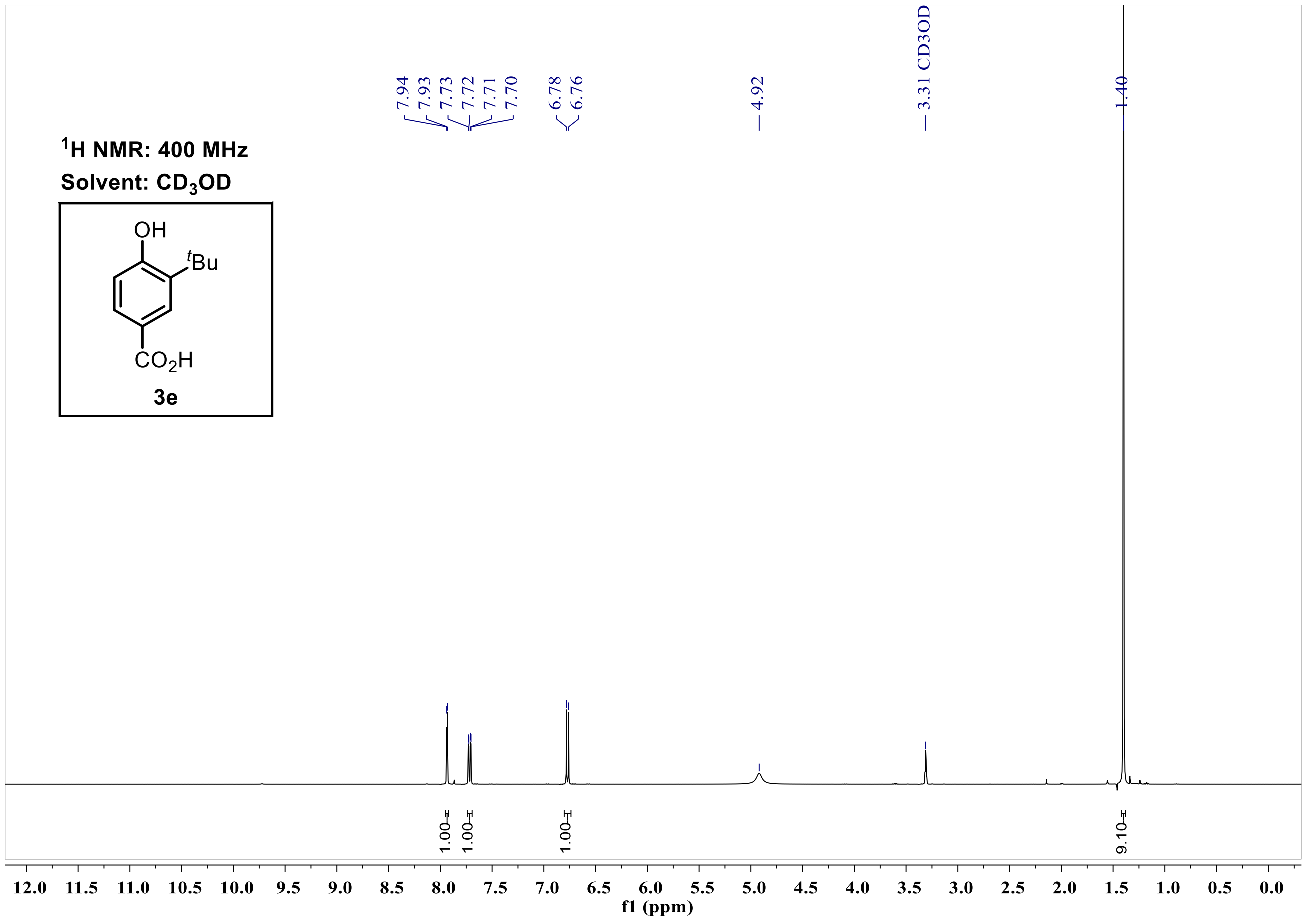

7.94
7.93
7.73
7.72
7.71
7.70
6.78
6.76
4.92
3.31 CD3OD
1.40
1H NMR: 400 MHz
Solvent: CD3OD
OH
tBu
CO2H
3e
1.00
1.00
1.00
9.10
12.0 11.5 11.0 10.5 10.0 9.5 9.0 8.5 8.0 7.5 7.0 6.5 6.0 5.5 5.0 4.5 4.0 3.5 3.0 2.5 2.0 1.5 1.0 0.5 0.0
f1 (ppm)

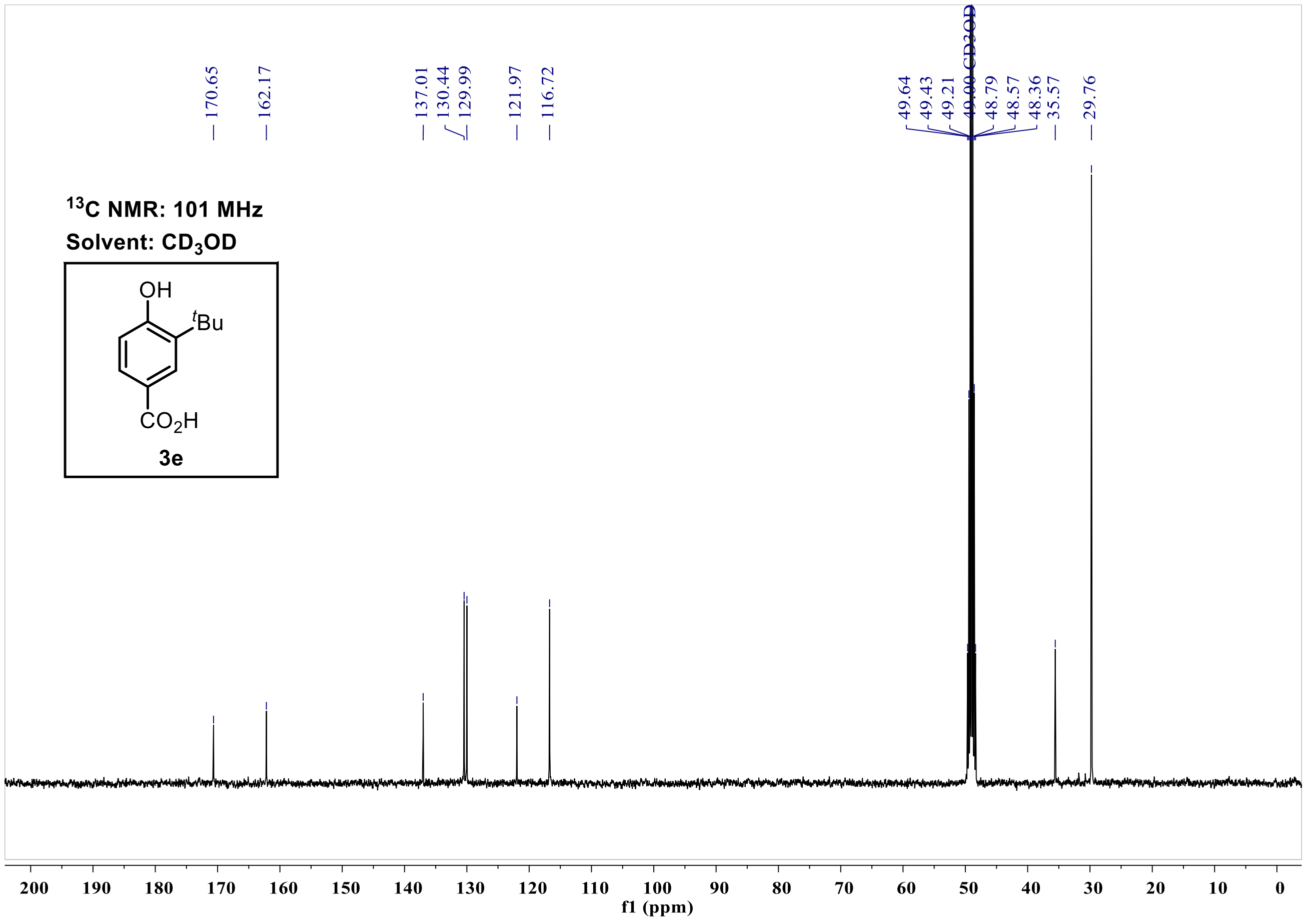

170.65
162.17
137.01
130.44
129.99
121.97
116.72
49.64
49.43
49.21
49.00 CD3OD
48.79
48.57
48.36
35.57
29.76
13C NMR: 101 MHz
Solvent: CD3OD
OH
tBu
CO2H
3e
200 190 180 170 160 150 140 130 120 110 100 90 80 70 60 50 40 30 20 10 0
f1 (ppm)

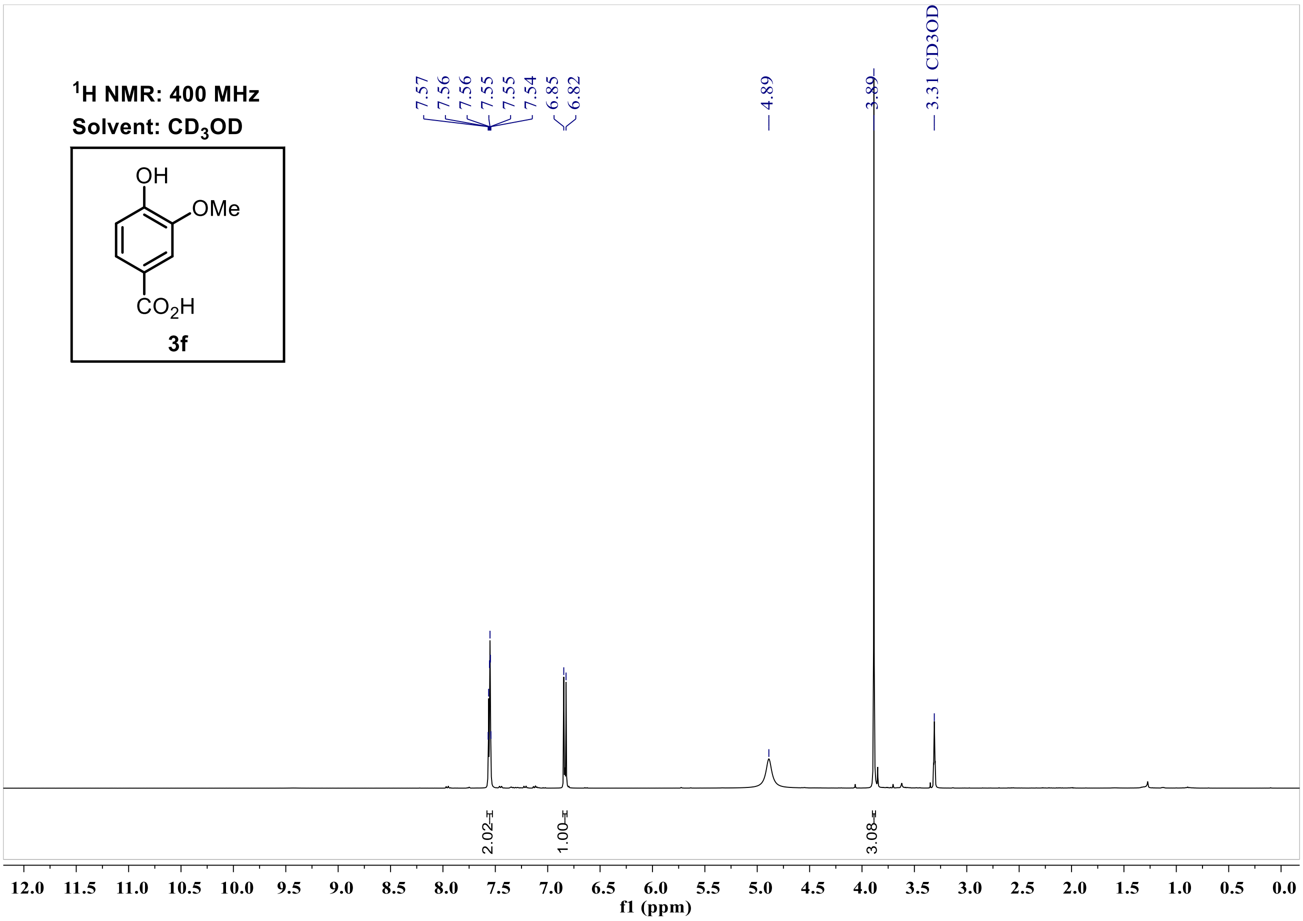

1H NMR: 400 MHz
Solvent: CD3OD
OH
OMe
CO2H
3f
7.57
7.56
7.56
7.55
7.55
7.54
6.85
6.82
4.89
3.89
3.31 CD3OD
2.02
1.00
3.08
12.0 11.5 11.0 10.5 10.0 9.5 9.0 8.5 8.0 7.5 7.0 6.5 6.0 5.5 5.0 4.5 4.0 3.5 3.0 2.5 2.0 1.5 1.0 0.5 0.0
f1 (ppm)

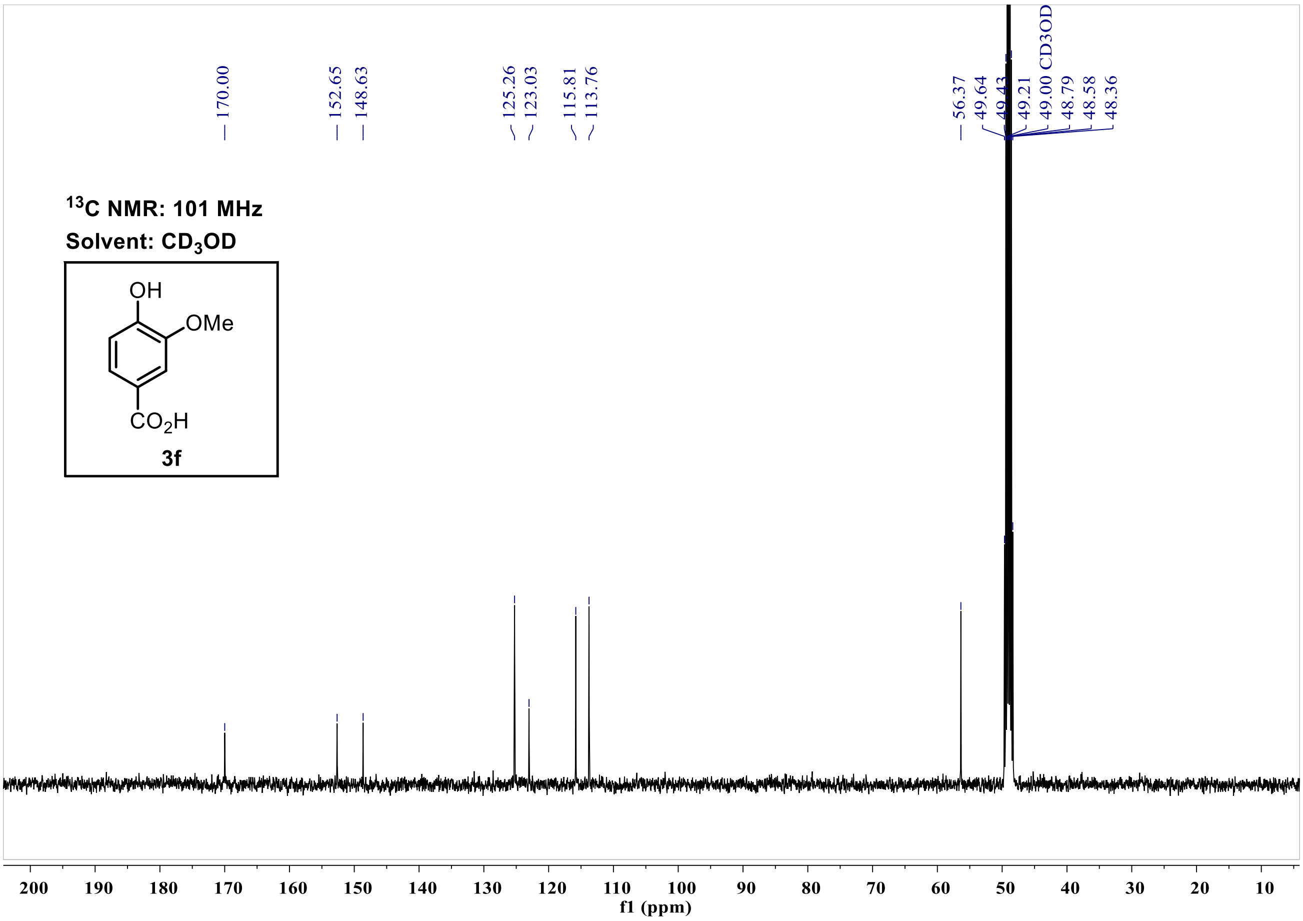

13C NMR: 101 MHz
Solvent: CD3OD
OH
OMe
CO2H
3f
170.00
152.65
148.63
125.26
123.03
115.81
113.76
56.37
49.64
49.43
49.21
49.00 CD3OD
48.79
48.58
48.36
200 190 180 170 160 150 140 130 120 110 100 90 80 70 60 50 40 30 20 10
f1 (ppm)

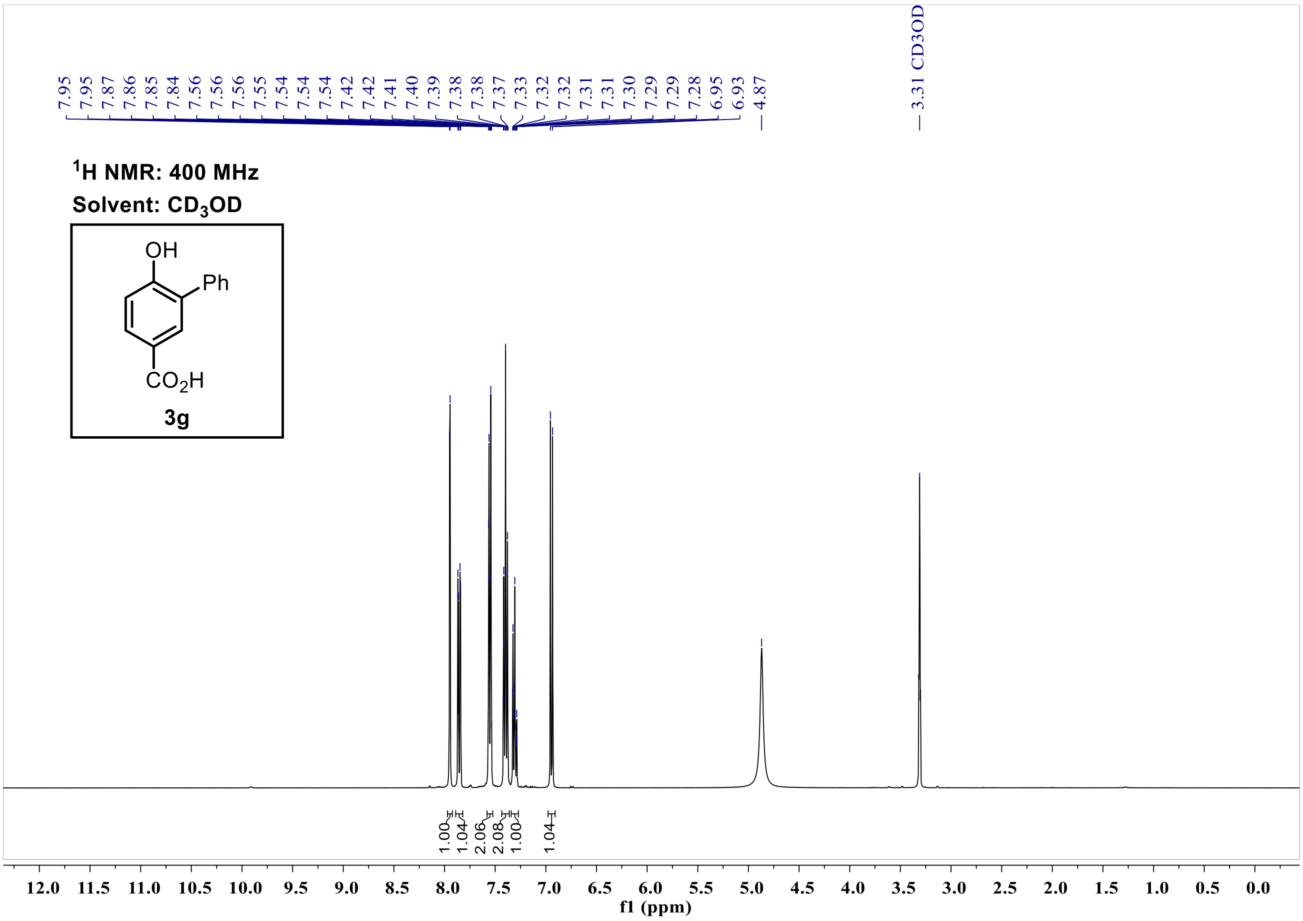

7.95
7.95
7.87
7.86
7.85
7.84
7.56
7.56
7.56
7.55
7.54
7.54
7.54
7.42
7.42
7.41
7.40
7.39
7.38
7.38
7.37
7.33
7.32
7.32
7.31
7.31
7.30
7.29
7.29
7.28
6.95
6.93
4.87
3.31 CD3OD
1H NMR: 400 MHz
Solvent: CD3OD
OH
Ph
CO2H
3g
1.00
1.04
2.06
2.08
1.00
1.04
12.0 11.5 11.0 10.5 10.0 9.5 9.0 8.5 8.0 7.5 7.0 6.5 6.0 5.5 5.0 4.5 4.0 3.5 3.0 2.5 2.0 1.5 1.0 0.5 0.0
f1 (ppm)

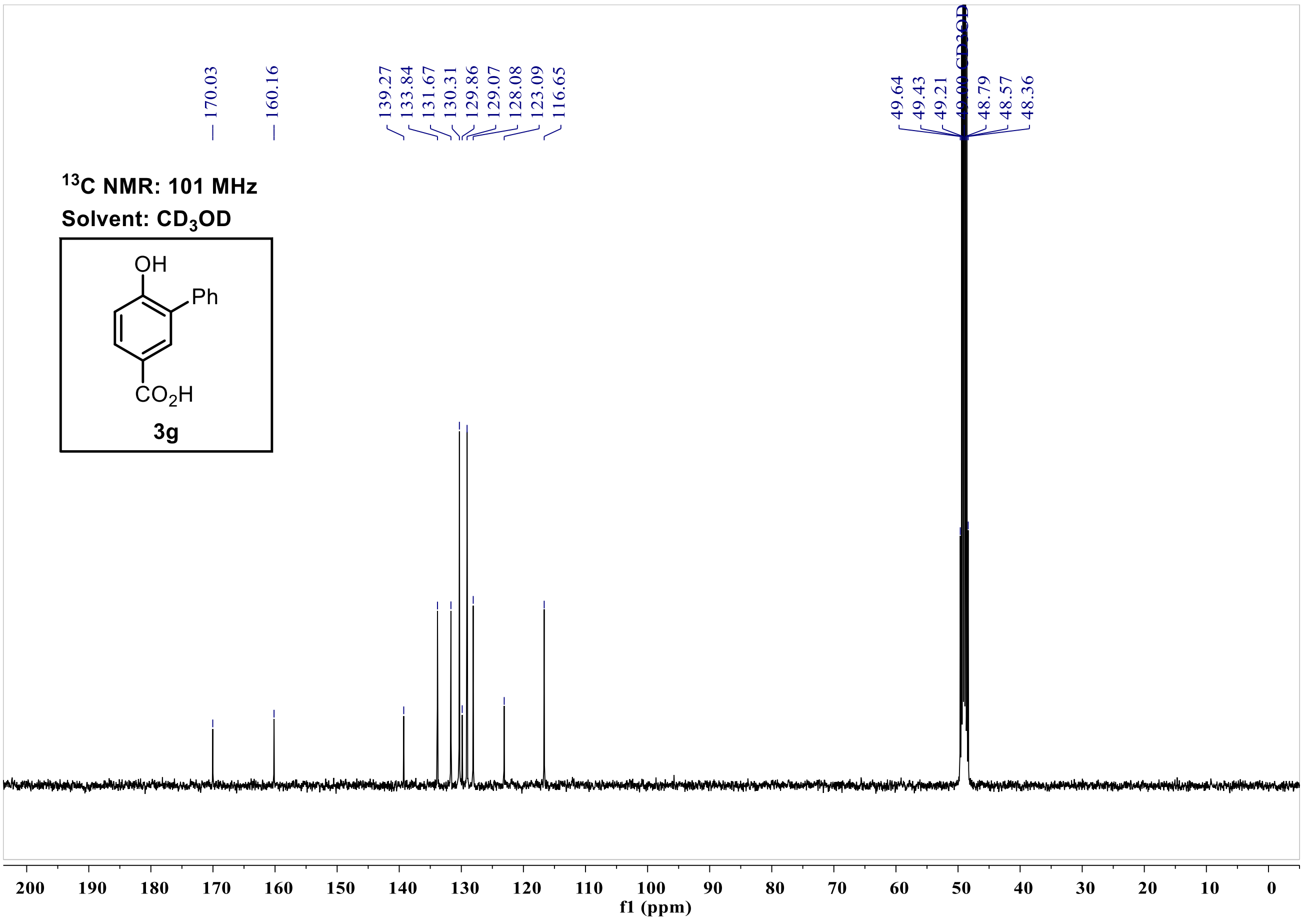

170.03
160.16
139.27
133.84
131.67
130.31
129.86
129.07
128.08
123.09
116.65
49.64
49.43
49.21
49.00 CD3OD
48.79
48.57
48.36
13C NMR: 101 MHz
Solvent: CD3OD
OH
Ph
CO2H
3g
200 190 180 170 160 150 140 130 120 110 100 90 80 70 60 50 40 30 20 10 0
f1 (ppm)

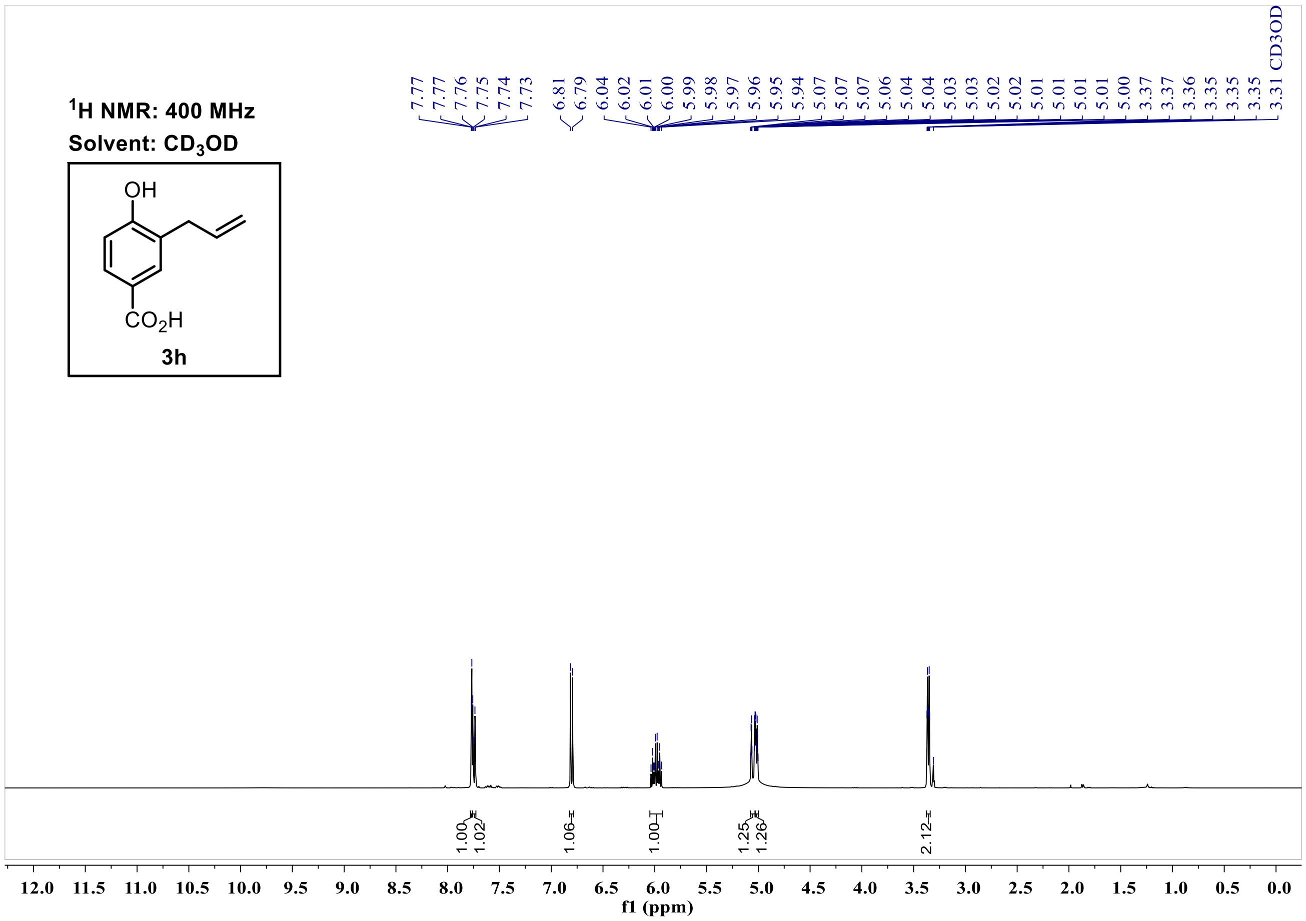
1H NMR: 400 MHz
Solvent: CD3OD
OH
CO2H
3h
7.77 7.77 7.76 7.75 7.74 7.73 6.81 6.79 6.04 6.02 6.01 6.00 5.99 5.98 5.97 5.96 5.95 5.94 5.07 5.07 5.06 5.04 5.04 5.03 5.03 5.02 5.02 5.01 5.01 5.01 5.00 3.37 3.37 3.36 3.35 3.35 3.35 3.31 CD3OD
1.00 1.02 1.06 1.00 1.25 1.26 2.12
12.0 11.5 11.0 10.5 10.0 9.5 9.0 8.5 8.0 7.5 7.0 6.5 6.0 5.5 5.0 4.5 4.0 3.5 3.0 2.5 2.0 1.5 1.0 0.5 0.0
f1 (ppm)

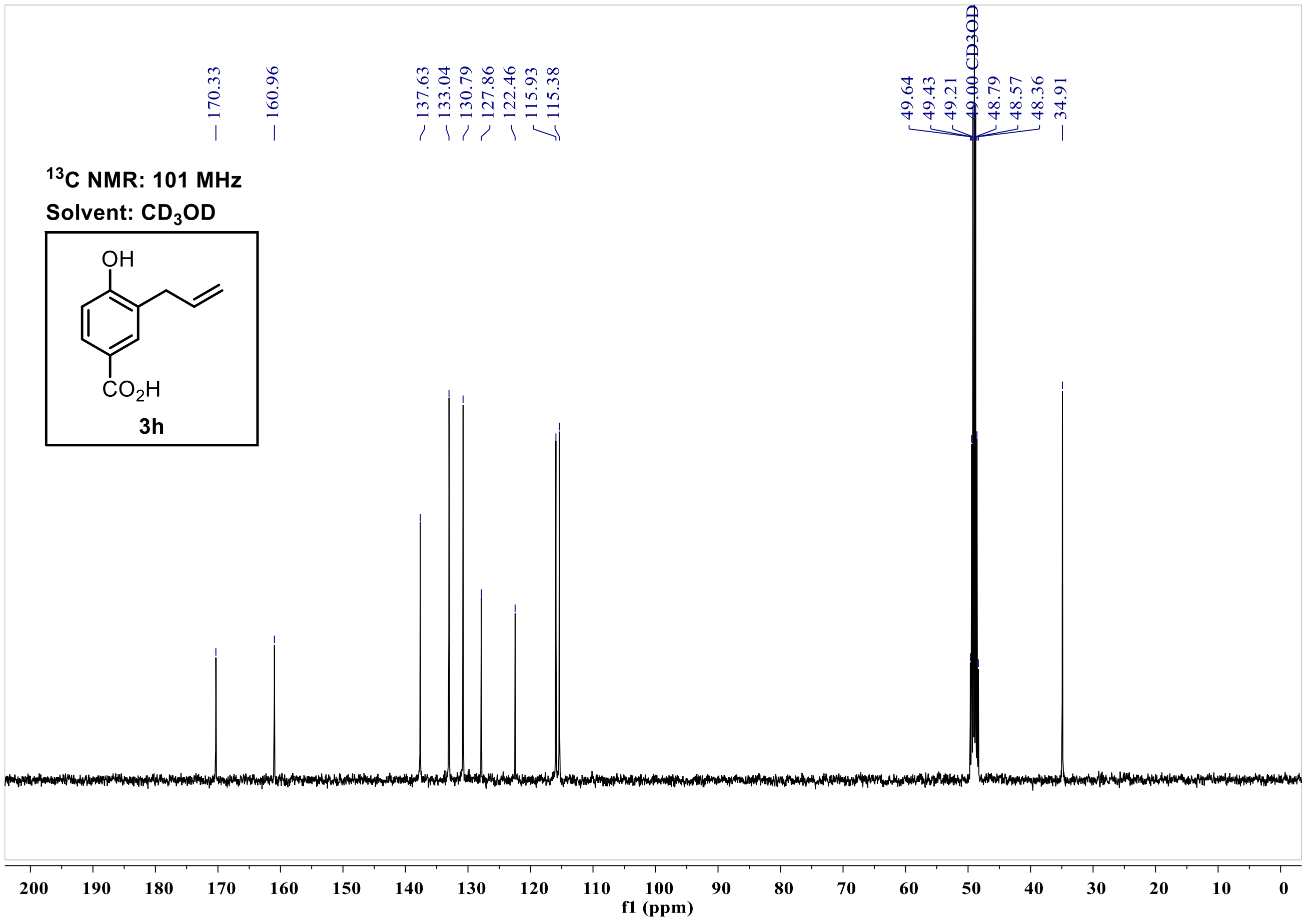
13C NMR: 101 MHz
Solvent: CD3OD
OH
CO2H
3h
170.33 160.96 137.63 133.04 130.79 127.86 122.46 115.93 115.38
49.64 49.43 49.21 49.00 CD3OD 48.79 48.57 48.36 34.91
200 190 180 170 160 150 140 130 120 110 100 90 80 70 60 50 40 30 20 10 0
f1 (ppm)

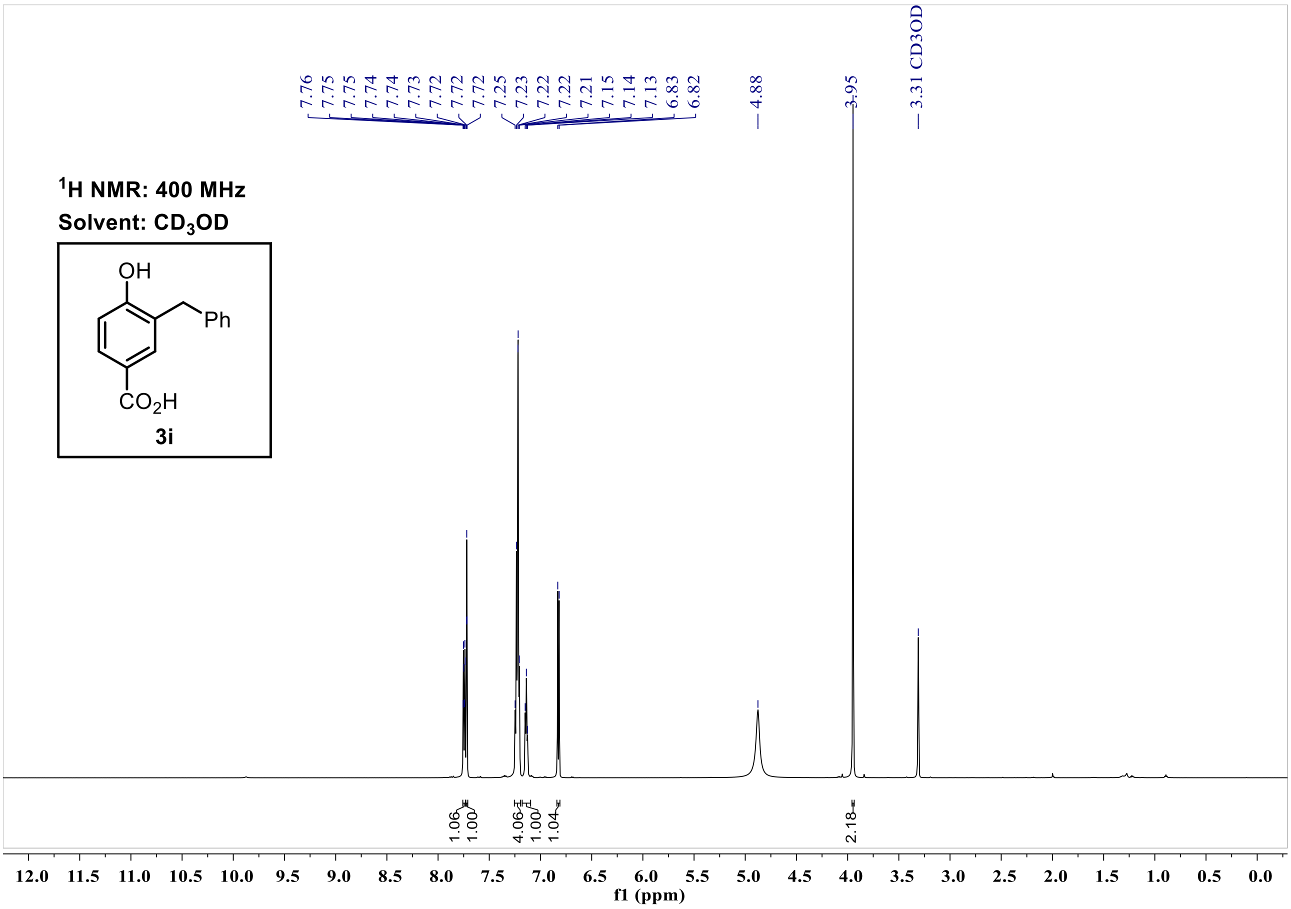
7.76
7.75
7.75
7.74
7.74
7.73
7.72
7.72
7.72
7.25
7.23
7.22
7.22
7.21
7.15
7.14
7.13
6.83
6.82
4.88
3.95
3.31 CD3OD
1H NMR: 400 MHz
Solvent: CD3OD
OH
Ph
CO2H
3i
1.06
1.00
4.06
1.00
1.04
2.18
12.0
11.5
11.0
10.5
10.0
9.5
9.0
8.5
8.0
7.5
7.0
6.5
6.0
5.5
5.0
4.5
4.0
3.5
3.0
2.5
2.0
1.5
1.0
0.5
0.0
f1 (ppm)

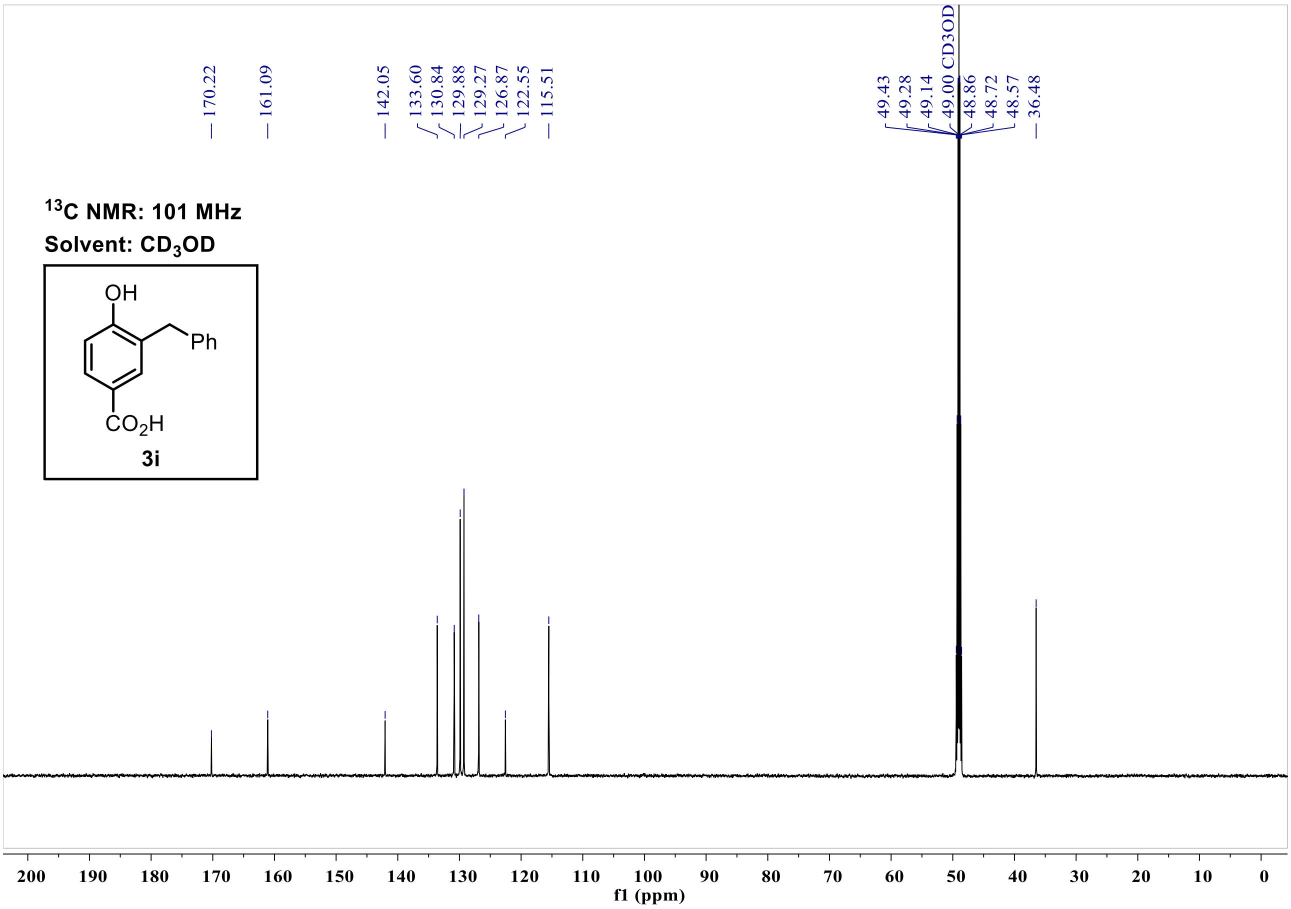
170.22
161.09
142.05
133.60
130.84
129.88
129.27
126.87
122.55
115.51
49.43
49.28
49.14
49.00 CD3OD
48.86
48.72
48.57
36.48
13C NMR: 101 MHz
Solvent: CD3OD
OH
Ph
CO2H
3i
200
190
180
170
160
150
140
130
120
110
100
90
80
70
60
50
40
30
20
10
0
f1 (ppm)

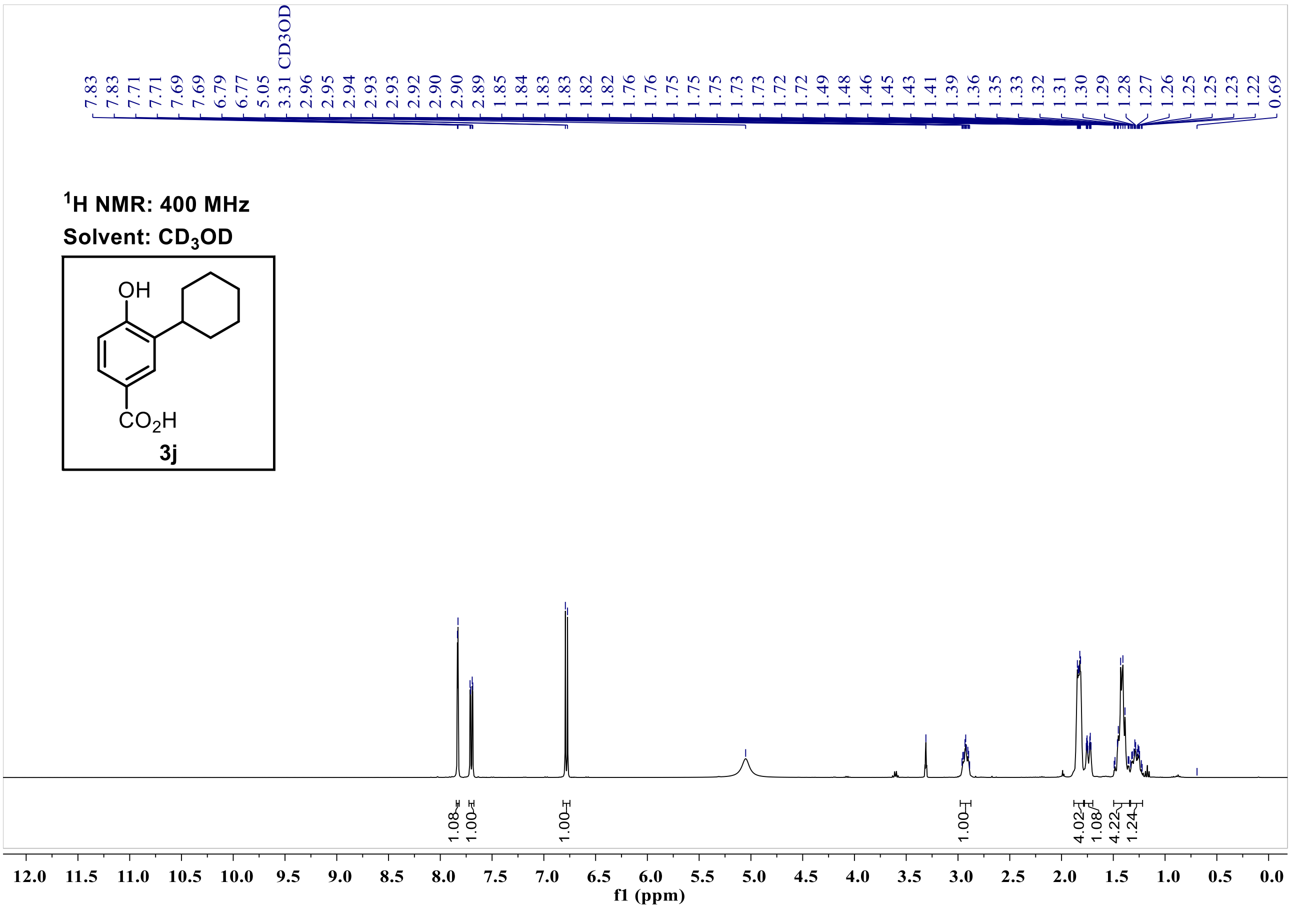

1H NMR: 400 MHz
Solvent: CD_3OD
OH
CO_2H
3j
f1 (ppm)

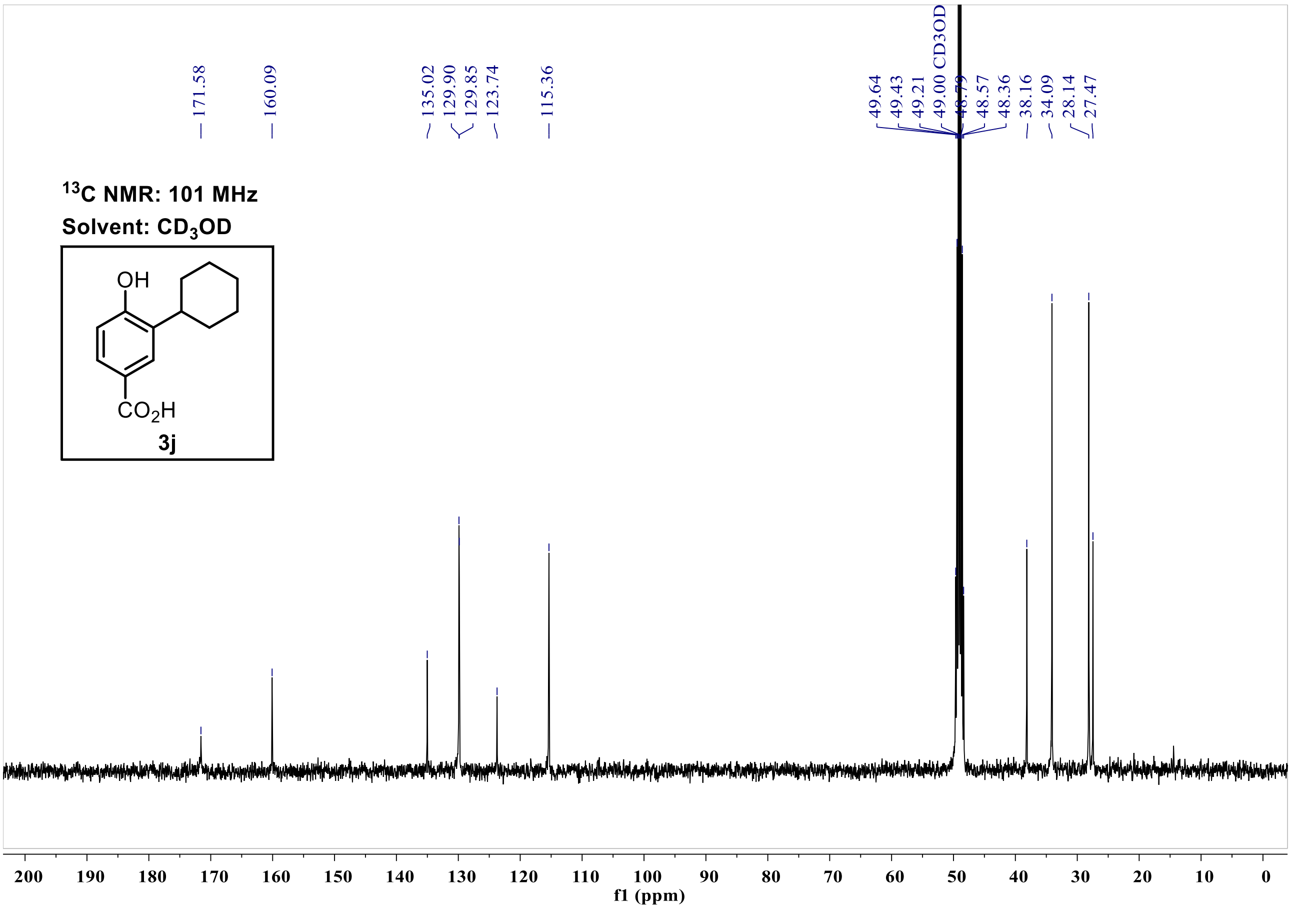

13C NMR: 101 MHz
Solvent: CD_3OD
OH
CO_2H
3j
f1 (ppm)

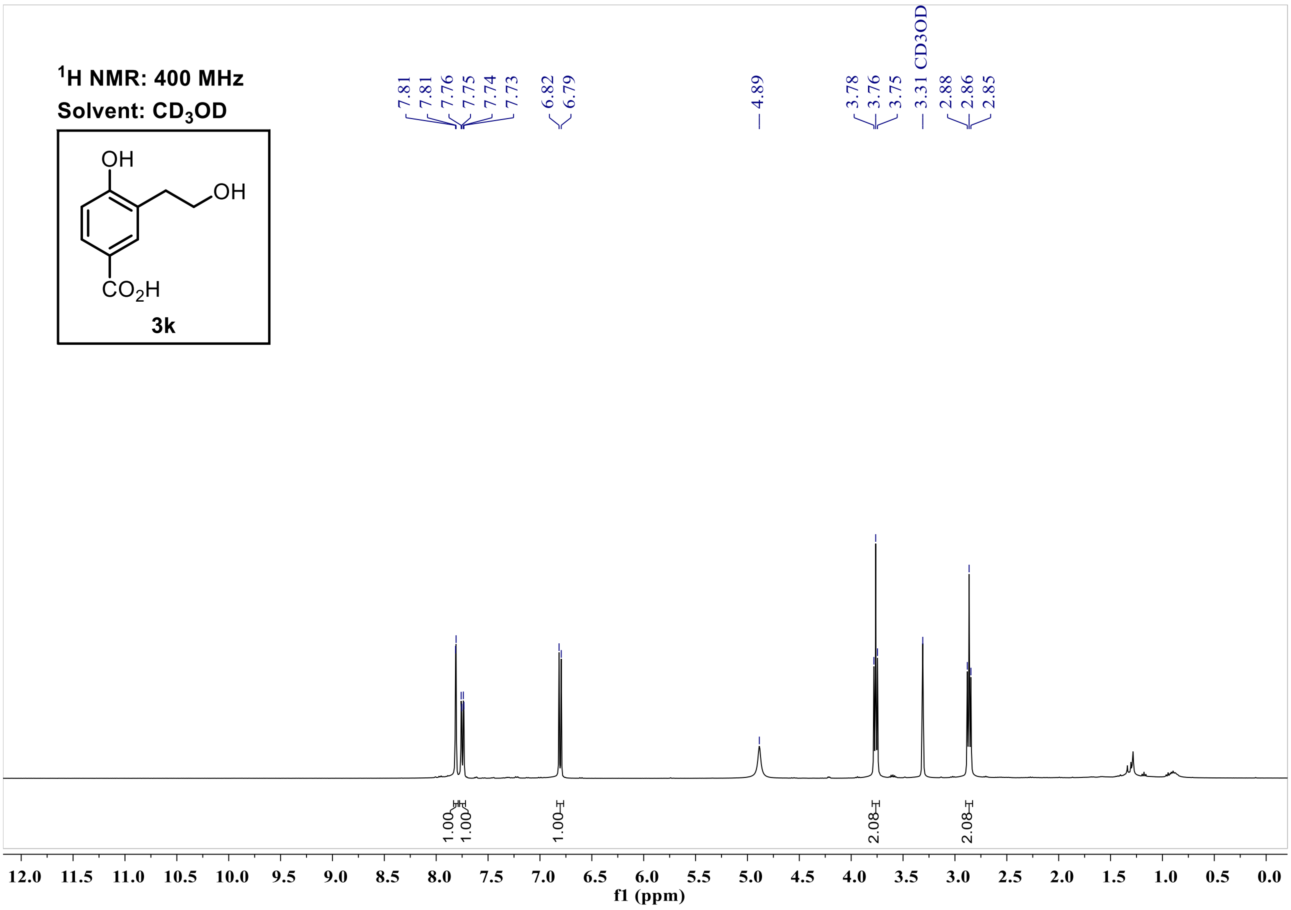

^{1}H NMR: 400 MHz
Solvent: CD_3OD
3k
7.81
7.81
7.76
7.75
7.74
7.73
6.82
6.79
4.89
3.78
3.76
3.75
3.31 CD3OD
2.88
2.86
2.85
1.00
1.00
1.00
2.08
2.08
f1 (ppm)

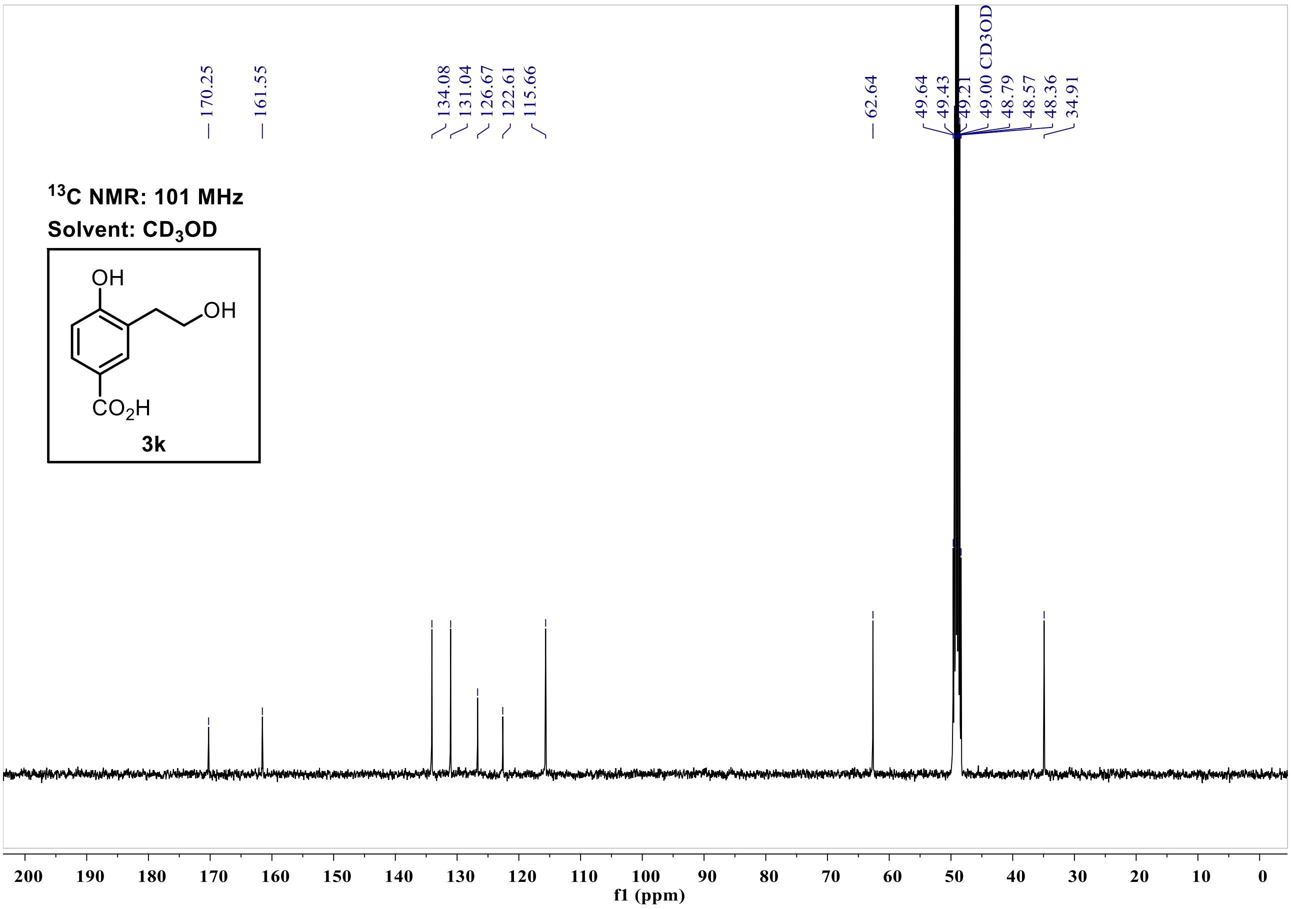

^{13}C NMR: 101 MHz
Solvent: CD_3OD
3k
170.25
161.55
134.08
131.04
126.67
122.61
115.66
62.64
49.64
49.43
49.21
49.00 CD3OD
48.79
48.57
48.36
34.91
f1 (ppm)

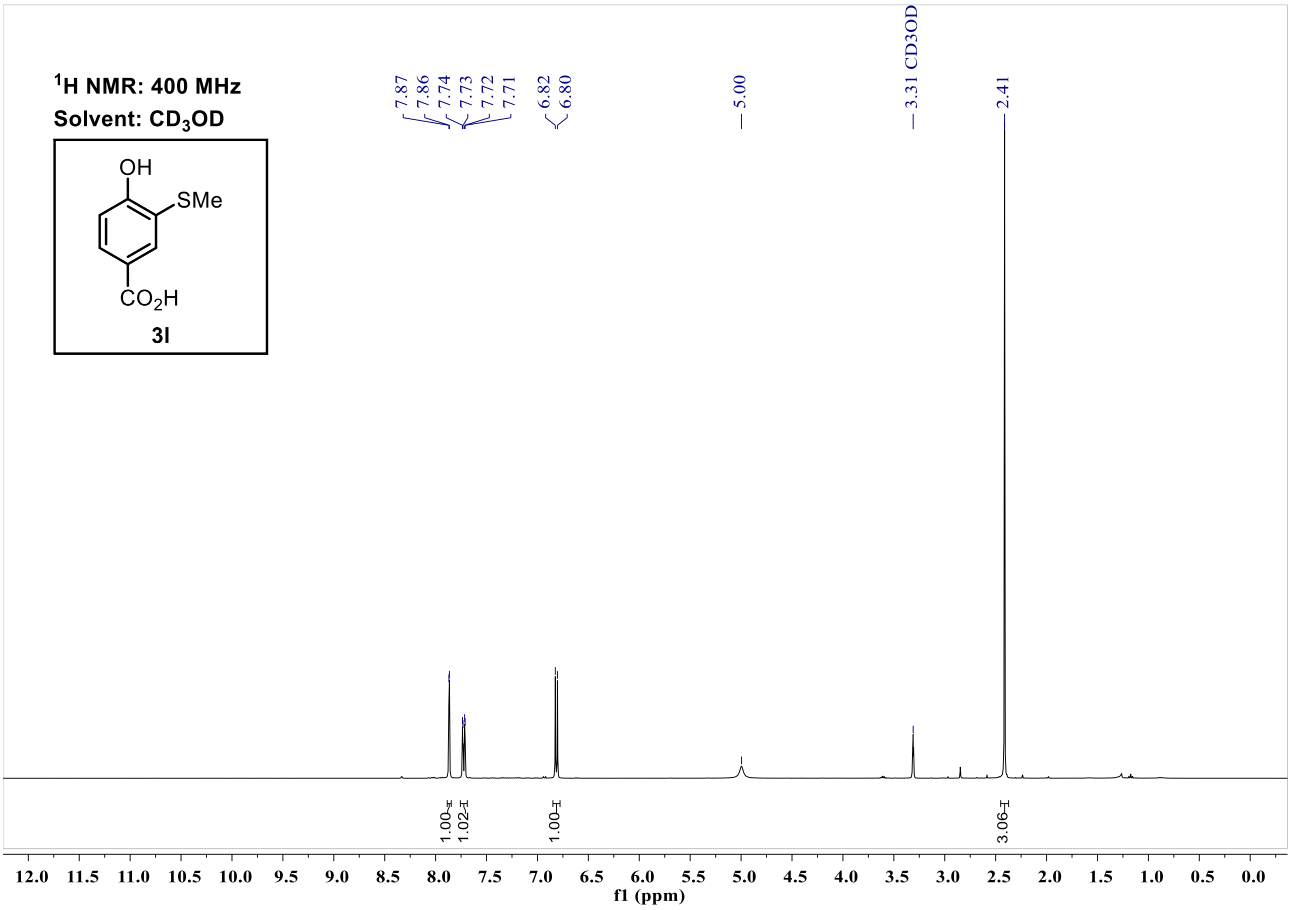

1H NMR: 400 MHz
Solvent: CD3OD
OH
SMe
CO2H
3l
7.87
7.86
7.74
7.73
7.72
7.71
6.82
6.80
5.00
3.31 CD3OD
2.41
1.00
1.02
1.00
3.06
12.0 11.5 11.0 10.5 10.0 9.5 9.0 8.5 8.0 7.5 7.0 6.5 6.0 5.5 5.0 4.5 4.0 3.5 3.0 2.5 2.0 1.5 1.0 0.5 0.0
f1 (ppm)

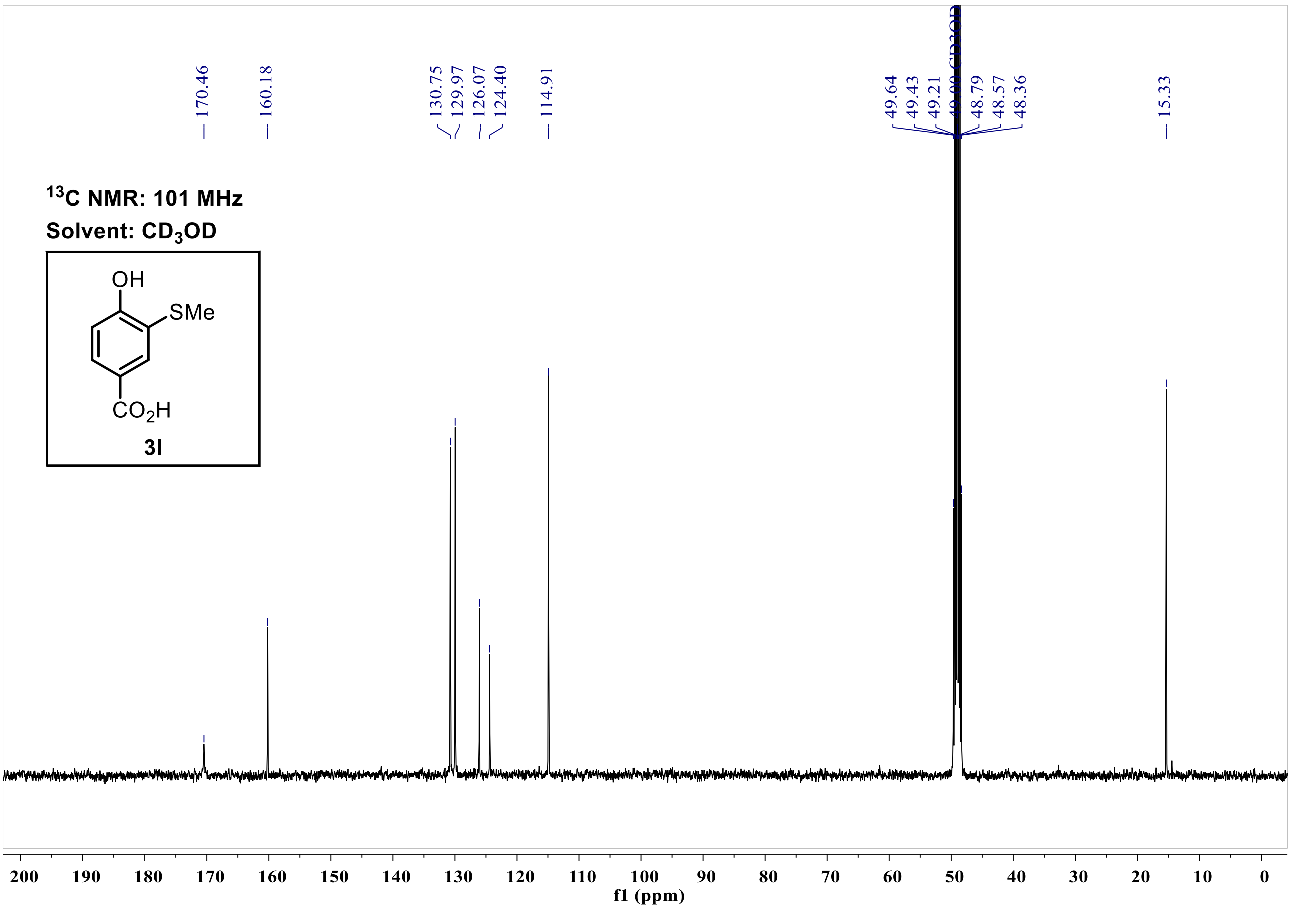

170.46
160.18
130.75
129.97
126.07
124.40
114.91
49.64
49.43
49.21
48.79
48.57
48.36
15.33
13C NMR: 101 MHz
Solvent: CD3OD
OH
SMe
CO2H
3l
200 190 180 170 160 150 140 130 120 110 100 90 80 70 60 50 40 30 20 10 0
f1 (ppm)

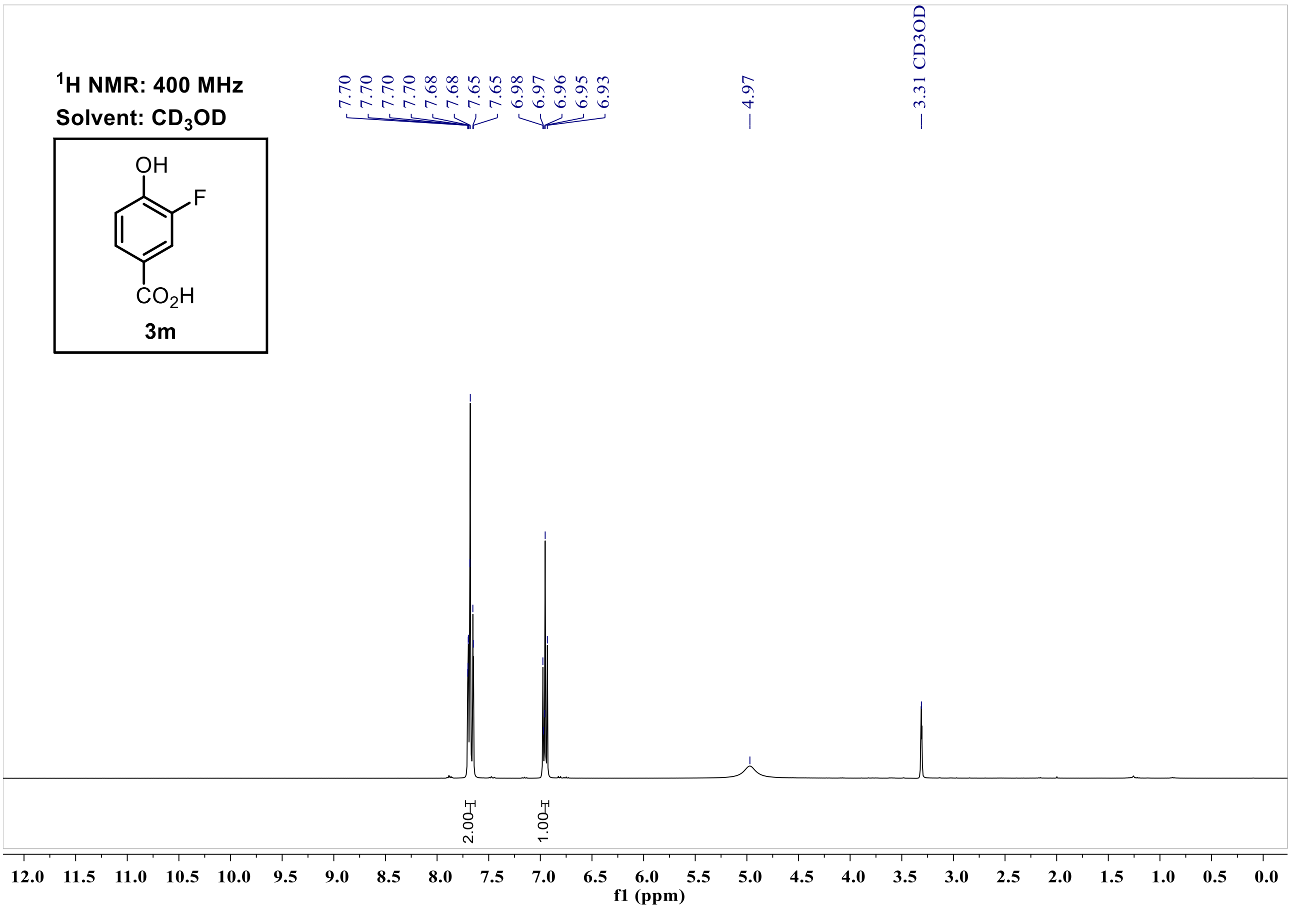
1H NMR: 400 MHz
Solvent: CD3OD
OH
F
CO2H
3m
7.70
7.70
7.70
7.70
7.68
7.68
7.65
7.65
6.98
6.97
6.96
6.95
6.93
4.97
3.31 CD3OD
2.00
1.00
12.0 11.5 11.0 10.5 10.0 9.5 9.0 8.5 8.0 7.5 7.0 6.5 6.0 5.5 5.0 4.5 4.0 3.5 3.0 2.5 2.0 1.5 1.0 0.5 0.0
f1 (ppm)

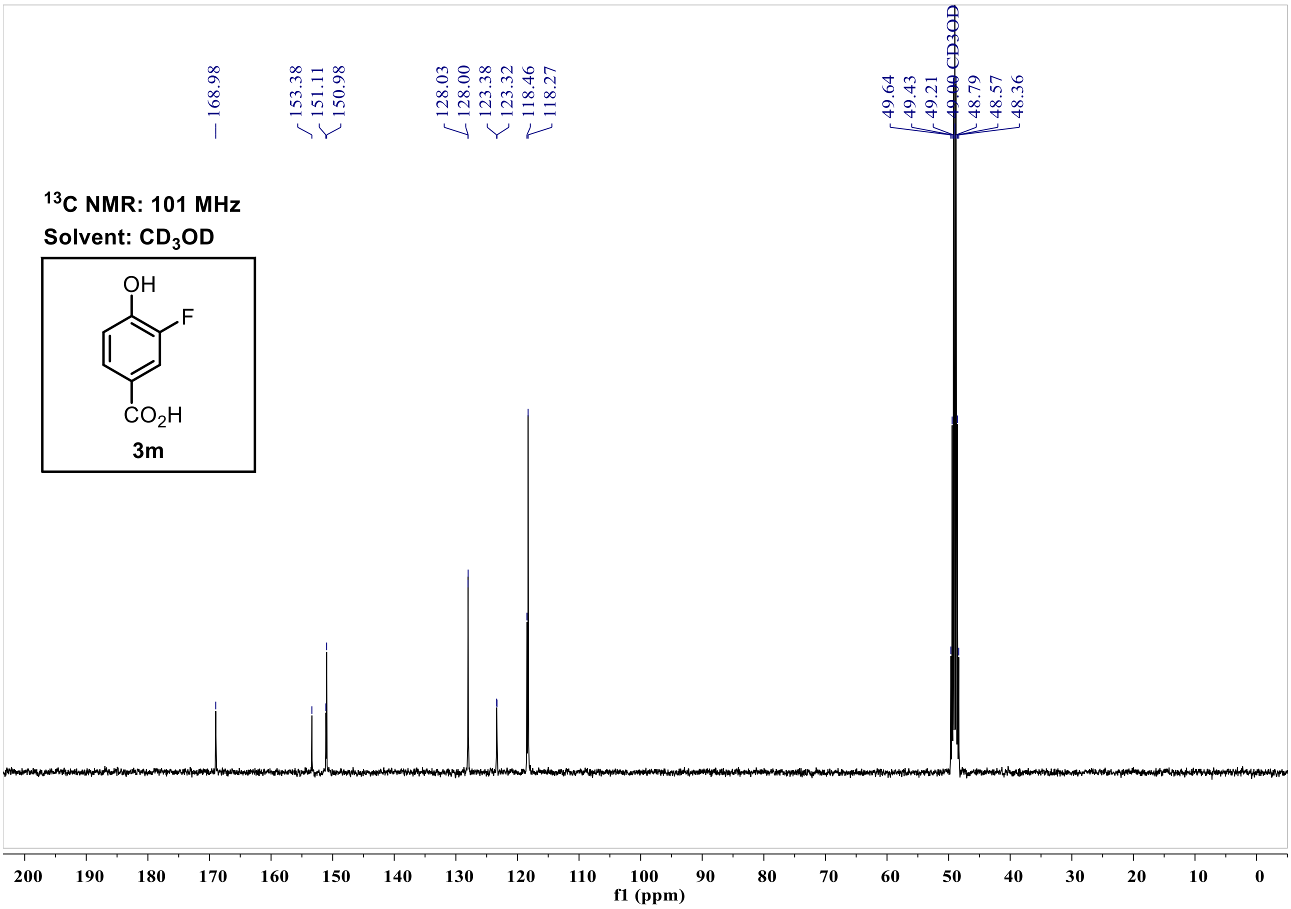
13C NMR: 101 MHz
Solvent: CD3OD
OH
F
CO2H
3m
168.98
153.38
151.11
150.98
128.03
128.00
123.38
123.32
118.46
118.27
49.64
49.43
49.21
49.00 CD3OD
48.79
48.57
48.36
200 190 180 170 160 150 140 130 120 110 100 90 80 70 60 50 40 30 20 10 0
f1 (ppm)

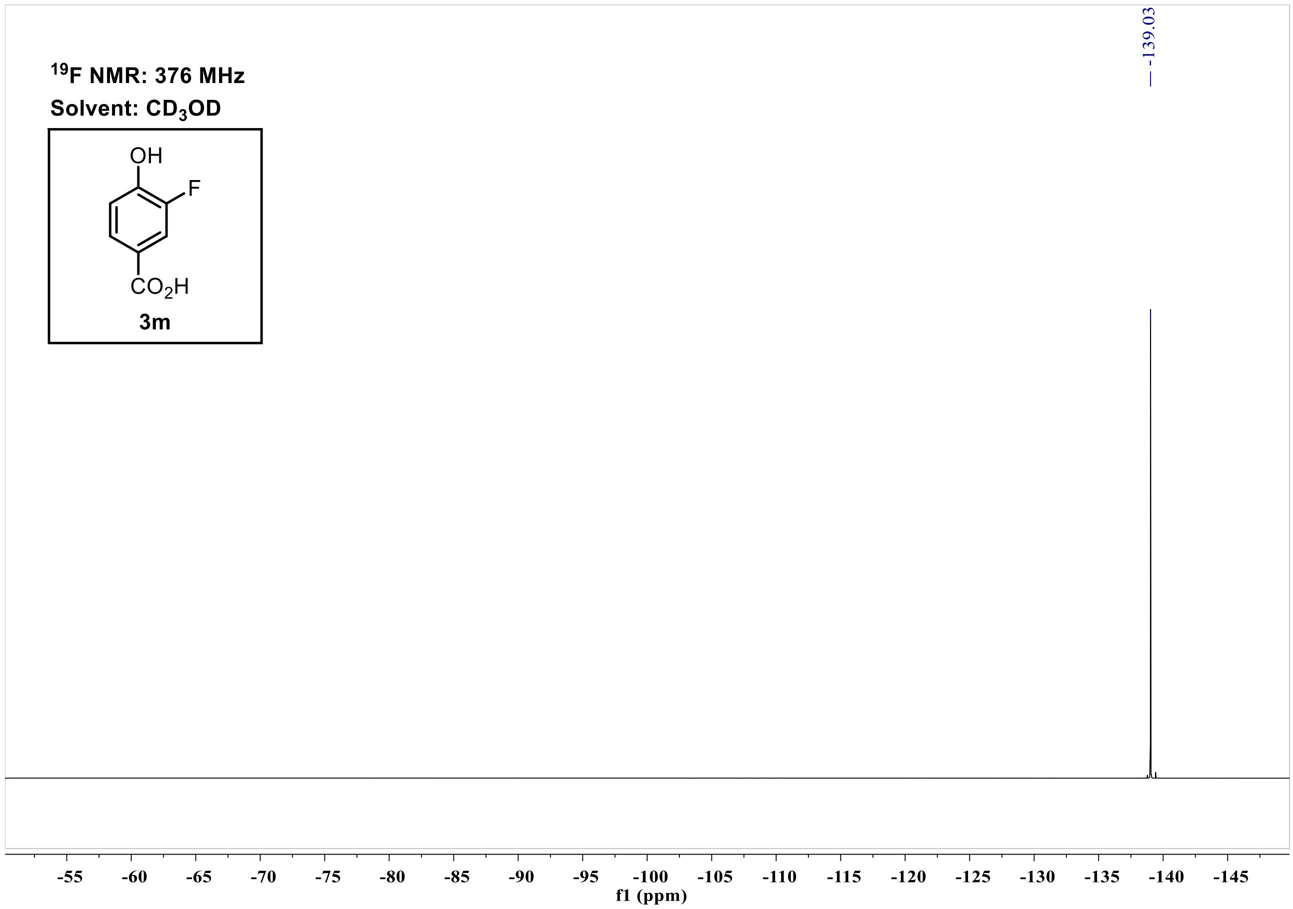
-139.03
19F NMR: 376 MHz
Solvent: CD3OD
OH
F
CO2H
3m
-55 -60 -65 -70 -75 -80 -85 -90 -95 -100 -105 -110 -115 -120 -125 -130 -135 -140 -145
f1 (ppm)

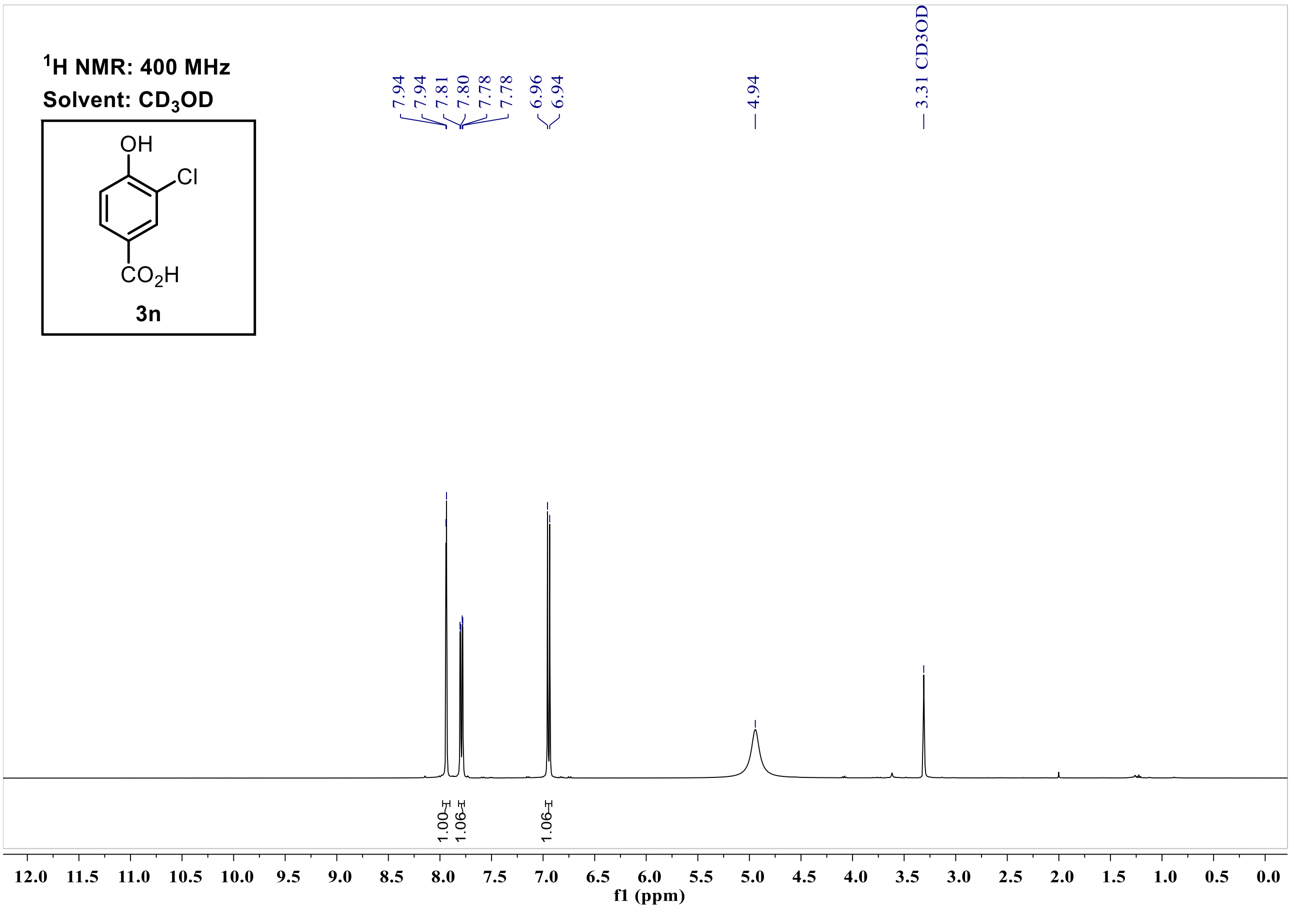

^{1}H NMR: 400 MHz
Solvent: CD_3OD
OH
Cl
CO_2H
3n
7.94
7.94
7.81
7.80
7.78
7.78
6.96
6.94
4.94
3.31 CD3OD
1.00
1.06
1.06
12.0 11.5 11.0 10.5 10.0 9.5 9.0 8.5 8.0 7.5 7.0 6.5 6.0 5.5 5.0 4.5 4.0 3.5 3.0 2.5 2.0 1.5 1.0 0.5 0.0
f1 (ppm)

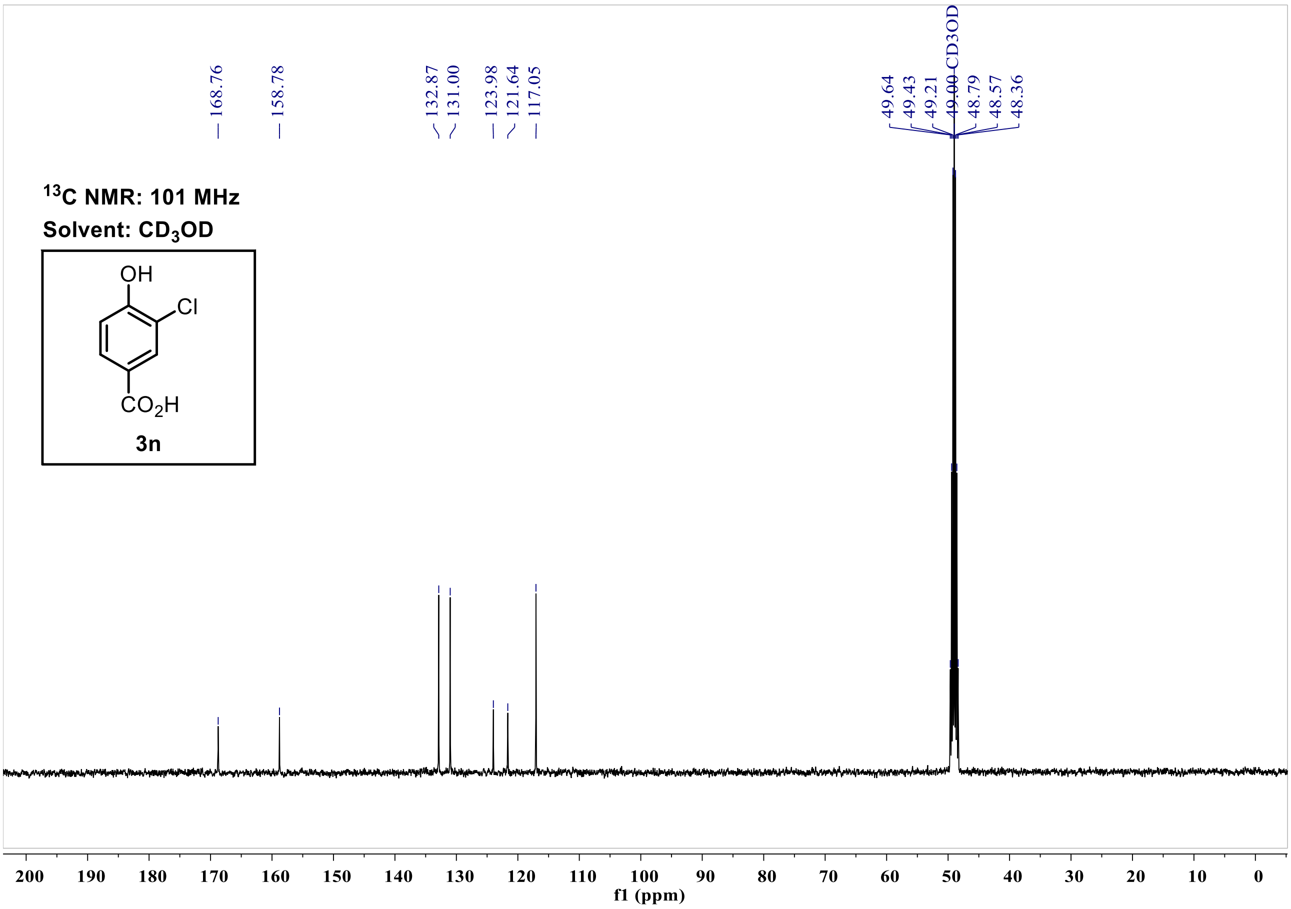

168.76
158.78
132.87
131.00
123.98
121.64
117.05
49.64
49.43
49.21
49.00 CD3OD
48.79
48.57
48.36
^{13}C NMR: 101 MHz
Solvent: CD_3OD
OH
Cl
CO_2H
3n
200 190 180 170 160 150 140 130 120 110 100 90 80 70 60 50 40 30 20 10 0
f1 (ppm)

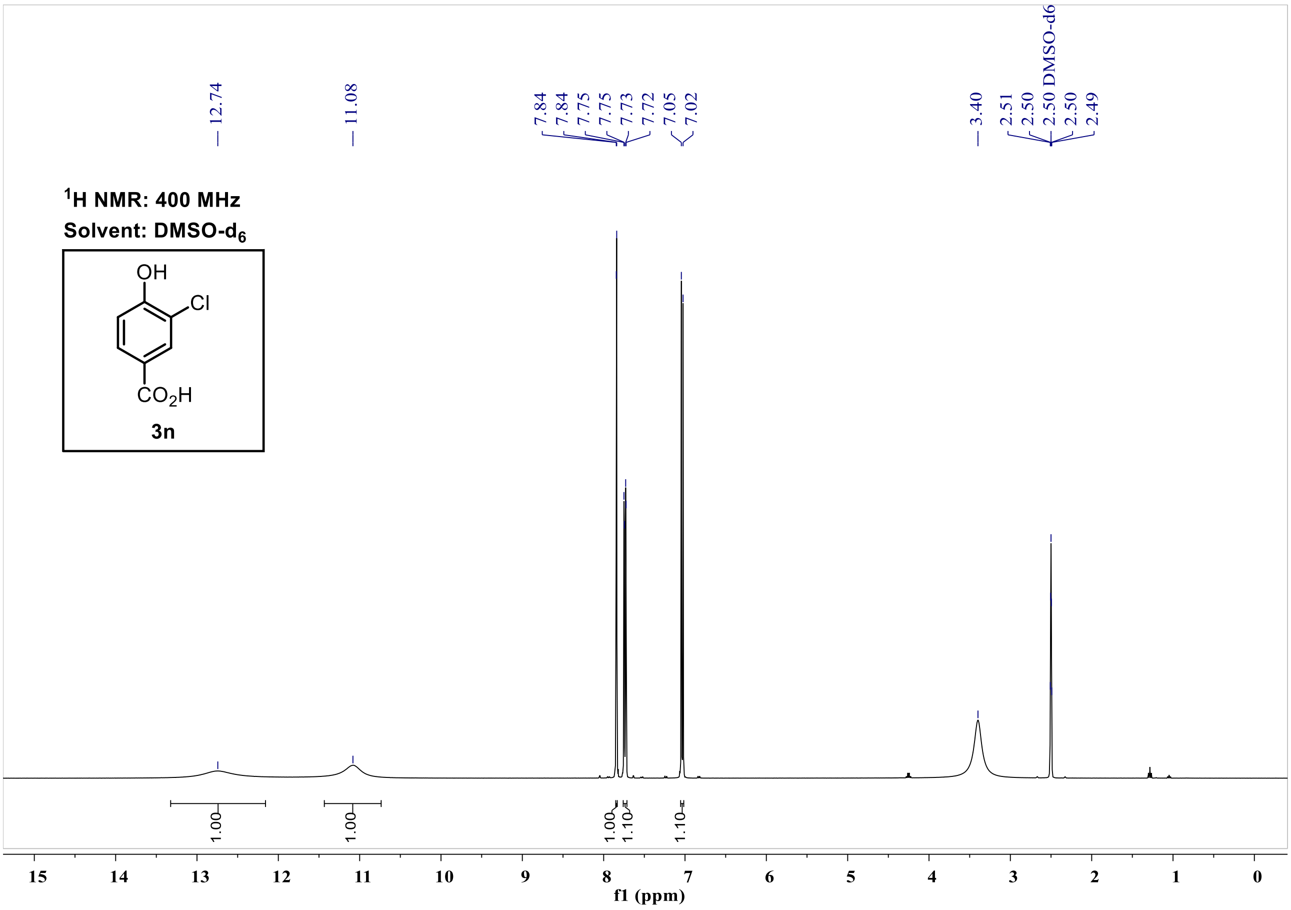
12.74
11.08
7.84
7.84
7.75
7.75
7.73
7.72
7.05
7.02
3.40
2.51
2.50
2.50 DMSO-d6
2.50
2.49
1H NMR: 400 MHz
Solvent: DMSO-d6
OH
Cl
CO2H
3n
1.00
1.00
1.00
1.10
1.10
15
14
13
12
11
10
9
8
7
6
5
4
3
2
1
0
f1 (ppm)

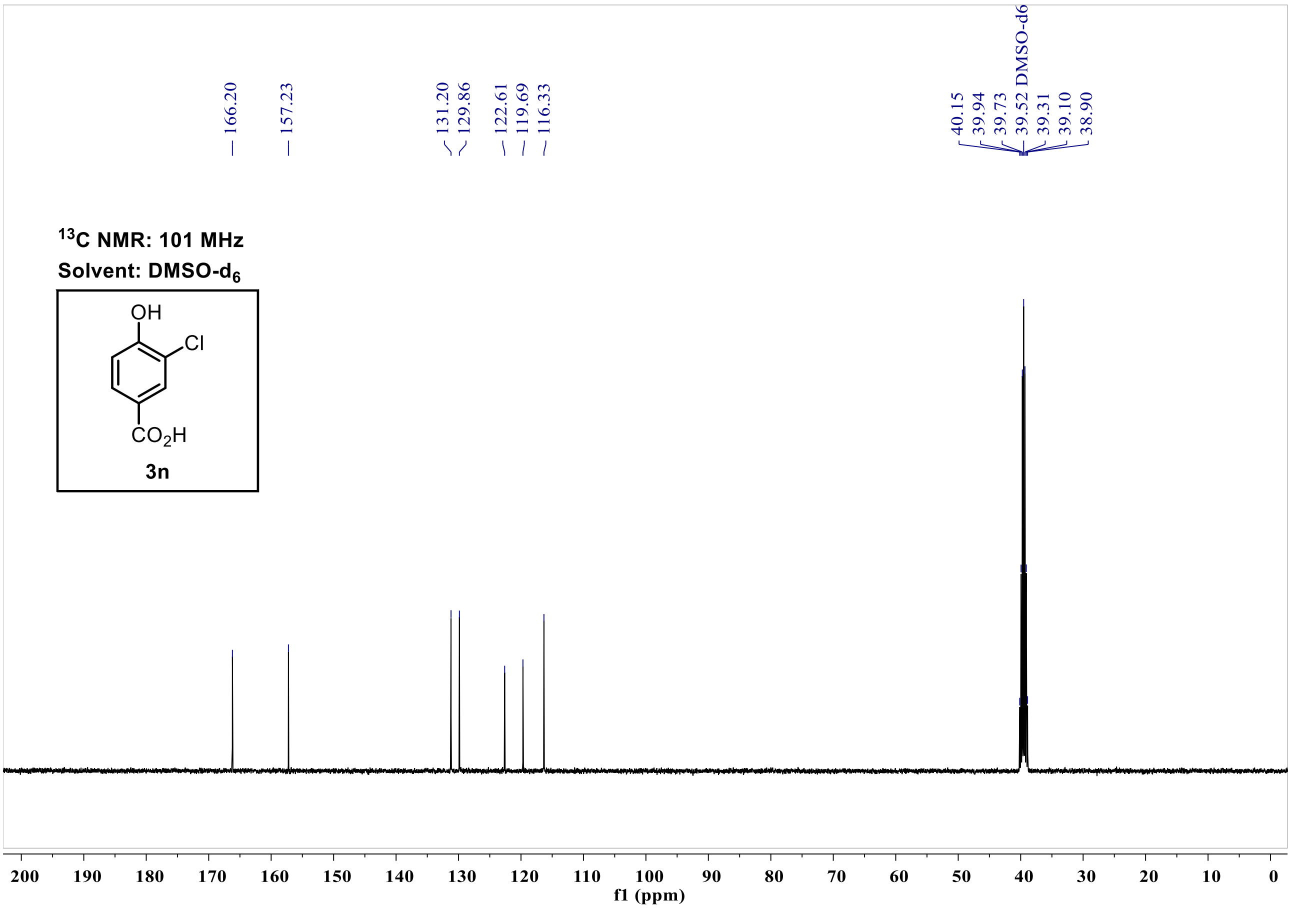
166.20
157.23
131.20
129.86
122.61
119.69
116.33
40.15
39.94
39.73
39.52 DMSO-d6
39.31
39.10
38.90
13C NMR: 101 MHz
Solvent: DMSO-d6
OH
Cl
CO2H
3n
200
190
180
170
160
150
140
130
120
110
100
90
80
70
60
50
40
30
20
10
0
f1 (ppm)

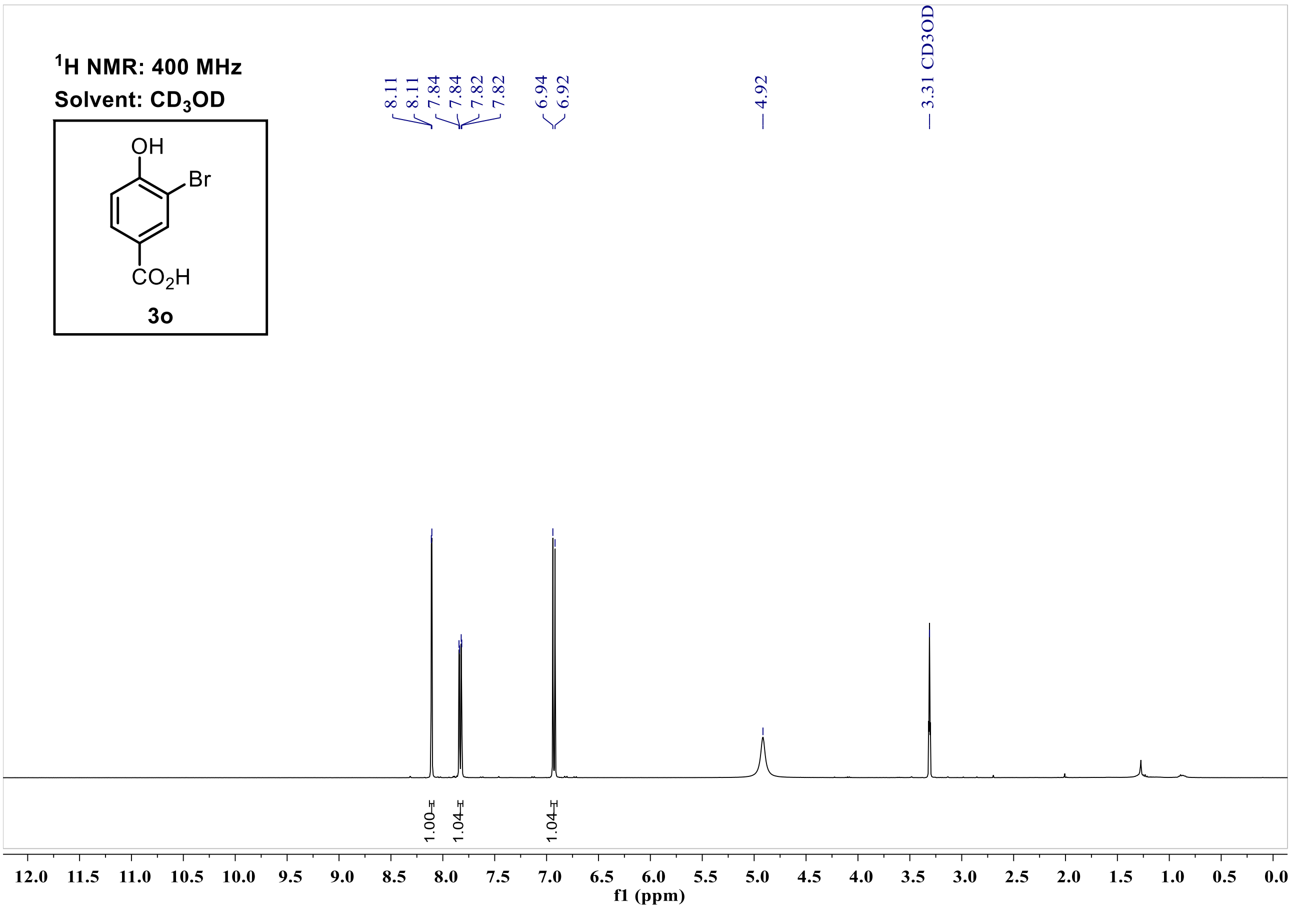

1H NMR: 400 MHz
Solvent: CD3OD
OH
Br
CO2H
3o
8.11
8.11
7.84
7.84
7.82
7.82
6.94
6.92
4.92
3.31 CD3OD
1.00
1.04
1.04
12.0 11.5 11.0 10.5 10.0 9.5 9.0 8.5 8.0 7.5 7.0 6.5 6.0 5.5 5.0 4.5 4.0 3.5 3.0 2.5 2.0 1.5 1.0 0.5 0.0
f1 (ppm)

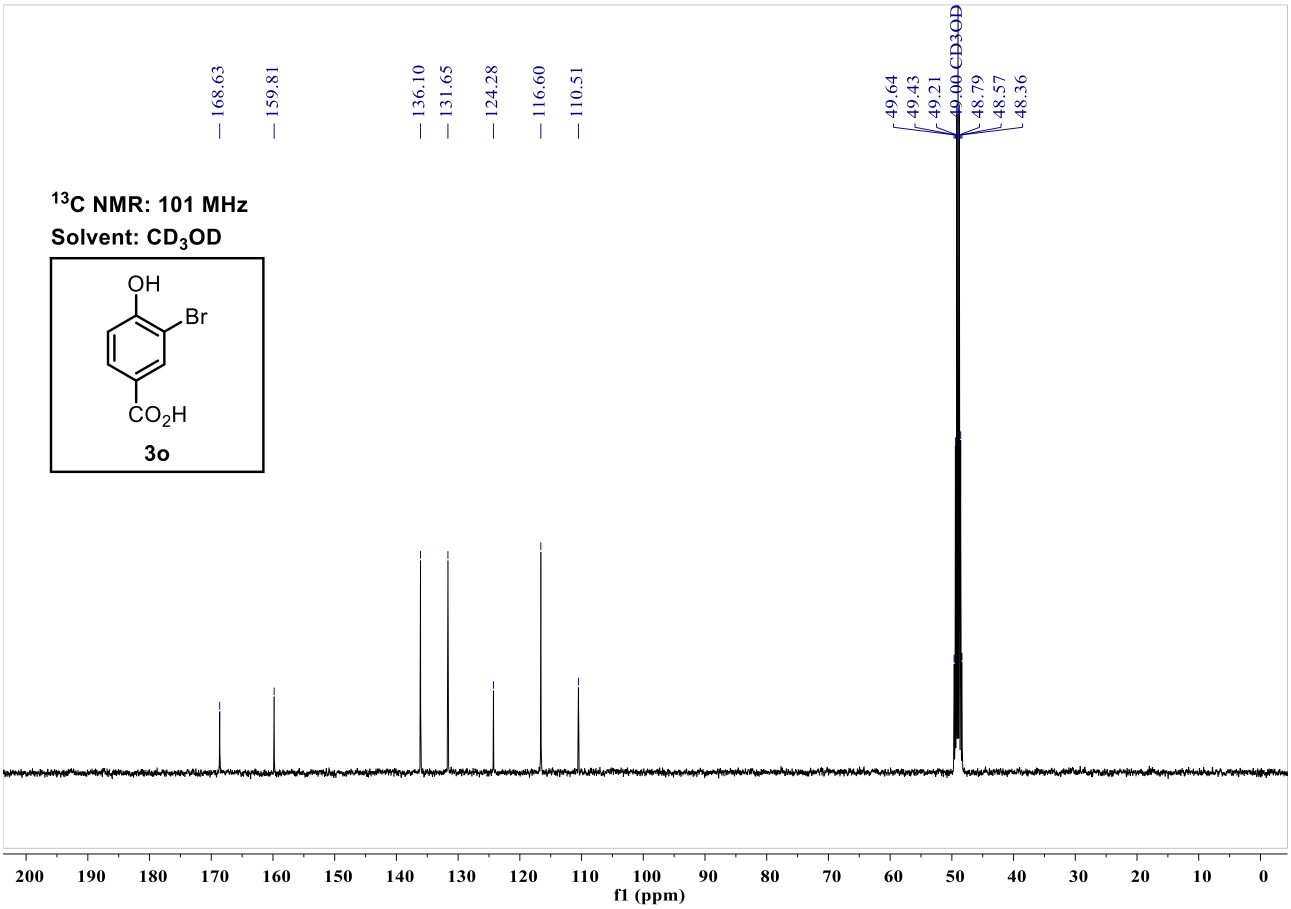

13C NMR: 101 MHz
Solvent: CD3OD
OH
Br
CO2H
3o
168.63
159.81
136.10
131.65
124.28
116.60
110.51
49.64
49.43
49.21
49.00 CD3OD
48.79
48.57
48.36
200 190 180 170 160 150 140 130 120 110 100 90 80 70 60 50 40 30 20 10 0
f1 (ppm)

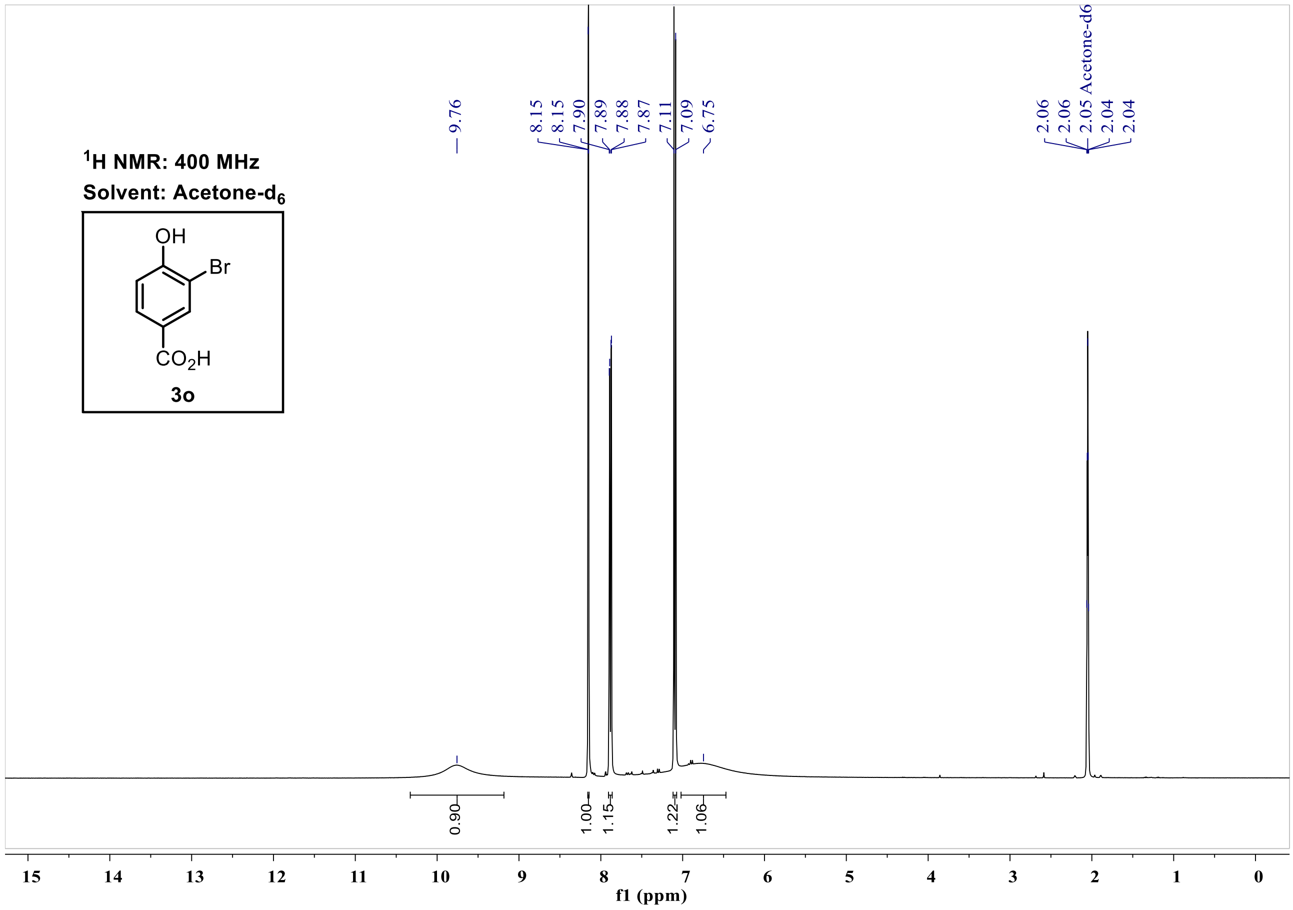

9.76
8.15
8.15
7.90
7.89
7.88
7.87
7.11
7.09
6.75
2.06
2.06
2.05 Acetone-d6
2.04
2.04
^{1}H NMR: 400 MHz
Solvent: Acetone-d6
OH
Br
CO2H
3o
0.90
1.00
1.15
1.22
1.06
15
14
13
12
11
10
9
8
7
6
5
4
3
2
1
0
f1 (ppm)

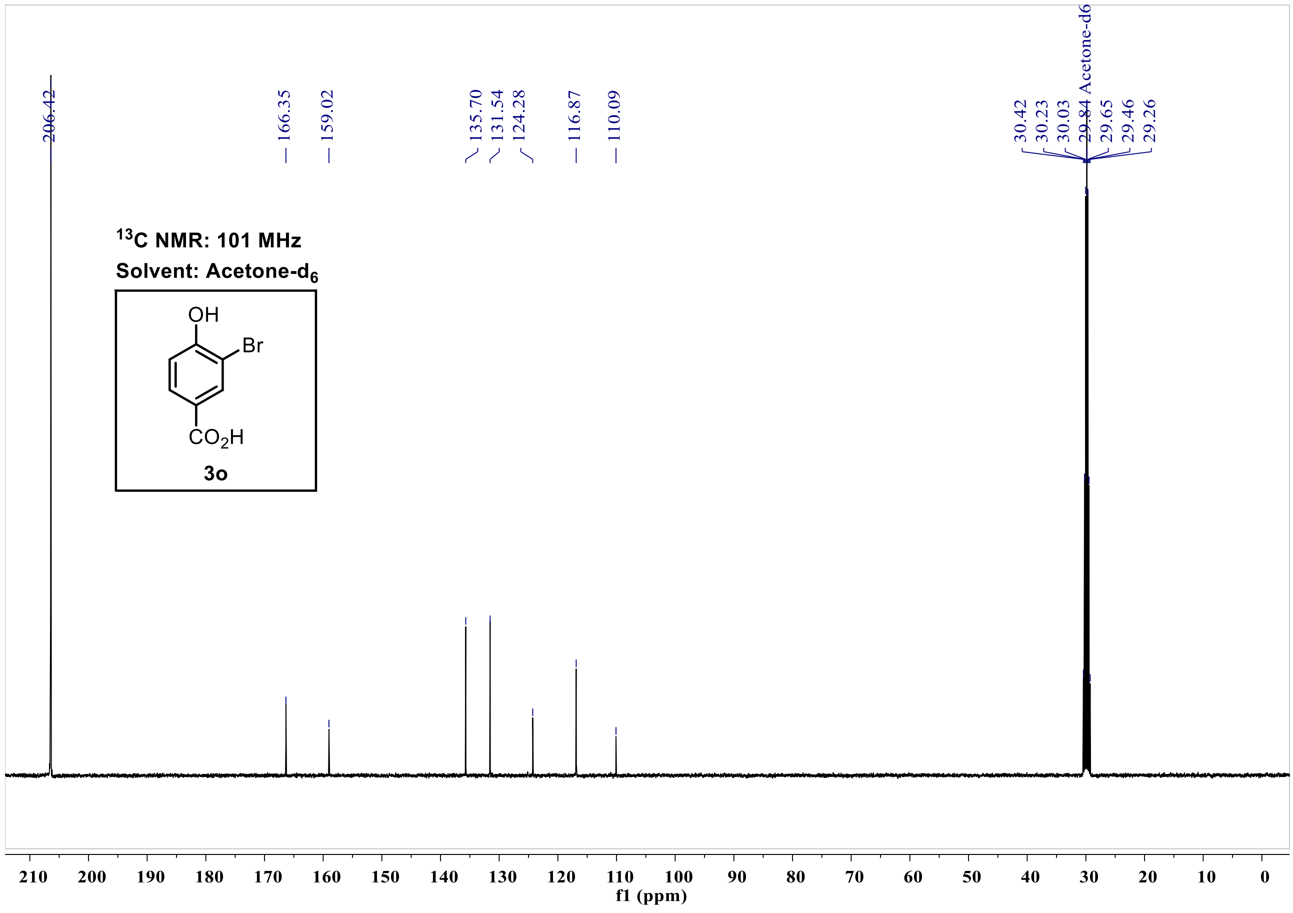

206.42
166.35
159.02
135.70
131.54
124.28
116.87
110.09
30.42
30.23
30.03
29.84 Acetone-d6
29.65
29.46
29.26
^{13}C NMR: 101 MHz
Solvent: Acetone-d6
OH
Br
CO2H
3o
210
200
190
180
170
160
150
140
130
120
110
100
90
80
70
60
50
40
30
20
10
0
f1 (ppm)

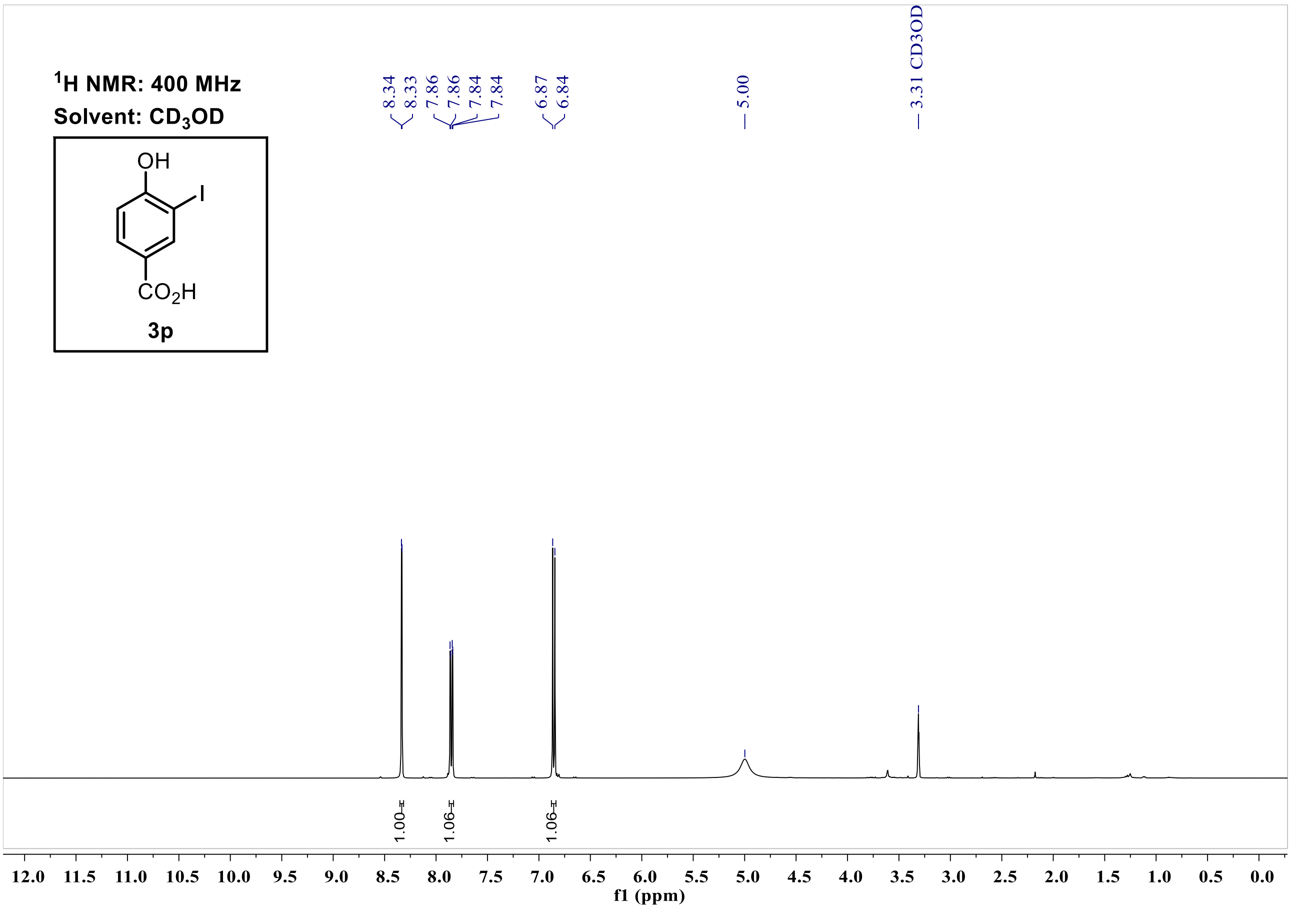

^{1}H NMR: 400 MHz
Solvent: CD_3OD
OH
I
CO_2H
3p
8.34
8.33
7.86
7.86
7.84
7.84
6.87
6.84
5.00
3.31 CD3OD
1.00
1.06
1.06
12.0 11.5 11.0 10.5 10.0 9.5 9.0 8.5 8.0 7.5 7.0 6.5 6.0 5.5 5.0 4.5 4.0 3.5 3.0 2.5 2.0 1.5 1.0 0.5 0.0
f1 (ppm)

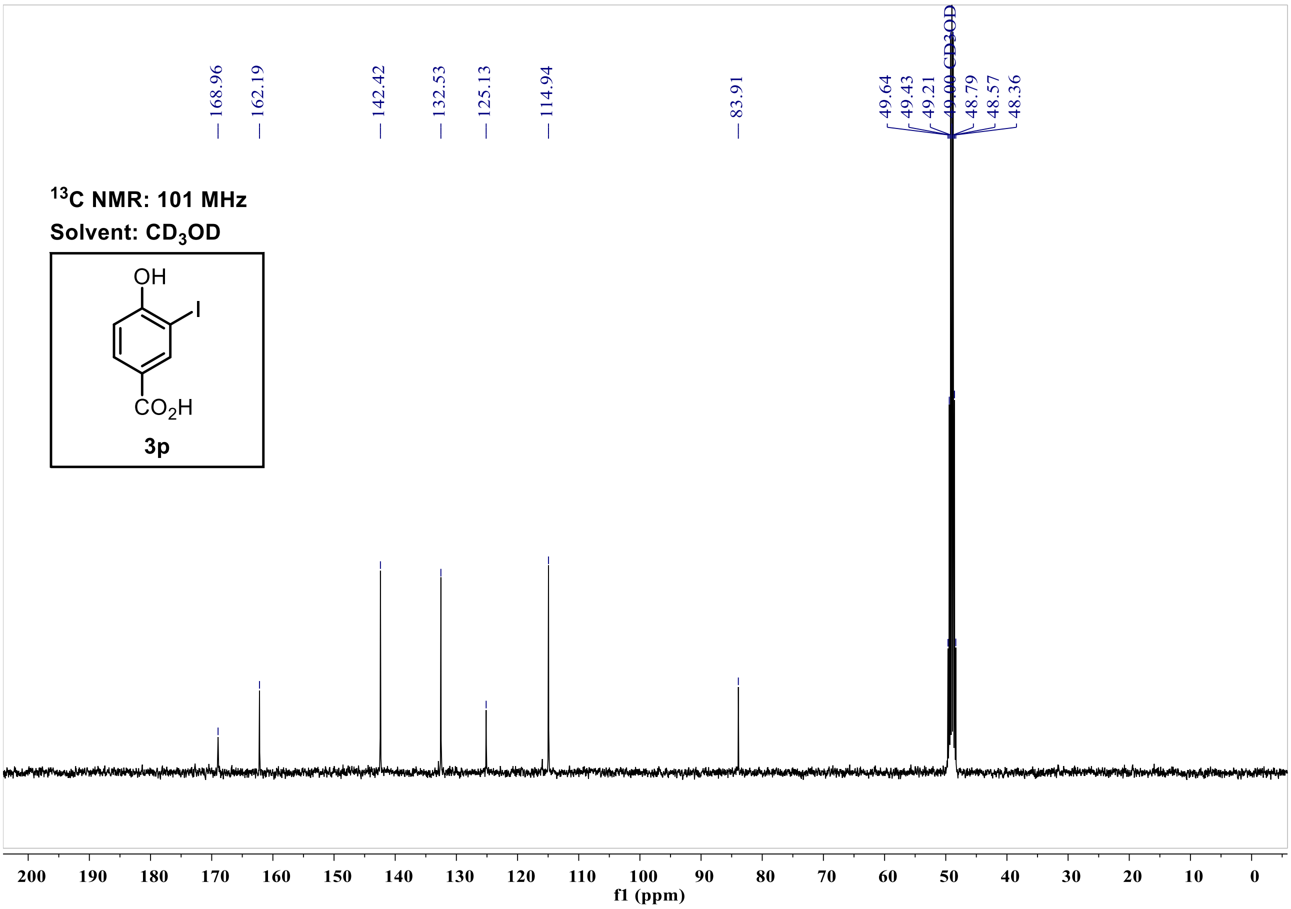

168.96
162.19
142.42
132.53
125.13
114.94
83.91
49.64
49.43
49.21
49.00 CD3OD
48.79
48.57
48.36
^{13}C NMR: 101 MHz
Solvent: CD_3OD
OH
I
CO_2H
3p
200 190 180 170 160 150 140 130 120 110 100 90 80 70 60 50 40 30 20 10 0
f1 (ppm)

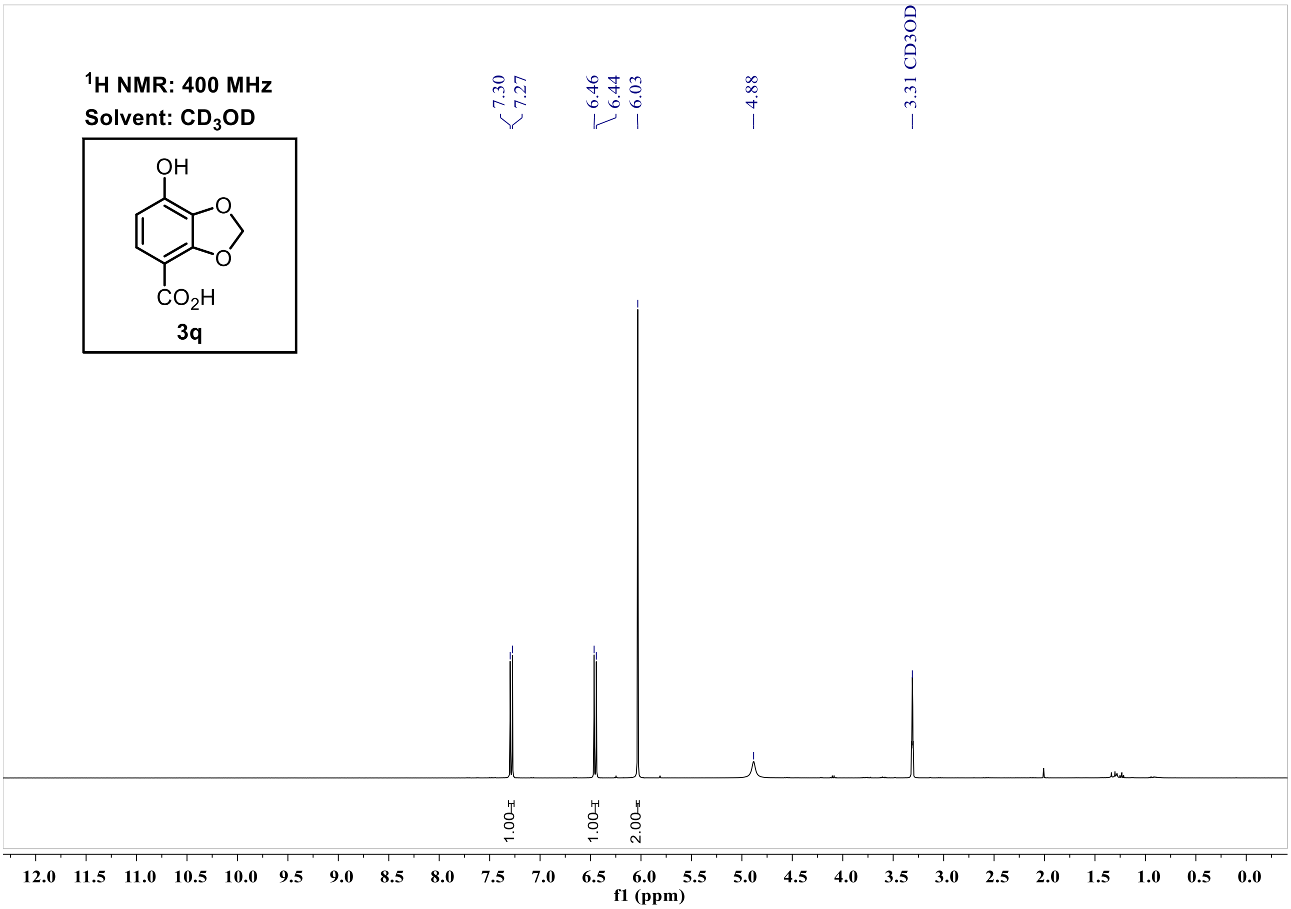

1H NMR: 400 MHz
Solvent: CD3OD
OH
O
O
CO2H
3q
7.30
7.27
6.46
6.44
6.03
4.88
3.31 CD3OD
1.00
1.00
2.00
12.0 11.5 11.0 10.5 10.0 9.5 9.0 8.5 8.0 7.5 7.0 6.5 6.0 5.5 5.0 4.5 4.0 3.5 3.0 2.5 2.0 1.5 1.0 0.5 0.0
f1 (ppm)

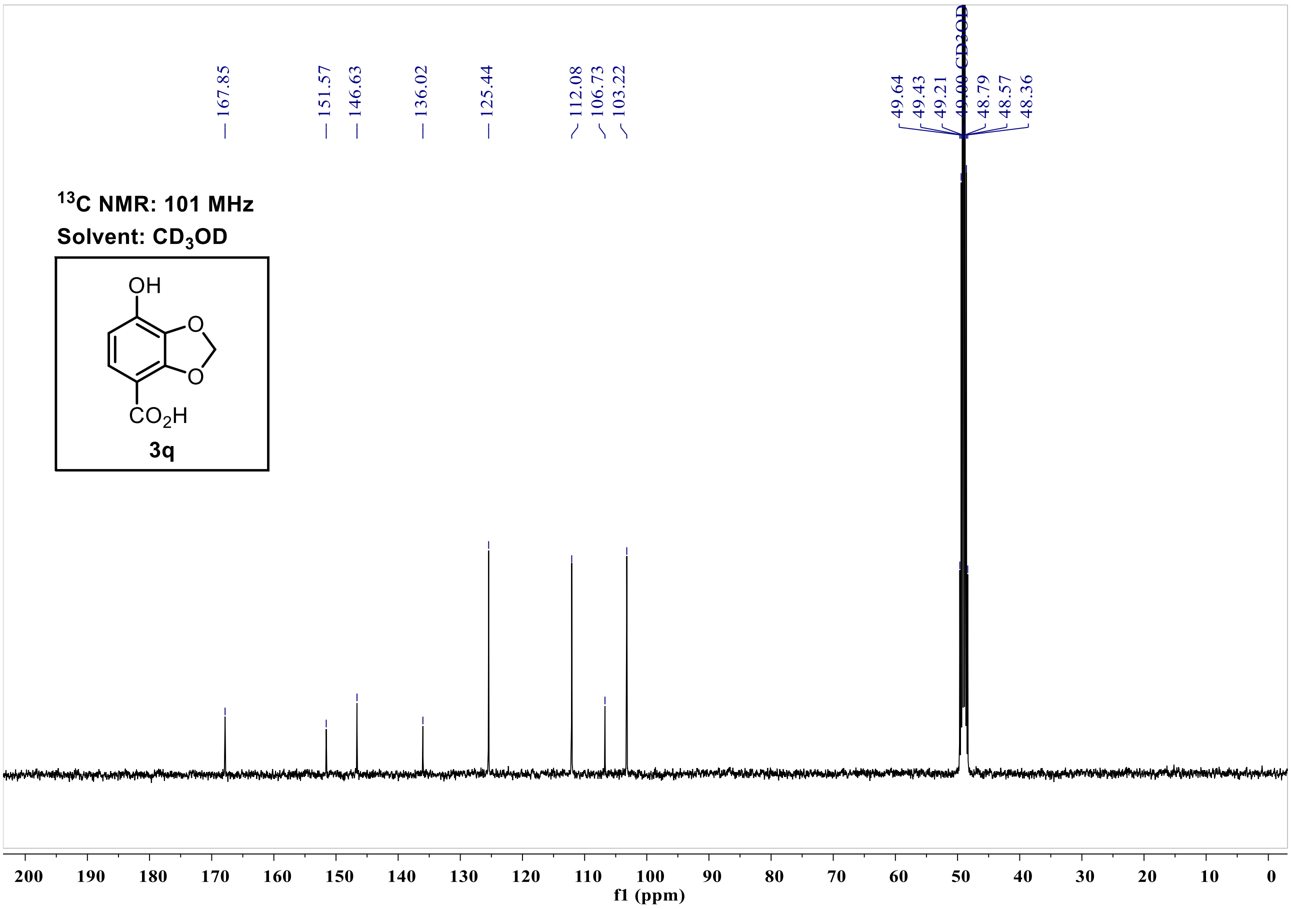

167.85
151.57
146.63
136.02
125.44
112.08
106.73
103.22
49.64
49.43
49.21
49.00 CD3OD
48.79
48.57
48.36
13C NMR: 101 MHz
Solvent: CD3OD
OH
O
O
CO2H
3q
200 190 180 170 160 150 140 130 120 110 100 90 80 70 60 50 40 30 20 10 0
f1 (ppm)

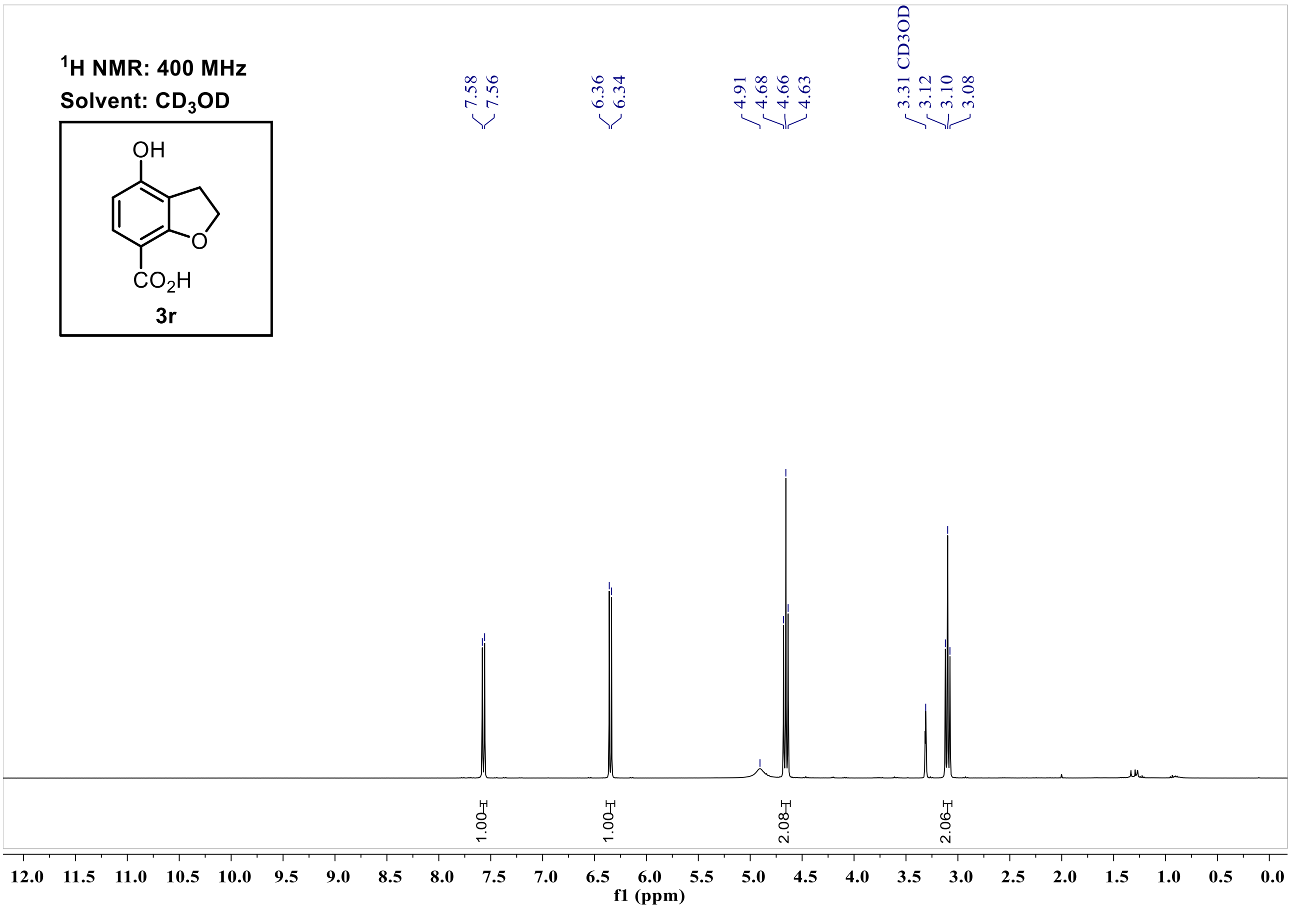

1H NMR: 400 MHz
Solvent: CD3OD
OH
O
CO2H
3r
7.58
7.56
6.36
6.34
4.91
4.68
4.66
4.63
3.31 CD3OD
3.12
3.10
3.08
1.00
1.00
2.08
2.06
12.0 11.5 11.0 10.5 10.0 9.5 9.0 8.5 8.0 7.5 7.0 6.5 6.0 5.5 5.0 4.5 4.0 3.5 3.0 2.5 2.0 1.5 1.0 0.5 0.0
f1 (ppm)

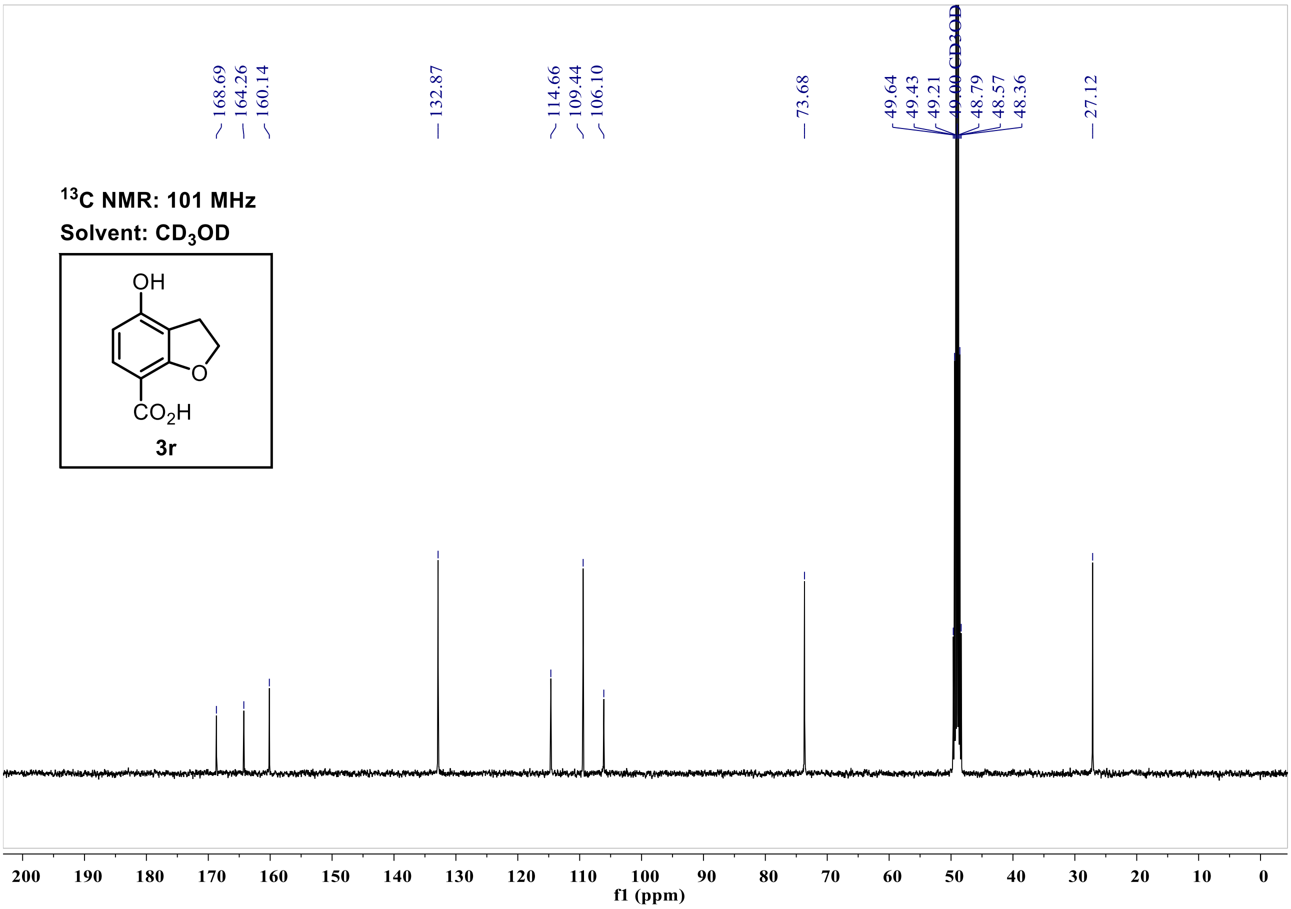

13C NMR: 101 MHz
Solvent: CD3OD
OH
O
CO2H
3r
168.69
164.26
160.14
132.87
114.66
109.44
106.10
73.68
49.64
49.43
49.21
49.00 CD3OD
48.79
48.57
48.36
27.12
200 190 180 170 160 150 140 130 120 110 100 90 80 70 60 50 40 30 20 10 0
f1 (ppm)

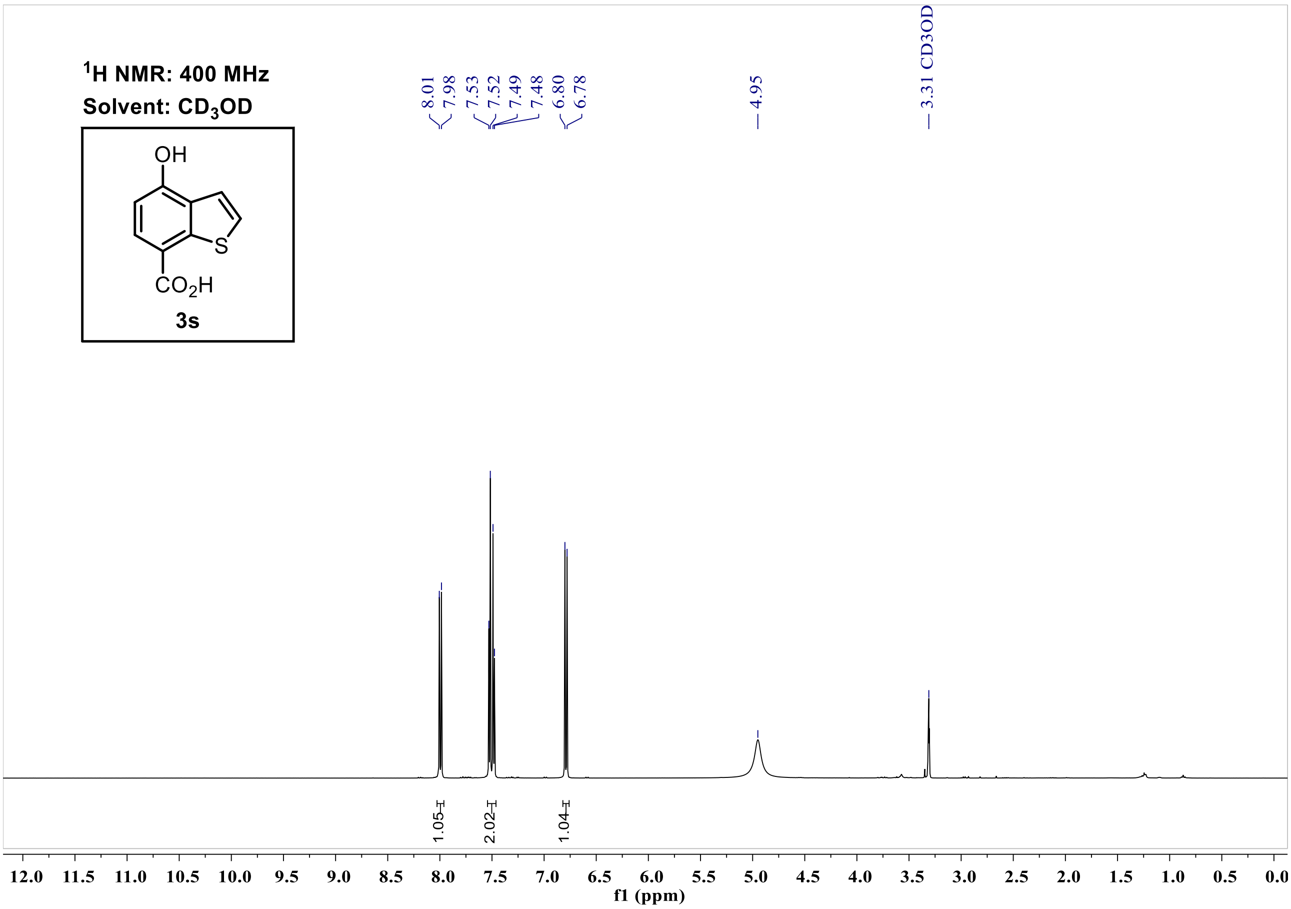
1H NMR: 400 MHz
Solvent: CD3OD
OH
S
CO2H
3s
8.01
7.98
7.53
7.52
7.49
7.48
6.80
6.78
4.95
3.31 CD3OD
1.05
2.02
1.04
12.0 11.5 11.0 10.5 10.0 9.5 9.0 8.5 8.0 7.5 7.0 6.5 6.0 5.5 5.0 4.5 4.0 3.5 3.0 2.5 2.0 1.5 1.0 0.5 0.0
f1 (ppm)

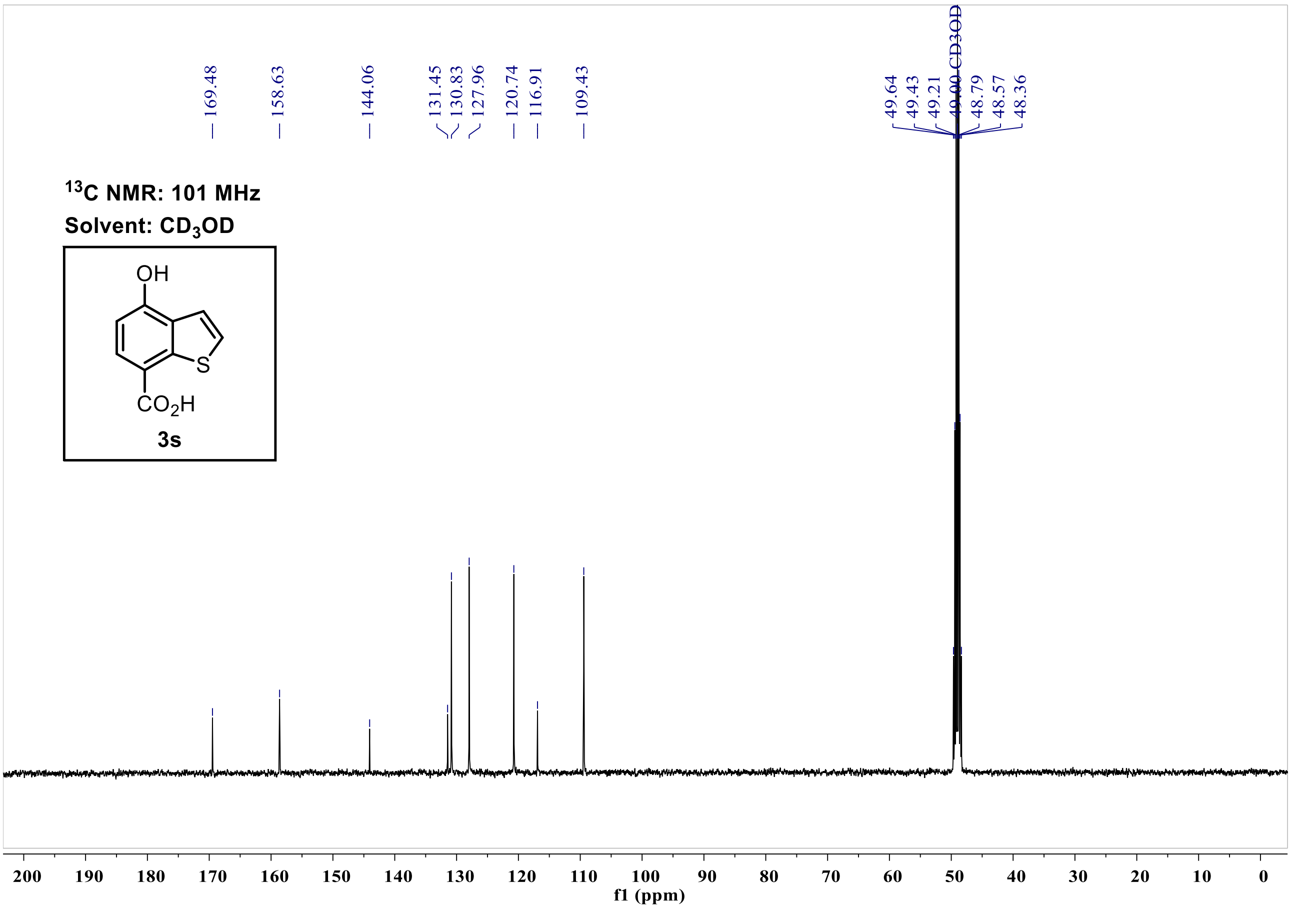
169.48
158.63
144.06
131.45
130.83
127.96
120.74
116.91
109.43
49.64
49.43
49.21
49.00 CD3OD
48.79
48.57
48.36
13C NMR: 101 MHz
Solvent: CD3OD
OH
S
CO2H
3s
200 190 180 170 160 150 140 130 120 110 100 90 80 70 60 50 40 30 20 10 0
f1 (ppm)

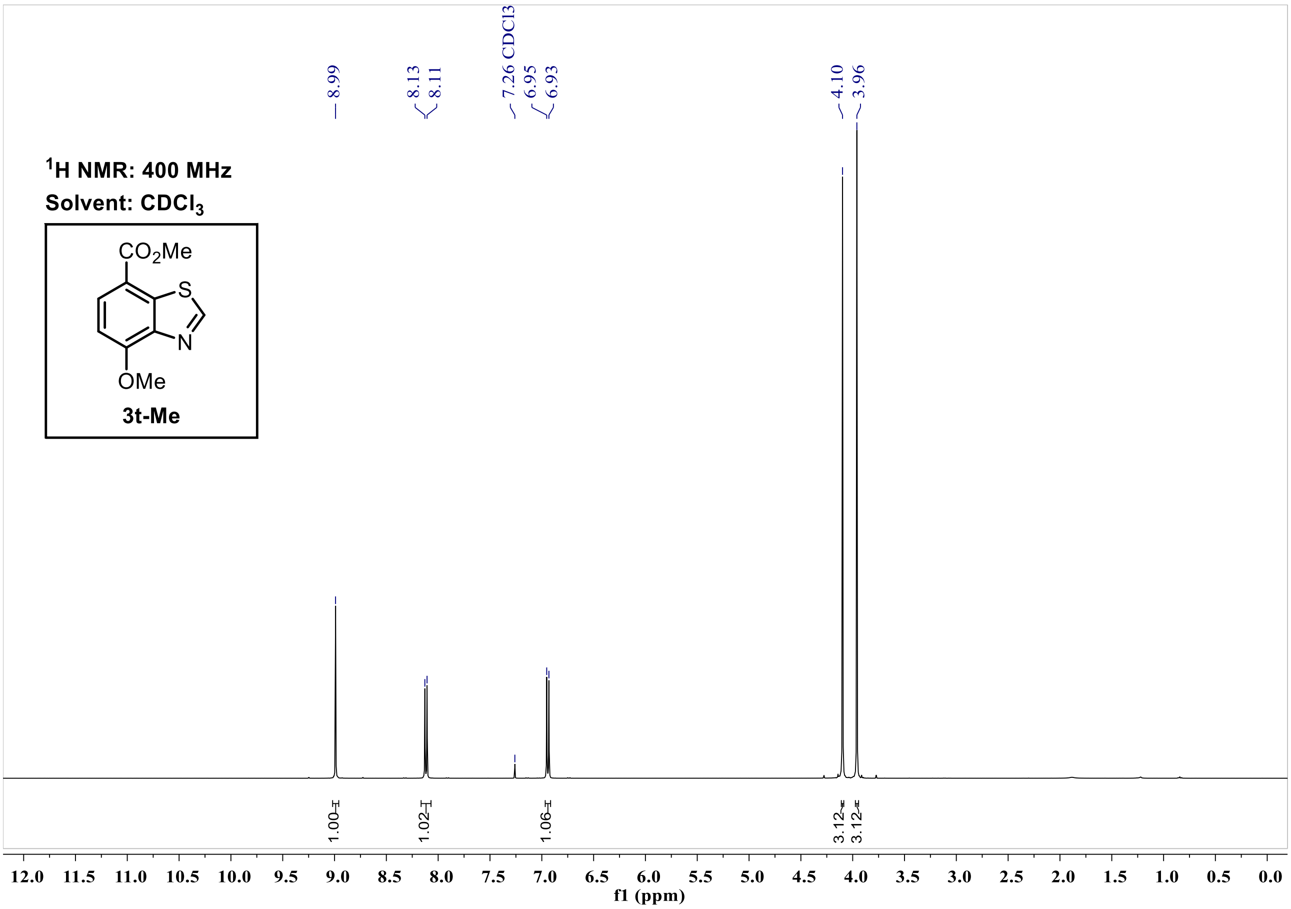

8.99
8.13
8.11
7.26 CDCl3
6.95
6.93
4.10
3.96
1H NMR: 400 MHz
Solvent: CDCl3
CO2Me
S
N
OMe
3t-Me
1.00
1.02
1.06
3.12
3.12
12.0 11.5 11.0 10.5 10.0 9.5 9.0 8.5 8.0 7.5 7.0 6.5 6.0 5.5 5.0 4.5 4.0 3.5 3.0 2.5 2.0 1.5 1.0 0.5 0.0
f1 (ppm)

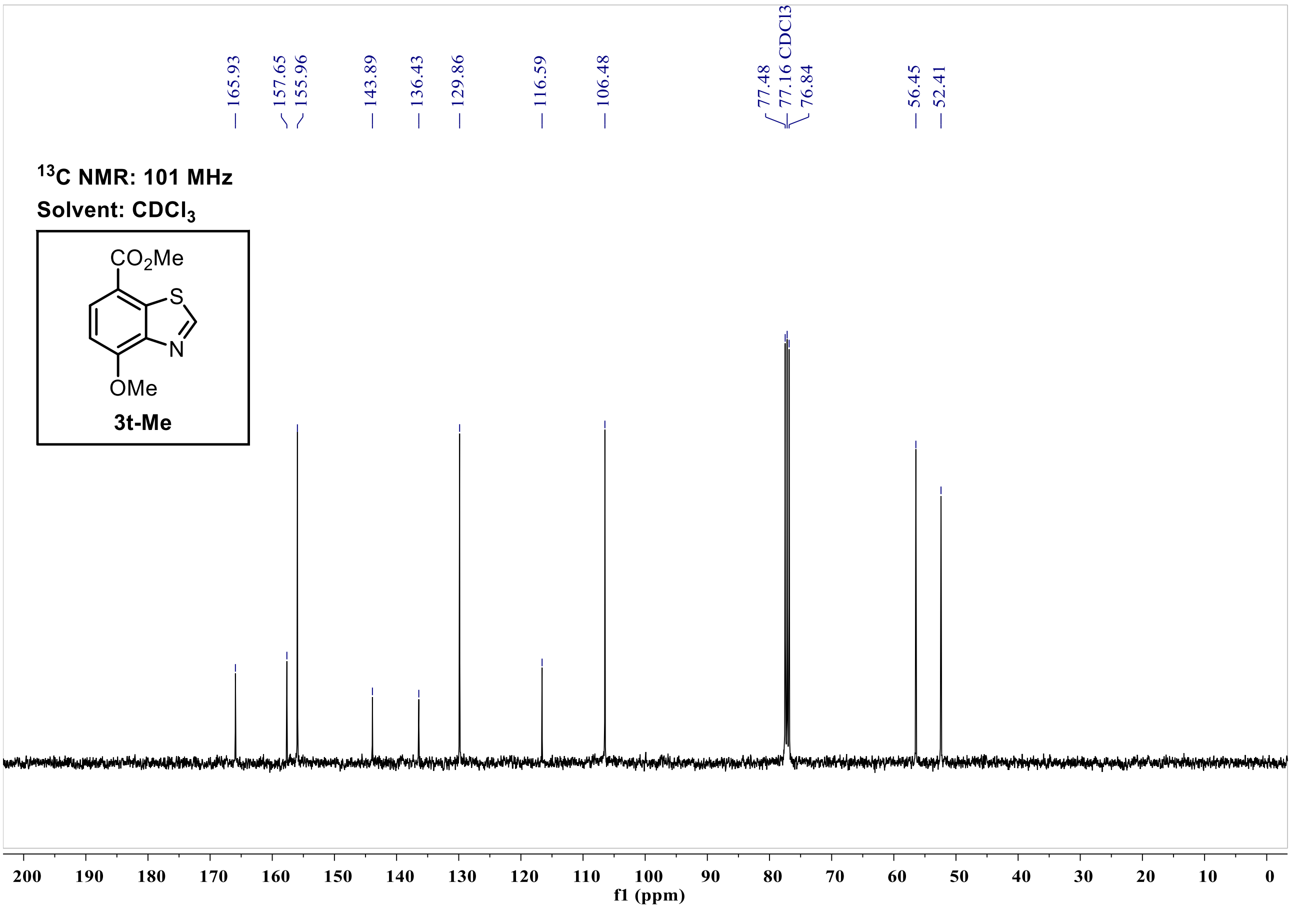

165.93
157.65
155.96
143.89
136.43
129.86
116.59
106.48
77.48
77.16 CDCl3
76.84
56.45
52.41
13C NMR: 101 MHz
Solvent: CDCl3
CO2Me
S
N
OMe
3t-Me
200 190 180 170 160 150 140 130 120 110 100 90 80 70 60 50 40 30 20 10 0
f1 (ppm)

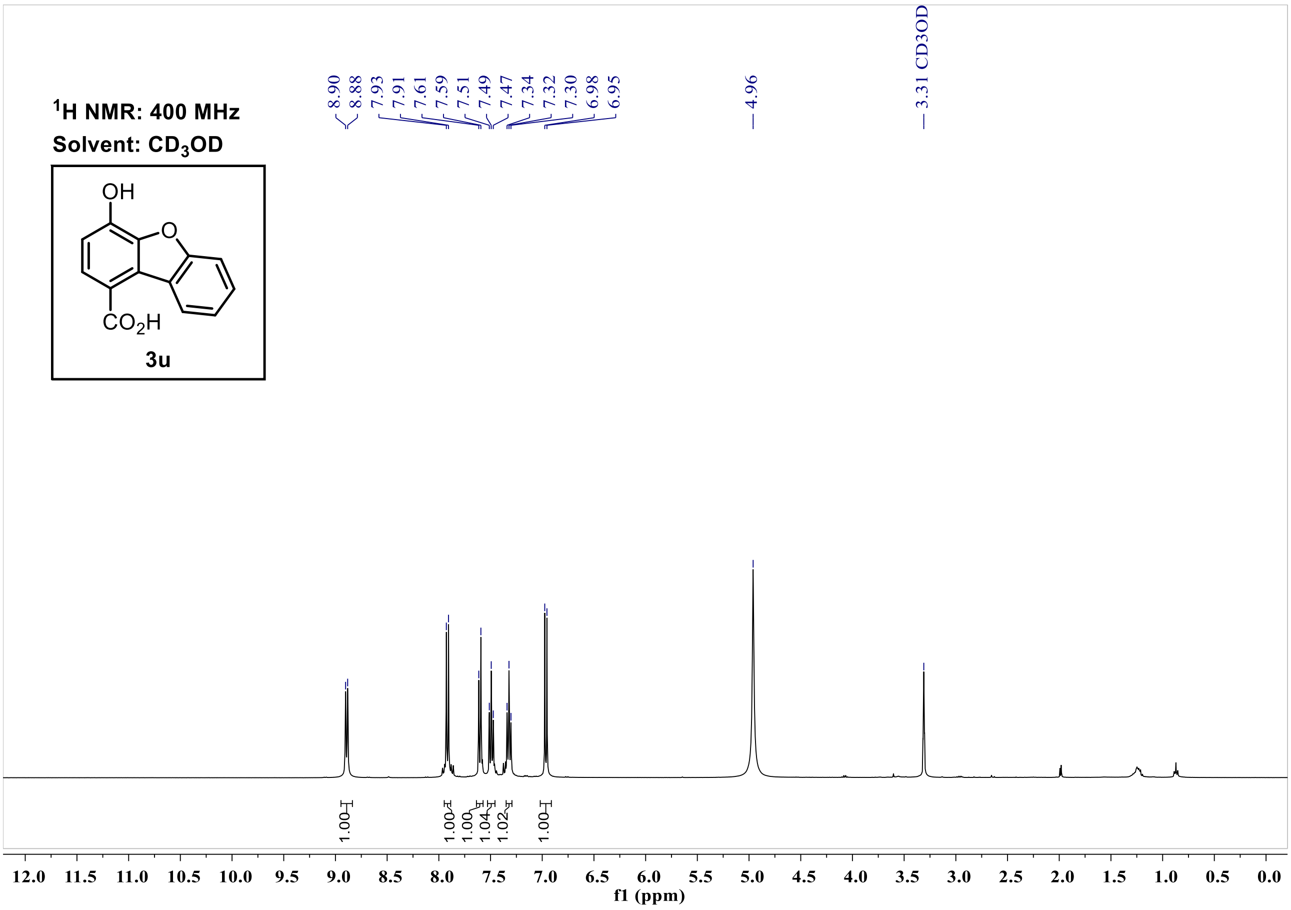

8.90
8.88
7.93
7.91
7.61
7.59
7.51
7.49
7.47
7.34
7.32
7.30
6.98
6.95
4.96
3.31 CD3OD
^{1}H NMR: 400 MHz
Solvent: CD$_3$OD
OH
O
CO$_2$H
3u
1.00
1.00
1.00
1.04
1.02
1.00
12.0 11.5 11.0 10.5 10.0 9.5 9.0 8.5 8.0 7.5 7.0 6.5 6.0 5.5 5.0 4.5 4.0 3.5 3.0 2.5 2.0 1.5 1.0 0.5 0.0
f1 (ppm)

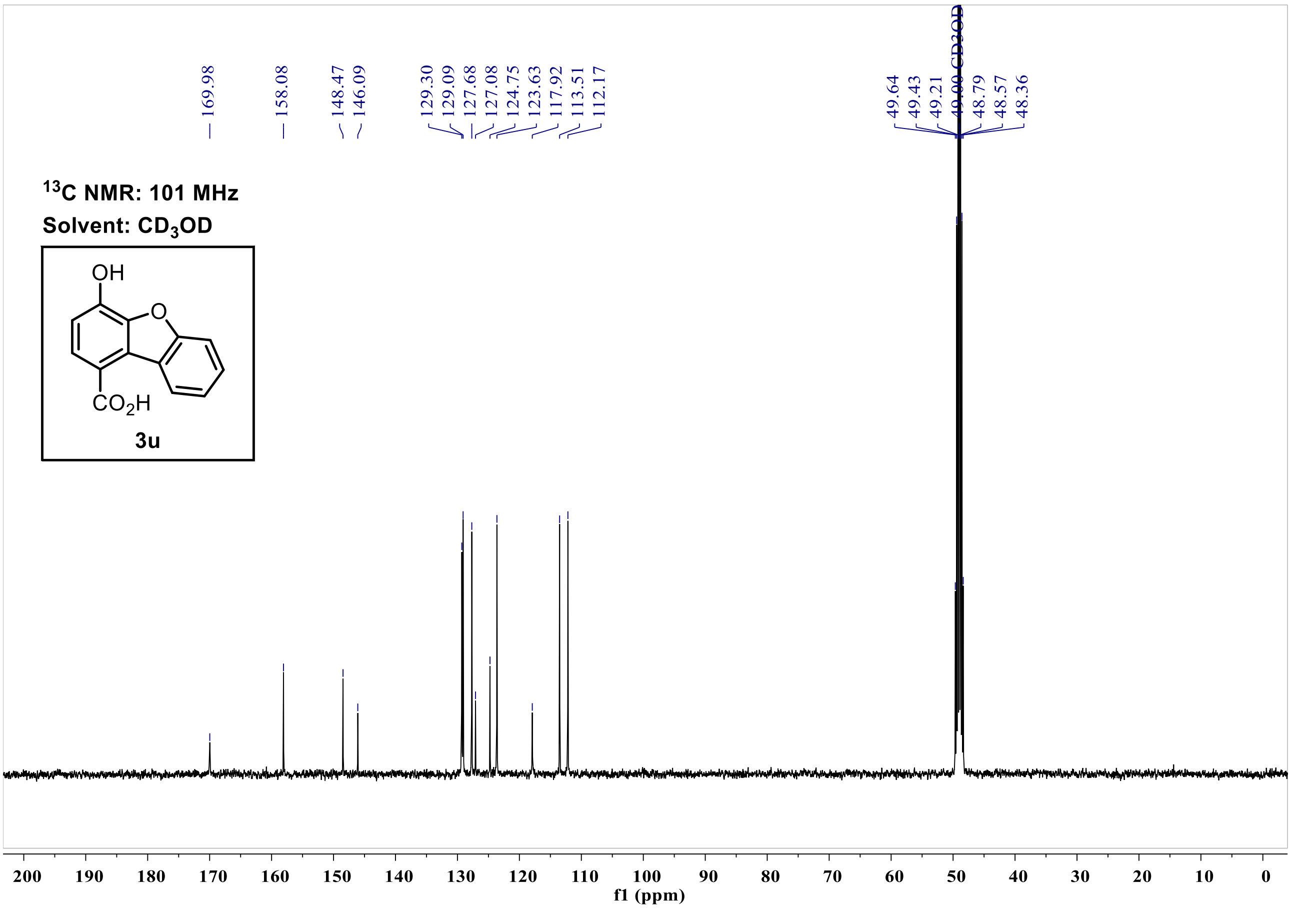

169.98
158.08
148.47
146.09
129.30
129.09
127.68
127.08
124.75
123.63
117.92
113.51
112.17
49.64
49.43
49.21
49.00 CD3OD
48.79
48.57
48.36
^{13}C NMR: 101 MHz
Solvent: CD$_3$OD
OH
O
CO$_2$H
3u
200 190 180 170 160 150 140 130 120 110 100 90 80 70 60 50 40 30 20 10 0
f1 (ppm)

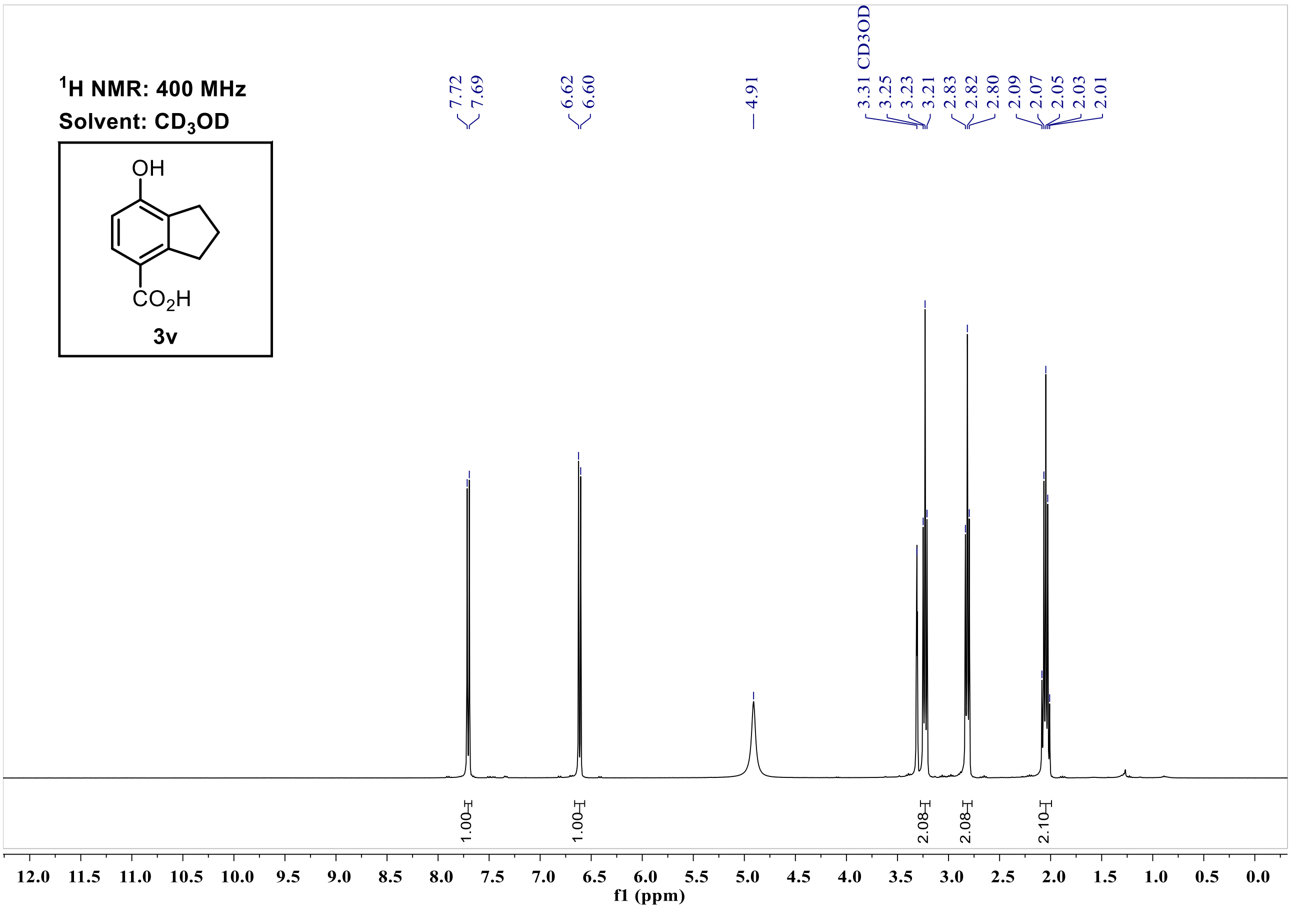

1H NMR: 400 MHz
Solvent: CD3OD
OH
CO2H
3v
7.72
7.69
6.62
6.60
4.91
3.31 CD3OD
3.25
3.23
3.21
2.83
2.82
2.80
2.09
2.07
2.05
2.03
2.01
1.00
1.00
2.08
2.08
2.10
12.0 11.5 11.0 10.5 10.0 9.5 9.0 8.5 8.0 7.5 7.0 6.5 6.0 5.5 5.0 4.5 4.0 3.5 3.0 2.5 2.0 1.5 1.0 0.5 0.0
f1 (ppm)

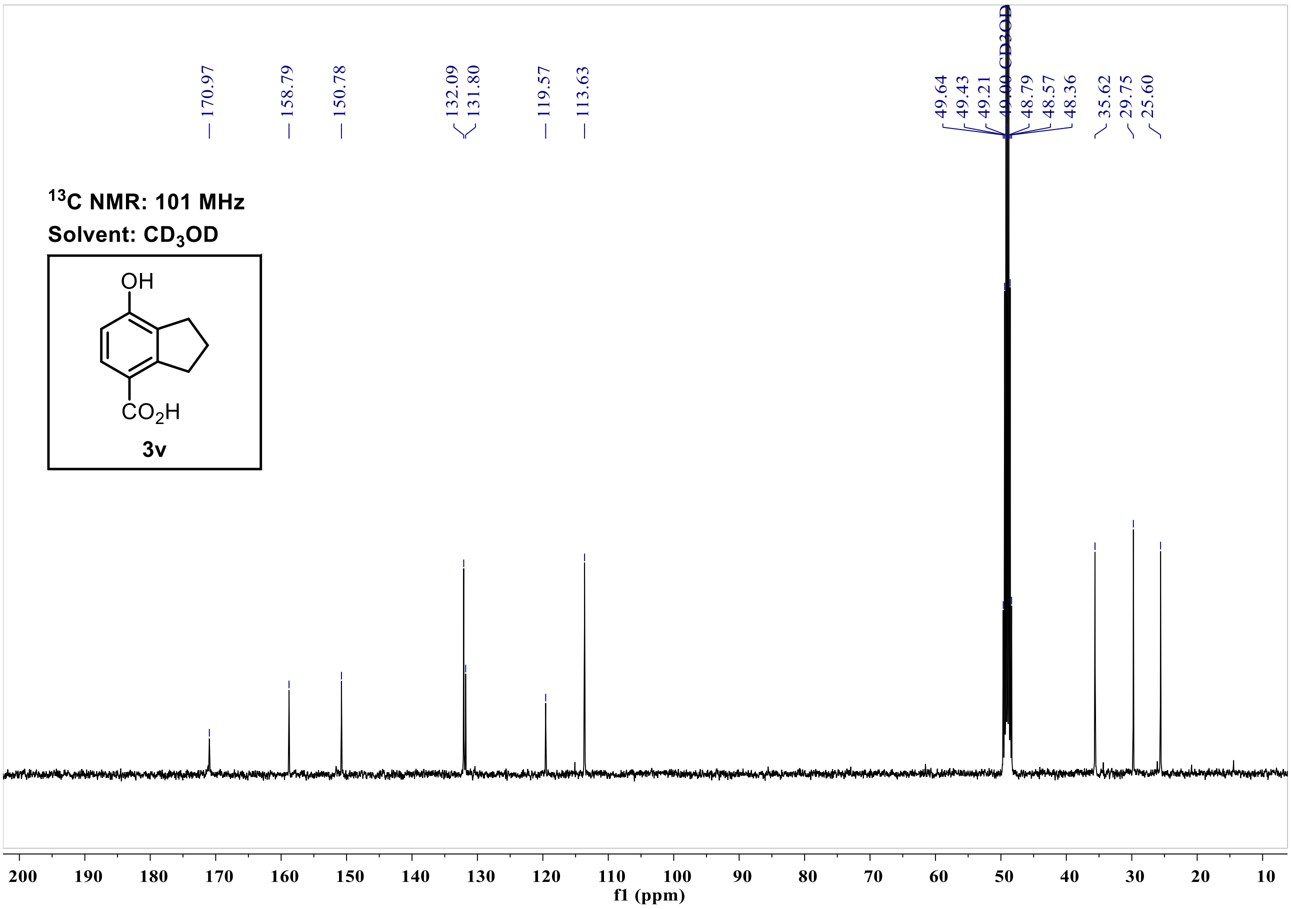

13C NMR: 101 MHz
Solvent: CD3OD
OH
CO2H
3v
170.97
158.79
150.78
132.09
131.80
119.57
113.63
49.64
49.43
49.21
49.00 CD3OD
48.79
48.57
48.36
35.62
29.75
25.60
200 190 180 170 160 150 140 130 120 110 100 90 80 70 60 50 40 30 20 10
f1 (ppm)

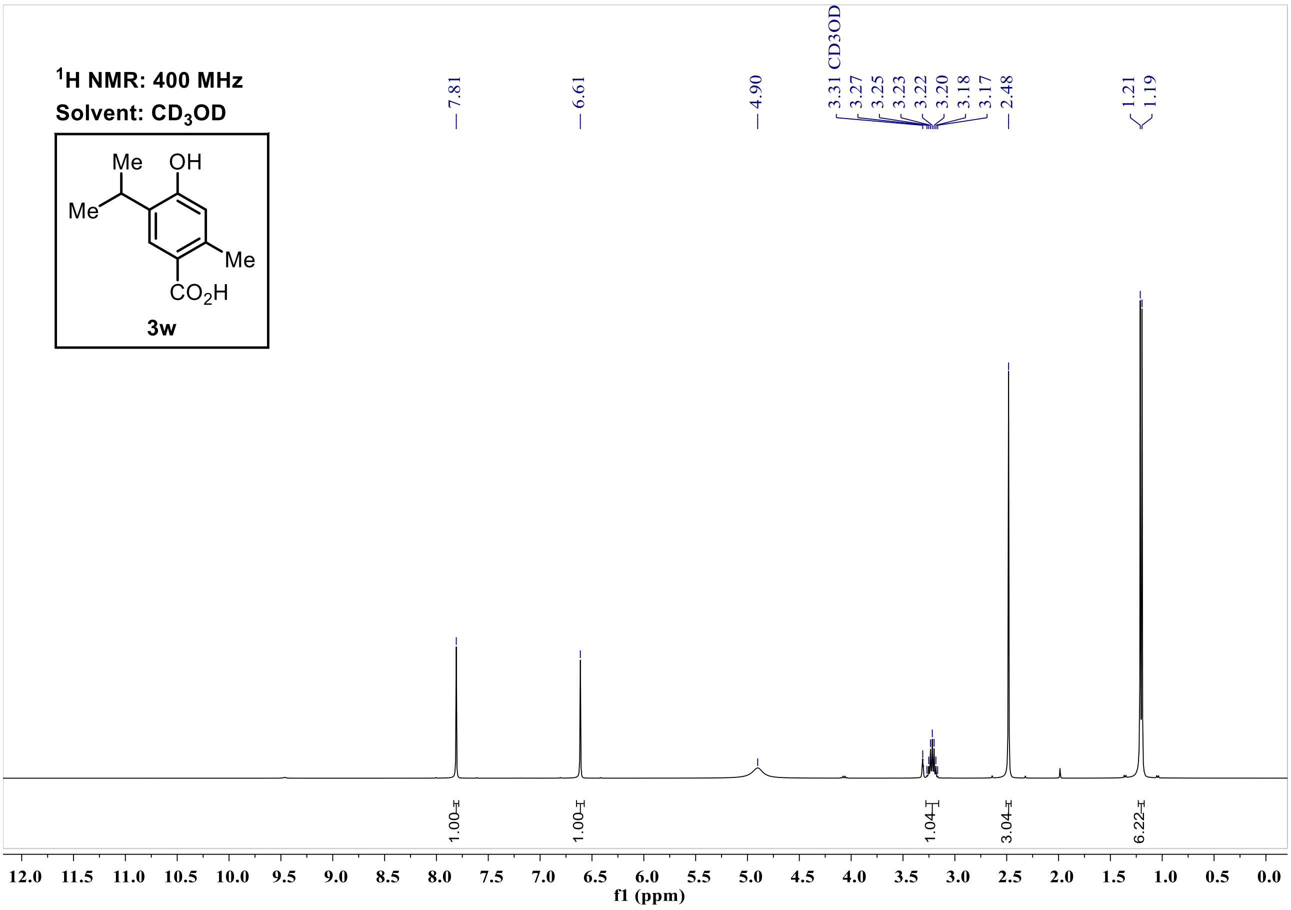

1H NMR: 400 MHz
Solvent: CD3OD
Me
OH
Me
Me
CO2H
3w
7.81
6.61
4.90
3.31 CD3OD
3.27
3.25
3.23
3.22
3.20
3.18
3.17
2.48
1.21
1.19
1.00
1.00
1.04
3.04
6.22
12.0 11.5 11.0 10.5 10.0 9.5 9.0 8.5 8.0 7.5 7.0 6.5 6.0 5.5 5.0 4.5 4.0 3.5 3.0 2.5 2.0 1.5 1.0 0.5 0.0
f1 (ppm)

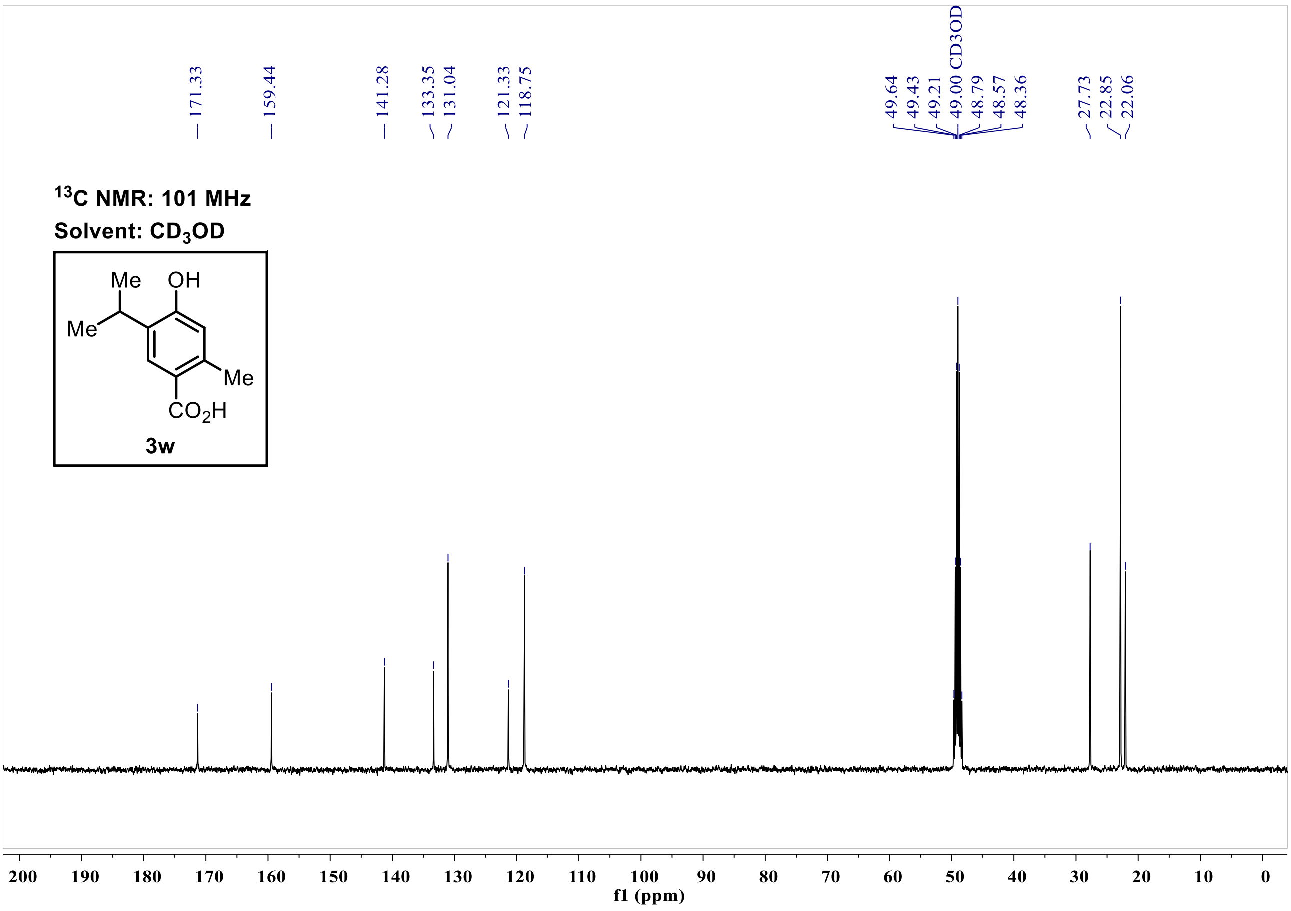

13C NMR: 101 MHz
Solvent: CD3OD
Me
OH
Me
Me
CO2H
3w
171.33
159.44
141.28
133.35
131.04
121.33
118.75
49.64
49.43
49.21
49.00 CD3OD
48.79
48.57
48.36
27.73
22.85
22.06
200 190 180 170 160 150 140 130 120 110 100 90 80 70 60 50 40 30 20 10 0
f1 (ppm)

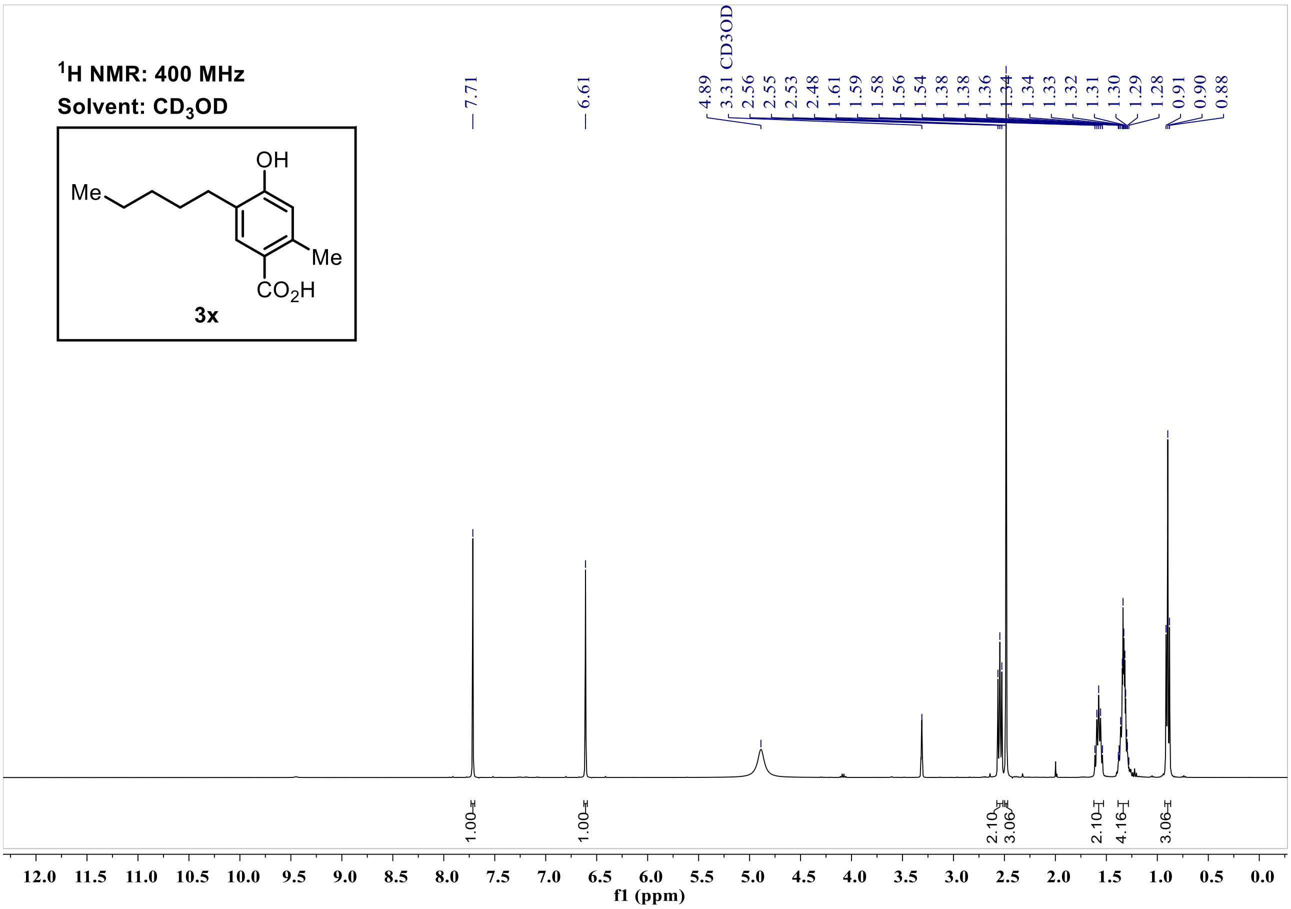
1H NMR: 400 MHz
Solvent: CD3OD
OH
Me
Me
CO2H
3x
7.71
6.61
4.89
3.31 CD3OD
2.56
2.55
2.53
2.48
1.61
1.59
1.58
1.56
1.54
1.38
1.38
1.36
1.34
1.34
1.33
1.32
1.31
1.30
1.29
1.28
0.91
0.90
0.88
1.00
1.00
2.10
3.06
2.10
4.16
3.06
12.0 11.5 11.0 10.5 10.0 9.5 9.0 8.5 8.0 7.5 7.0 6.5 6.0 5.5 5.0 4.5 4.0 3.5 3.0 2.5 2.0 1.5 1.0 0.5 0.0
f1 (ppm)

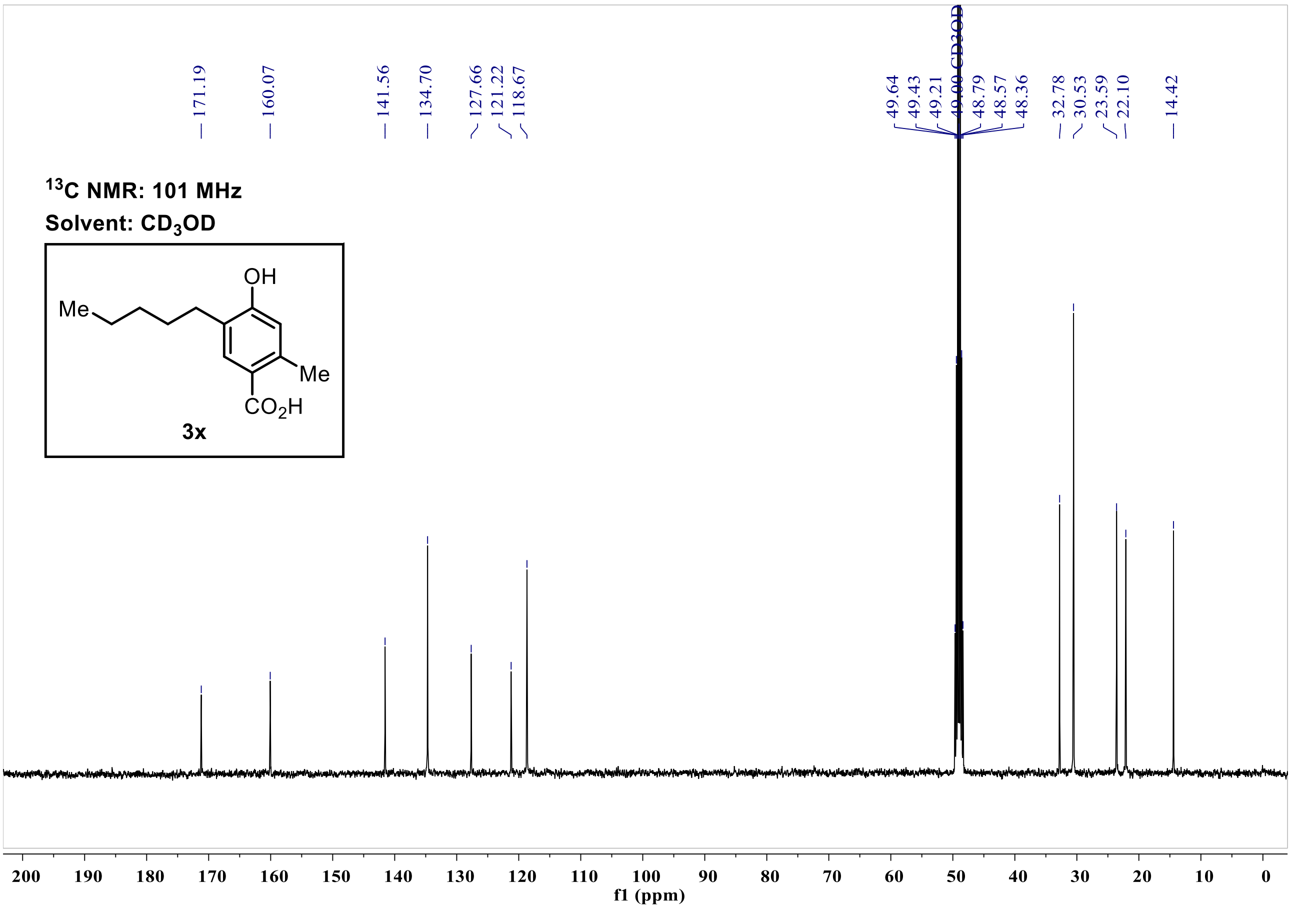
13C NMR: 101 MHz
Solvent: CD3OD
OH
Me
Me
CO2H
3x
171.19
160.07
141.56
134.70
127.66
121.22
118.67
49.64
49.43
49.21
49.00 CD3OD
48.79
48.57
48.36
32.78
30.53
23.59
22.10
14.42
200 190 180 170 160 150 140 130 120 110 100 90 80 70 60 50 40 30 20 10 0
f1 (ppm)

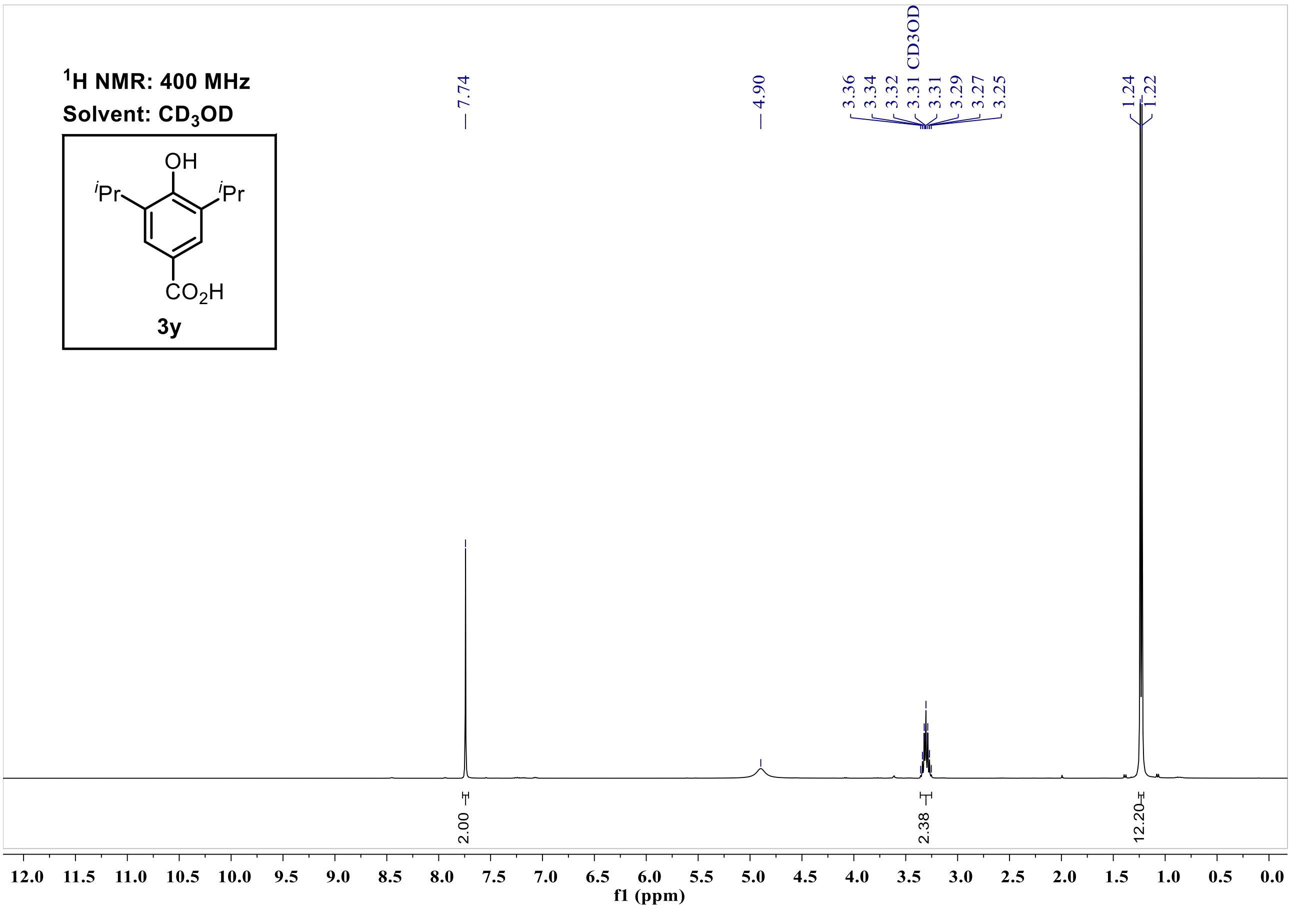

1H NMR: 400 MHz
Solvent: CD3OD
OH
iPr
iPr
CO2H
3y
7.74
4.90
3.36
3.34
3.32
3.31 CD3OD
3.31
3.29
3.27
3.25
1.24
1.22
2.00
2.38
12.20
12.0 11.5 11.0 10.5 10.0 9.5 9.0 8.5 8.0 7.5 7.0 6.5 6.0 5.5 5.0 4.5 4.0 3.5 3.0 2.5 2.0 1.5 1.0 0.5 0.0
f1 (ppm)

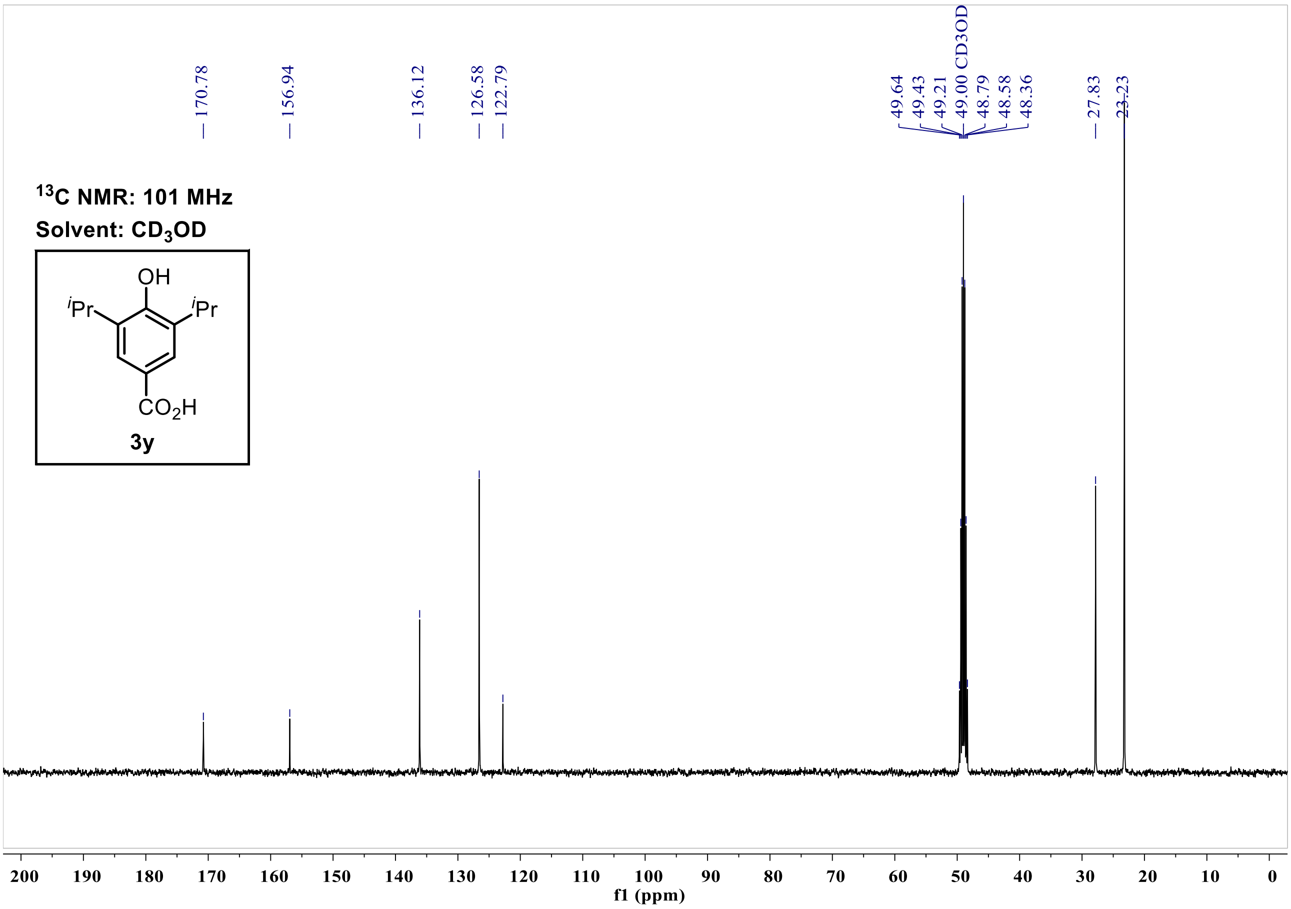

13C NMR: 101 MHz
Solvent: CD3OD
OH
iPr
iPr
CO2H
3y
170.78
156.94
136.12
126.58
122.79
49.64
49.43
49.21
49.00 CD3OD
48.79
48.58
48.36
27.83
23.23
200 190 180 170 160 150 140 130 120 110 100 90 80 70 60 50 40 30 20 10 0
f1 (ppm)

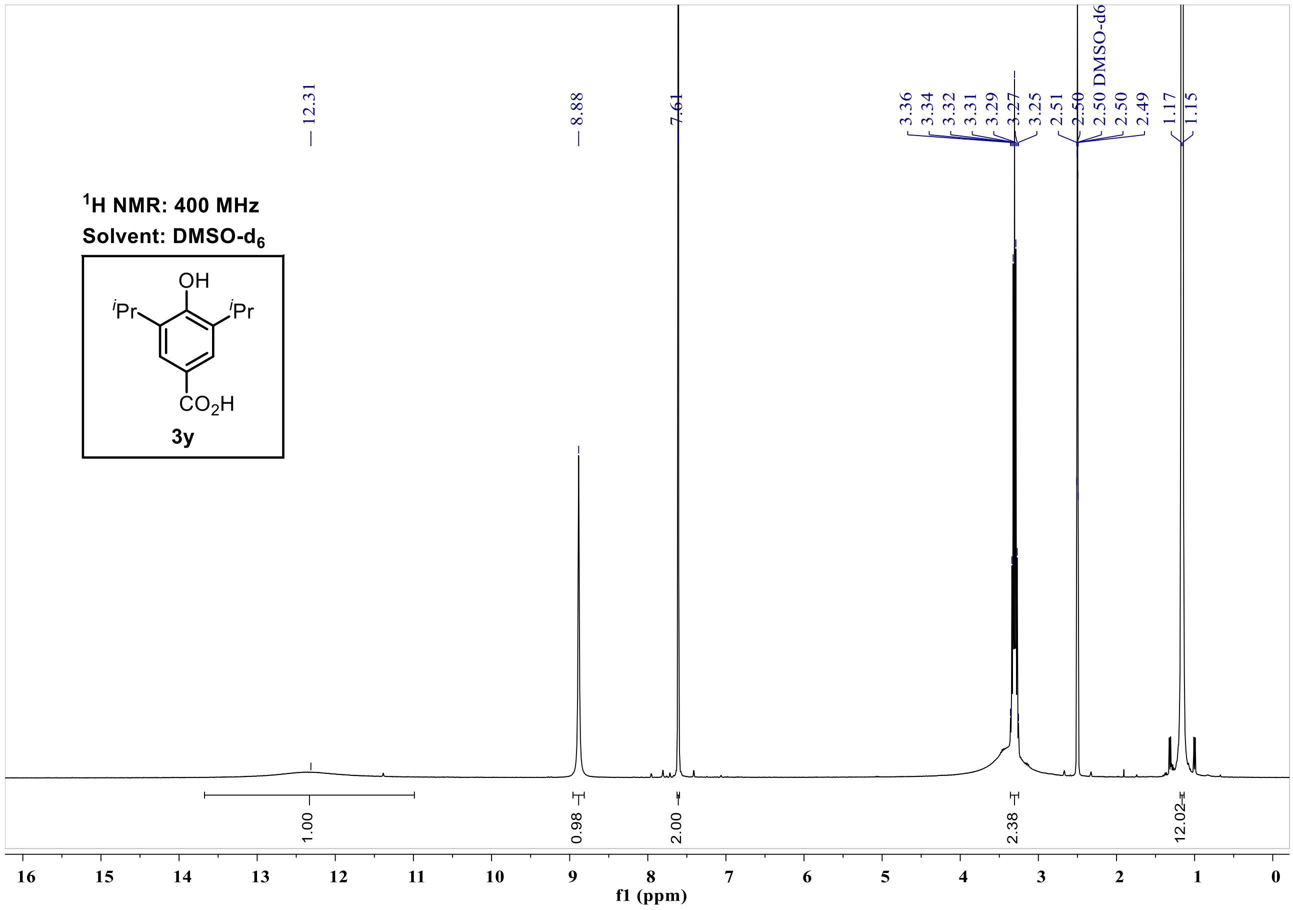
12.31
8.88
7.61
3.36
3.34
3.32
3.31
3.29
3.27
3.25
2.51
2.50
2.50 DMSO-d6
2.50
2.49
1.17
1.15
1H NMR: 400 MHz
Solvent: DMSO-d6
OH
iPr
iPr
CO2H
3y
1.00
0.98
2.00
2.38
12.02
16
15
14
13
12
11
10
9
8
7
6
5
4
3
2
1
0
f1 (ppm)

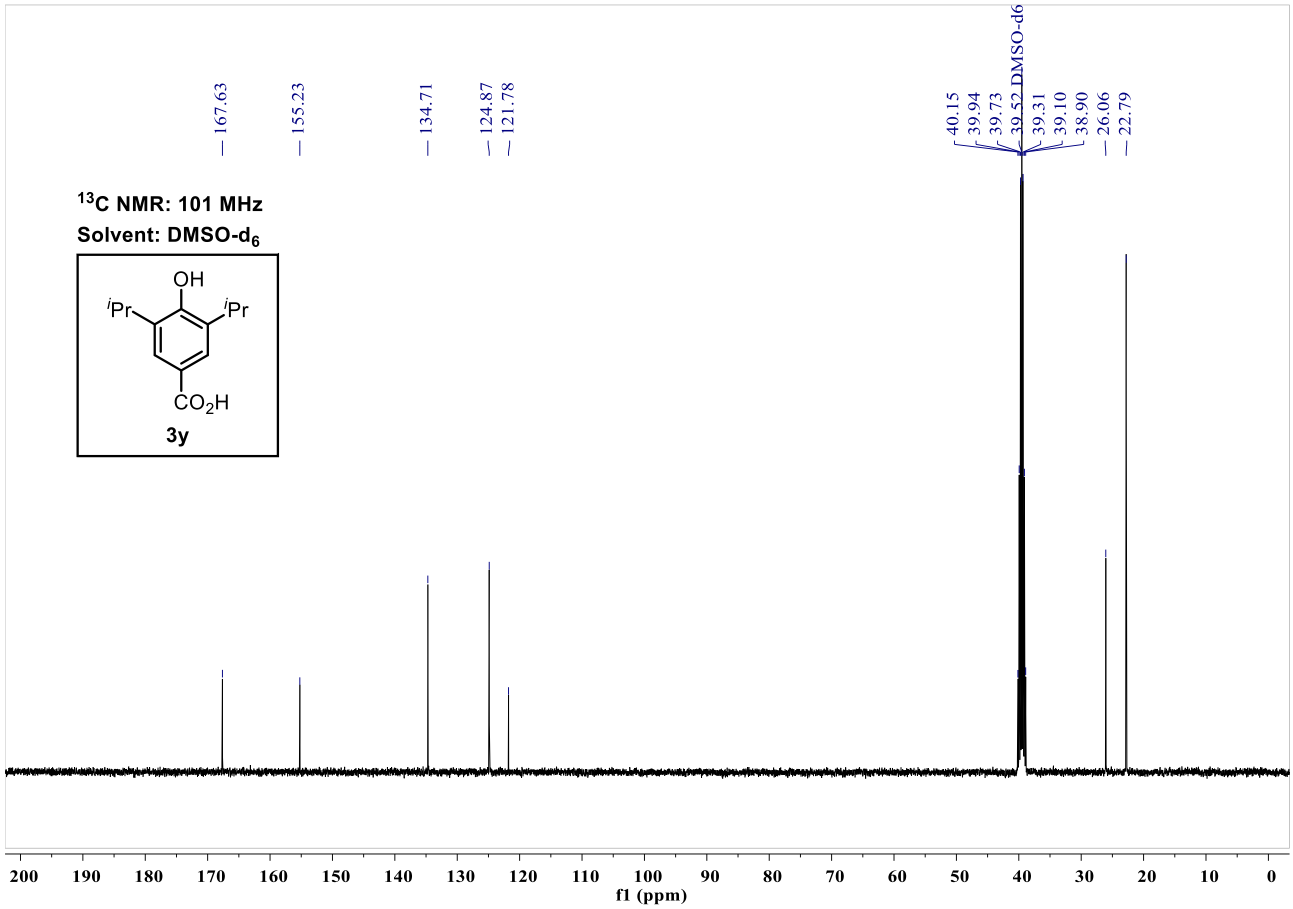
167.63
155.23
134.71
124.87
121.78
40.15
39.94
39.73
39.52 DMSO-d6
39.31
39.10
38.90
26.06
22.79
13C NMR: 101 MHz
Solvent: DMSO-d6
OH
iPr
iPr
CO2H
3y
200
190
180
170
160
150
140
130
120
110
100
90
80
70
60
50
40
30
20
10
0
f1 (ppm)

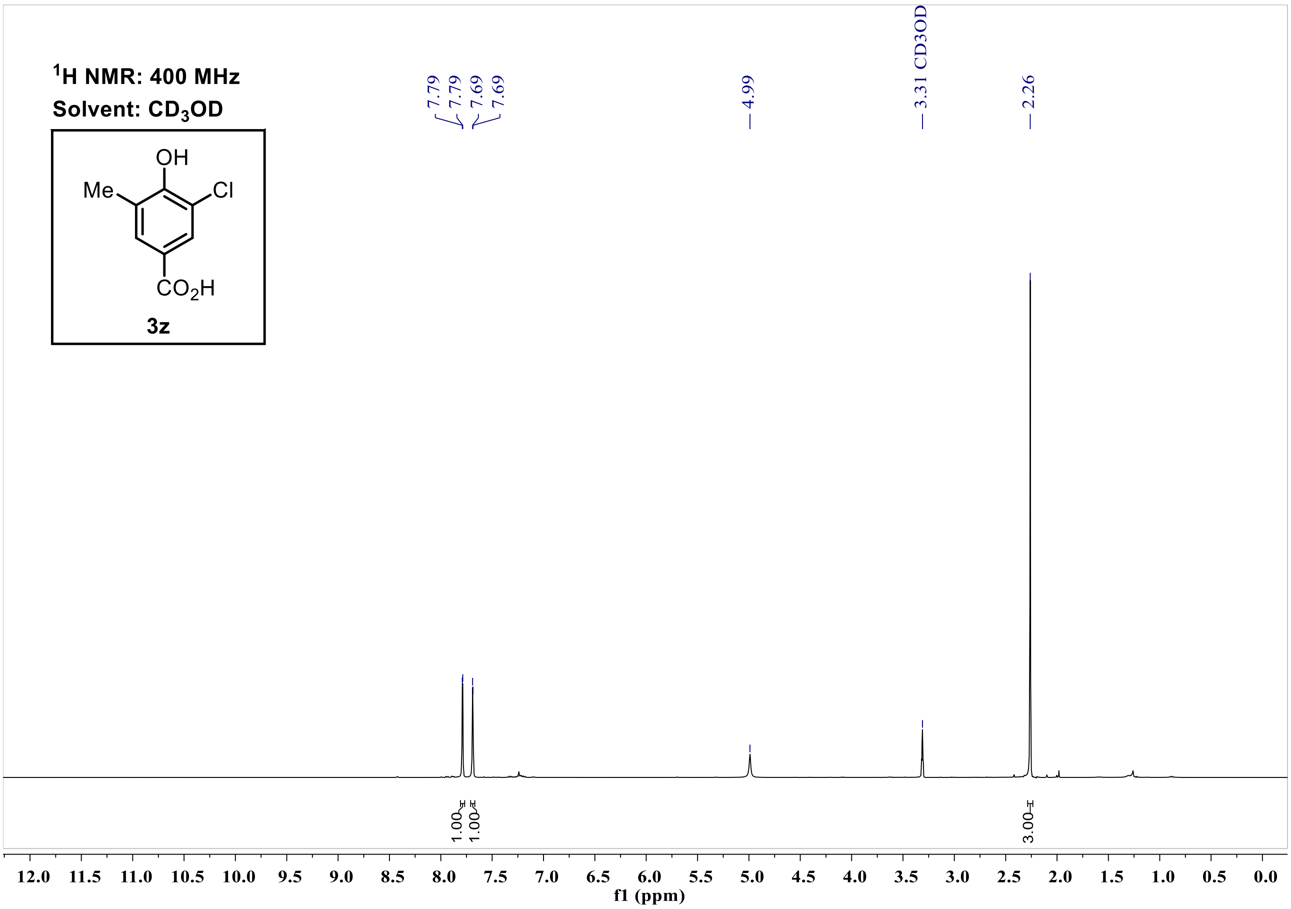

1H NMR: 400 MHz
Solvent: CD3OD
OH
Me
Cl
CO2H
3z
7.79
7.79
7.69
7.69
4.99
3.31 CD3OD
2.26
1.00
1.00
3.00
f1 (ppm)

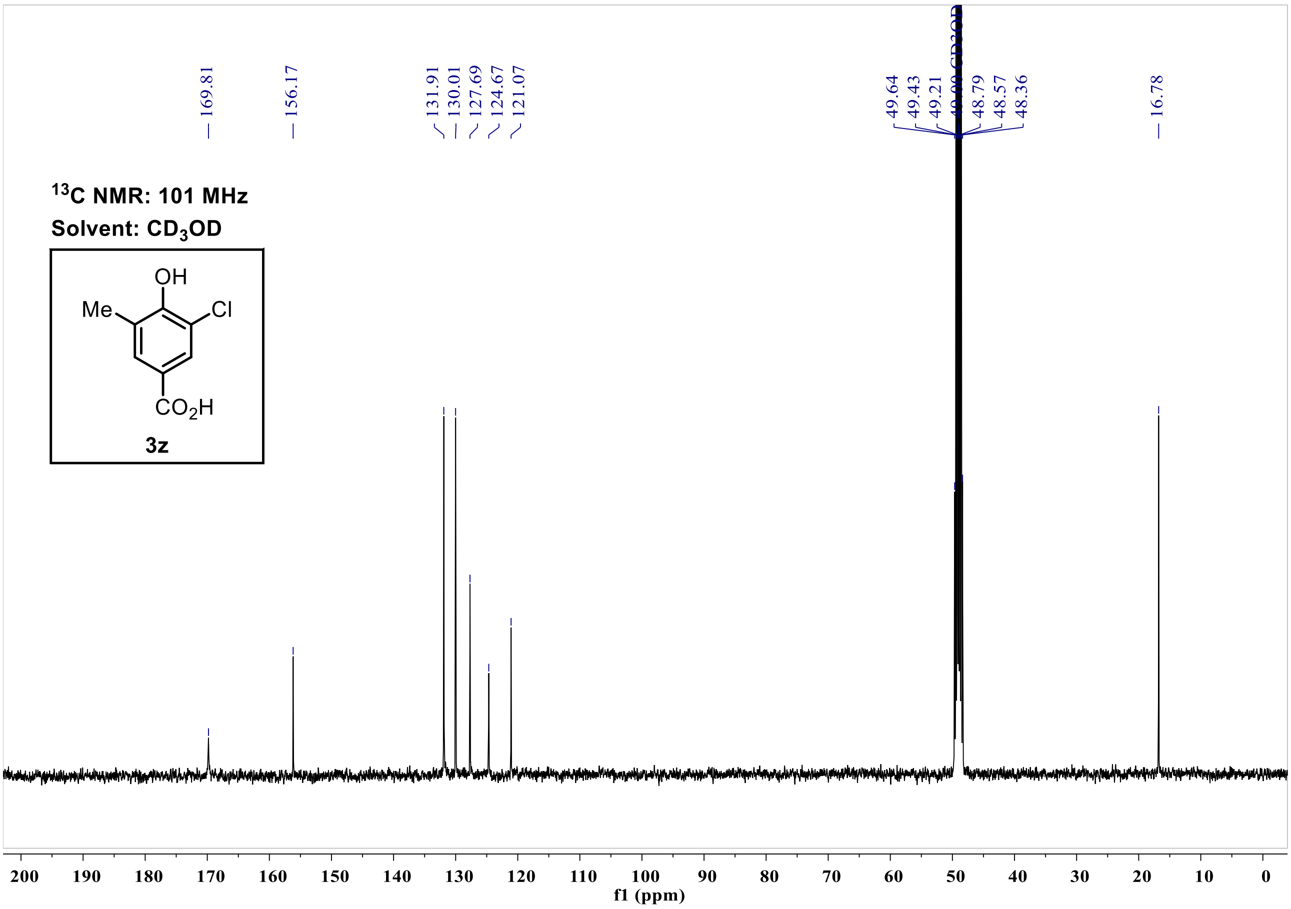

13C NMR: 101 MHz
Solvent: CD3OD
OH
Me
Cl
CO2H
3z
169.81
156.17
131.91
130.01
127.69
124.67
121.07
49.64
49.43
49.21
48.79
48.57
48.36
16.78
f1 (ppm)

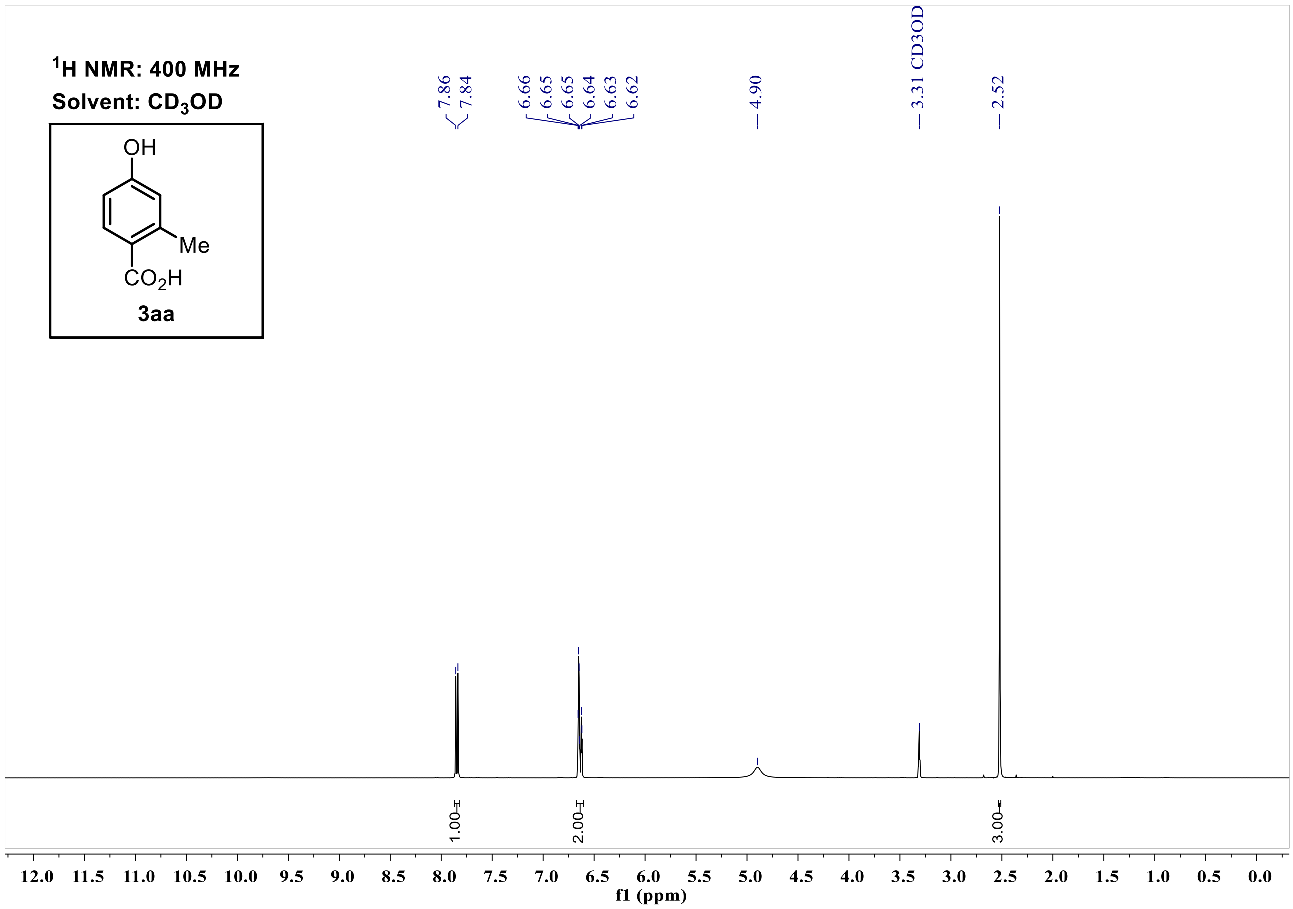

1H NMR: 400 MHz
Solvent: CD3OD
OH
Me
CO2H
3aa
7.86
7.84
6.66
6.65
6.65
6.64
6.63
6.62
4.90
3.31 CD3OD
2.52
1.00
2.00
3.00
12.0 11.5 11.0 10.5 10.0 9.5 9.0 8.5 8.0 7.5 7.0 6.5 6.0 5.5 5.0 4.5 4.0 3.5 3.0 2.5 2.0 1.5 1.0 0.5 0.0
f1 (ppm)

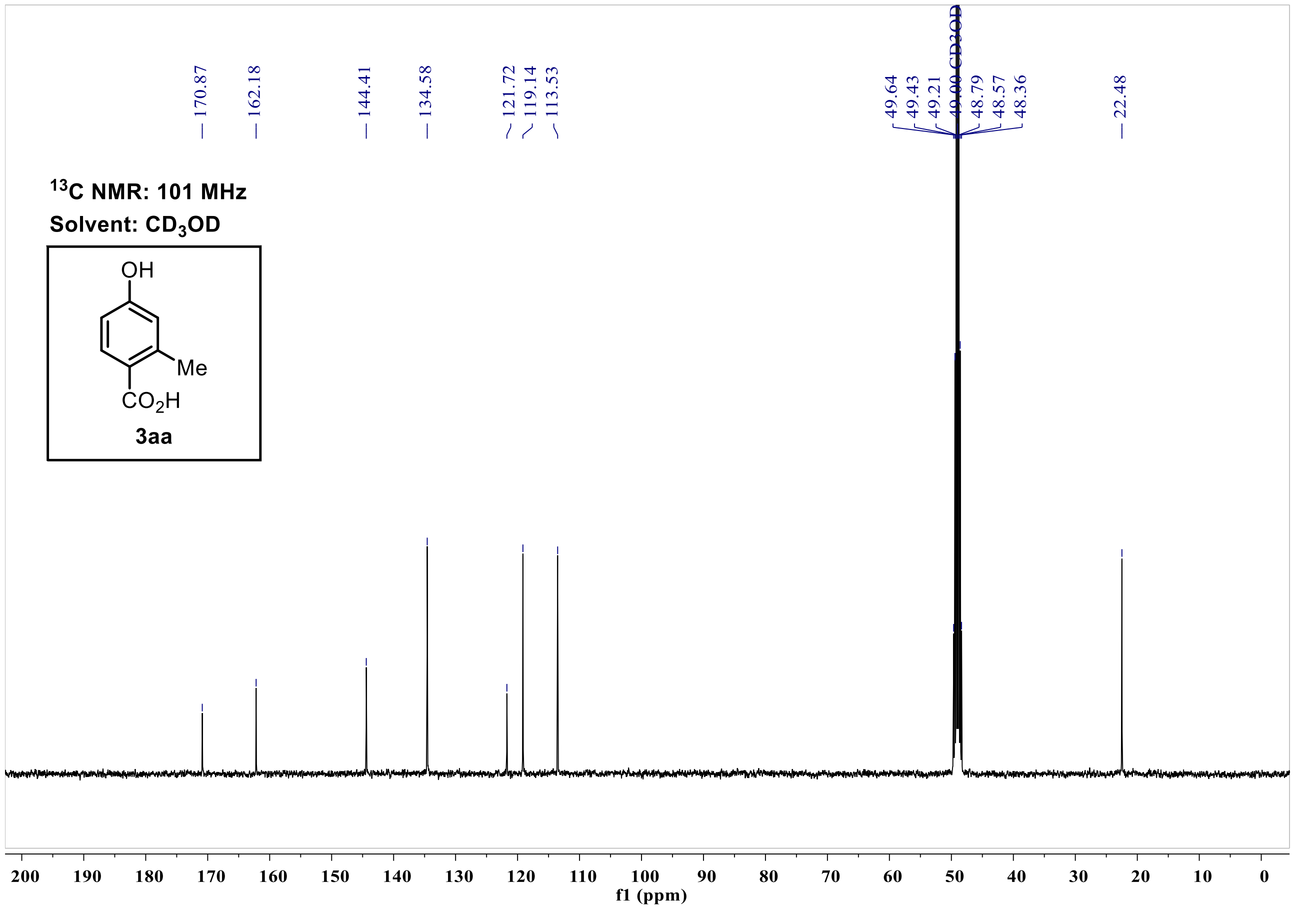

13C NMR: 101 MHz
Solvent: CD3OD
OH
Me
CO2H
3aa
170.87
162.18
144.41
134.58
121.72
119.14
113.53
49.64
49.43
49.21
49.00 CD3OD
48.79
48.57
48.36
22.48
200 190 180 170 160 150 140 130 120 110 100 90 80 70 60 50 40 30 20 10 0
f1 (ppm)

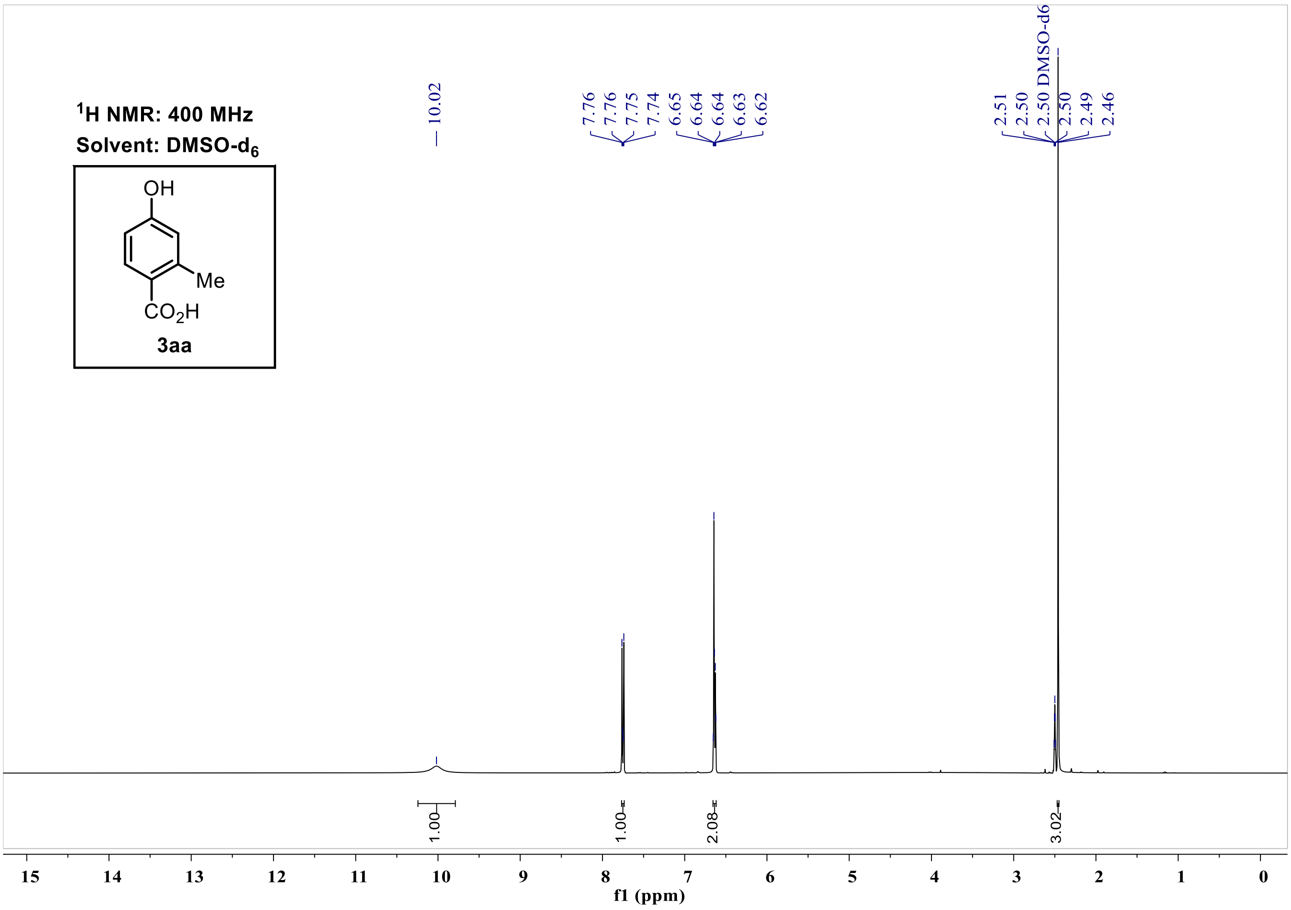

1H NMR: 400 MHz
Solvent: DMSO-d6
OH
Me
CO2H
3aa
10.02
7.76
7.76
7.75
7.74
6.65
6.64
6.64
6.63
6.62
2.51
2.50
2.50 DMSO-d6
2.50
2.49
2.46
1.00
1.00
2.08
3.02
f1 (ppm)

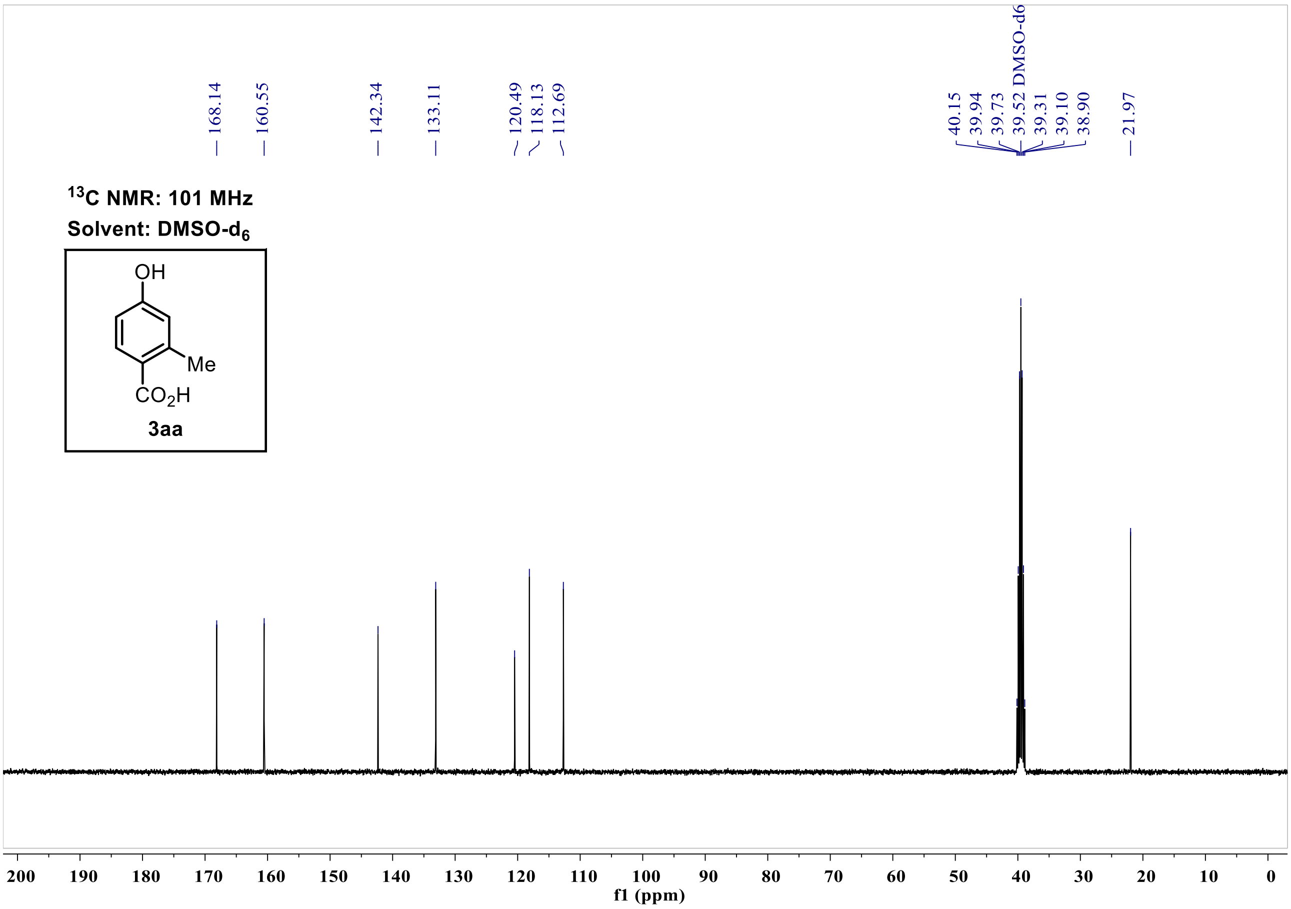

13C NMR: 101 MHz
Solvent: DMSO-d6
OH
Me
CO2H
3aa
168.14
160.55
142.34
133.11
120.49
118.13
112.69
40.15
39.94
39.73
39.52 DMSO-d6
39.31
39.10
38.90
21.97
f1 (ppm)

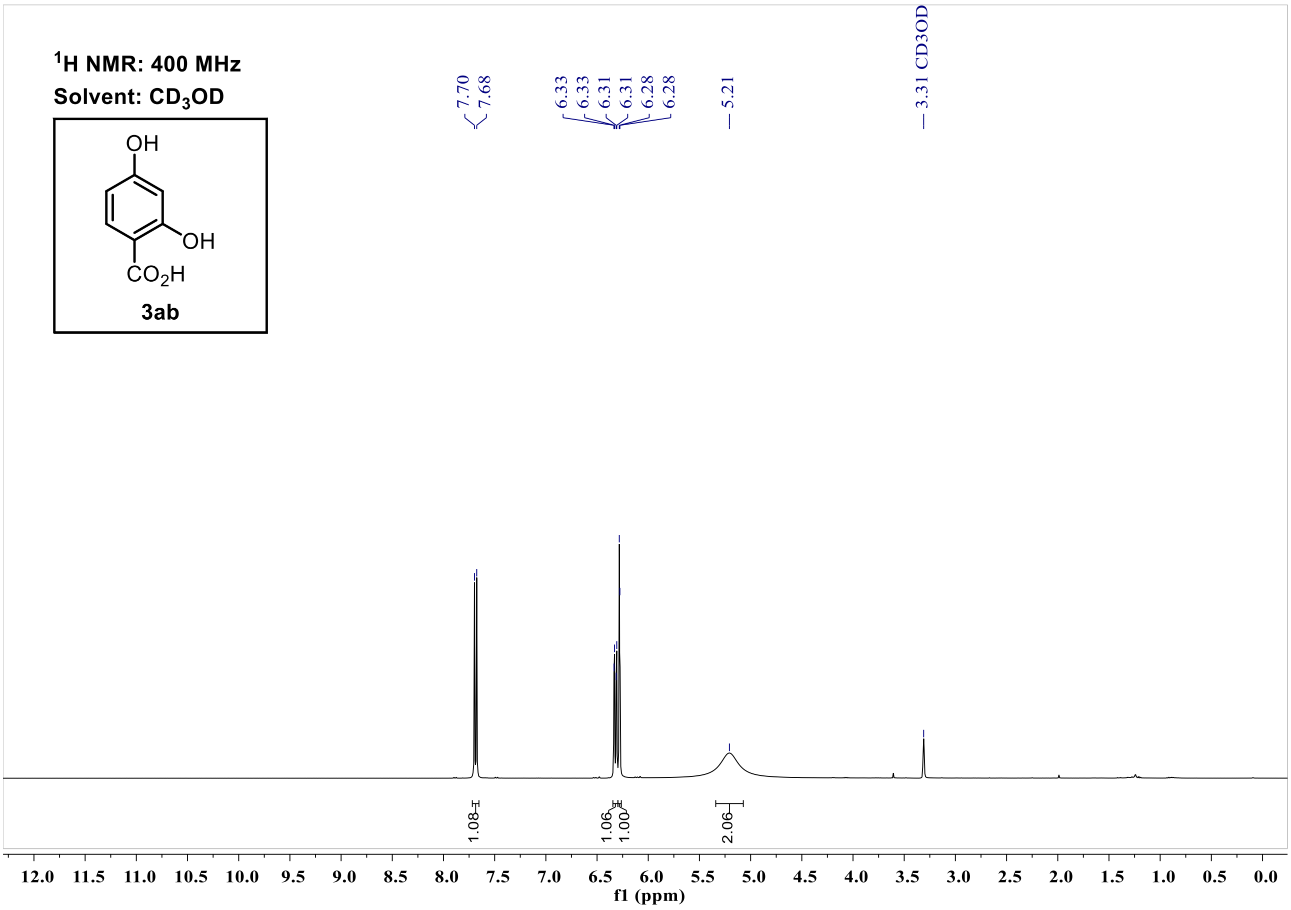

1H NMR: 400 MHz
Solvent: CD3OD
3ab
7.70
7.68
6.33
6.33
6.31
6.31
6.28
6.28
5.21
3.31 CD3OD
1.08
1.06
1.00
2.06
f1 (ppm)

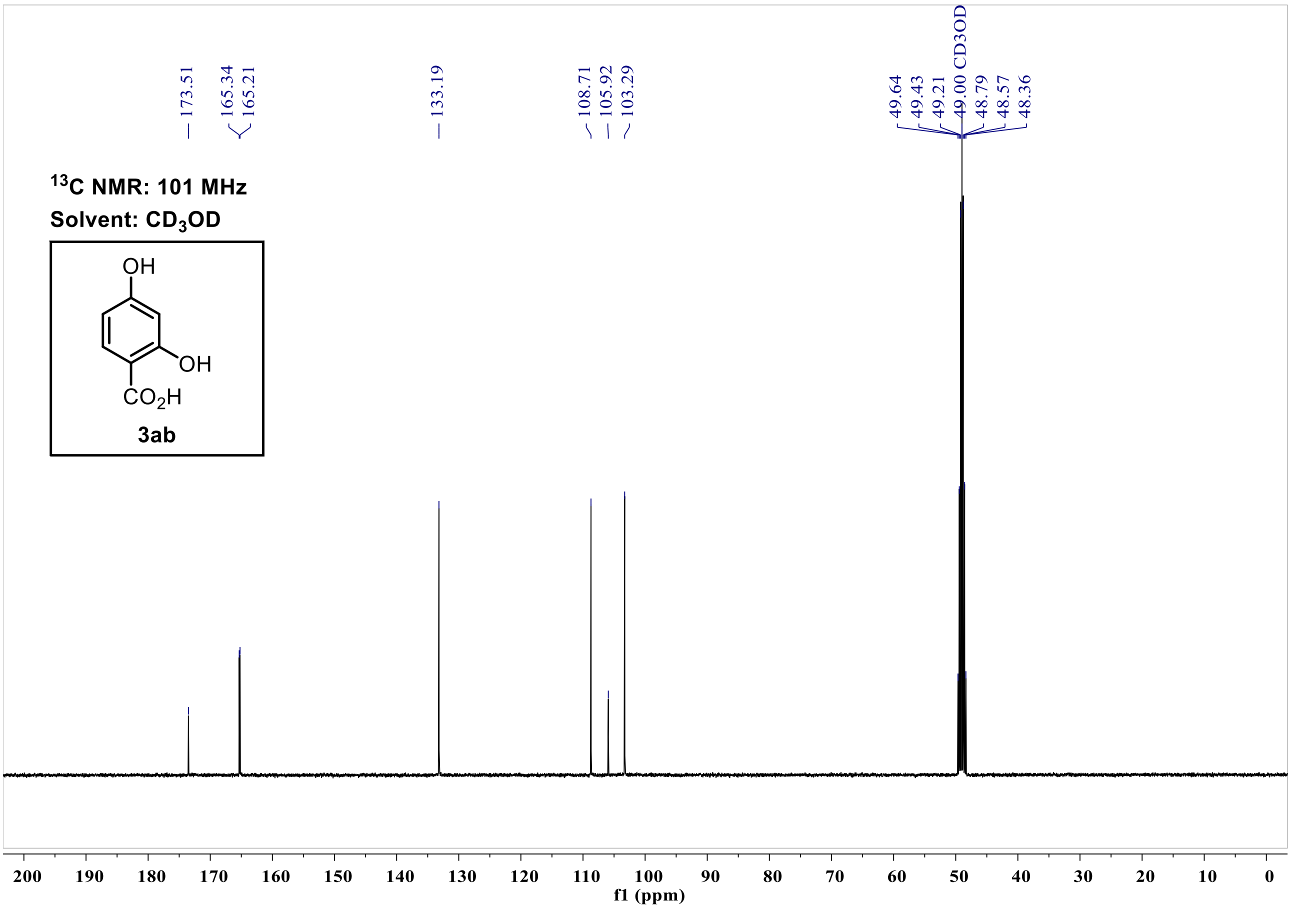

13C NMR: 101 MHz
Solvent: CD3OD
3ab
173.51
165.34
165.21
133.19
108.71
105.92
103.29
49.64
49.43
49.21
49.00 CD3OD
48.79
48.57
48.36
f1 (ppm)

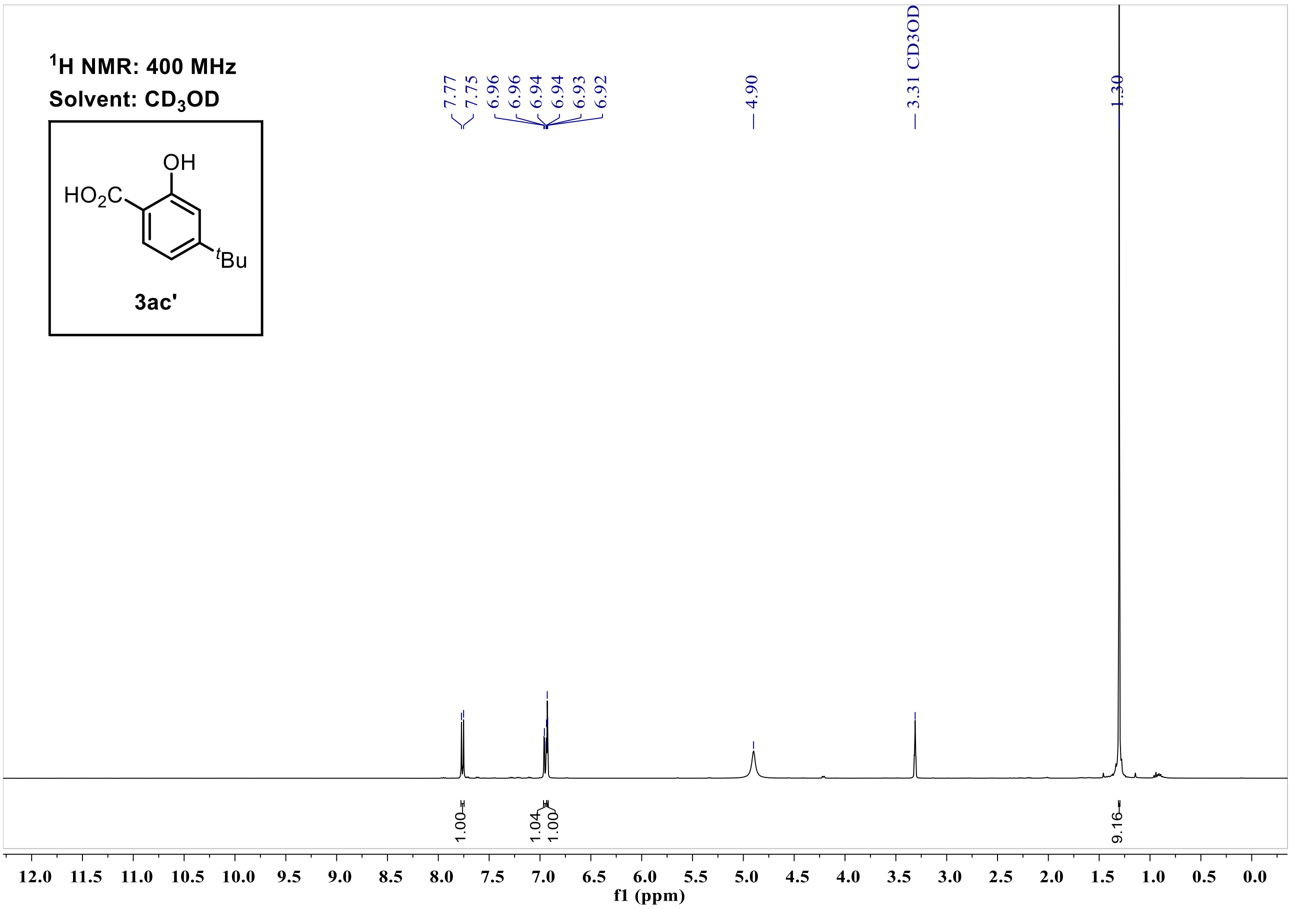
1H NMR: 400 MHz
Solvent: CD3OD
OH
HO2C
tBu
3ac'
7.77
7.75
6.96
6.96
6.94
6.94
6.93
6.92
4.90
3.31 CD3OD
1.30
1.00
1.04
1.00
9.16
12.0 11.5 11.0 10.5 10.0 9.5 9.0 8.5 8.0 7.5 7.0 6.5 6.0 5.5 5.0 4.5 4.0 3.5 3.0 2.5 2.0 1.5 1.0 0.5 0.0
f1 (ppm)

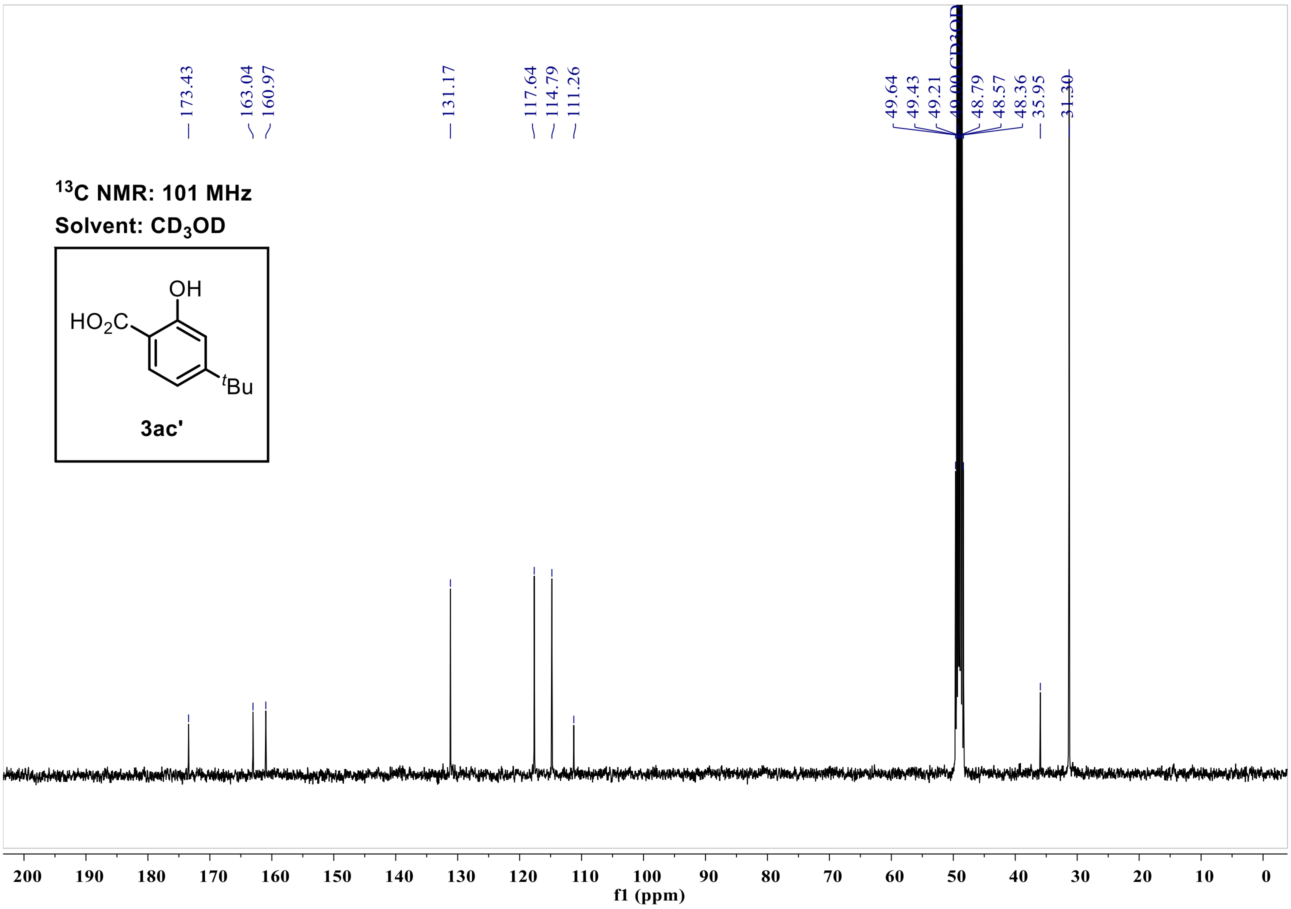
13C NMR: 101 MHz
Solvent: CD3OD
OH
HO2C
tBu
3ac'
173.43
163.04
160.97
131.17
117.64
114.79
111.26
49.64
49.43
49.21
48.79
48.57
48.36
35.95
31.30
200 190 180 170 160 150 140 130 120 110 100 90 80 70 60 50 40 30 20 10 0
f1 (ppm)

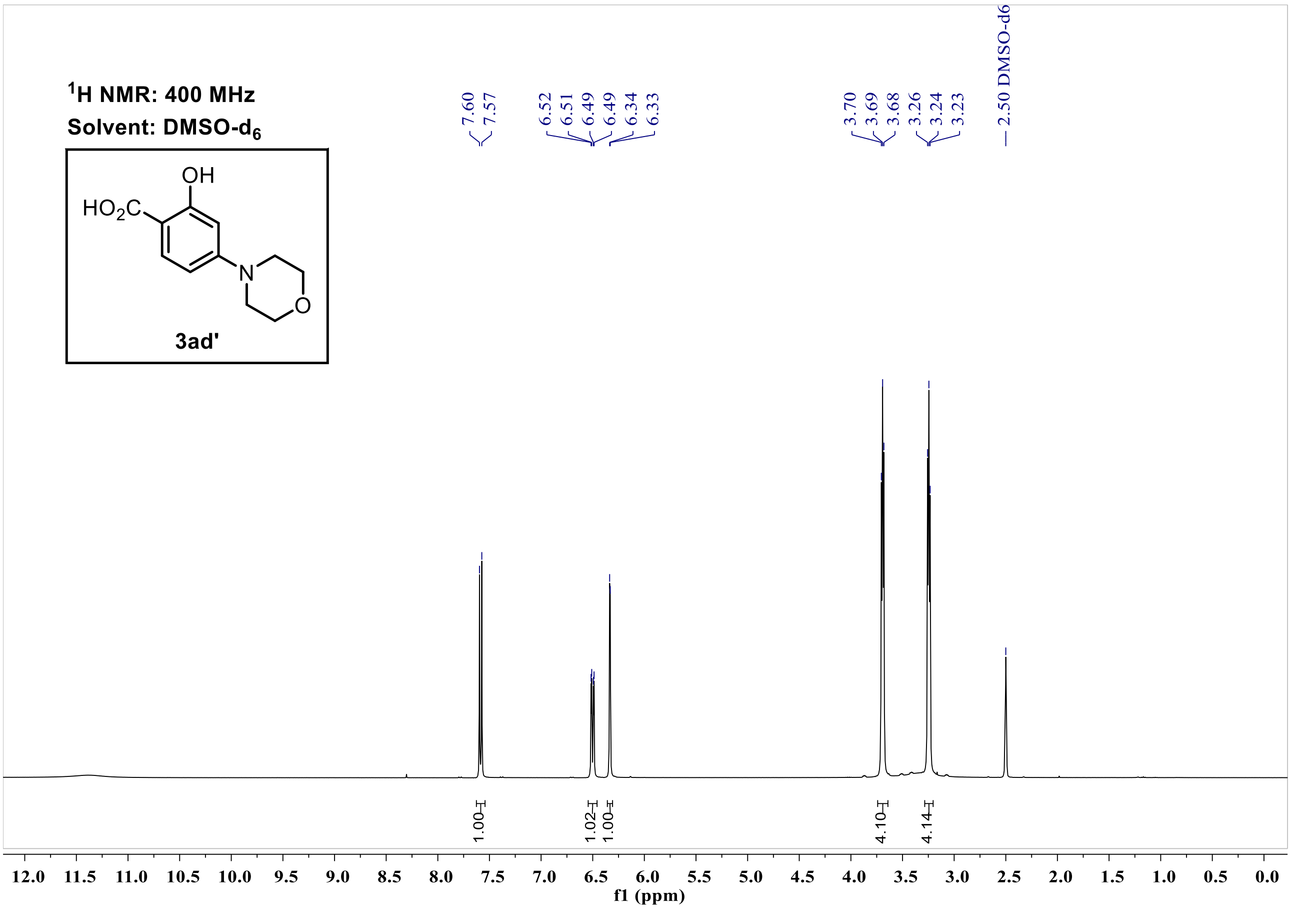

^{1}H NMR: 400 MHz
Solvent: DMSO-d_6
OH
HO_2C
N
O
3ad'
7.60
7.57
6.52
6.51
6.49
6.49
6.34
6.33
3.70
3.69
3.68
3.26
3.24
3.23
2.50 DMSO-d6
1.00
1.02
1.00
4.10
4.14
12.0 11.5 11.0 10.5 10.0 9.5 9.0 8.5 8.0 7.5 7.0 6.5 6.0 5.5 5.0 4.5 4.0 3.5 3.0 2.5 2.0 1.5 1.0 0.5 0.0
f1 (ppm)

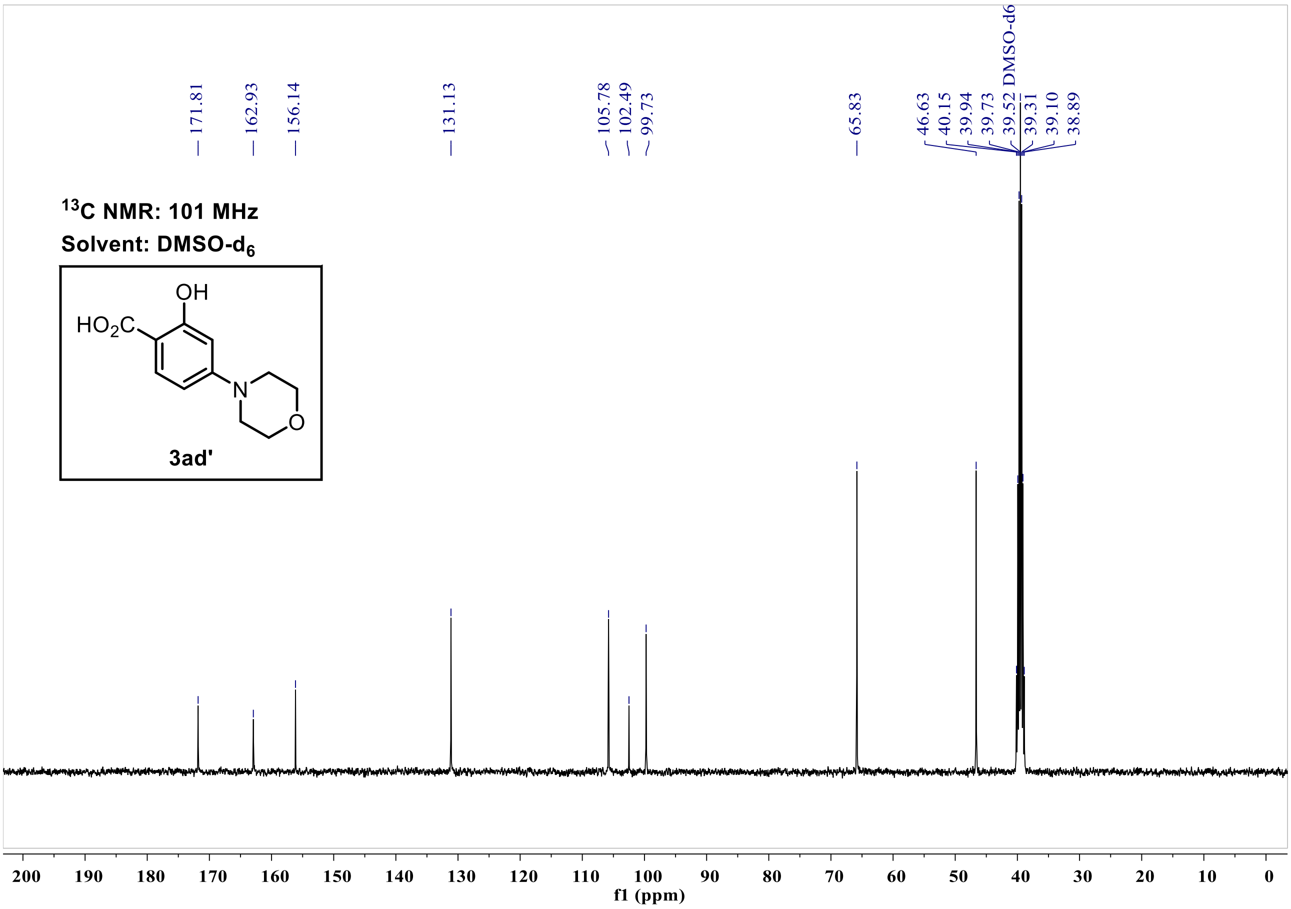

^{13}C NMR: 101 MHz
Solvent: DMSO-d_6
OH
HO_2C
N
O
3ad'
171.81
162.93
156.14
131.13
105.78
102.49
99.73
65.83
46.63
40.15
39.94
39.73
39.52 DMSO-d6
39.31
39.10
38.89
200 190 180 170 160 150 140 130 120 110 100 90 80 70 60 50 40 30 20 10 0
f1 (ppm)

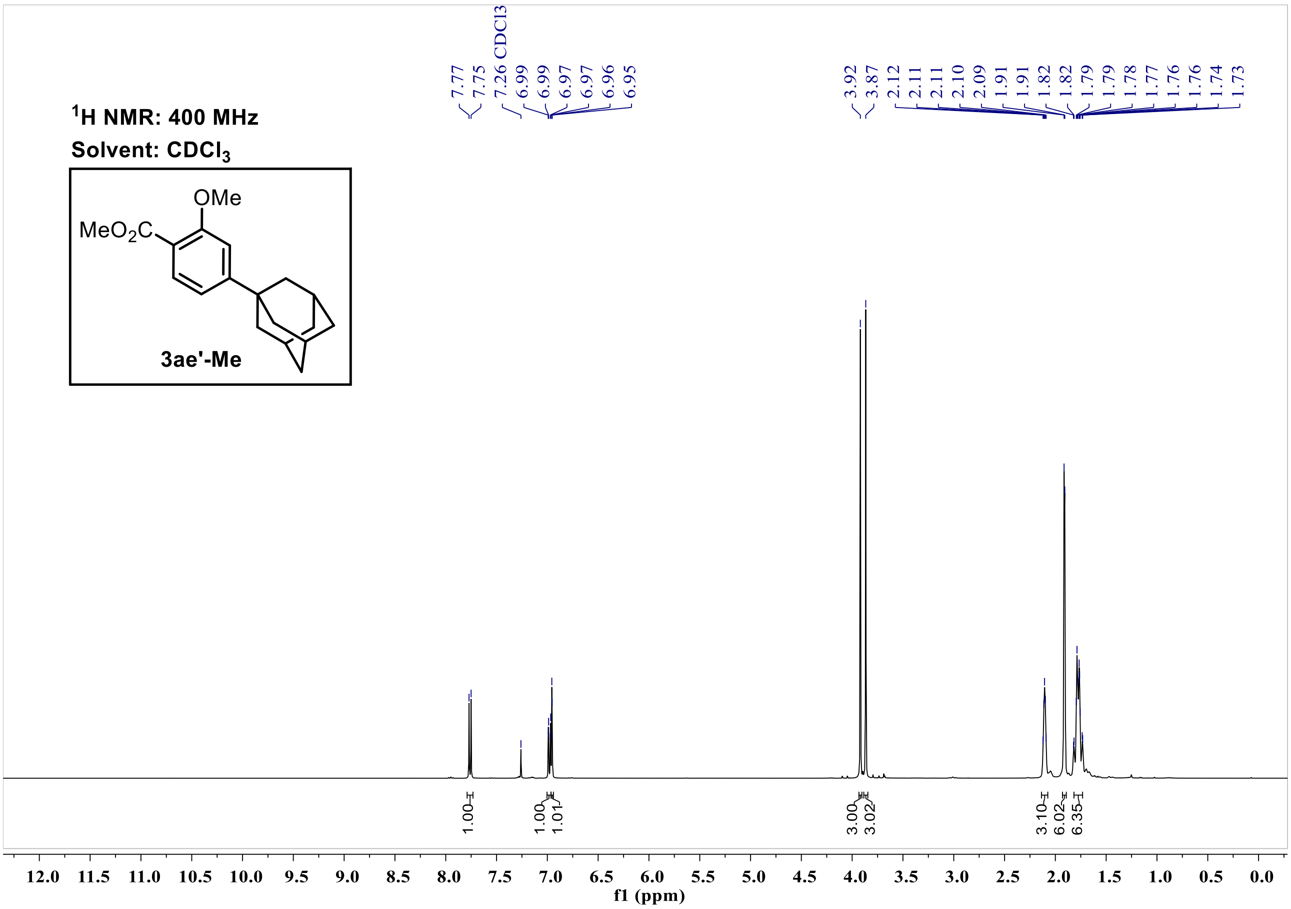

7.77
7.75
7.26 CDCl3
6.99
6.99
6.97
6.97
6.96
6.95
3.92
3.87
2.12
2.11
2.11
2.10
2.09
1.91
1.91
1.82
1.82
1.79
1.79
1.78
1.77
1.76
1.76
1.74
1.73
1H NMR: 400 MHz
Solvent: CDCl3
OMe
MeO2C
3ae'-Me
1.00
1.00
1.01
3.00
3.02
3.10
6.02
6.35
12.0 11.5 11.0 10.5 10.0 9.5 9.0 8.5 8.0 7.5 7.0 6.5 6.0 5.5 5.0 4.5 4.0 3.5 3.0 2.5 2.0 1.5 1.0 0.5 0.0
f1 (ppm)

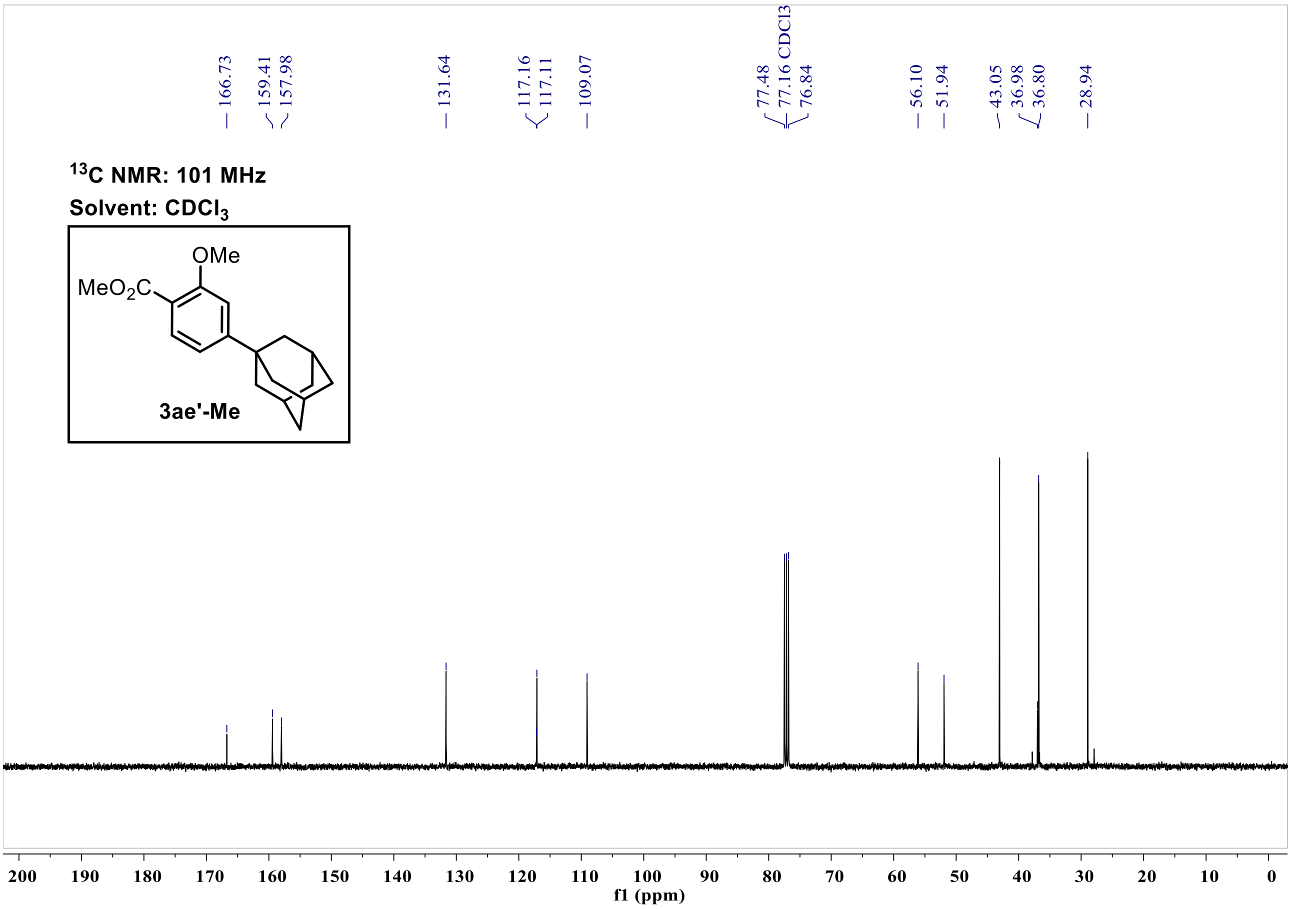

166.73
159.41
157.98
131.64
117.16
117.11
109.07
77.48
77.16 CDCl3
76.84
56.10
51.94
43.05
36.98
36.80
28.94
13C NMR: 101 MHz
Solvent: CDCl3
OMe
MeO2C
3ae'-Me
200 190 180 170 160 150 140 130 120 110 100 90 80 70 60 50 40 30 20 10 0
f1 (ppm)

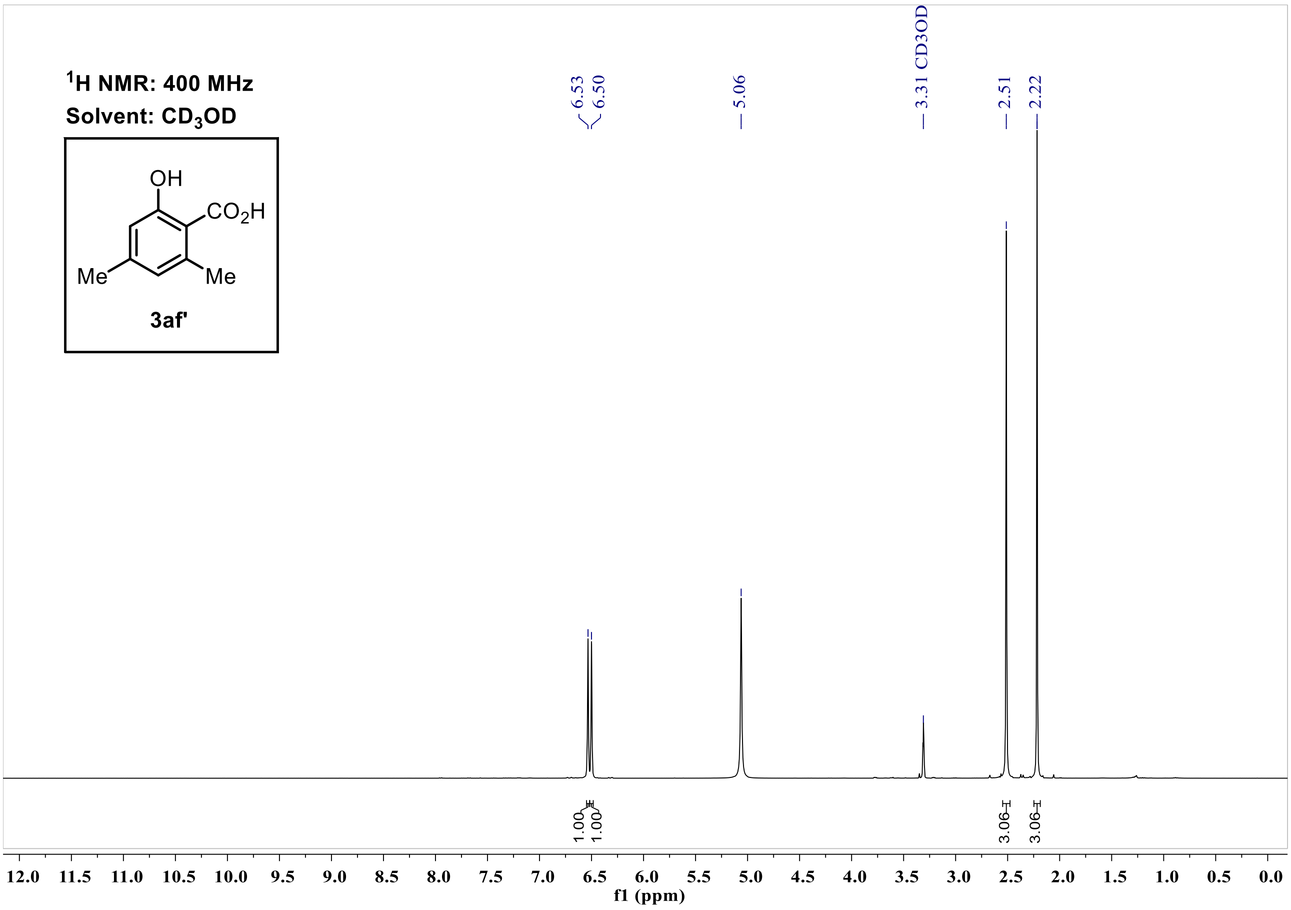

^{1}H NMR: 400 MHz
Solvent: CD_3OD
OH
CO_2H
Me
Me
3af'
6.53
6.50
5.06
3.31 CD3OD
2.51
2.22
1.00
1.00
3.06
3.06
12.0 11.5 11.0 10.5 10.0 9.5 9.0 8.5 8.0 7.5 7.0 6.5 6.0 5.5 5.0 4.5 4.0 3.5 3.0 2.5 2.0 1.5 1.0 0.5 0.0
f1 (ppm)

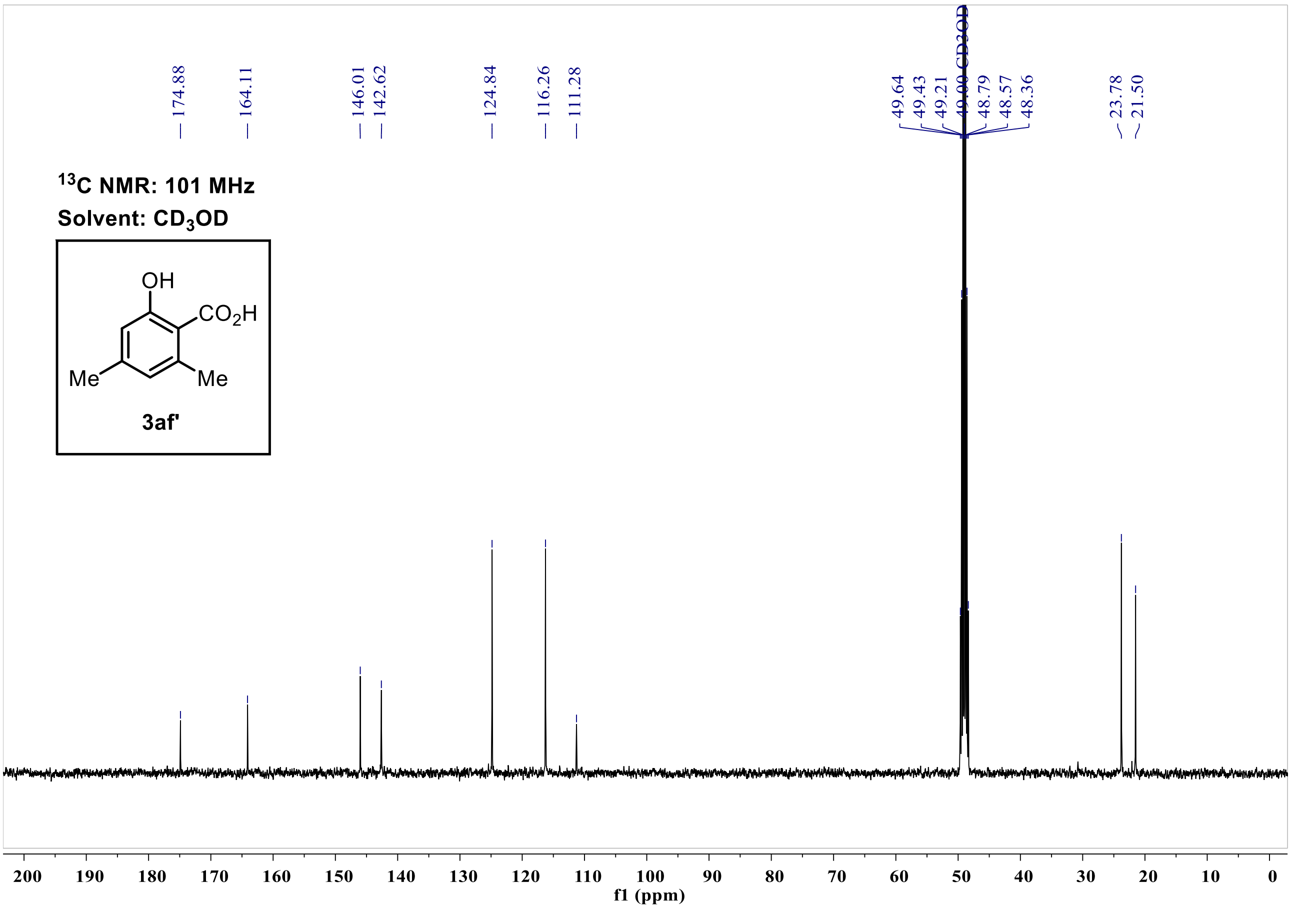

^{13}C NMR: 101 MHz
Solvent: CD_3OD
OH
CO_2H
Me
Me
3af'
174.88
164.11
146.01
142.62
124.84
116.26
111.28
49.64
49.43
49.21
49.00 CD3OD
48.79
48.57
48.36
23.78
21.50
200 190 180 170 160 150 140 130 120 110 100 90 80 70 60 50 40 30 20 10 0
f1 (ppm)

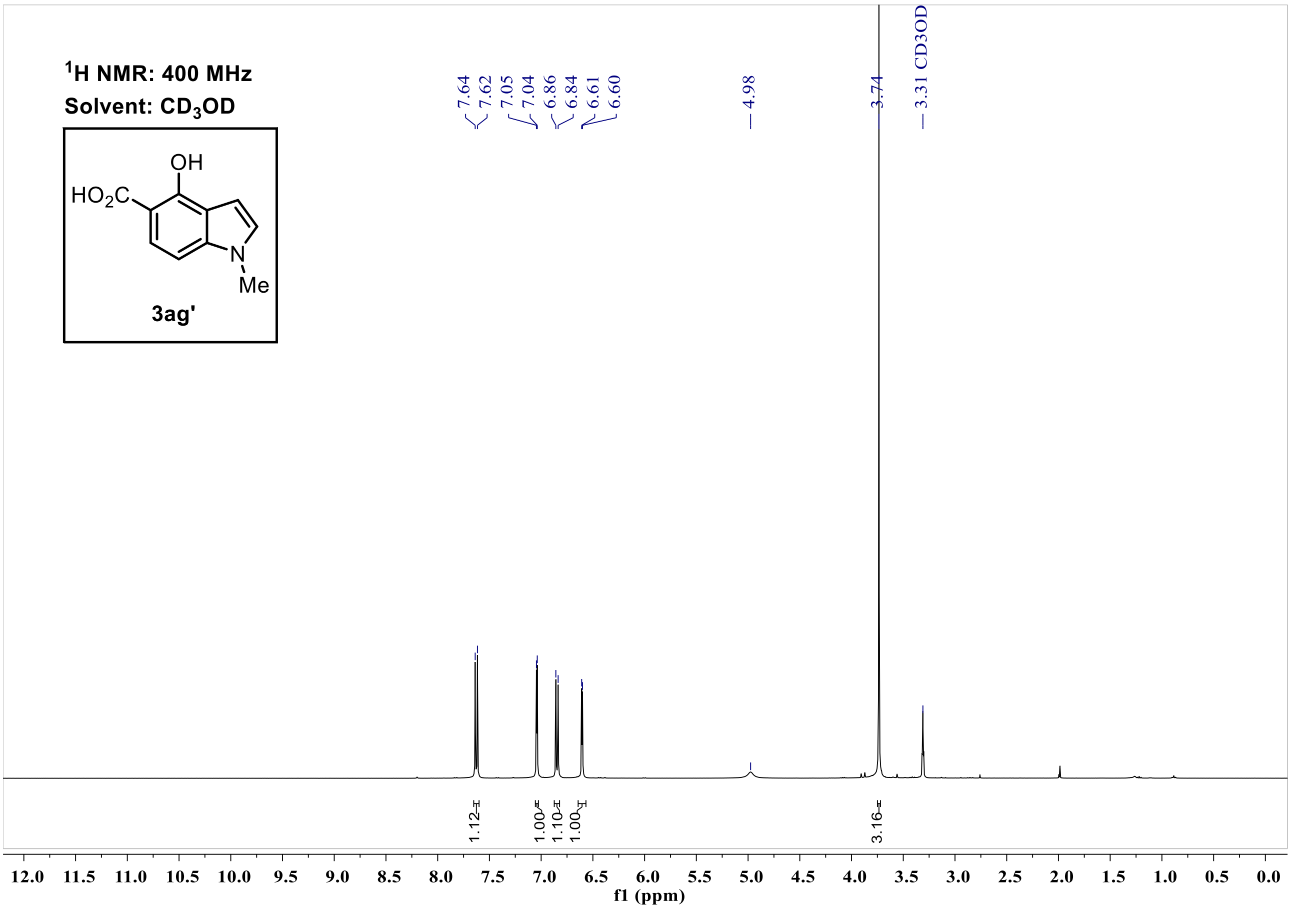

1H NMR: 400 MHz
Solvent: CD3OD
OH
HO2C
N
Me
3ag'
7.64
7.62
7.05
7.04
6.86
6.84
6.61
6.60
4.98
3.74
3.31 CD3OD
1.12
1.00
1.10
1.00
3.16
12.0 11.5 11.0 10.5 10.0 9.5 9.0 8.5 8.0 7.5 7.0 6.5 6.0 5.5 5.0 4.5 4.0 3.5 3.0 2.5 2.0 1.5 1.0 0.5 0.0
f1 (ppm)

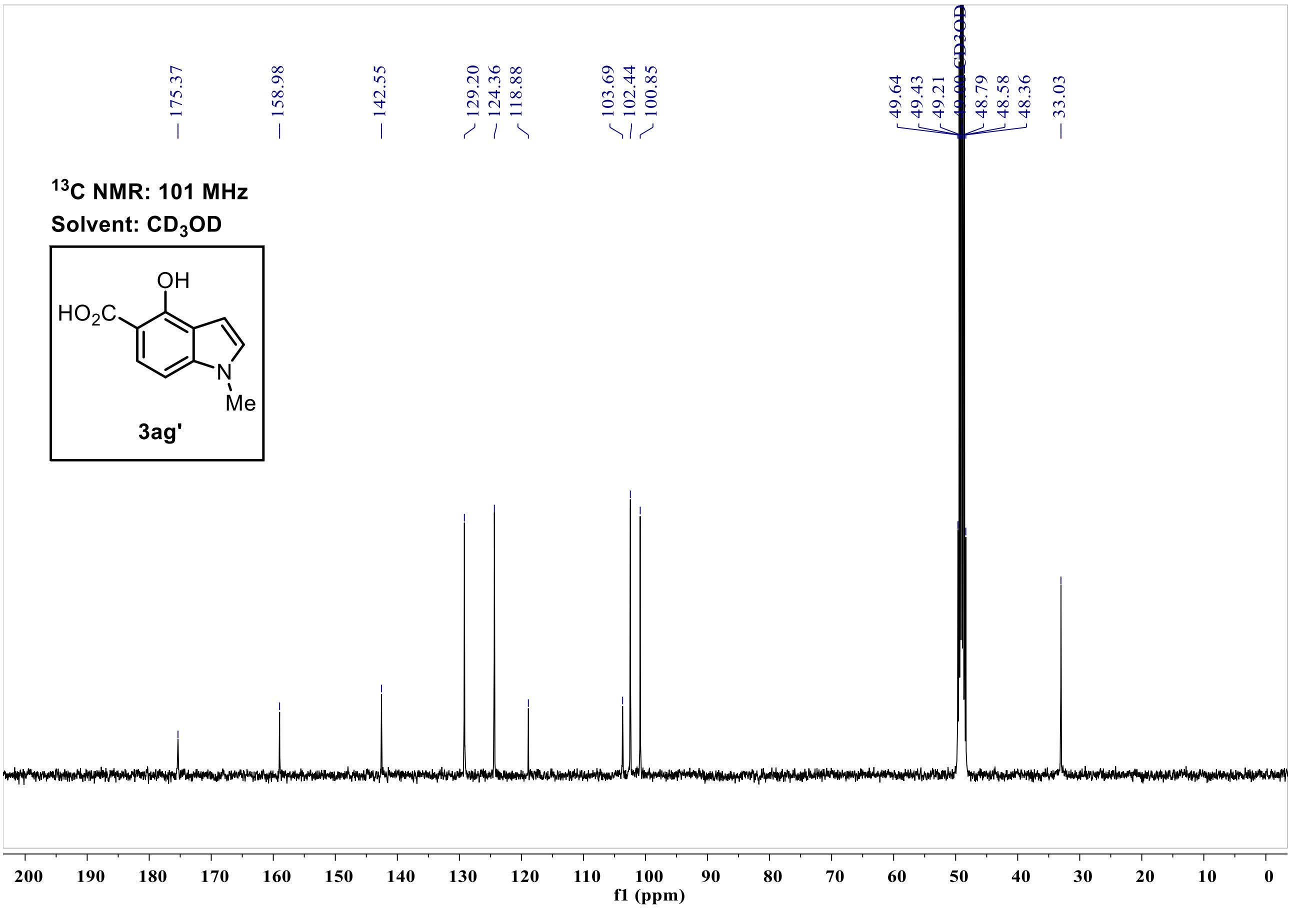

13C NMR: 101 MHz
Solvent: CD3OD
OH
HO2C
N
Me
3ag'
175.37
158.98
142.55
129.20
124.36
118.88
103.69
102.44
100.85
49.64
49.43
49.21
48.79
48.58
48.36
33.03
200 190 180 170 160 150 140 130 120 110 100 90 80 70 60 50 40 30 20 10 0
f1 (ppm)

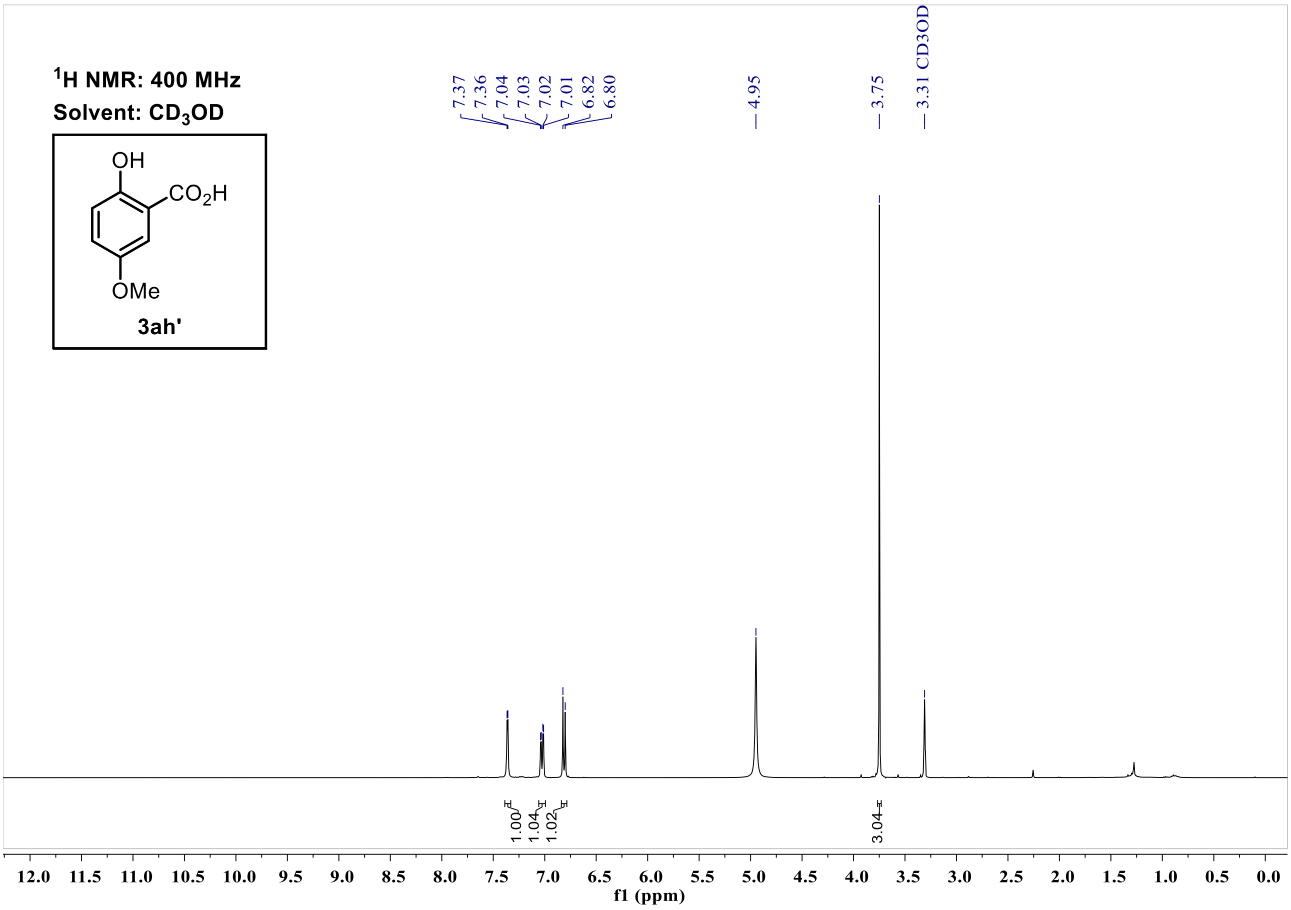

1H NMR: 400 MHz
Solvent: CD3OD
OH
CO2H
OMe
3ah'
7.37
7.36
7.04
7.03
7.02
7.01
6.82
6.80
4.95
3.75
3.31 CD3OD
1.00
1.04
1.02
3.04
f1 (ppm)

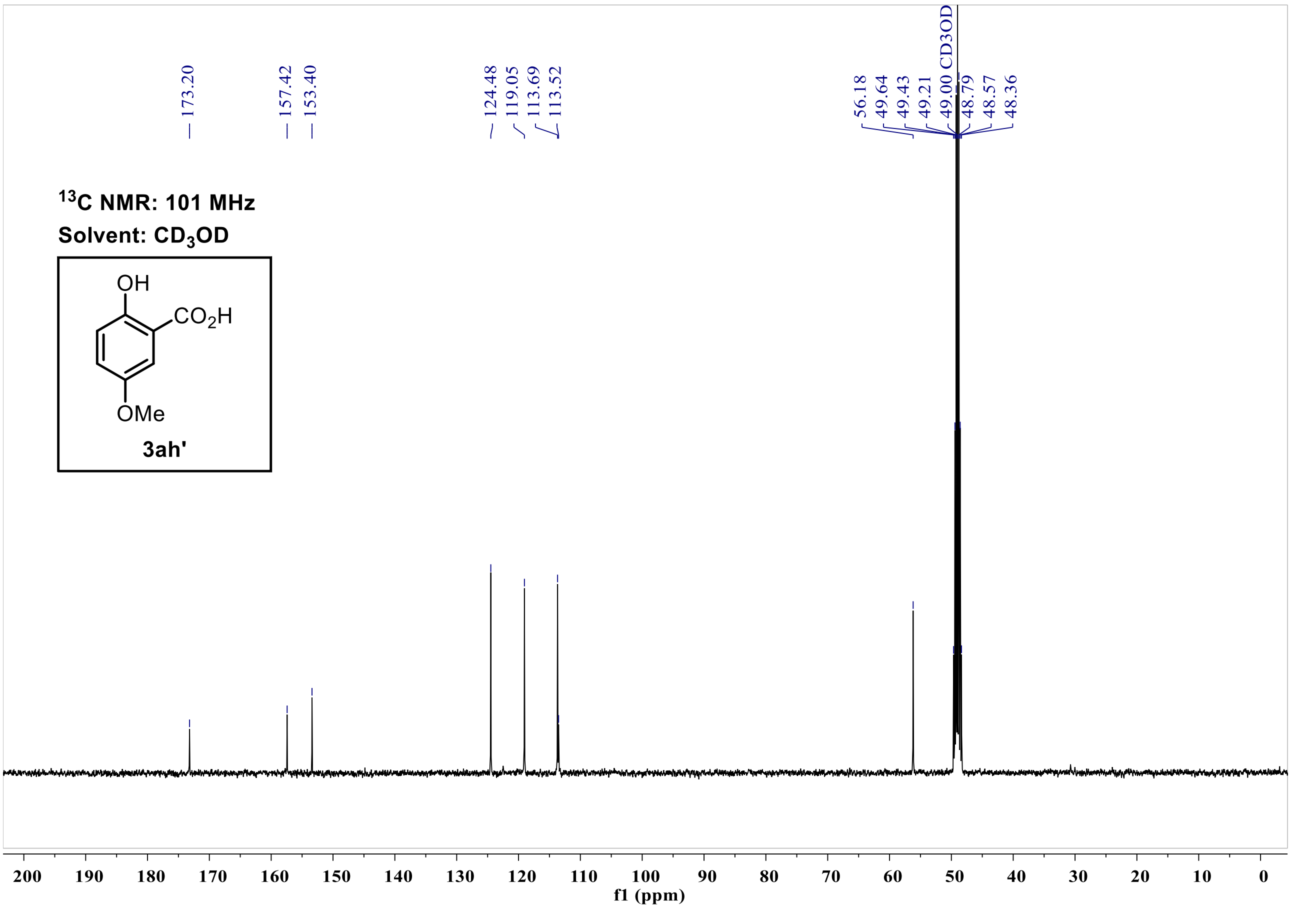

13C NMR: 101 MHz
Solvent: CD3OD
OH
CO2H
OMe
3ah'
173.20
157.42
153.40
124.48
119.05
113.69
113.52
56.18
49.64
49.43
49.21
49.00 CD3OD
48.79
48.57
48.36
f1 (ppm)

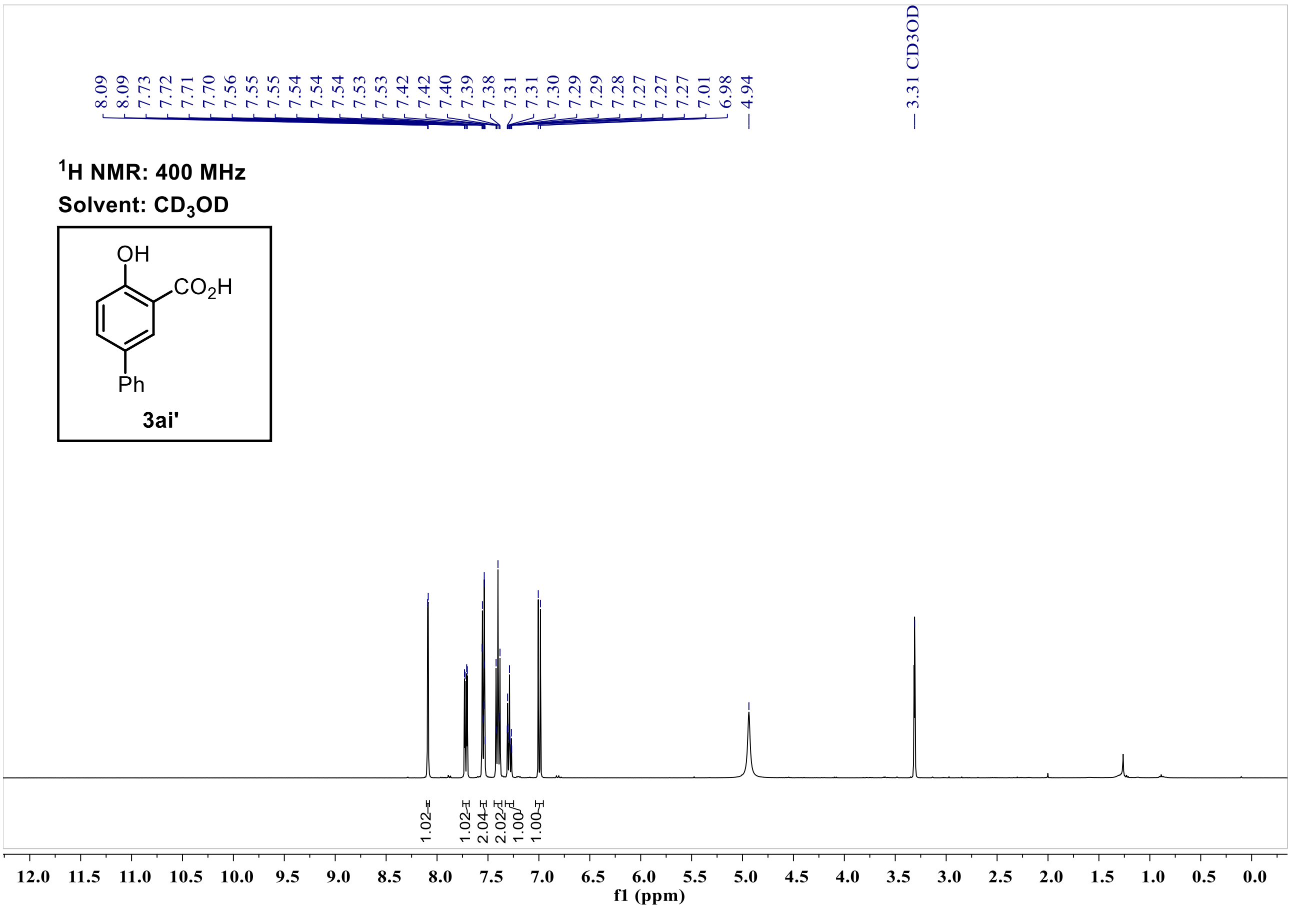
8.09
8.09
7.73
7.72
7.71
7.70
7.56
7.55
7.55
7.54
7.54
7.54
7.53
7.53
7.42
7.42
7.40
7.39
7.38
7.31
7.31
7.30
7.29
7.29
7.28
7.27
7.27
7.27
7.01
6.98
4.94
3.31 CD3OD
1H NMR: 400 MHz
Solvent: CD3OD
OH
CO2H
Ph
3ai'
1.02
1.02
2.04
2.02
1.00
1.00
12.0 11.5 11.0 10.5 10.0 9.5 9.0 8.5 8.0 7.5 7.0 6.5 6.0 5.5 5.0 4.5 4.0 3.5 3.0 2.5 2.0 1.5 1.0 0.5 0.0
f1 (ppm)

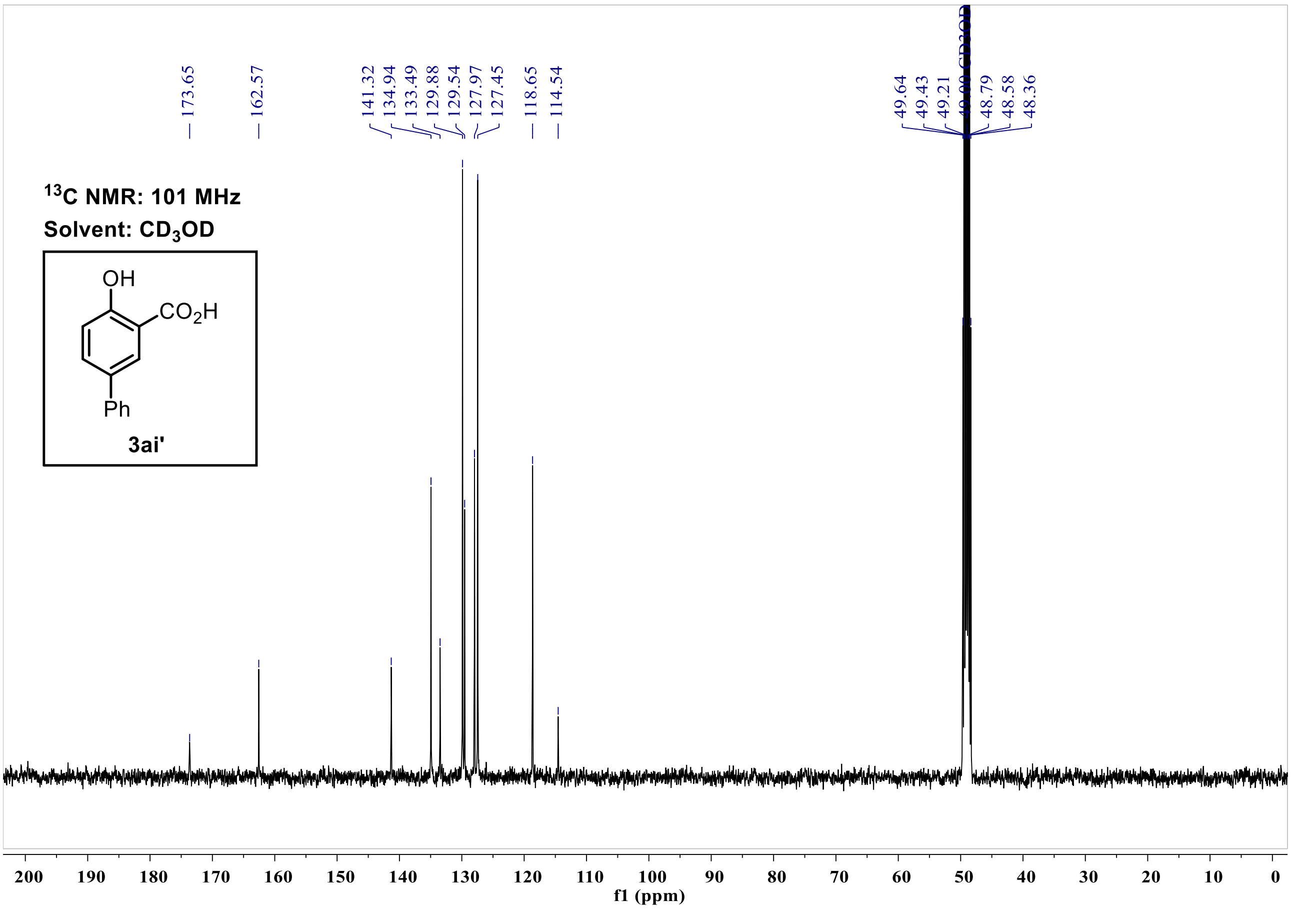
173.65
162.57
141.32
134.94
133.49
129.88
129.54
127.97
127.45
118.65
114.54
49.64
49.43
49.21
48.79
48.58
48.36
13C NMR: 101 MHz
Solvent: CD3OD
OH
CO2H
Ph
3ai'
200 190 180 170 160 150 140 130 120 110 100 90 80 70 60 50 40 30 20 10 0
f1 (ppm)

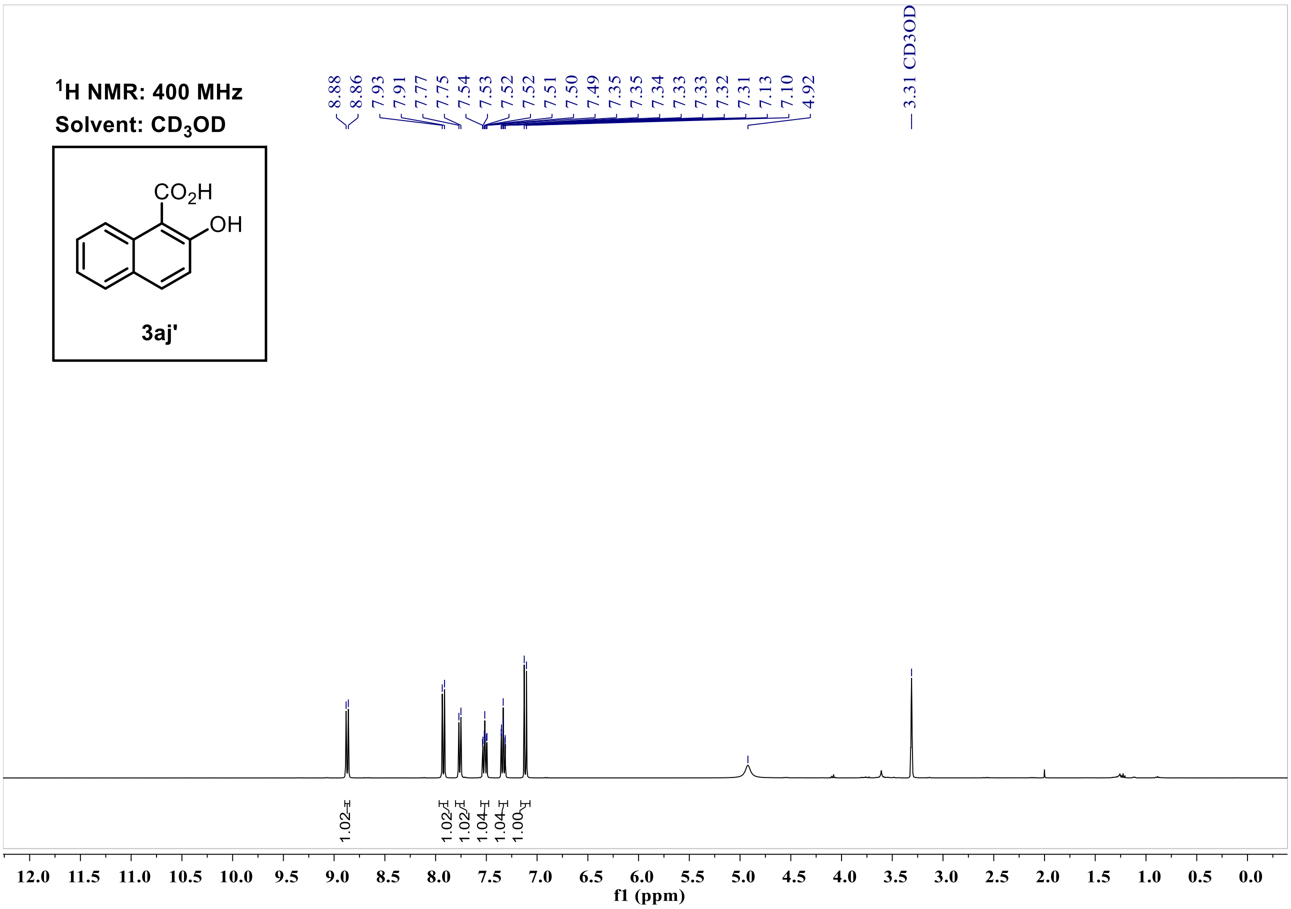
1H NMR: 400 MHz
Solvent: CD3OD
CO2H
OH
3aj'
8.88
8.86
7.93
7.91
7.77
7.75
7.54
7.53
7.52
7.52
7.51
7.50
7.49
7.35
7.35
7.34
7.33
7.33
7.32
7.31
7.13
7.10
4.92
3.31 CD3OD
1.02
1.02
1.02
1.04
1.04
1.00
12.0 11.5 11.0 10.5 10.0 9.5 9.0 8.5 8.0 7.5 7.0 6.5 6.0 5.5 5.0 4.5 4.0 3.5 3.0 2.5 2.0 1.5 1.0 0.5 0.0
f1 (ppm)

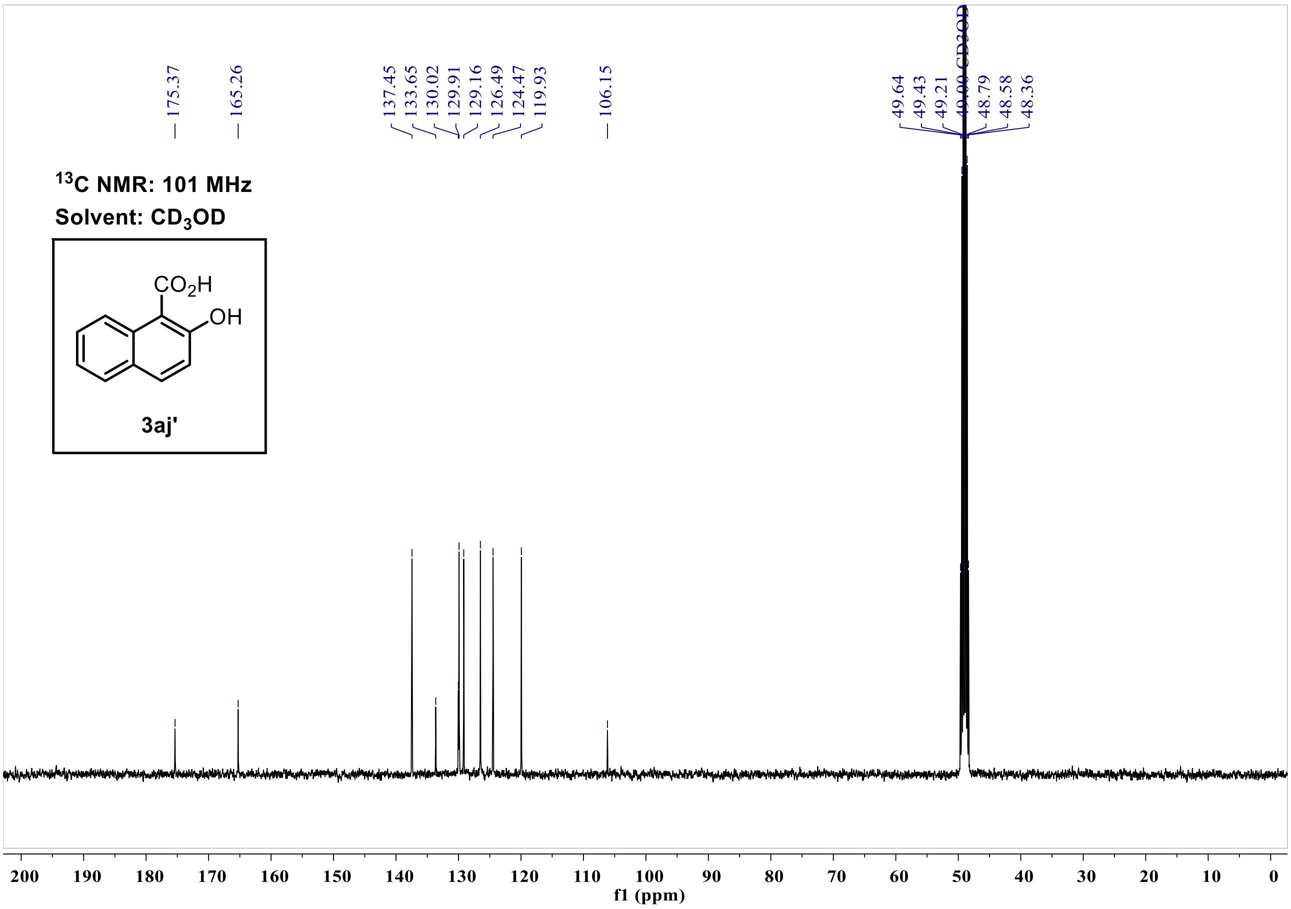
13C NMR: 101 MHz
Solvent: CD3OD
CO2H
OH
3aj'
175.37
165.26
137.45
133.65
130.02
129.91
129.16
126.49
124.47
119.93
106.15
49.64
49.43
49.21
49.00 CD3OD
48.79
48.58
48.36
200 190 180 170 160 150 140 130 120 110 100 90 80 70 60 50 40 30 20 10 0
f1 (ppm)

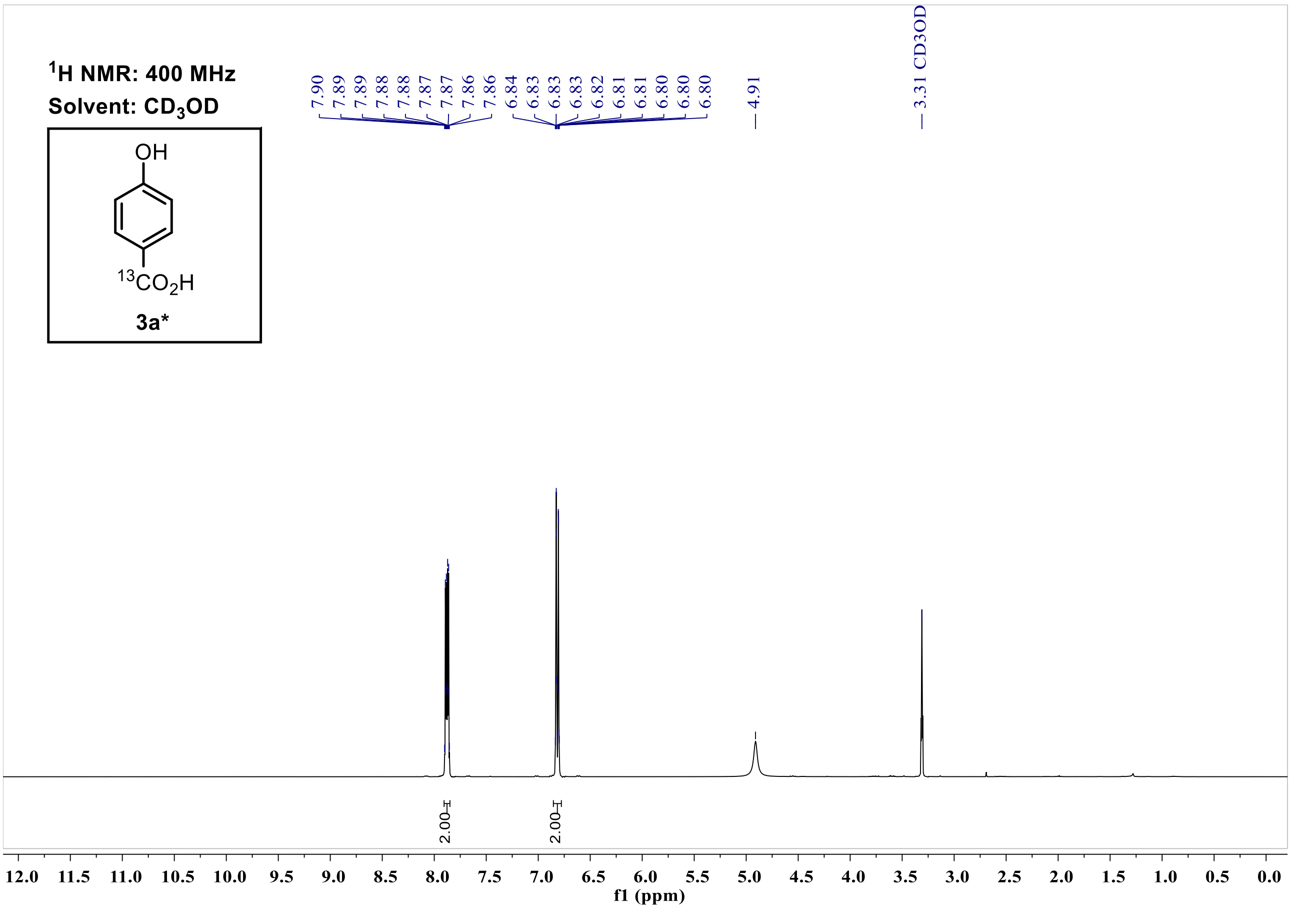

1H NMR: 400 MHz
Solvent: CD3OD
OH
13CO2H
3a*
7.90
7.89
7.89
7.88
7.88
7.87
7.87
7.86
7.86
6.84
6.83
6.83
6.83
6.82
6.81
6.81
6.80
6.80
6.80
4.91
3.31 CD3OD
2.00
2.00
12.0 11.5 11.0 10.5 10.0 9.5 9.0 8.5 8.0 7.5 7.0 6.5 6.0 5.5 5.0 4.5 4.0 3.5 3.0 2.5 2.0 1.5 1.0 0.5 0.0
f1 (ppm)

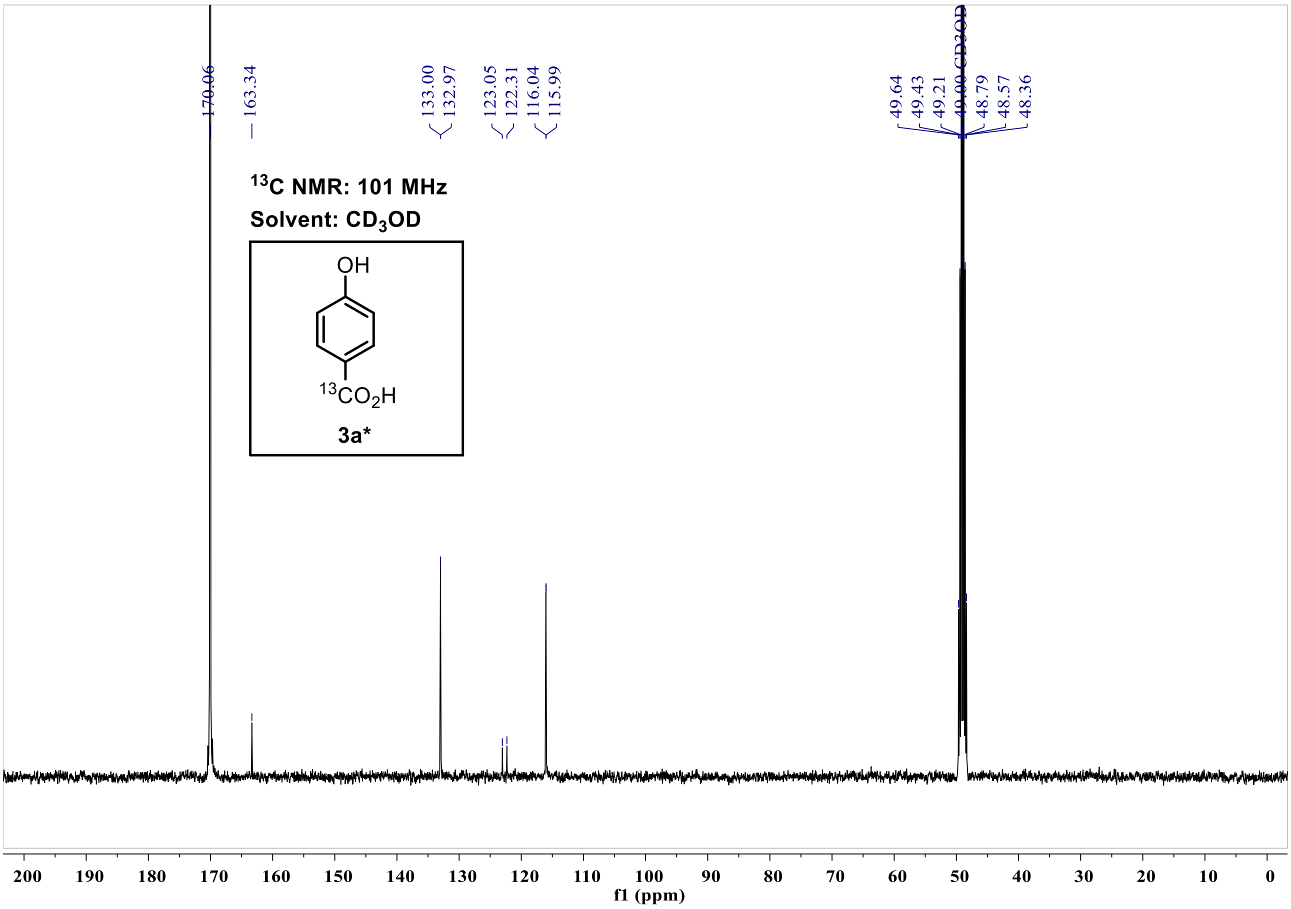

170.06
163.34
133.00
132.97
123.05
122.31
116.04
115.99
49.64
49.43
49.21
49.00 CD3OD
48.79
48.57
48.36
13C NMR: 101 MHz
Solvent: CD3OD
OH
13CO2H
3a*
200 190 180 170 160 150 140 130 120 110 100 90 80 70 60 50 40 30 20 10 0
f1 (ppm)

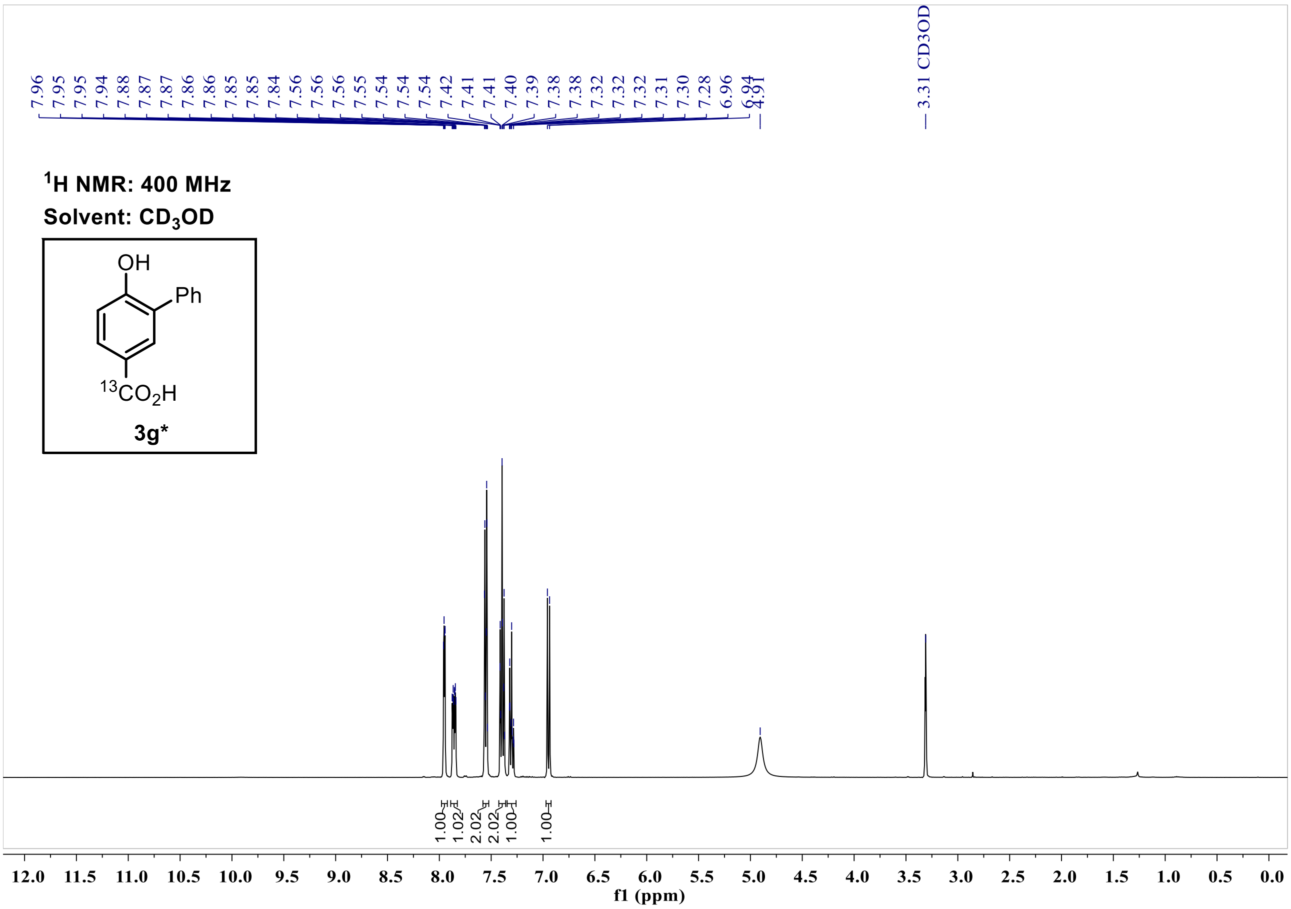
7.96 7.95 7.95 7.94 7.88 7.87 7.87 7.86 7.86 7.85 7.85 7.84 7.56 7.56 7.56 7.55 7.54 7.54 7.54 7.42 7.41 7.41 7.40 7.39 7.38 7.38 7.32 7.32 7.32 7.31 7.30 7.28 6.96 6.94 4.91
3.31 CD3OD
1H NMR: 400 MHz
Solvent: CD3OD
OH
Ph
13CO2H
3g*
1.00 1.02 2.02 2.02 1.00 1.00
12.0 11.5 11.0 10.5 10.0 9.5 9.0 8.5 8.0 7.5 7.0 6.5 6.0 5.5 5.0 4.5 4.0 3.5 3.0 2.5 2.0 1.5 1.0 0.5 0.0
f1 (ppm)

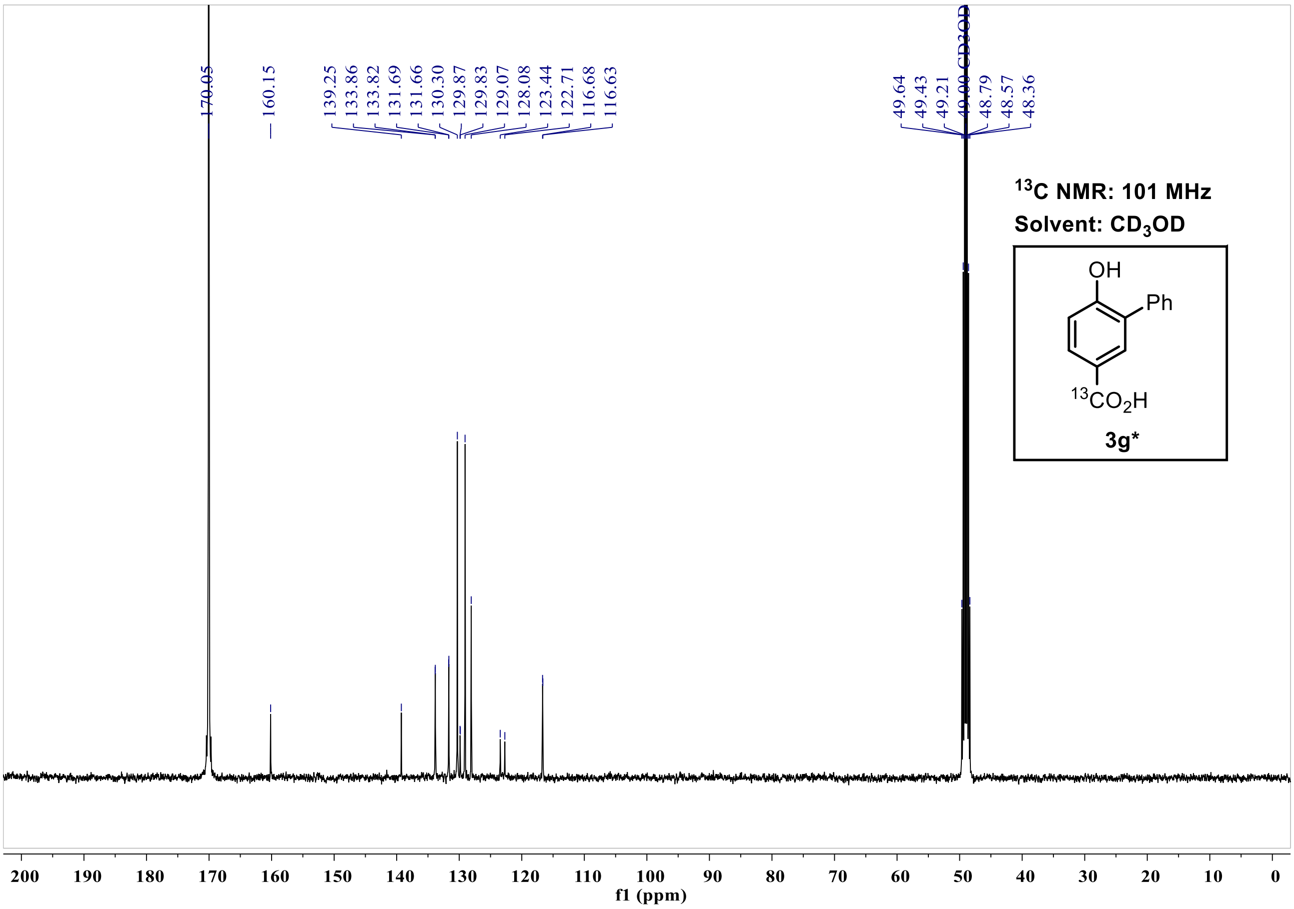
170.05
160.15
139.25 133.86 133.82 131.69 131.66 130.30 129.87 129.83 129.07 128.08 123.44 122.71 116.68 116.63
49.64 49.43 49.21 49.00 CD3OD 48.79 48.57 48.36
13C NMR: 101 MHz
Solvent: CD3OD
OH
Ph
13CO2H
3g*
200 190 180 170 160 150 140 130 120 110 100 90 80 70 60 50 40 30 20 10 0
f1 (ppm)

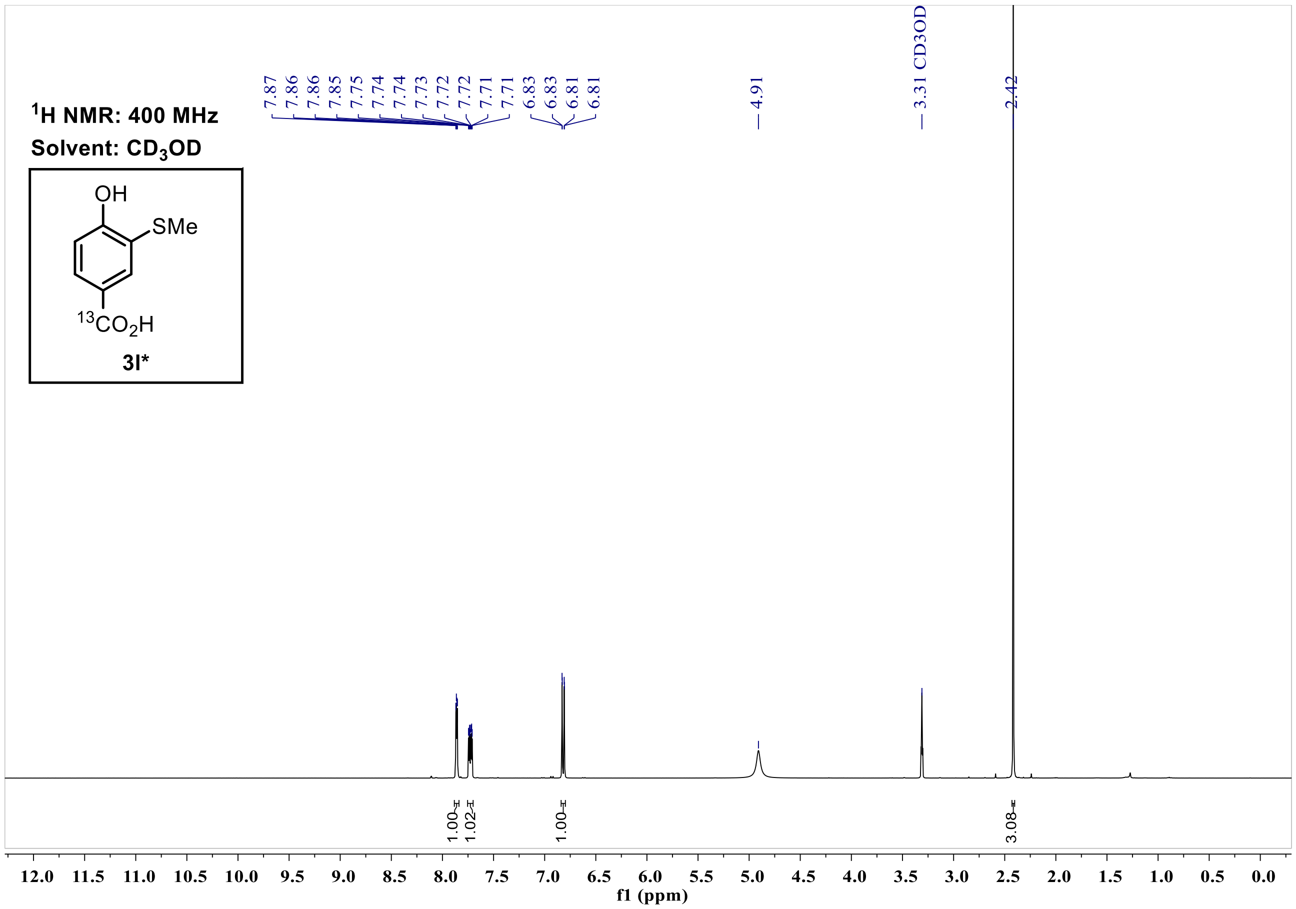
1H NMR: 400 MHz
Solvent: CD3OD
OH
SMe
13CO2H
3l*
7.87
7.86
7.86
7.85
7.75
7.74
7.74
7.73
7.72
7.72
7.71
7.71
6.83
6.83
6.81
6.81
4.91
3.31 CD3OD
2.42
1.00
1.02
1.00
3.08
12.0
11.5
11.0
10.5
10.0
9.5
9.0
8.5
8.0
7.5
7.0
6.5
6.0
5.5
5.0
4.5
4.0
3.5
3.0
2.5
2.0
1.5
1.0
0.5
0.0
f1 (ppm)

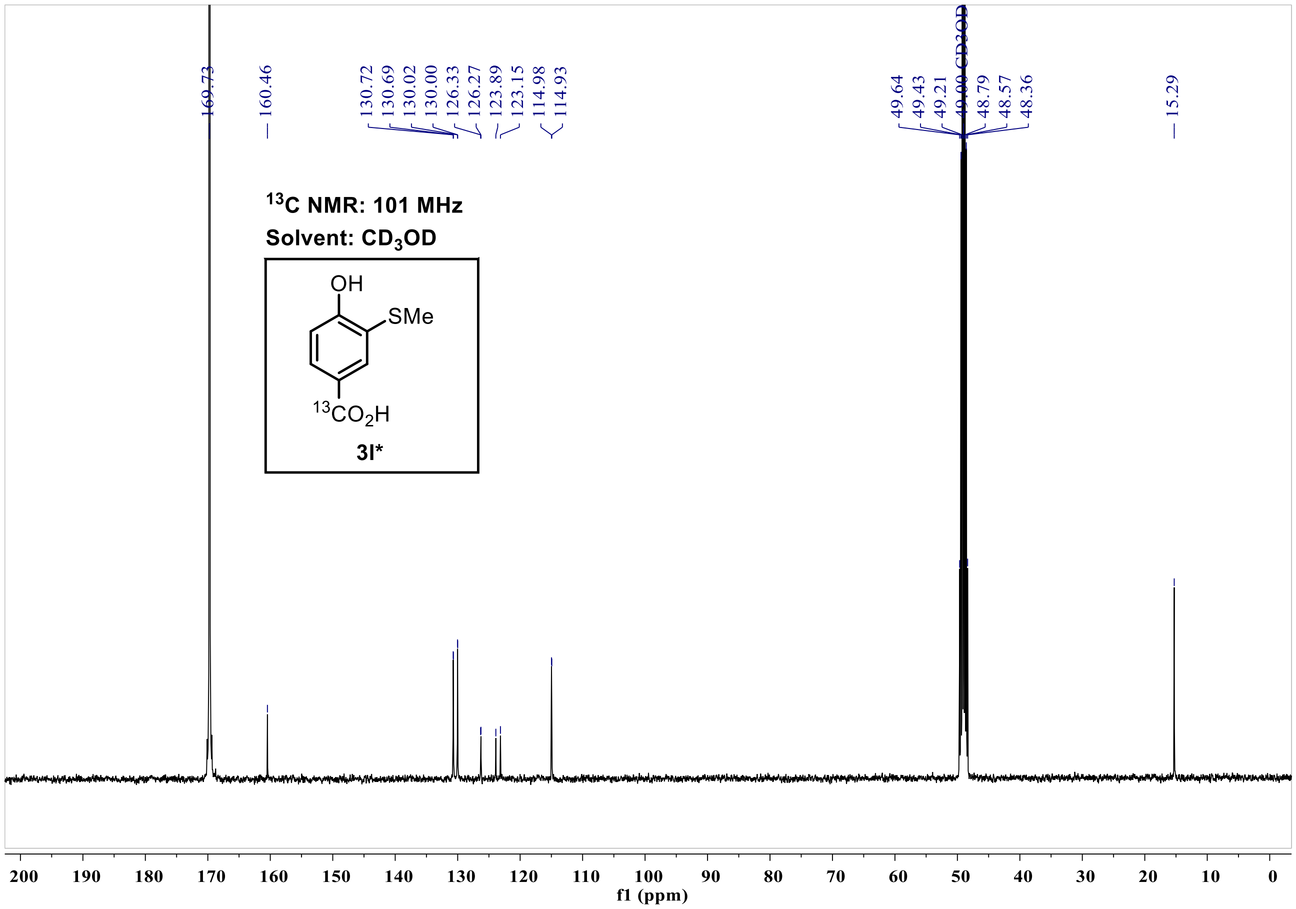
169.73
160.46
130.72
130.69
130.02
130.00
126.33
126.27
123.89
123.15
114.98
114.93
49.64
49.43
49.21
49.00 CD3OD
48.79
48.57
48.36
15.29
13C NMR: 101 MHz
Solvent: CD3OD
OH
SMe
13CO2H
3l*
200
190
180
170
160
150
140
130
120
110
100
90
80
70
60
50
40
30
20
10
0
f1 (ppm)

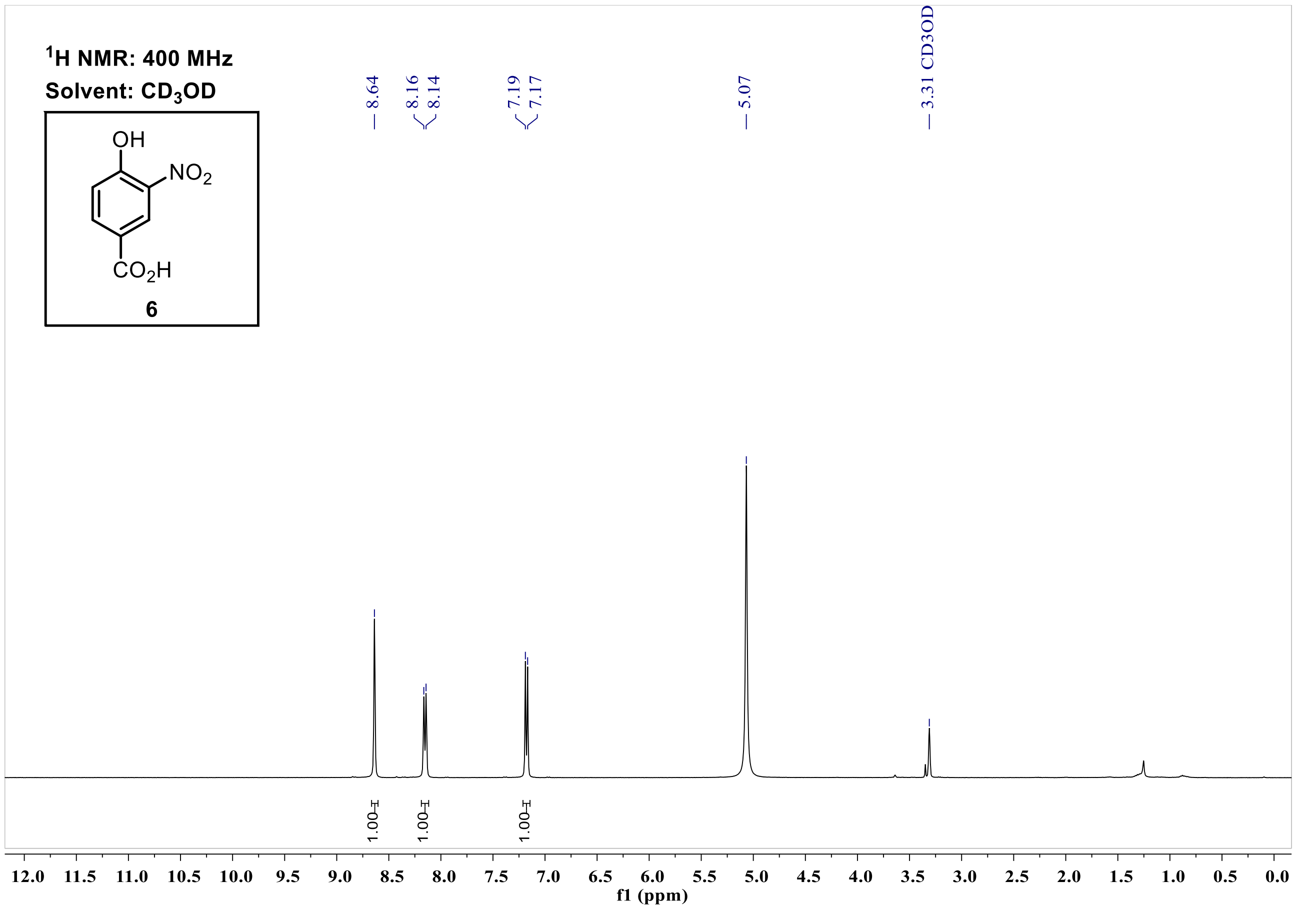

^{1}H NMR: 400 MHz
Solvent: CD_3OD
OH
NO_2
CO_2H
6
8.64
8.16
8.14
7.19
7.17
5.07
3.31 CD3OD
1.00
1.00
1.00
12.0 11.5 11.0 10.5 10.0 9.5 9.0 8.5 8.0 7.5 7.0 6.5 6.0 5.5 5.0 4.5 4.0 3.5 3.0 2.5 2.0 1.5 1.0 0.5 0.0
f1 (ppm)

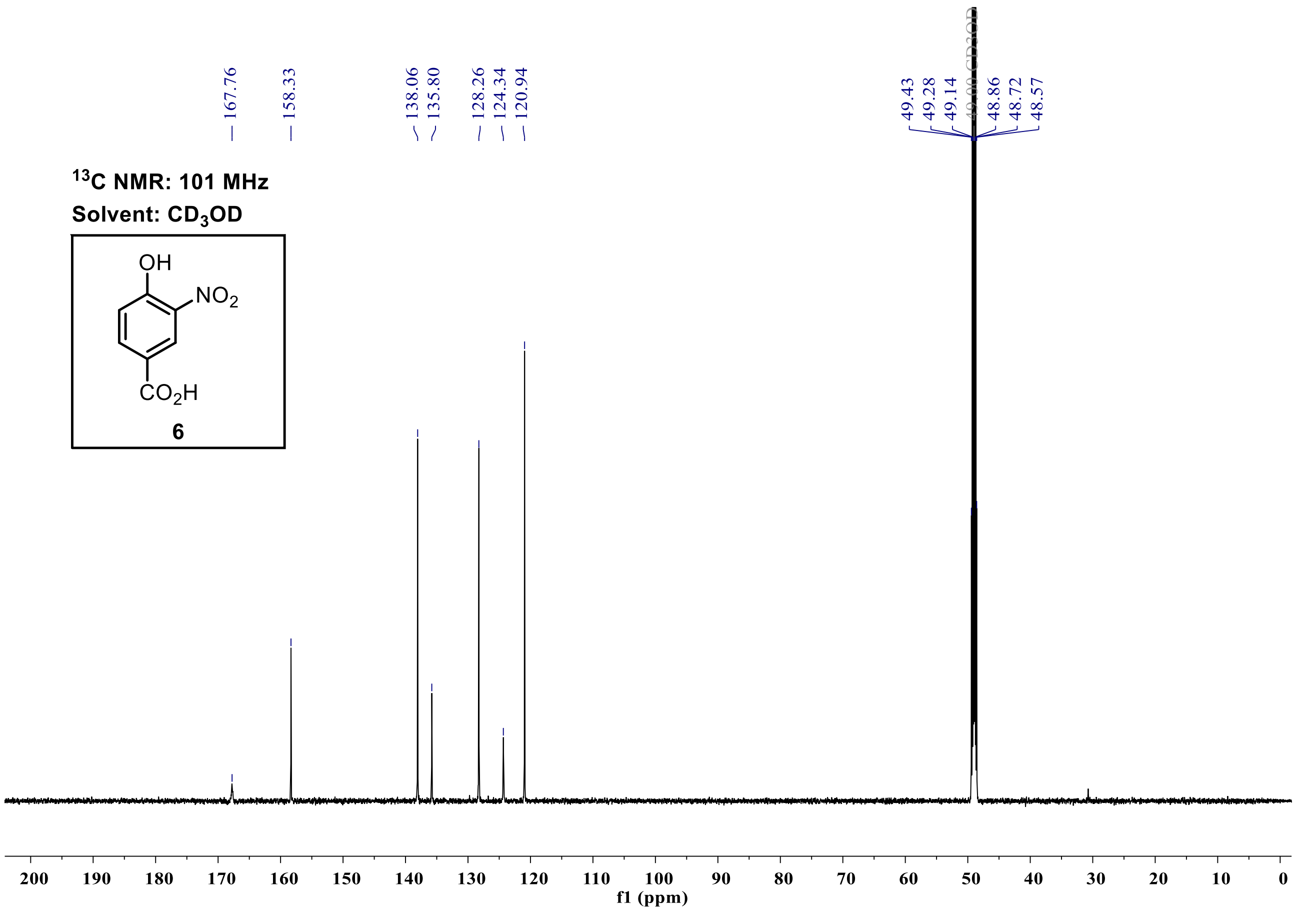

167.76
158.33
138.06
135.80
128.26
124.34
120.94
49.43
49.28
49.14
48.86
48.72
48.57
^{13}C NMR: 101 MHz
Solvent: CD_3OD
OH
NO_2
CO_2H
6
200 190 180 170 160 150 140 130 120 110 100 90 80 70 60 50 40 30 20 10 0
f1 (ppm)

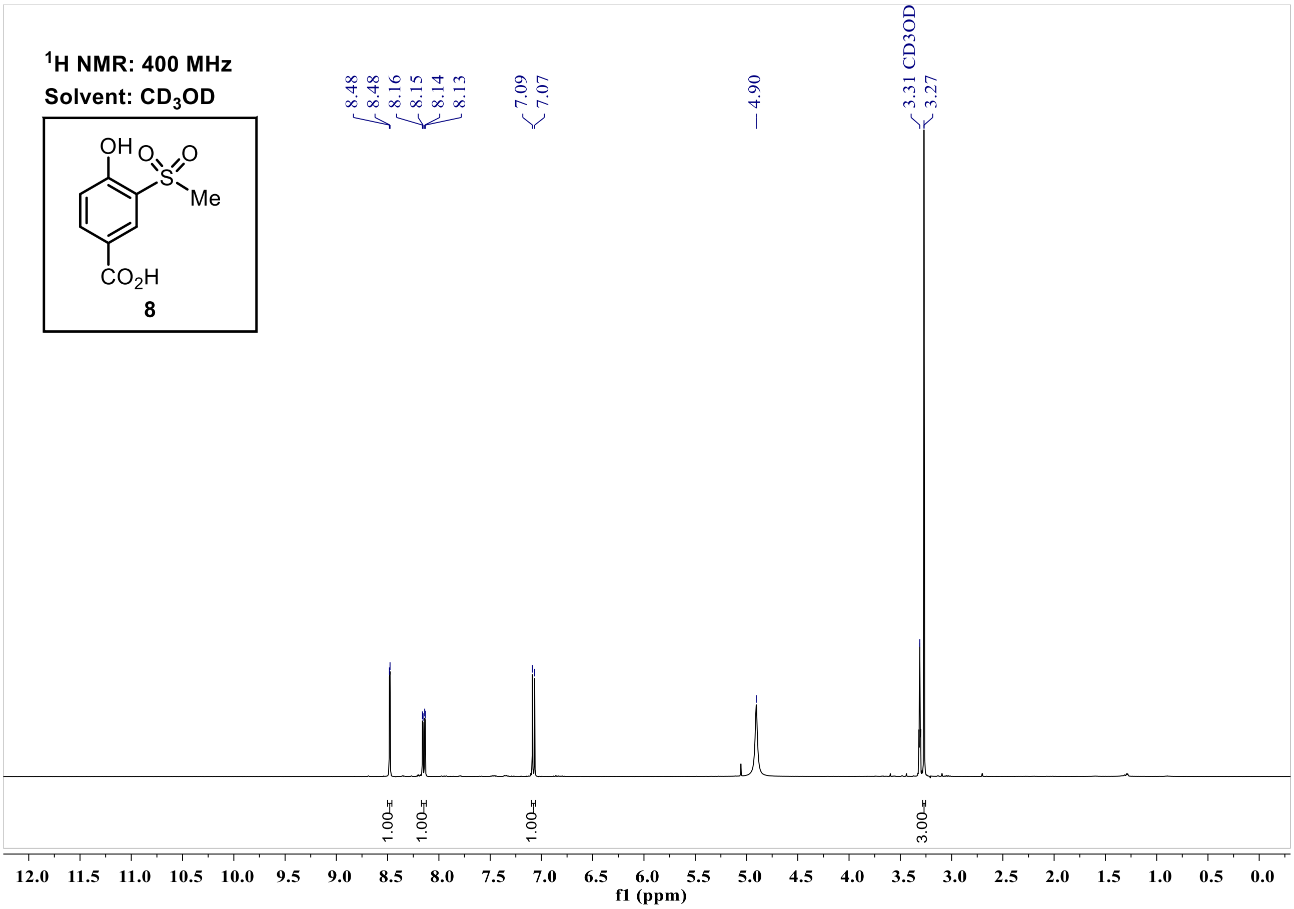

1H NMR: 400 MHz
Solvent: CD3OD
OH
O
S
O
Me
CO2H
8
8.48
8.48
8.16
8.15
8.14
8.13
7.09
7.07
4.90
3.31 CD3OD
3.27
1.00
1.00
1.00
3.00
12.0 11.5 11.0 10.5 10.0 9.5 9.0 8.5 8.0 7.5 7.0 6.5 6.0 5.5 5.0 4.5 4.0 3.5 3.0 2.5 2.0 1.5 1.0 0.5 0.0
f1 (ppm)

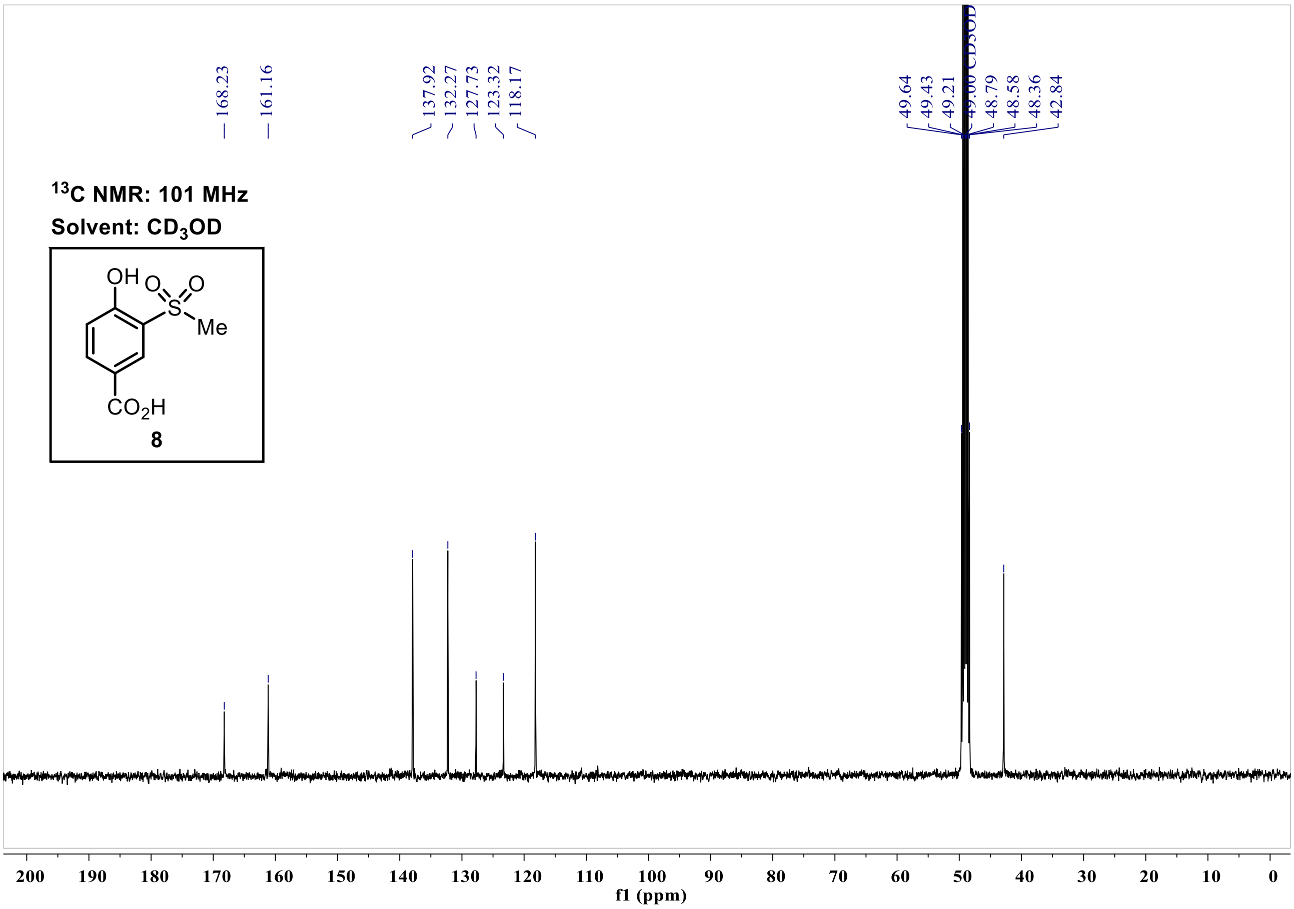

13C NMR: 101 MHz
Solvent: CD3OD
OH
O
S
O
Me
CO2H
8
168.23
161.16
137.92
132.27
127.73
123.32
118.17
49.64
49.43
49.21
49.00 CD3OD
48.79
48.58
48.36
42.84
200 190 180 170 160 150 140 130 120 110 100 90 80 70 60 50 40 30 20 10 0
f1 (ppm)

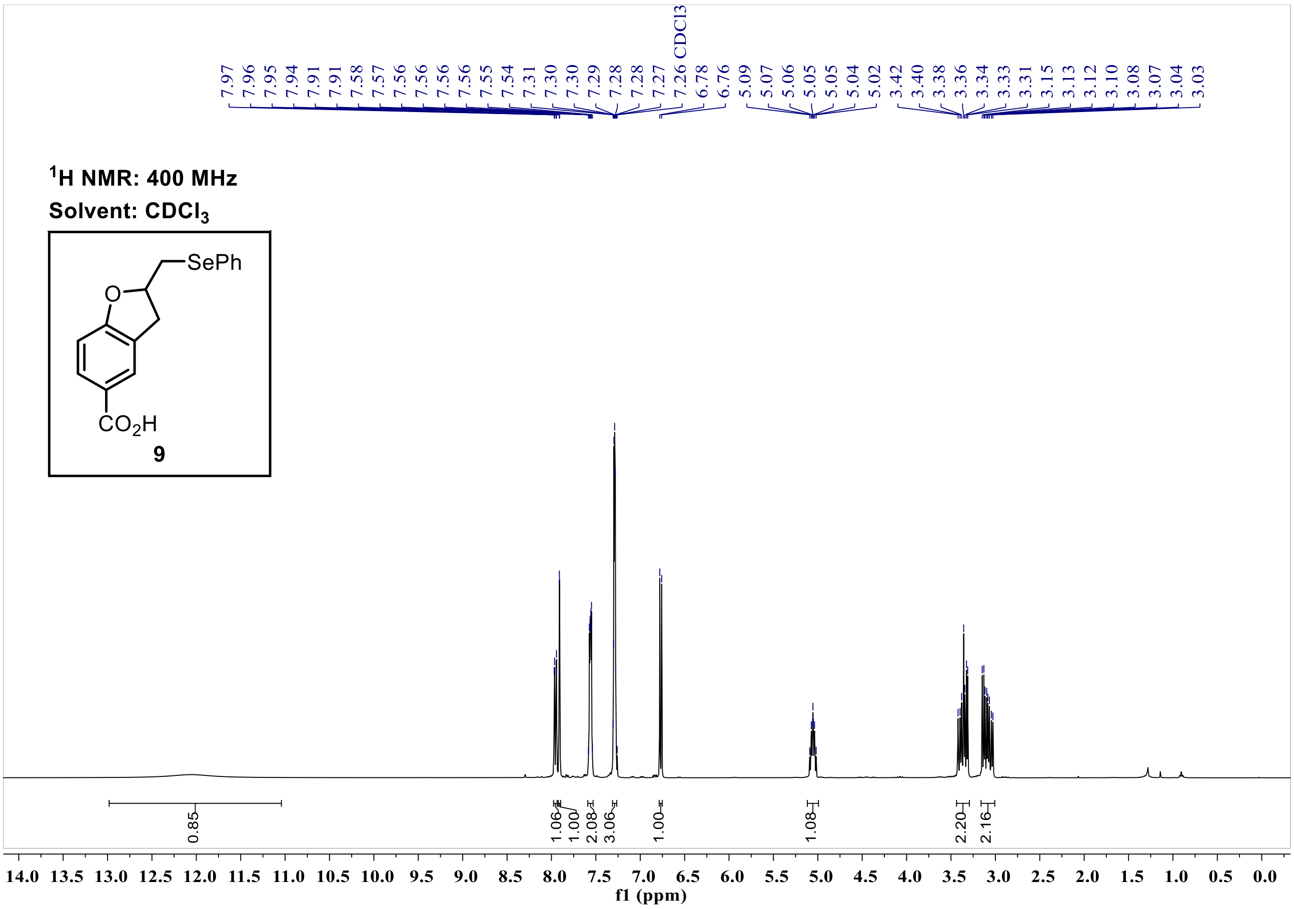
7.97 7.96 7.95 7.94 7.91 7.91 7.58 7.57 7.56 7.56 7.56 7.56 7.55 7.54 7.31 7.30 7.30 7.29 7.28 7.28 7.27 7.26 CDCl3 6.78 6.76 5.09 5.07 5.06 5.05 5.05 5.04 5.02 3.42 3.40 3.38 3.36 3.34 3.33 3.31 3.15 3.13 3.12 3.10 3.08 3.07 3.04 3.03
^{1}H NMR: 400 MHz
Solvent: $CDCl_3$
SePh
O
CO_2H
9
0.85
1.06
1.00
2.08
3.06
1.00
1.08
2.20
2.16
14.0 13.5 13.0 12.5 12.0 11.5 11.0 10.5 10.0 9.5 9.0 8.5 8.0 7.5 7.0 6.5 6.0 5.5 5.0 4.5 4.0 3.5 3.0 2.5 2.0 1.5 1.0 0.5 0.0
f1 (ppm)

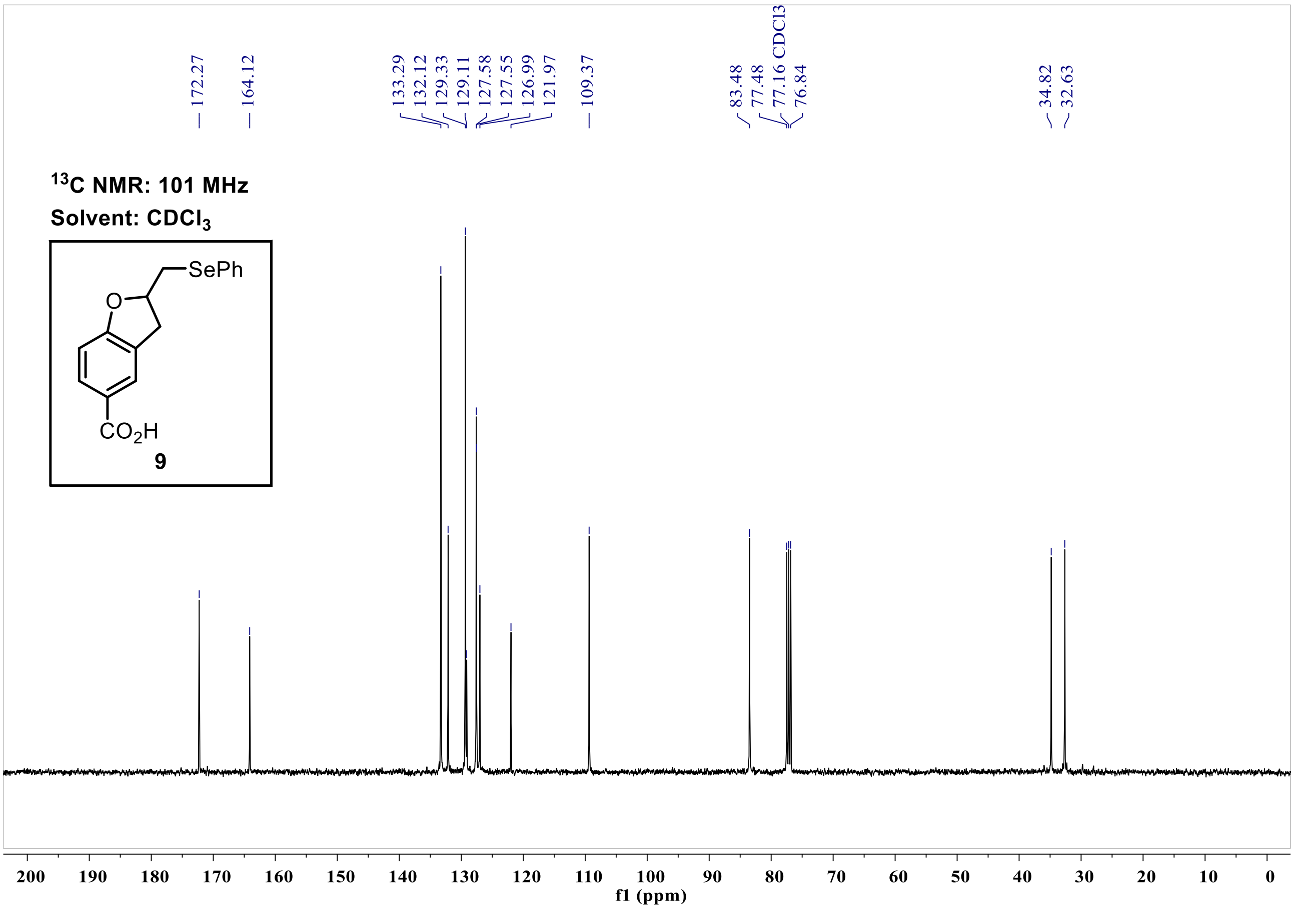
172.27 164.12 133.29 132.12 129.33 129.11 127.58 127.55 126.99 121.97 109.37 83.48 77.48 77.16 CDCl3 76.84 34.82 32.63
^{13}C NMR: 101 MHz
Solvent: $CDCl_3$
SePh
O
CO_2H
9
200 190 180 170 160 150 140 130 120 110 100 90 80 70 60 50 40 30 20 10 0
f1 (ppm)
